# MetaVelvet-SL: an extension of the Velvet assembler to a *de novo* metagenomic assembler utilizing supervised learning

**DOI:** 10.1093/dnares/dsu041

**Published:** 2014-11-27

**Authors:** Afiahayati, Kengo Sato, Yasubumi Sakakibara

**Affiliations:** Department of Biosciences and Informatics, Keio University, 3-14-1 Hiyoshi, Kohoku-ku, Yokohama 223-8522, Japan

**Keywords:** metagenomic, *de novo* assembler, short read, supervised learning, microbial community

## Abstract

The assembly of multiple genomes from mixed sequence reads is a bottleneck in metagenomic analysis. A single-genome assembly program (assembler) is not capable of resolving metagenome sequences, so assemblers designed specifically for metagenomics have been developed. MetaVelvet is an extension of the single-genome assembler Velvet. It has been proved to generate assemblies with higher N50 scores and higher quality than single-genome assemblers such as Velvet and SOAPdenovo when applied to metagenomic sequence reads and is frequently used in this research community. One important open problem for MetaVelvet is its low accuracy and sensitivity in detecting chimeric nodes in the assembly (de Bruijn) graph, which prevents the generation of longer contigs and scaffolds. We have tackled this problem of classifying chimeric nodes using supervised machine learning to significantly improve the performance of MetaVelvet and developed a new tool, called MetaVelvet-SL. A Support Vector Machine is used for learning the classification model based on 94 features extracted from candidate nodes. In extensive experiments, MetaVelvet-SL outperformed the original MetaVelvet and other state-of-the-art metagenomic assemblers, IDBA-UD, Ray Meta and Omega, to reconstruct accurate longer assemblies with higher N50 scores for both simulated data sets and real data sets of human gut microbial sequences.

## 1. Introduction

Metagenomic research studies genetic material recovered directly from environmental samples. Next-generation sequencing (NGS) technologies have enabled an explosion in sequencing with increased throughput and decreased cost,^1^ which provides opportunities to generate sequence reads from metagenomes effectively covering highly diverse microbial populations, even for genomes with low coverage. An important step in metagenomic analysis is the assembly of multiple genomes from mixed sequence reads of the multiple species that exist in the sample.^2^ This can present problems, because, in a microbial community, the number of genomes and the coverage of each genome are initially unknown and the coverage distribution is inhomogeneous and potentially skewed.^1–8^ Another major difficulty is the short length of sequence reads from next-generation sequencers.^2^

Currently, there are several *de novo* assemblers that attempt to analyse metagenomic data. MAP,^4^ Genovo^5^ and Xgenovo^8^ are used for rather long sequence reads, while MetaVelvet,^2^ Meta-IDBA,^7^ IDBA-UD,^9^ Ray Meta^10^ and Omega^11^ are used for short sequence reads. MAP was designed for the sequence reads produced by Sanger (700–1,000 bp) and 454 sequencing technology (200–500 bp). It uses an improved Overlap-Layout-Consensus (OLC) strategy integrating mate pair information.^4^ Genovo was designed for 454 sequencing data: it is a metagenomic assembler employing a generative probabilistic model.^5^ Xgenovo is an extension of Genovo incorporating paired-end information.^8^ MetaVelvet, Meta-IDBA and IDBA-UD use the de Bruijn graph approach. These assemblers are specifically designed for the huge numbers of short reads generated by Illumina-type next-generation sequencers that enable deep sequencing of the inhomogeneous and divergent species in a microbial community. IDBA-UD is an extension of Meta-IDBA dealing with the uneven sequencing depths of different regions of genomes from different species.^9^ Both MetaVelvet^2^ and IDBA-UD^9^ have been shown to produce longer high-quality assemblies than single-genome assemblers, such as Velvet^12,13^ and SOAPdenovo2.^14^ Ray Meta is an extension of the Ray assembler for *de novo* metagenome assembly, which is scalable because it is highly distributed computing.^10^ Omega is a metagenomic assembler using overlap graph approach. Omega was most recently proposed for rather longer Illumina sequencing data of microbial communities.^11^

MetaVelvet^2^ is an extension of a single-genome assembly program (assembler), named Velvet.^12,13^ The fundamental concept used in MetaVelvet is that a de Bruijn graph constructed from mixed sequence reads of multiple species is considered to be equivalent to the union of multiple de Bruijn sub-graphs, each of which is constructed from the sequence reads of individual species. The strategy of MetaVelvet is, first, to decompose a de Bruijn graph constructed from mixed short reads into individual sub-graphs and, second, to assemble scaffolds from each decomposed de Bruijn sub-graph to build an isolated genome.

**Figure 1.**
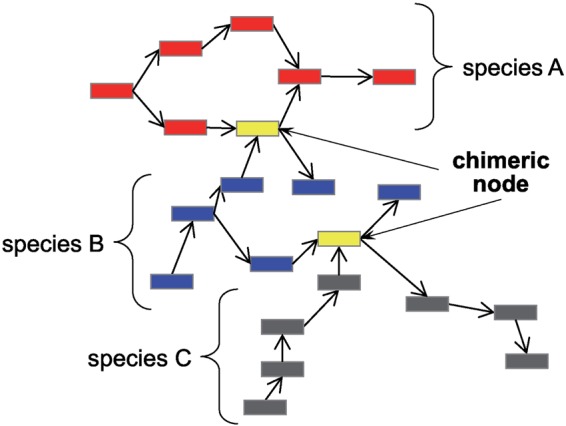
Chimeric nodes need to be split to obtain independent sub-graphs in a metagenomic assembly.

For the graph disconnection task, MetaVelvet identifies nodes shared between two sub-graphs (named chimeric nodes) and disconnects two sub-graphs by splitting the shared nodes, as illustrated in Fig. 1. Chimeric nodes are shared between the genomes of two closely related species and could represent orthologous sequences, conserved sequences (e.g. rRNA sequences) or horizontal transfer sequences.

To identify chimeric nodes, MetaVelvet uses simple heuristics based on coverage difference and paired-end information, which results in low accuracy and low sensitivity. Our primary goal in this study is to improve chimeric node detection and generate longer accurate scaffolds. Such scaffolds can help to extract more information from the reads, leading to the discovery of more genes and better functional annotation.^15^ To do this, we have developed an assembler called MetaVelvet-SL, which classifies every node in a de Bruijn graph constructed from mixed short reads of multiple species into the following four types by employing supervised machine learning.

1. Chimeric node: A node which is shared between the genomes of two closely related species. This node should be split. Chimeric nodes are illustrated in Fig. 1.
2. Repeat node: A repeat node represents a sequence that occurs several times in the genome. Note that, in multiple genome assembly, nodes at a crossing point between two incoming and two outgoing edges are not necessarily repeats. Such nodes are sometimes chimeric nodes.
3. Unique node: A unique node is one which is neither chimeric nor a repeat. The challenge is to determine the unique nodes of each species correctly.
4. Low-coverage node: In metagenomic assembly, all nodes, even those with low coverage, must be examined to account for species with small populations, but low-coverage nodes must be distinguished from nodes generated by sequencing errors.

The first new procedure in MetaVelvet-SL is to develop the model to classify a node at a crossing point between two paths as chimeric or not. In the process of learning the model, 94 features are extracted for each chimeric node candidate, which is a node at a crossing point that has two incoming edges and two outgoing edges. A Support Vector Machine (SVM) is used for learning the classification model. If chimeric nodes can be identified correctly, it means that the de Bruijn graph can be disconnected appropriately by splitting the chimeric nodes. The second new procedure in MetaVelvet-SL is that the expected coverage to extract the unique nodes is calculated for each sub-graph. Based on the assumption that each sub-graph represents a single species, the expected coverage per sub-graph can precisely determine the unique nodes of each species, even those with low coverage. This expected coverage calculation per sub-graph replaces the original MetaVelvet strategy of detecting multiple peaks on the histograms of *k*-mer frequencies and defining each peak as expected coverage.

MetaVelvet-SL consists of two main modules: first, the supervised learning module to develop a model for the classification of chimeric nodes and, second, the assembly module. (The technical details are described in the Materials and methods section.) MetaVelvet-SL also provides a couple of tools to allow users to generate their classification model using prior knowledge about the taxonomic profile of the target microbial community. The taxonomic profile can be inferred from sequence reads by using taxonomic profiling methods, such as MetaPhlAn.^16^ We have developed a pipeline connecting MetaPhlAn and MetaVelvet-SL. The pipeline automatically generates a classification model only from metagenomic sequence read data. This customized classification model could be well suited to the assembly of the target metagenomes. MetaVelvet-SL also provides a library of pre-trained classification models for several typical environments, such as soil, deep sea, mud, human blood, intestine and mouth.

The source code of MetaVelvet-SL, the pipeline connecting MetaPhlAn and MetaVelvet-SL, and the library of classification models for several typical environments are freely available under the GNU General Public License at http://metavelvet.dna.bio.keio.ac.jp.

We conducted computational experiments to evaluate the performance of Metavelvet-SL. The assembly performance of Metavelvet-SL was compared with those of MetaVelvet, the state-of-the-art metagenomic assemblers IDBA-UD, Ray Meta and Omega, and a standard single-genome assembler for massive short sequencing reads, SOAPdenovo2. For simulated data sets, first, we conducted experiments using the pipeline connecting MetaPhlAn and MetaVelvet-SL. The taxonomic profile for the training data set was inferred by MetaPhlAn. Second, to measure the performance of MetaVelvet-SL for different degrees of similarity between the training data set and the assembly data set, we conducted experiments using three levels of training data sets from the highest to the lowest similarity (genus, family and order) to the assembly data sets. For all assembly data sets, MetaVelvet-SL with any training data set generated the highest accurate N50 scores and longest maximum length of accurate scaffolds among the assemblers. (The N50 score is a standard statistical measure that evaluates assembly quality. Scaffolds with higher N50 scores are especially beneficial for the identification of protein-coding genes.^2^) On real data sets of human gut microbial short read data, sequenced as part of the MetaHIT project^17^ and the Human Microbiome Project Consortium,^18^ MetaVelvet-SL using models constructed by supervised learning from the taxonomy profile inferred by MetaPhlAn generated longer scaffolds.

## 2. Materials and methods

A de Bruijn graph is a data structure that compactly represents overlaps between short reads. Several *de novo* methods based on de Bruijn graphs have been proposed to assemble short reads generated from next-generation sequencers for single genomes and metagenomes.^2,7,9,12,13^ In a de Bruijn graph, a *k*-mer (word of length *k*) is assigned to a node, so the size of a de Bruijn graph is independent of the size of the input of reads. The assembly (reconstruction) of the target genome from the de Bruijn graph can be reduced to finding an Eulerian path that is computable in polynomial time.

First, we briefly review the Velvet and MetaVelvet assemblers upon which our method is based. Then we describe MetaVelvet-SL, our extension of Velvet to metagenomic assembly, that utilizes supervised learning.

### 2.1. Brief outline of Velvet and MetaVelvet

Velvet is slightly different from other de Bruijn-graph-based assemblers in that each node is attached to a twin node that represents a series of *k*-mers and their reverse complements for reads from both strands. For each input read, Velvet defines an ordered set of overlapping *k*-mers. The ordered set is cut whenever an overlap with another read begins or ends. For each uninterrupted ordered subset of the original *k*-mers, a node is created. Velvet has three functions to manipulate the de Bruijn graph: node merging for simplification, removing tips and removing bubbles for error reduction. Velvet has two functions, Pebble and Rock Band, for constructing the scaffold and for repeat resolution using paired-end and long-read information. In these functions, Velvet distinguishes the unique nodes from the repeat nodes based on the node coverage. A repeat node represents a sequence that occurs several times in the genome and can be described as a node at a crossing point between two paths with multiple incoming and outgoing edges. In multiple genome assembly, such nodes are not necessarily repeats since they can sometimes be shared between the genomes of two closely related species and represent orthologous sequences, conserved sequences (such as rRNA sequences) or horizontal transfer sequences.

MetaVelvet is an extension of Velvet for the assembly of metagenomes. The fundamental concept used in MetaVelvet is that a de Bruijn graph constructed from mixed sequence reads of multiple species is considered to be equivalent to the union of multiple de Bruijn sub-graphs, each of which is constructed from sequence reads of individual species. MetaVelvet has two functions. First, MetaVelvet decomposes a de Bruijn graph constructed from mixed short reads into individual sub-graphs. To do this, MetaVelvet calculates the histogram of *k*-mer frequencies and detects multiple peaks on the histogram, each peak of which would correspond to one species in a microbial community. Then, MetaVelvet classifies every node into one of the peaks to form sub-graphs composed of nodes belonging to the same peak. MetaVelvet identifies shared (chimeric) nodes between two sub-graphs and disconnects the sub-graphs by splitting the shared nodes, as illustrated in Fig. 1. To distinguish chimeric nodes from repeat nodes, MetaVelvet uses coverage difference and paired-end information. The second function of MetaVelvet builds scaffolds using Velvet's Pebble and Rock Band functions by treating each decomposed de Bruijn sub-graph as an isolated species genome.

### 2.2. Extension to MetaVelvet-SL

**Figure 2.**
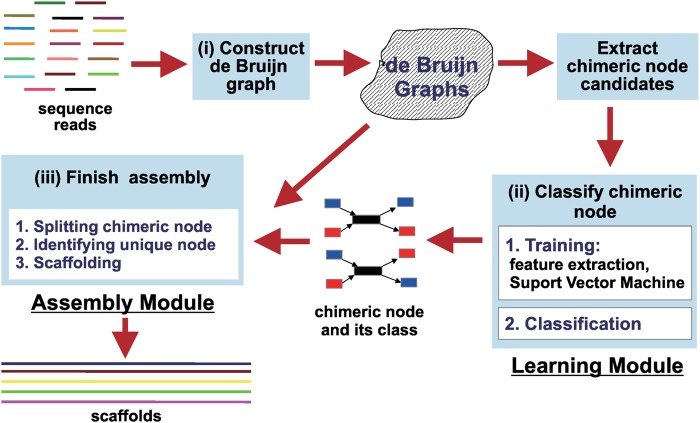
MetaVelvet-SL system consists of three major procedures: (i) construction of a de Bruijn graph; (ii) classification of chimeric nodes and (iii) final assembly tasks.

MetaVelvet-SL consists of three major procedures, as illustrated in Fig. 2.

**Figure 3.**
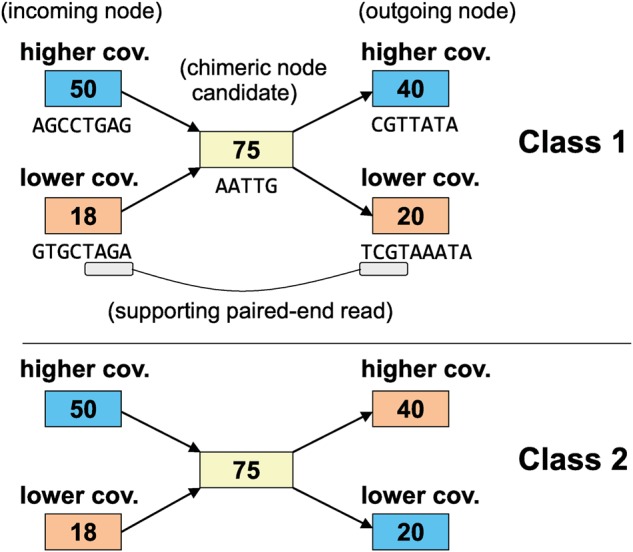
Chimeric nodes fall into two classes. Nodes of the same colour represent the same species. The number in each node represents the coverage value of the node. A contig sequence is also attached to each node.

1. Construction of a de Bruijn graph.
  MetaVelvet-SL constructs a de Bruijn graph from mixed sequence reads of multiple species genomes using Velvet functions.
2. Learning and classification of chimeric nodes.
  This procedure, first, extracts the chimeric node candidates from the main de Bruijn graph. A chimeric node candidate is defined as a node that has two incoming edges and two outgoing edges. MetaVelvet-SL utilizes LIBSVM^19^ to develop a model for classification of chimeric nodes. We used the RBF kernel that is recommended by LIBSVM. Grid search was used to find optimal parameters for the RBF kernel. For the classification, 94 features are extracted for each chimeric node candidate. These features are (Fig. 3):
  - The dinucleotide frequencies in the chimeric node candidate, the two incoming nodes and the two outgoing nodes. Each node has 16 dinucleotide frequencies (AA, AT, AC, AG, TA, … , GG). (16 features × 5 = 80 features).
  - The number of paired-end reads supporting the connection between the incoming node with higher coverage and the outgoing node with higher coverage (1 feature).
  - The number of paired-end reads supporting the connection between the incoming node with higher coverage and the outgoing node with lower coverage (1 feature).
  - The number of paired-end reads supporting the connection between the incoming node with lower coverage and the outgoing node with higher coverage (1 feature).
  - The number of paired-end reads supporting the connection between the incoming node with lower coverage and the outgoing node with lower coverage (1 feature).
  - The ratio between the coverage of each incoming node and the coverage of the chimeric node candidate (2 features).
  - The ratio between the coverage of each outgoing node and the coverage of the chimeric node candidate (2 features).
  - The coverage of the chimeric node candidate (1 feature).
  - The lengths of contigs attached to the chimeric node candidate, the two incoming nodes and the two outgoing nodes (5 features).
  There are three defined classes. Classes 1 and 2 are the positive classes, which contain chimeric nodes, while Class 3 is the negative class, containing non-chimeric nodes. Class 1 contains chimeric nodes in which the incoming node of higher coverage and the outgoing node of higher coverage come from a same species, and the incoming node of lower coverage and the outgoing node of lower coverage come from another species. Class 2 contains chimeric nodes in which the incoming node of higher coverage and the outgoing node of lower coverage come from a same species, and the incoming node of lower coverage and the outgoing node of higher coverage come from another species. Classes 1 and 2 are illustrated in Fig. 3.
  One additional task in MetaVelvet-SL is the preparation of the training sample that is required for learning the classification model. MetaVelvet-SL uses prior knowledge about the taxonomic profile (composition) of the target microbial community to generate the training sample. This taxonomic profile can be inferred from sequence reads by using taxonomic profiling methods, such as MetaPhlAn.^16^ MetaVelvet-SL has the following functions to generate the training sample. First, by using the taxonomic profile, a set of reference genomes that belong to species that are the same as or are closely related to those in the taxonomy profile are collected from the public genome database. Second, the collected reference genome sequences are used to generate simulated sequence reads, and a de Bruijn graph is constructed from the simulated read data. Third, by aligning each node in the de Bruijn graph to the reference genome sequences, it can be determined to which species genome each node belongs. Fourth, each node at a crossing point between two paths in the de Bruijn graph is labelled as Class 1, 2 or 3, generating the training samples.
3. Final assembly tasks:
  Metavelvet-SL has five major steps for this procedure.
  - Load the main de Bruijn graph which has been constructed.
  - Extract the chimeric node candidates and classify them based on the model that has been learned. After obtaining the classifications of chimeric node candidates, split the chimeric nodes that are classified as being Class 1 or 2.
  - Decompose the de Bruijn graph into connected sub-graphs.
  - Identify unique nodes. The expected coverage of each sub-graph is calculated to determine the unique nodes based on the formula used to identify a unique node in Velvet.^12^ This formula is given in the Supplementary data.
  - Perform the scaffolding procedure (Pebble and Rock Band procedures). The scaffolding procedure is iterated for each set of unique nodes from the lowest expected coverage to the highest expected coverage.

In the implementation, MetaVelvet-SL consists of two main modules: (i) the supervised learning module to develop a model for the classification of chimeric nodes and (ii) the assembly module. We provide both modules for MetaVelvet-SL's users. Users can infer the taxonomic profile from sequence reads using several well-known accurate taxonomic profiling methods, such as MetaPhlAn.^16^ Alternatively, users can generate a classification model by using prior knowledge about the taxonomy profile of the target microbial community. In either case, the resulting customized model could be well suited to the assembly of the target metagenomes. MetaVelvet-SL also provides a library of pre-trained classification models for several typical environments, such as soil, deep sea, mud, human blood, intestine and mouth.

Like other assemblers, the input for the assembly module is a set of reads from metagenomes. A pipeline connecting MetaPhlAn and MetaVelvet-SL has been developed which allows users to automatically generate a classification model and then assemble their metagenomic short read data. The source code of MetaVelvet-SL, the pipeline connecting MetaPhlAn and MetaVelvet-SL, and the library of learning models for several typical environments are freely available at http://metavelvet.dna.bio.keio.ac.jp.

## 3. Results and discussion

The results of MetaVelvet-SL were compared with those from the original MetaVelvet (version 1.2.02),^2^ the last version of other state-of-the-art metagenomic assemblers such as IDBA-UD,^9^ Ray Meta (version 2.3.1)^10^ and Omega (version 1.0.2),^11^ and the single-genome assembler for massive short sequencing reads SOAPdenovo2.^14^ We conducted extensive experiments to evaluate the performance on simulated data sets and on real metagenomic data sets of human gut microbial short read data.

### 3.1. Simulated data set

We generated simulated metagenomic sequence reads using the most frequently used simulator—the DWGSIM component in the DNAA package (available at http://sourceforge.net/projects/dnaa). We generated short reads with a length of 80 bp and used the default Illumina sequencing noise, whose error rate is 1%. The average and standard deviation of the insert size for paired-end reads were set at 500 and 50 bp, respectively.

To measure the performance for various taxonomic levels of diversity, we generated four types of assembly data sets from distant to closer taxonomic levels (order, family, genus and species). We selected 20 genomes for each data set and generated short read data sets from the 20 genomes. Since the log-normal distribution has been generally used to model microbial abundance distributions,^20^ we used the log-normal distribution for species abundance. MetaVelvet-SL requires a training data set for learning the classification model. First, we conducted experiments using the pipeline connecting MetaPhlAn and MetaVelvet-SL. The taxonomic profile for the training data set was inferred by MetaPhlAn. Second, to measure the performance of MetaVelvet-SL for different degrees of similarity between the training data set and the assembly data set, we conducted experiments using three levels of training data sets that contain different reference genomes from the assembly data sets. The three levels of training data sets consist of similarities to the assembly data set, from the highest to the lowest (genus, family and order). The genus-level training data set contains different species but in the same genus from the assembly data set. The family-level training data set contains different genus but in the same family from the assembly data set. The order-level training data set contains different families but in the same order from the assembly data set. The list of selected genomes, the coverage of each genome, the number of reads generated and the length of each reference genome for each training data set and each assembly data set are provided in Supplementary Tables S1–S20.

We compared the performance of MetaVelvet-SL with those of MetaVelvet,^2^ other state-of-the-art superior metagenomic assemblers, IDBA-UD^9^ with the default parameters for metagenomic assembly, Ray Meta^10^ with the *k*-mer size suggested in the Ray Meta paper and Omega^11^ with the overlap length suggested in the Omega's instruction, and a single-genome assembler, SOAPdenovo2^14^ with the same *k*-mer size as MetaVelvet and MetaVelvet-SL.

We evaluated the assembly performance with Nm50 and three other measurements: the total length, the maximum length and the number of accurate scaffolds (sub-scaffolds not containing any chimeric region). We defined Nm50, the corrected N50 length for metagenomic assembly results. We cut every scaffold at chimeric mis-assembled points into sub-scaffolds so that the sub-scaffolds no longer contain any chimeric region. The usual N50 is defined to indicate the scaffold length such that 50% of the total length of scaffolds lies in scaffolds of this size or larger. Nm50 is N50 length of the sub-scaffolds not containing any chimeric region. Chimeric regions in a scaffold were determined by two steps. First, the best-fit alignments between a scaffold and the set of input reference genomes are calculated using BLAST so that the predicted reference genome for the scaffold can be obtained. Second, if any region in the scaffold is aligned to another reference genome different from the predicted reference genome for the scaffold, the region is determined as a chimeric region. (The technical details are described in the Supplementary data.)

The statistics of assembly results are shown in Table 1. The taxonomic profile for the training data set used in MetaVelvet-SL was inferred by MetaPhlAn. For all assembly data sets, MetaVelvet-SL generated higher Nm50 and longer maximum length of scaffolds than MetaVelvet, IDBA-UD, Ray Meta, Omega and SOAPdenovo2. The total length of scaffolds is similar among the assemblers. The results showed that MetaVelvet-SL generated longer accurate scaffolds.

**Table 1.** Statistics of assembly results for simulated data sets

	MetaVelvet-SL (+MetaPhlAn)	MetaVelvet	IDBA-UD	SOAPdenovo2	Ray Meta	Omega
Order						
Nm50 (bp)	**695,261**	222,972	243,336	10,529	154,899	29,668
Maximum length (bp)	**3,546,677**	1,312,990	1,700,200	141,628	927,783	405,783
Total scaffold length (bp)	69,383,955	70,711,008	70,743,471	72,886,145	72,067,809	71,331,763
Number of scaffolds	4,755	2,889	1,395	37,391	1,184	17,075
Required CPU time (s)	26,735	12,606	283,378	29,980	401,873	59,275
Family						
Nm50 (bp)	**375,942**	227,243	251,915	6,751	167,523	42,500
Maximum length (bp)	**1,875,576**	1,570,565	1,247,435	167,532	1,181,121	521,402
Total scaffold length (bp)	83,890,270	76,369,071	81,721,590	86,524,823	84,549,043	84,756,027
Number of scaffolds	8,809	5,456	1,884	58,952	1,655	18,704
Required CPU time (s)	35,685	14,855	379,757	17,155	544,821	27,233
Genus						
Nm50 (bp)	**226,033**	100,132	121,196	4,642	91,637	16,533
Maximum length (bp)	**2,259,591**	2,099,603	1,246,124	85,991	1,212,747	212,138
Total scaffold length (bp)	83,281,358	84,636,187	79,218,358	81,965,701	83,171,453	73,537,116
Number of scaffolds	10,555	14,802	10,362	97,463	6,822	24,244
Required CPU time (s)	188,170	19,514	306,073	35,648	1,259,371	97,573
Species						
Nm50 (bp)	**174,495**	91,159	74,670	4,469	80,592	13,053
Maximum length (bp)	**3,808,921**	1,878,401	2,107,202	103,314	702,714	193,065
Total scaffold length (bp)	81,524,460	82,381,332	65,980,631	85,892,445	74,075,828	67,422,938
Number of scaffolds	22,440	29,472	18,864	132,284	17,077	24,096
Required CPU time (s)	195,454	17,610	353,353	20,082	352,521	208,417

All computations were executed using Intel(R) Xeon(R) E5540 processors (2.53 GHz), with 96-GB physical memory, except for a few cases. Top performances are shown in bold.

For all assembly data sets, Ray Meta required the largest computation times, followed by IDBA-UD. The computation times of MetaVelvet-SL increased at low taxonomic levels compared with MetaVelvet. This is mainly because MetaVelvet-SL requires the computation time for learning the classification model and classifying chimeric node candidates the number of which is larger in low taxonomic levels. The genomes become more similar and share more *k*-mers in low taxonomic levels. Table 2 represents the number of chimeric node candidates in de Bruijn graph constructed from each assembly data set. The species data set has the highest number of chimeric node candidates which is >10 times of the number of chimeric node candidates in the order data set.

**Table 2.** The number of chimeric node candidates in de Bruijn graph constructed from each assembly data set

	Positive		Negative	Total no. of chimeric node candidates
	Class 1	Class 2	Class 3	
Order	82	0	1,515	1,597
Family	146	1	2,456	2,603
Genus	2,918	731	8,505	12,154
Species	3,074	246	14,589	17,909

The statistics of assembly of MetaVelvet-SL using different training datasets are shown in Table 3. The models used in MetaVelvet-SL were generated from the taxonomic profiles predicted by MetaPhlAn and three similarity levels of training data sets (genus, family and order) for each assembly data set. There was no significant difference among the assembly results using different training data sets. This result showed that MetaVelvet-SL was robust for the dissimilarity between the training data set and the assembly data set.

**Table 3.** Statistics of assembly results of MetaVelvet-SL using different training data sets

	MetaVelvet-SL			
	(+MetaPhlAn)	Genus-level training data set	Family-level training data set	Order-level training data set
Order				
Nm50 (bp)	695,261	672,952	686,074	695,557
Maximum length (bp)	3,546,677	3,415,875	3,547,025	3,818,061
Total scaffold length (bp)	69,383,955	69,881,185	69,288,924	69,387,743
Number of scaffolds	4,755	4,379	4,747	4,829
Required CPU time (s)	26,735	26,773	26,612	26,660
Family				
Nm50 (bp)	375,942	377,604	384,795	384,795
Maximum length (bp)	1,875,576	2,326,125	2,326,197	1,927,551
Total scaffold length (bp)	83,890,270	83,888,560	83,877,454	83,877,321
Number of scaffolds	8,809	8,777	8,687	8,679
Required CPU time (s)	35,685	35,477	35,577	35,469
Genus				
Nm50 (bp)	226,033	233,924	266,018	257,292
Maximum length (bp)	2,259,591	2,888,749	2,974,950	2,843,963
Total scaffold length (bp)	83,281,358	82,525,756	83,385,233	83,634,261
Number of scaffolds	10,555	11,450	9,376	8,512
Required CPU time (s)	188,170	187,963	188,285	188,325
Species				
Nm50 (bp)	174,495	166,528	158,509	167,722
Maximum length (bp)	3,808,921	3,292,179	3,292,250	3,292,179
Total scaffold length (bp)	81,524,460	81,141,481	81,447,218	81,414,639
Number of scaffolds	22,440	19,114	21,073	22,735
Required CPU time (s)	195,454	195,512	195,534	195,460

Since one of our primary goals in this work is to improve the sensitivity and accuracy for detecting chimeric nodes by supervised learning, we compared the classification capability of chimeric nodes by MetaVelvet-SL and MetaVelvet. The results of the classification are shown in Table 4. We evaluated the sensitivity and the accuracy. Sensitivity is the true positive rate, the percentage of true identified chimeric nodes (positive classes consists of Class 1 and Class 2), while accuracy is the percentage of true results, both true positive (true identified chimeric nodes) and true negative (true identified non-chimeric nodes, consisting of Class 3). As shown in Table 2, all of the assembly data sets have imbalanced classes of chimeric node candidates. Therefore, to avoid inflated performance estimates on imbalanced data sets, we calculated the balanced accuracy too. Balanced accuracy is the average between sensitivity and specificity.^21^ Specificity is the true negative rate, the percentage of true identified non-chimeric nodes. As shown in Table 4, the balanced accuracy of MetaVelvet-SL, even for the lowest similarity level of training data set (order-level training data set), was higher than MetaVelvet.

**Table 4.** Classification results for chimeric nodes

	MetaVelvet-SL												MetaVelvet		
	(Training: Genus)			(Training: Family)			(Training: Order)			(+MetaPhlAn)					
	Sen	Acc	BA	Sen	Acc	BA	Sen	Acc	BA	Sen	Acc	BA	Sen	Acc	BA
Order	84.15	94.49	89.60	54.88	96.12	76.61	57.32	95.49	77.44	42.68	96.62	71.11	42.68	92.42	67.74
Family	80.27	97.54	89.42	63.27	97.08	81.80	55.10	96.85	77.23	65.99	97.35	82.61	44.90	93.32	69.21
Genus	62.76	76.92	72.88	50.07	65.03	60.76	54.29	67.88	64.00	58.98	81.90	75.35	33.13	62.37	46.91
Species	52.80	54.84	54.05	36.83	70.60	57.32	33.10	82.19	63.23	40.24	83.15	66.58	15.21	71.61	48.09

Sen (%) means the percentage of sensitivity; Acc (%) means the percentage of accuracy and BA (%) means the percentage of balanced accuracy.

### 3.2. Real data set

To evaluate the performance of MetaVelvet-SL on real metagenomic data, we used human gut microbial data sets. We assembled five human gut microbial data sets: MH0006 (ERS006497), MH0012 (ERS006494) and MH0047 (ERS006592) from the MetaHIT Consortium^17^ and SRS017227 and SRS018661 from the Human Microbiome Project Consortium.^18^ Two of the data sets (MH0006 and MH0012) were the deepest and second deepest data sets while another data set, MH0047, is a low-coverage data set.

As for the simulated data sets, the real data sets were assembled by MetaVelvet-SL, MetaVelvet,^2^ IDBA-UD^9^ with the default parameters for metagenomic assembly, Ray Meta^10^ with the *k*-mer size suggested in the Ray Meta paper, Omega^11^ with the overlap length suggested in the Omega's instruction and SOAPdenovo2.^14^ The statistics of assembly performances are summarized in Table 5. The classification model for MetaVelvet-SL was obtained by the pipeline using MetaPhlAn to infer the taxonomic profile and then generating the training data set.

**Table 5.** Assembly results for the real human gut microbial data sets

	MetaVelvet-SL (+ MetaPhlAn)	MetaVelvet	IDBA-UD	SOAPdenovo2	Ray Meta	Omega
MH0006 (ERS006497)						
Maximum length (bp)	**1,073,577**	82,400	424,786	248,752	245,285	293,858
Total scaffold length (bp)	**366,474,614**	228,356,028	293,629,444	314,842,356	211,199,449	134,644,249
Number of scaffold	927,151	387,193	197,401	521,577	609,062	150,907
AUC of N-len(x)	**9,148,384**	909,250	6,002,739	3,042,215	2,260,838	2,527,198
MH0012 (ERS006494)						
Maximum length (bp)	**1,320,619**	119,936	594,225	792,429	512,973	1,144,479
Total scaffold length (bp)	**357,949,718**	255,566,175	290,340,811	325,057,612	272,663,103	170,102,775
Number of scaffold	718,438	327,103	198,771	482,983	635,814	125,383
AUC of N-len(x)	**22,126,171**	2,129,027	10,344,620	8,856,698	6,977,480	10,229,304
MH0047 (ERS006592)						
Maximum length (bp)	**188,905**	69,475	185,593	44,319	137,473	52,084
Total scaffold length (bp)	**101,916,143**	75,290,864	75,032,143	88,092,865	50,174,724	29,134,928
Number of scaffold	374,148	210,477	89,786	263,713	141,466	31,961
AUC of N-len(x)	**906,906**	237,568	802,594	201,366	544,742	208,223
SRS017227						
Maximum length (bp)	**478,428**	108,476	372,927	227,256	199,208	217,259
Total scaffold length (bp)	370,496,571	250,969,598	349,934,212	**395,257,497**	273,595,801	206,705,202
Number of scaffold	602,463	485,307	282,097	802,952	536,708	217,259
AUC of N-len(x)	**4,709,551**	1,064,102	4,010,530	2,227,194	2,501,039	1,617,896
SRS018661						
Maximum length (bp)	**699,395**	111,404	511,735	426,297	274,042	180,946
Total scaffold length (bp)	**114,676,867**	71,339,406	109,507,232	107,557,997	75,351,327	47,619,933
Number of scaffold	284,036	195,950	109,860	274,896	212,267	34,244
AUC of N-len(x)	**1,945,258**	253,138	1,296,606	848,798	1,004,371	611,636

Top performances are shown in bold. MetaVelvet-SL, MetaVelvet and SOAPdenovo2 set the *k*-mer size at 37 for the MH0006 and MH0047 data sets, 43 for the MH0012 data set and 51 for SRS017227 and SRS018661.

**Figure 4.**
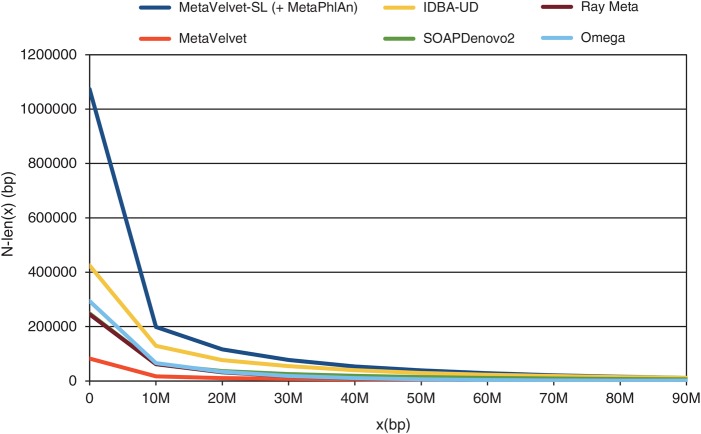
The N-len(x) plots for the MH0006 data set of human gut microbial data.

When the total scaffold lengths of two assemblies are quite different in the human gut microbial data sets, the naive use of N50 score is inadequate, because the longer total length decreases the N50 score. The generalized score N-len(*x*) is more appropriate for comparing scaffold integrity than the raw N50 score.^2^ N-len(*x*) is defined by

(1) $$\text{N-len(}x)=|S_{i}|\;\mathrm{such}\;\mathrm{that}\;\sum_{\;j=1}^{i}|S_{j}|\geq x\;\text{and}\;\sum_{\;j=1}^{i-1}|S_{j}|<x,$$

where *S_1_, S_2_, … , S_n_* denote the list of scaffolds in descending order of length as output by an assembler. The N50 score corresponds to the N-len(*x*) score for *x* = *L*/2 (*x* is 50% of *L*), where *L* denotes the total scaffold length. The N-len(*x*) plots for the MH0006 data sets produced by MetaVelvet-SL, MetaVelvet, IDBA-UD, SOAPdenovo2, Ray Meta and Omega are shown in Fig. 4. MetaVelvet-SL significantly increased the scaffold integrity. For example, when *x* = 5,000,000, the N-len(*x*) score of MetaVelvet-SL was 306,496, the N-len(*x*) score of MetaVelvet was 24,554, the N-len(*x*) score of IDBA-UD was 178,659, the N-len(*x*) score of SOAPdenovo2 was 90,861, the N-len(*x*) score of Ray Meta was 101,726 and the N-len(*x*) score of Omega was 117,010. (The N-len(*x*) plots for the MH0012, MH0047, SRS017227, and SRS018661 data sets are shown in Supplementary Figs S1–S4.) As in the MetaVelvet paper, we calculated the area under the curve (AUC) of N-len(*x*) for 0 *< x ≤ L* in units of 1,000,000 bp; that is, the cumulative sum of N-len(*x*) scores (0 *< x ≤ L*), where *L* denotes the total scaffold length.

MetaVelvet-SL generated much longer accurate scaffolds than MetaVelvet, IDBA-UD, Ray Meta, Omega and SOAPdenovo2, showing that MetaVelvet-SL improved scaffold integrity. MetaVelvet-SL outperformed MetaVelvet, IDBA-UD, Ray Meta, Omega and SOAPdenovo2 for all data sets in terms of all three of the performance indicators (total length of scaffolds, maximum length of scaffolds and AUC), except for the SRS017227 data sets, SOAPdenovo2 generated slightly longer total length of scaffolds. The identification of chimeric nodes by MetaVelvet-SL using classification models generated from the taxonomic profile inferred by MetaPhlAn is shown in Supplementary Table S21.

Comparisons between the taxonomic profile predicted by MetaPhlAn and the taxonomic profile based on assembly results of MetaVelvet-SL using BLAST were accomplished. The NCBI genomic reference sequences were used, which provide stable references, as the database for BLAST searching. The database contains 41,913 organisms as of September 2014 (Release 67). The numbers of species predicted by MetaPhlAn and predicted from the assembly of MetaVelvet-SL are shown in Table 6. The taxonomic profile based on assembly results covered >90% of the taxonomic profile predicted by MetaPhlAn. This result indicated that the assembly capacity of MetaVelvet-SL was high enough to capture the target diverse microbial community. As shown in Table 6, much larger number of species was predicted from assembly results by BLAST than predicted by MetaPhlAn. This is mainly because MetaPhlAn used 2,887 genomes available from the Integrated Microbial Genomes (IMG) system, which were much fewer than the number of organisms in the NCBI database used in BLAST searching. The taxonomic profile predicted by MetaPhlAn for each real data set is shown in Supplementary Tables S22–S26, whereas the taxonomy profile predicted from the assembly of MetaVelvet-SL using BLAST is shown in Supplementary Tables S27–S31.

**Table 6.** The number of species in the taxonomic profile predicted by MetaPhlAn and the taxonomic profile based on assembly results of MetaVelvet-SL using BLAST

	Number of species predicted by both	Number of species predicted only by MetaPhlAn	Number of species predicted only by assembly
MH0006 (ERS006497)	99	5	2,932
MH0012 (ERS006494)	124	9	2,872
MH0047 (ERS006592)	65	2	2,137
SRS017227	83	3	2,992
SRS018661	81	8	1,529

The first column represents the number of species predicted by MetaPhlAn and predicted from assembly results by BLAST (intersection). The second column represents the number of species only predicted by MetaPhlAn and not predicted from assembly results by BLAST. The third column represents the number of species only predicted from assembly results by BLAST and not predicted by MetaPhlAn.

### 3.3. Conclusion

Extensive experiments on simulated and real metagenomic data sets showed that MetaVelvet-SL outperformed other metagenomic assemblers MetaVelvet, IDBA-UD, Ray Meta and Omega as well as a single-genome assembler, SOAPdenovo2.

The main strategy in MetaVelvet-SL is to develop a model to classify a candidate node at a crossing point between two incoming and two outgoing edges as a chimeric node or not. We also developed a procedure to identify unique nodes more precisely based on the expected coverage for each sub-graph and considered very low-coverage nodes by determining an appropriate threshold to remove error nodes. Since MetaVelvet-SL needs to learn a model for the classification of chimeric nodes, we have provided a pipeline connecting MetaPhlAn and MetaVelvet-SL, which can generate a classification model and assemble automatically. MetaVelvet-SL also provides a library of pre-trained classification models for several typical environments such as soil, deep sea, mud, human blood, intestine and mouth.

MetaVelvet-SL defines a chimeric node as a node that has two incoming edges and two outgoing edges. In a de Bruijn graph, in single-genome assembly, a node with multiple incoming and outgoing edges represents a repeat node. In multiple genome assembly, such node is not necessarily a repeat since it is sometimes shared between the genomes of two closely related species and represents orthologous sequences, conserved sequences (such as rRNA sequences) or horizontal transfer sequences. In a de Bruijn graph, nodes having multiple incoming and outgoing edges can be divided into (i) those with two incoming edges and two outgoing edges and (ii) those with higher order connectivity (i.e. more than two incoming edges and more than two outgoing edges). The number of nodes having higher order connectivity is much fewer than the number of nodes having two incoming edges and two outgoing edges. We counted the number of nodes having multiple incoming and outgoing edges in de Bruijn graphs for both simulated data sets and real data sets of human gut microbial short read data. On average, the number of nodes having higher order connectivity is only 1.79% of the number of nodes having two incoming edges and two outgoing edges. The number of nodes for each data set is provided in Supplementary Table S32. Although MetaVelvet-SL defines a candidate for a chimeric node as a node that has two incoming edges and two outgoing edges, MetaVelvet-SL outperformed the other metagenomic assemblers MetaVelvet, IDBA-UD, Ray Meta and Omega, and also a single genome assembler, SOAPdenovo2. We continue to consider the impact of higher order connectivity.

## Supplementary data

Supplementary data are available at www.dnaresearch.oxfordjournals.org.

## Funding

This work was supported by a Grant-in-Aid for Scientific Research in Innovative Areas No. 221S0002 and Scientific Research (A) No. 23241066 from the Ministry of Education, Culture, Sports, Science and Technology of Japan. Funding to pay the Open Access publication charges for this article was provided by Grant-in-Aid for Scientific Research in Innovative Areas No. 221S0002 from the Ministry of Education, Culture, Sports, Science and Technology of Japan.

## Supplementary Material

Supplementary Data
