# Supplementary material for: MetaVelvet-SL: an extension of the Velvet assembler to a de novo metagenomic assembler utilizing supervised learning
Source: DNA Res. 2014 Nov 27;22(1):69–77. doi: 10.1093/dnares/dsu041 (PMC4379979; doi:10.1093/dnares/dsu041)
Supplement: Supplementary Data [file supp_22_1_69__index.html]

MetaVelvet-SL: an extension of the Velvet assembler to a de novo metagenomic assembler utilizing supervised learning — MetaVelvet-SL: an extension of the Velvet assembler to a de novo metagenomic assembler utilizing supervised learning — Supplementary Data 

# MetaVelvet-SL: an extension of the Velvet assembler to a *de novo* metagenomic assembler utilizing supervised learning

## Supplementary Data

Supplementary Data

**Files in this Data Supplement:**

- Supplementary Data - Pdf file
