# Supplementary material for: MetaVelvet-SL: an extension of the Velvet assembler to a de novo metagenomic assembler utilizing supervised learning
Source: DNA Res. 2014 Nov 27;22(1):69–77. doi: 10.1093/dnares/dsu041 (PMC4379979; doi:10.1093/dnares/dsu041)
Supplement: Supplementary Data [file supp_dsu041_dsu041supp.pdf]

Supplementary Figures and Tables

MetaVelvet-SL: An extension of the Velvet assembler to a *de novo* metagenomic assembler utilizing supervised learning

Afiahayati [1] , Kengo Sato [1] and Yasubumi Sakakibara [1*]
Department of Biosciences and Informatics, Keio University, 3-14-1 Hiyoshi, Kohoku-ku, Yokohama 223-8522, Japan

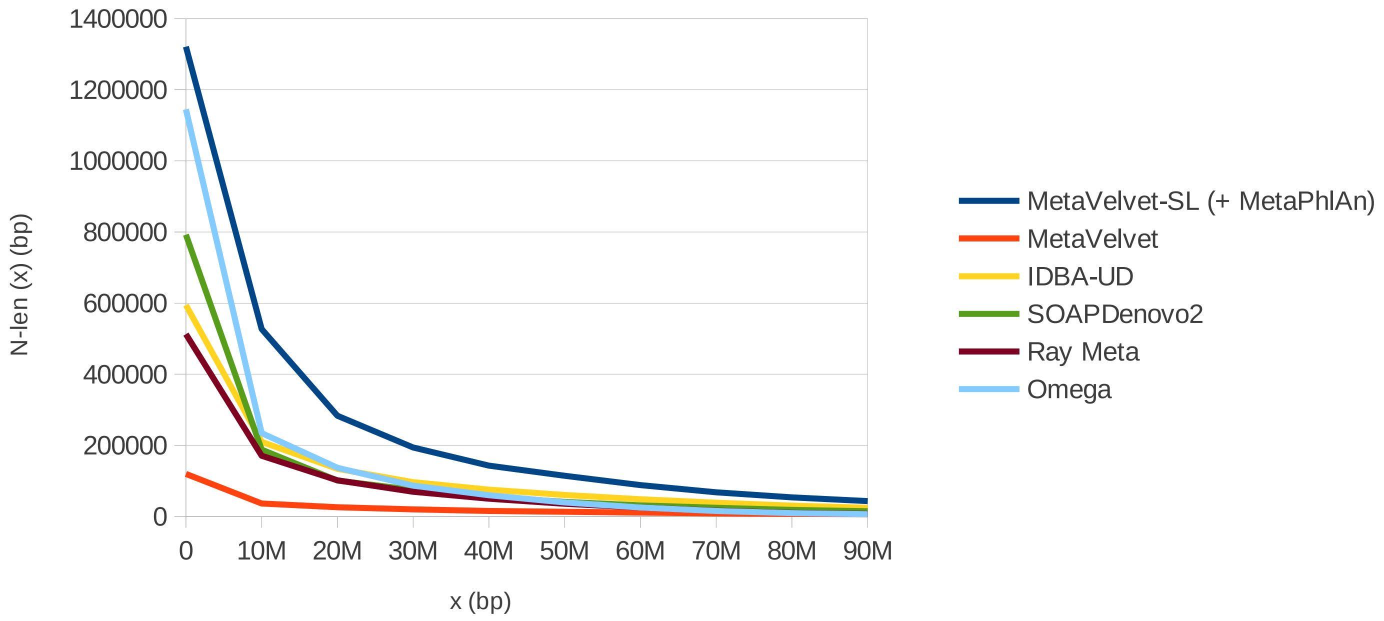

Figure S1: The N-len(x) plots for the MH0012 dataset of human gut microbial data.

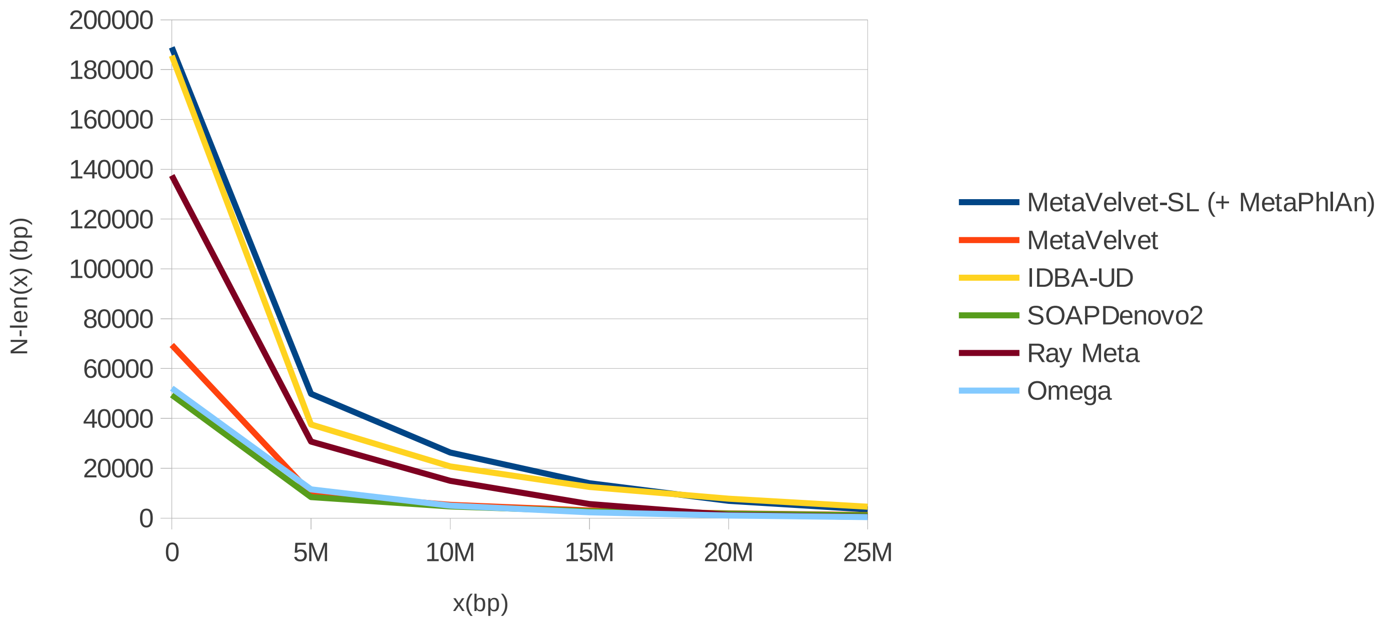

Figure S2: The N-len(x) plots for the MH0047 dataset of human gut microbial data.

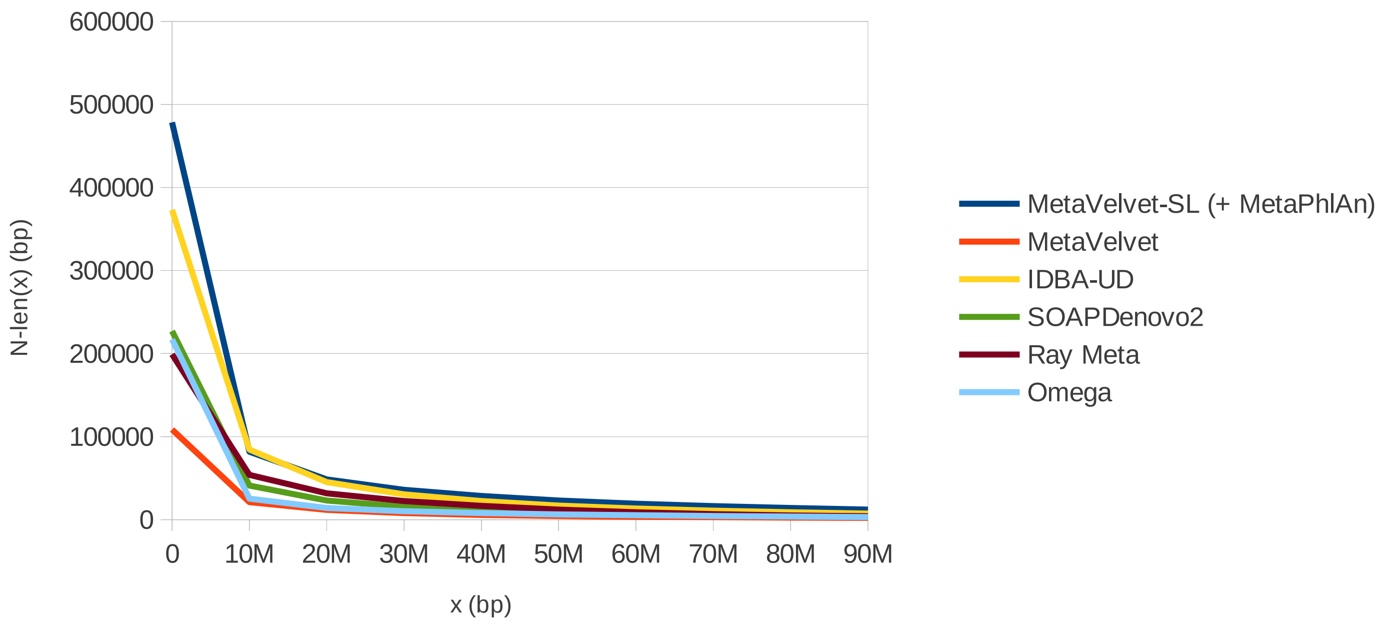

Figure S3: The N-len(x) plots for the SRS017227 dataset of human gut microbial data.

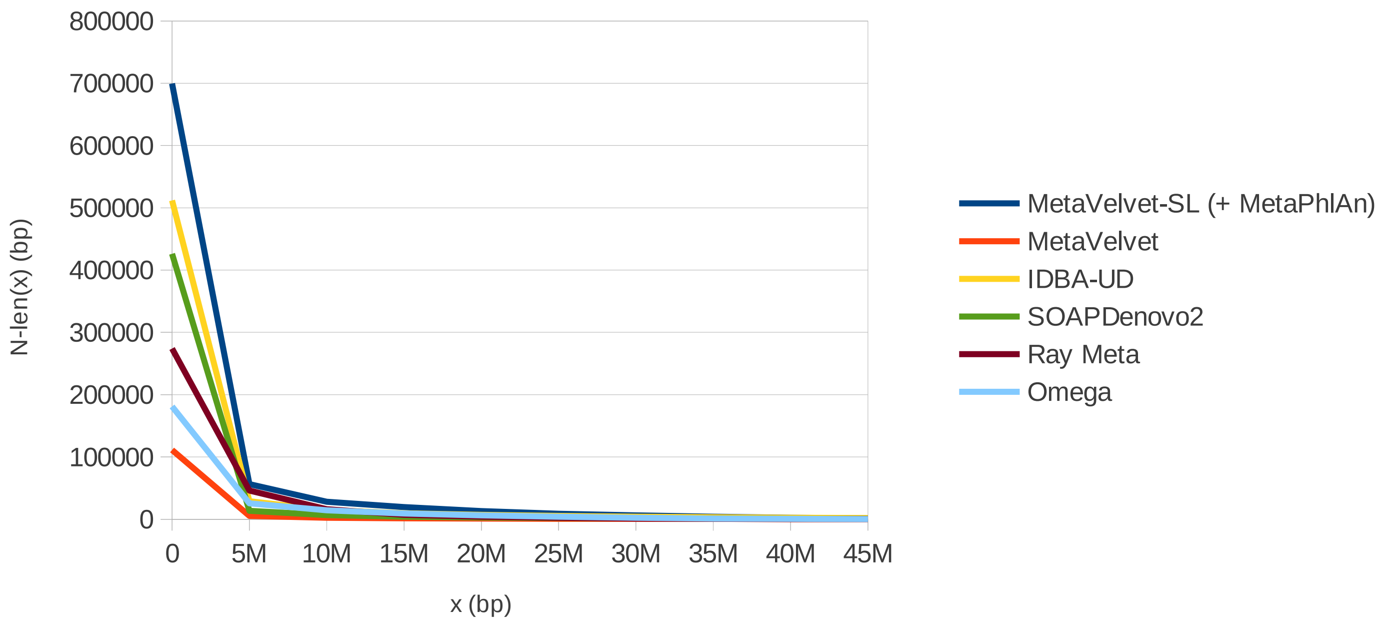

Figure S4: The N-len(x) plots for the SRS018661 dataset of human gut microbial data.

Formula to identify unique nodes in Velvet (Zerbino and Birney, 2008)

$$F(\bar{x},n,\rho)=\frac{\log 2}{2}+n\frac{\rho^2-\frac{\bar{x}^2}{2}}{2}$$

where

$\bar{x}$ = the coverage of node

ρ = expected coverage of subgraph

n = the length of node

A node is “unique”, if its $F > 5$.

Procedure to determine the chimeric regions in a scaffold.

Nm50, the corrected N50 length for metagenomic assembly results, is the N50 length of the sub-scaffolds not containing any chimeric region. We cut every scaffold at chimeric mis-assembled points into sub-scaffolds so that the sub-scaffolds no longer contain any chimeric region. Chimeric regions in a scaffold were determined by two steps :

1. The best fit alignments between a scaffold and the set of input reference genomes are calculated using BLAST so that the predicted reference genome for the scaffold can be obtained. The predicted reference genome of the scaffold is the subject sequence of the alignment hit with the highest bit score. We only consider alignment hits with identity higher than 95%.

2. If any region in the scaffold is aligned to another reference genome different from the predicted reference genome for the scaffold, the region is determined as a chimeric region.

The illustration is shown in the Figure S5. A scaffold is aligned to the reference genomes. The predicted reference genome of the scaffold is the reference genome of the alignment hit with the highest bit score (colour: brown). The chimeric region is any region aligned to different reference genome with the predicted reference genome of the scaffold. In the illustration, there are two chimeric regions: chimeric region 1 and chimeric region 2. The scaffold is cut in each chimeric region into sub-scaffolds no longer containing any chimeric region. In the illustration, there are three sub-scaffolds generated: sub-scaffold a, sub-scaffold b and sub-scaffold c .

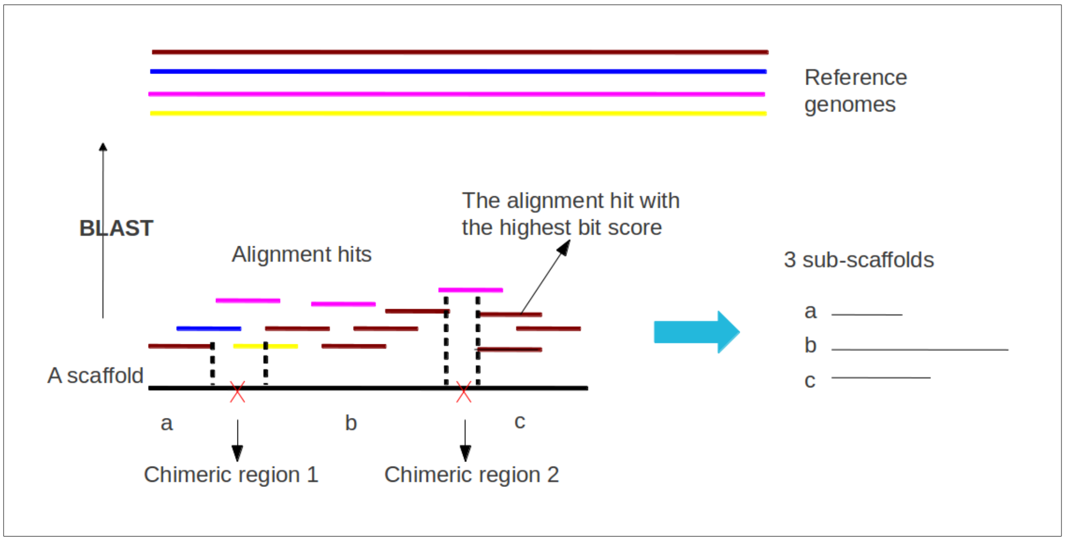

Figure S5: Illustration of the procedure to determine the chimeric regions.

Table S1 : Taxonomic profile of the order target assembly dataset.

Domain	Phylum	Class	Order	Family	Genus	Species	Strain	Length	Coverage	# read pairs
Bacteria	Proteobacteria	Alphaproteobacteria	**Caulobacterales**	Caulobacteraceae	Caulobacter	Caulobacter crescentus	NA1000	4042929	90.68	4823852
Bacteria	Proteobacteria	Alphaproteobacteria	**Rhizobiales**	Rhizobiaceae	Rhizobium	Rhizobium etli	CFN 42	4381608	30.00	1729582
Bacteria	Proteobacteria	Alphaproteobacteria	**Rhodobacterales**	Rhodobacteraceae	Dinoroseobacter	Dinoroseobacter shibae	DFL 12	3789584	224,47	11192736
Bacteria	Proteobacteria	Alphaproteobacteria	**Parvularculales**	Parvularculaceae	Parvularcula	Parvularcula bermudensis	HTCC2503	2902643	57.69	2203335
Bacteria	Proteobacteria	Alphaproteobacteria	**Rhodospirillales**	Rhodospirillaceae	Azospirillum	Azospirillum sp.	B510	3311395	48.6	2128878
Bacteria	Proteobacteria	Alphaproteobacteria	**Sphingomonadales**	Erythrobacteraceae	Erythrobacter	Erythrobacter litoralis	HTCC2594	3052398	30.00	1204894
Bacteria	Proteobacteria	Gammaproteobacteria	**Acidithiobacillales**	Acidithiobacillaceae	Acidithiobacillus	Acidithiobacillus ferrooxidans	ATCC 23270	2982397	356.34	13983518
Bacteria	Proteobacteria	Gammaproteobacteria	**Aeromonadales**	Aeromonadaceae	Aeromonas	Aeromonas hydrophila subsp. hydrophila	ATCC 7966	4744448	30.00	1872808
Bacteria	Proteobacteria	Gammaproteobacteria	**Alteromonadales**	Alteromonadaceae	Alteromonas	Alteromonas macleodii	Deep ecotype	4412282	54,35	3181606
Bacteria	Proteobacteria	Gammaproteobacteria	**Cardiobacteriales**	Cardiobacteriaceae	Dichelobacter	Dichelobacter nodosus	VCS1703A	1389350	45.97	840374
Bacteria	Proteobacteria	Gammaproteobacteria	**Chromatiales**	Ectothiorhodospiraceae	Alkalilimnicola	Alkalilimnicola ehrlichei	MLHE-1	3275944	30.00	1293136
Bacteria	Proteobacteria	Gammaproteobacteria	**Enterobacteriales**	Enterobacteriaceae	Escherichia	Escherichia coli	str. K-12 substr. MG1655	4639675	144.19	8802562
Bacteria	Proteobacteria	Gammaproteobacteria	**Legionellales**	Legionellaceae	Legionella	Legionella pneumophila	Paris	3503610	30.00	1383004
Bacteria	Proteobacteria	Gammaproteobacteria	**Methylococcales**	Methylococcaceae	Methylococcus	Methylococcus capsulatus	Bath	3304561	134.78	5860378
Bacteria	Proteobacteria	Gammaproteobacteria	**Oceanospirillales**	Halomonadaceae	Chromohalobacter	Chromohalobacter salexigens	DSM 3043	3696649	30.00	1459204
Bacteria	Proteobacteria	Gammaproteobacteria	**Pasteurellales**	Pasteurellaceae	Pasteurella	Pasteurella multocida subsp. multocida	Pm70	2257487	100.76	2992952
Bacteria	Proteobacteria	Gammaproteobacteria	**Pseudomonadales**	Pseudomonadaceae	Pseudomonas	Pseudomonas putida	W619	5774330	87.97	6683787
Bacteria	Proteobacteria	Gammaproteobacteria	**Thiotrichales**	Piscirickettsiaceae	Thiomicrospira	Thiomicrospira crunogena	XCL-2	2427734	99.93	3192151
Bacteria	Proteobacteria	Gammaproteobacteria	**Vibrionales**	Vibrionaceae	Vibrio	Vibrio cholerae O1 biovar eltor	N16961	2961149	174.74	6808305
Bacteria	Proteobacteria	Gammaproteobacteria	**Xanthomonadales**	Xanthomonadaceae	Xanthomonas	Xanthomonas campestris pv. campestris	B100	5079002	275.25	18394675

Table S2 : Taxonomic profile of the family target assembly dataset.

Domain	Phylum	Class	Order	Family	Genus	Species	Strain	Length	Coverage	# read pairs
Bacteria	Proteobacteria	Alphaproteobacteria	Rhizobiales	**Bartonellaceae**	Bartonella	Bartonella bacilliformis	KC583	1445021	124.84	2373638
Bacteria	Proteobacteria	Alphaproteobacteria	Rhizobiales	**Beijerinckiaceae**	Methylocella	Methylocella silvestris	BL2	4305430	413.22	23409076
Bacteria	Proteobacteria	Alphaproteobacteria	Rhizobiales	**Bradyrhizobiaceae**	Nitrobacter	Nitrobacter hamburgensis	X14	4406967	30.00	1739592
Bacteria	Proteobacteria	Alphaproteobacteria	Rhizobiales	**Brucellaceae**	Brucella	Brucella suis	1330	2107794	30.00	832024
Bacteria	Proteobacteria	Alphaproteobacteria	Rhizobiales	**Hyphomicrobiaceae**	Hyphomicrobium	Hyphomicrobium denitrificans	ATCC 51888	3638969	30.16	1444096
Bacteria	Proteobacteria	Alphaproteobacteria	Rhizobiales	**Methylobacteriaceae**	Methylobacterium	Methylobacterium chloromethanicum	CM4	5777908	120.26	9143388
Bacteria	Proteobacteria	Alphaproteobacteria	Rhizobiales	**Phyllobacteriaceae**	Mesorhizobium	Mesorhizobium loti	MAFF303099	7036071	147.34	13640720
Bacteria	Proteobacteria	Alphaproteobacteria	Rhizobiales	**Rhizobiaceae**	Rhizobium	Rhizobium etli	CFN 42	4381608	35.00	2017846
Bacteria	Proteobacteria	Alphaproteobacteria	Rhizobiales	**Xanthobacteraceae**	Azorhizobium	Azorhizobium caulinodans	ORS 571	5369772	56.20	3971370
Bacteria	Proteobacteria	Gammaproteobacteria	Alteromonadales	**Alteromonadaceae**	Alteromonas	Alteromonas macleodii	Deep ecotype	4412282	30.00	1756176
Bacteria	Proteobacteria	Gammaproteobacteria	Alteromonadales	**Colwelliaceae**	Colwellia	Colwellia psychrerythraea	34H	5373180	30.00	2120992
Bacteria	Proteobacteria	Gammaproteobacteria	Alteromonadales	**Ferrimonadaceae**	Ferrimonas	Ferrimonas balearica	DSM 9799	4279159	484.74	27293152
Bacteria	Proteobacteria	Gammaproteobacteria	Alteromonadales	**Idiomarinaceae**	Idiomarina	Idiomarina loihiensis	L2TR	2839318	58.56	2187770
Bacteria	Proteobacteria	Gammaproteobacteria	Alteromonadales	**Pseudoalteromonadaceae**	Pseudoalteromonas	Pseudoalteromonas haloplanktis	TAC125	3214944	40.58	1716610
Bacteria	Proteobacteria	Gammaproteobacteria	Alteromonadales	**Psychromonadaceae**	Psychromonas	Psychromonas ingrahamii	37	4559598	141.38	8482052
Bacteria	Proteobacteria	Gammaproteobacteria	Alteromonadales	**Shewanellaceae**	Shewanella	Shewanella oneidensis	MR-1	4969803	49.15	3214030
Bacteria	Firmicutes	Bacilli	Bacillales	**Bacillaceae**	Bacillus	Bacillus subtilis subsp. subtilis	168	4214630	101.03	5603982
Bacteria	Firmicutes	Bacilli	Bacillales	**Listeriaceae**	Listeria	Listeria monocytogenes serotype 4b	F2365	2905187	38.08	1455652
Bacteria	Firmicutes	Bacilli	Bacillales	**Paenibacillaceae**	Brevibacillus	Brevibacillus brevis	NBRC 100599	6296436	55.90	4631194
Bacteria	Firmicutes	Bacilli	Bacillales	**Alicyclobacillaceae**	Alicyclobacillus	Alicyclobacillus acidocaldarius subsp. acidocaldarius	DSM 446	3018755	30.10	1195586

Table S3 : Taxonomic profile of the genus target assembly dataset.

Domain	Phylum	Class	Order	Family	Genus	Species	Strain	Length	Coverage	# read pairs
Bacteria	Proteobacteria	Gammaproteobacteria	Enterobacteriales	Enterobacteriaceae	**Yersinia**	Yersinia pestis	Angola	4504254	390.28	23130530
Bacteria	Proteobacteria	Gammaproteobacteria	Enterobacteriales	Enterobacteriaceae	**Candidatus Moranella**	Candidatus Moranella endobia	PCIT	53294	243.89	1727428
Bacteria	Proteobacteria	Gammaproteobacteria	Enterobacteriales	Enterobacteriaceae	**Sodalis**	Sodalis glossinidius	morsitans	4171146	92.53	5078370
Bacteria	Proteobacteria	Gammaproteobacteria	Enterobacteriales	Enterobacteriaceae	**Citrobacter**	Citrobacter koseri	ATCC BAA-895	4720462	30 .00	1863340
Bacteria	Proteobacteria	Gammaproteobacteria	Enterobacteriales	Enterobacteriaceae	**Cronobacter**	Cronobacter sakazakii	ATCC BAA-894	4368373	30 .00	1724358
Bacteria	Proteobacteria	Gammaproteobacteria	Enterobacteriales	Enterobacteriaceae	**Dickeya**	Dickeya dadantii	Ech703	4679450	166.48	10250458
Bacteria	Proteobacteria	Gammaproteobacteria	Enterobacteriales	Enterobacteriaceae	**Edwardsiella**	Edwardsiella ictaluri	93-146	3812315	81.42	4084178
Bacteria	Proteobacteria	Gammaproteobacteria	Enterobacteriales	Enterobacteriaceae	**Enterobacter**	Enterobacter asburiae	LF7a	4812833	72.73	4605754
Bacteria	Proteobacteria	Gammaproteobacteria	Enterobacteriales	Enterobacteriaceae	**Erwinia**	Erwinia billingiae	Eb661	5100167	30 .00	2013224
Bacteria	Proteobacteria	Gammaproteobacteria	Enterobacteriales	Enterobacteriaceae	**Escherichia**	Escherichia coli	str. K-12 substr. MG1655	4639675	81.31	4963842
Bacteria	Proteobacteria	Gammaproteobacteria	Enterobacteriales	Enterobacteriaceae	**Klebsiella**	Klebsiella variicola	At-22	5458505	30 .00	2154673
Bacteria	Proteobacteria	Gammaproteobacteria	Enterobacteriales	Enterobacteriaceae	**Pantoea**	Pantoea vagans	C9-1	4024986	241.81	12806340
Bacteria	Proteobacteria	Gammaproteobacteria	Enterobacteriales	Enterobacteriaceae	**Pectobacterium**	Pectobacterium atrosepticum	SCRI 1043	5064019	30 .00	1998955
Bacteria	Proteobacteria	Gammaproteobacteria	Enterobacteriales	Enterobacteriaceae	**Photorhabdus**	Photorhabdus asymbiotica subsp. asymbiotica	ATCC 43949	5064808	63.75	4248441
Bacteria	Proteobacteria	Gammaproteobacteria	Enterobacteriales	Enterobacteriaceae	**Proteus**	Proteus mirabilis	HI4320	4063606	83.65	4472640
Bacteria	Proteobacteria	Gammaproteobacteria	Enterobacteriales	Enterobacteriaceae	**Rahnella**	Rahnella sp.	Y9602	4864217	57.65	3689765
Bacteria	Proteobacteria	Gammaproteobacteria	Enterobacteriales	Enterobacteriaceae	**Salmonella**	Salmonella bongori	NCTC 12419	4460105	30 .00	1760568
Bacteria	Proteobacteria	Gammaproteobacteria	Enterobacteriales	Enterobacteriaceae	**Serratia**	Serratia proteamaculans	568	5448853	96.20	6897101
Bacteria	Proteobacteria	Gammaproteobacteria	Enterobacteriales	Enterobacteriaceae	**Shigella**	Shigella flexneri5	8401	4574284	80.21	4827675
Bacteria	Proteobacteria	Gammaproteobacteria	Enterobacteriales	Enterobacteriaceae	**Xenorhabdus**	Xenorhabdus bovienii	SS-2004	4225498	182.67	10156207

Table S4 : Taxonomic profile of the species target assembly dataset.

Domain	Phylum	Class	Order	Family	Genus	Species	Strain	Length	Coverage	# read pairs
Bacteria	Firmicutes	Bacilli	Bacillales	Bacillaceae	Bacillus	**Bacillus amyloliquefaciens**	DSM 7	3980199	92.04	4820230
Bacteria	Firmicutes	Bacilli	Bacillales	Bacillaceae	Bacillus	**Bacillus anthracis**	Ames	5227293	270.68	18617416
Bacteria	Firmicutes	Bacilli	Bacillales	Bacillaceae	Bacillus	**Bacillus atrophaeus**	1942	4168266	30.04	1647562
Bacteria	Firmicutes	Bacilli	Bacillales	Bacillaceae	Bacillus	**Bacillus cellulosilyticus**	DSM 2522	4681672	100.35	6181656
Bacteria	Firmicutes	Bacilli	Bacillales	Bacillaceae	Bacillus	**Bacillus cereus**	Q1	5214195	31.46	2158402
Bacteria	Firmicutes	Bacilli	Bacillales	Bacillaceae	Bacillus	**Bacillus clausii**	KSM K16	4303871	114.47	6482422
Bacteria	Firmicutes	Bacilli	Bacillales	Bacillaceae	Bacillus	**Bacillus coagulans**	36D1	3552226	215.27	10061680
Bacteria	Firmicutes	Bacilli	Bacillales	Bacillaceae	Bacillus	**Bacillus cytotoxicus**	NVH 391 98	4087024	93.45	5025426
Bacteria	Firmicutes	Bacilli	Bacillales	Bacillaceae	Bacillus	**Bacillus halodurans**	C 125	4202352	47.38	2619835
Bacteria	Firmicutes	Bacilli	Bacillales	Bacillaceae	Bacillus	**Bacillus licheniformis**	ATCC 14580	4222597	123.48	6860686
Bacteria	Firmicutes	Bacilli	Bacillales	Bacillaceae	Bacillus	**Bacillus megaterium**	DSM319	5097447	30.00	2012150
Bacteria	Firmicutes	Bacilli	Bacillales	Bacillaceae	Bacillus	**Bacillus pseudofirmus**	OF4	3858997	303.41	15406030
Bacteria	Firmicutes	Bacilli	Bacillales	Bacillaceae	Bacillus	**Bacillus pumilus**	SAFR 032	3704465	30.00	1462288
Bacteria	Firmicutes	Bacilli	Bacillales	Bacillaceae	Bacillus	**Bacillus selenitireducens**	MLS10	3592487	161.57	7637344
Bacteria	Firmicutes	Bacilli	Bacillales	Bacillaceae	Bacillus	**Bacillus subtilis subsp. subtilis**	168	4214630	30.00	1664055
Bacteria	Firmicutes	Bacilli	Bacillales	Bacillaceae	Bacillus	**Bacillus thuringiensis**	Al Hakan	5257091	240.41	16698873
Bacteria	Firmicutes	Bacilli	Bacillales	Bacillaceae	Bacillus	**Bacillus tusciae**	DSM 2912	3384766	30.00	1336092
Bacteria	Firmicutes	Bacilli	Bacillales	Bacillaceae	Bacillus	**Bacillus weihenstephanensis**	KBAB4	5262775	30.00	2077411
Bacteria	Firmicutes	Bacilli	Bacillales	Bacillaceae	Geobacillus	**Geobacillus kaustophilus**	HTA426	3544776	30.00	1399254
Bacteria	Firmicutes	Bacilli	Bacillales	Bacillaceae	Geobacillus	**Geobacillus thermoglucosidasius**	C56 YS93	3893306	42.95	2200230

Table S5 : Taxonomic profile of the genus level training dataset for the order target assembly dataset.

Domain	Phylum	Class	Order	Family	Genus	Species	Strain	Length	Coverage	# read pairs
Bacteria	Proteobacteria	Alphaproteobacteria	Caulobacterales	Caulobacteraceae	Caulobacter	Caulobacter segnis	ATCC21756	4655622	90.68	5554892
Bacteria	Proteobacteria	Alphaproteobacteria	Rhizobiales	Rhizobiaceae	Rhizobium	Rhizobium leguminosarum bv.trifolii	WSM1325	4767043	30	1881728
Bacteria	Proteobacteria	Alphaproteobacteria	Rhodobacterales	Rhodobacteraceae	Ketogulonicigenium	Ketogulonicigenium vulgare	Y25	2776084	224.47	8199310
Bacteria	Proteobacteria	Alphaproteobacteria	Parvularculales	Parvularculaceae	Parvularcula	Parvularcula oceani	JLT2013	500140	57.69	379646
Bacteria	Proteobacteria	Alphaproteobacteria	Rhodospirillales	Rhodospirillaceae	Azospirillum	Azospirillum brasilense	Az39	3064393	48.6	1970082
Bacteria	Proteobacteria	Alphaproteobacteria	Sphingomonadales	Erythrobacteraceae	Erythrobacter	Erythrobacter	NAP1	3265065	30	1288842
Bacteria	Proteobacteria	Gammaproteobacteria	Acidithiobacillales	Acidithiobacillaceae	Acidithiobacillus	Acidithiobacillus caldus	ATCC51756	2777717	356.34	13023838
Bacteria	Proteobacteria	Gammaproteobacteria	Aeromonadales	Aeromonadaceae	Aeromonas	Aeromonas salmonicida	A449	4702402	30	1856212
Bacteria	Proteobacteria	Gammaproteobacteria	Alteromonadales	Alteromonadaceae	Alteromonas	Alteromonas australica	H17	4308833	54.35	3081382
Bacteria	Proteobacteria	Gammaproteobacteria	Cardiobacteriales	Cardiobacteriaceae	Cardiobacterium	Cardiobacterium hominis	ATCC15826	797982	45.97	482674
Bacteria	Proteobacteria	Gammaproteobacteria	Chromatiales	Ectothiorhodospiraceae	Halorhodospira	Halorhodospira halophila	SL1	2678452	30	1057284
Bacteria	Proteobacteria	Gammaproteobacteria	Enterobacteriales	Enterobacteriaceae	Escherichia	Escherichia albertii	TW0762	781636	144.19	1482948
Bacteria	Proteobacteria	Gammaproteobacteria	Legionellales	Legionellaceae	Legionella	Legionella longbeachae	NSW150	4077332	30	1609474
Bacteria	Proteobacteria	Gammaproteobacteria	Methylococcales	Methylococcaceae	Methylococcus	Methylomicrobium agile	ATCC35068	3660293	134.78	6491240
Bacteria	Proteobacteria	Gammaproteobacteria	Oceanospirillales	Halomonadaceae	Halomonas	Halomonas elongata	DSM2581	4061296	30	1603144
Bacteria	Proteobacteria	Gammaproteobacteria	Pasteurellales	Pasteurellaceae	Pasteurella	Pasteurella dagmatis	ATCC43325	308287	100.76	408724
Bacteria	Proteobacteria	Gammaproteobacteria	Pseudomonadales	Pseudomonadaceae	Pseudomonas	Pseudomonas entomophila	L48	5888780	87.97	6816262
Bacteria	Proteobacteria	Gammaproteobacteria	Thiotrichales	Piscirickettsiaceae	Thiomicrospira	Thiomicrospira kuenenii	DSM12350	1421009	99.93	1868440
Bacteria	Proteobacteria	Gammaproteobacteria	Vibrionales	Vibrionaceae	Vibrio	Vibrio ordalii	FF93	540137	174.74	1241888
Bacteria	Proteobacteria	Gammaproteobacteria	Xanthomonadales	Xanthomonadaceae	Xanthomonas	Xanthomonas axonopodis	Xac291	5153455	275.25	18664322

Table S6 : Taxonomic profile of the family level training dataset for the order target assembly dataset.

Domain	Phylum	Class	Order	Family	Genus	Species	Strain	Length	Coverage	# read pairs
Bacteria	Proteobacteria	Alphaproteobacteria	Caulobacterales	Caulobacteraceae	Brevundimonas	Brevundimonas subvibrioides	ATCC15264	3445263	90.68	4110742
Bacteria	Proteobacteria	Alphaproteobacteria	Rhizobiales	Rhizobiaceae	Agrobacterium	Agrobacterium vitis	S4	3726375	30	1470938
Bacteria	Proteobacteria	Alphaproteobacteria	Rhodobacterales	Rhodobacteraceae	Jannaschia	Jannaschia sp.	CCS1	4317977	224,47	12753372
Bacteria	Proteobacteria	Gammaproteobacteria	Enterobacteriales	Enterobacteriaceae	Thioalkalivibrio	Thioalkalivibrio sulfidophilus	HLEbGr7	3464554	57.69	2629870
Bacteria	Proteobacteria	Alphaproteobacteria	Rhodospirillales	Rhodospirillaceae	Magnetospirillum	Magnetospirillum magneticum	AMB1DNA	4967148	48.6	3193354
Bacteria	Proteobacteria	Gammaproteobacteria	Enterobacteriales	Enterobacteriaceae	Cronobacter	Cronobacter sakazakii	ATCCBAA89	4368373	30	1724358
Bacteria	Proteobacteria	Gammaproteobacteria	Acidithiobacillales	Acidithiobacillaceae	Saccharophagus	Saccharophagus degradans	240	5057531	356.34	23713166
Bacteria	Proteobacteria	Gammaproteobacteria	Aeromonadales	Aeromonadaceae	Tolumonas	Tolumonas auensis	DSM9187	3471292	30	1370246
Bacteria	Proteobacteria	Gammaproteobacteria	Alteromonadales	Alteromonadaceae	Marinobacter	Marinobacter aquaeolei	VT8	4326849	54.35	3094266
Bacteria	Proteobacteria	Gammaproteobacteria	Cardiobacteriales	Cardiobacteriaceae	Coriobacterium	Coriobacterium glomerans	PW2DSM20642	2115681	45.97	1279708
Bacteria	Proteobacteria	Gammaproteobacteria	Chromatiales	Ectothiorhodospiraceae	Halorhodospira	Halorhodospira halophila	SL1	2678452	30	1057284
Bacteria	Proteobacteria	Gammaproteobacteria	Enterobacteriales	Enterobacteriaceae	Erwinia	Erwinia tasmaniensis	Et199	3883467	144.19	7367856
Bacteria	Proteobacteria	Gammaproteobacteria	Enterobacteriales	Enterobacteriaceae	Dickeya	Dickeya dadantii	Ech703	4679450	30	1847152
Bacteria	Proteobacteria	Gammaproteobacteria	Methylococcales	Methylococcaceae	Methylomonas	Methylomonas mecanica	MC09	5051681	134.78	8958758
Bacteria	Proteobacteria	Gammaproteobacteria	Oceanospirillales	Halomonadaceae	Halomonas	Halomonas elongata	DSM2581	4061296	30	1603144
Bacteria	Proteobacteria	Gammaproteobacteria	Pasteurellales	Pasteurellaceae	Mannheimia	Mannheimia haemolytica	USDAARSUSM ARC183	2658332	100.76	3524388
Bacteria	Proteobacteria	Gammaproteobacteria	Pseudomonadales	Pseudomonadaceae	Azotobacter	Azotobacter vinelandii	DJ	5365318	87.97	6210356
Bacteria	Proteobacteria	Gammaproteobacteria	Thiotrichales	Piscirickettsiaceae	Cycloclasticus	Cycloclasticus pugetii	PS1	2383924	99.93	3134546
Bacteria	Proteobacteria	Gammaproteobacteria	Vibrionales	Vibrionaceae	Photobacterium	Photobacterium profundum	SS9	4085304	174.74	9392974
Bacteria	Proteobacteria	Gammaproteobacteria	Xanthomonadales	Xanthomonadaceae	Xylella	Xylella fastidiosa	9a5c	2679306	275.25	9703670

Table S7 : Taxonomic profile of the order level training dataset for the order target assembly dataset.

Domain	Phylum	Class	Order	Family	Genus	Species	Strain	Length	Coverage	# read pairs
Bacteria	Proteobacteria	Alphaproteobacteria	Rhodospirillales	Acetobacteraceae	Acidiphilium	Acidiphilium cryptum	JF5	3389227	90.68	4261852
Bacteria	Proteobacteria	Alphaproteobacteria	Rhizobiales	Beijerinckiaceae	Methylocella	Methylocella silvestris	BL2	4305430	30	1799538
Bacteria	Proteobacteria	Alphaproteobacteria	Rhodobacterales	Hyphomonadaceae	Hirschia	Hirschia baltica	ATCC49814	3455622	224.47	10857460
Bacteria	Proteobacteria	Gammaproteobacteria	Rhodospirillales	Acetobacteraceae	Gluconacetobacter	Gluconacetobacter diazotrophicus	PAl5	3887492	57.69	3113900
Bacteria	Proteobacteria	Alphaproteobacteria	Rhodospirillales	Acetobacteraceae	Gluconobacter	Gluconobacter oxydans	H24	3602424	48.6	2466980
Bacteria	Proteobacteria	Gammaproteobacteria	Legionellales	Coxiellaceae	Coxiella	Coxiella burnetii	CbuGQ212	2008870	30	825294
Bacteria	Proteobacteria	Gammaproteobacteria	Alteromonadales	Colwelliaceae	Colwellia	Colwellia psychrerythraea	34H	5373180	356.34	26898762
Bacteria	Proteobacteria	Gammaproteobacteria	Aeromonadales	Pseudoalteromonadaceae	Pseudoalteromonas	Pseudoalteromonas atlantica	T6c	5187005	30	2189426
Bacteria	Proteobacteria	Gammaproteobacteria	Alteromonadales	Shewanellaceae	Shewanella	Shewanella amazonensis	SB2B	4306142	54.35	3307388
Bacteria	Proteobacteria	Gammaproteobacteria	Oceanospirillales	Oceanospirillaceae	Marinomonas	Marinomonas posidonica	IVIAPo181	3899940	45.97	2481420
Bacteria	Proteobacteria	Gammaproteobacteria	Chromatiales	Ectothiorhodospiraceae	Alkalilimni	Alkalilimni colaehrlichii	MLHE-1	3526903	30	1504260
Bacteria	Proteobacteria	Alphaproteobacteria	Rhodobacterales	Rhodobacteraceae	Jannaschia	Jannaschia sp.	CCS1	4317977	144.19	8556126
Bacteria	Proteobacteria	Alphaproteobacteria	Sphingomonadales	Sphingomonadaceae	Novosphingobium	Novosphingobium aromaticivorans	DSM12444	3561584	30	1495822
Bacteria	Proteobacteria	Gammaproteobacteria	Pseudomonadales	Moraxellaceae	Acinetobacter	Acinetobacter baumannii	AC12	3846404	134.78	7175044
Bacteria	Proteobacteria	Gammaproteobacteria	Oceanospirillales	Hahellaceae	Hahella	Hahella chejuensis	KCTC2396	7215267	30	3053308
Bacteria	Proteobacteria	Gammaproteobacteria	Pseudomonadales	Moraxellaceae	Psychrobacter	Psychrobacter arcticus	2734	2650701	100.76	3707430
Bacteria	Proteobacteria	Gammaproteobacteria	Pseudomonadales	Moraxellaceae	Moraxella	Moraxella catarrhalis	BBH18	1863286	87.97	2278128
Bacteria	Proteobacteria	Gammaproteobacteria	Thiotrichales	Francisellaceae	Francisella	Francisella tularensis	SCHUS4	1892775	99.93	2589926
Bacteria	Proteobacteria	Alphaproteobacteria	Rhizobiales	Beijerinckiaceae	Beijerinckia	Beijerinckia indica	ATCC9039	4170153	174.74	10093256
Bacteria	Proteobacteria	Alphaproteobacteria	Rhizobiales	Bartonellaceae	Bartonella	Bartonella bacilliformis	KC583	1445021	275.25	5233448

Table S8 : Taxonomic profile of the genus level training dataset for the family target assembly dataset.

Domain	Phylum	Class	Order	Family	Genus	Species	Strain	Length	Coverage	# read pairs
Bacteria	Proteobacteria	Alphaproteobacteria	Rhizobiales	Bartonellaceae	Bartonella	Bartonella clarridgeiae	73	1522743	62.42	1250652
Bacteria	Proteobacteria	Alphaproteobacteria	Rhizobiales	Beijerinckiaceae	Beijerinckia	Beijerinckia indica	ATCC9039	4170153	15.00	823056
Bacteria	Proteobacteria	Alphaproteobacteria	Rhizobiales	Bradyrhizobiaceae	Nitrobacter	Nitrobacter winogradskyi	Nb255	3402093	15.00	671466
Bacteria	Proteobacteria	Alphaproteobacteria	Rhizobiales	Brucellaceae	Brucella	Brucella pinnipedialis	B294	2138342	15.00	422042
Bacteria	Proteobacteria	Alphaproteobacteria	Rhizobiales	Hyphomicrobiaceae	Hyphomicrobium	Hyphomicrobium	MC1	4757528	15.08	944306
Bacteria	Proteobacteria	Alphaproteobacteria	Rhizobiales	Methylobacteriaceae	Methylobacterium	Methylobacterium extorquens	AM1	5511322	242.37	17576040
Bacteria	Proteobacteria	Alphaproteobacteria	Rhizobiales	Phyllobacteriaceae	Mesorhizobium	Mesorhizobium ciceri biovar biserrulae	WSM1271	6264489	73.67	6072432
Bacteria	Proteobacteria	Alphaproteobacteria	Rhizobiales	Rhizobiaceae	Rhizobium	Rhizobium leguminosarum trifolii	WSM1325	4767043	15.59	978184
Bacteria	Proteobacteria	Alphaproteobacteria	Rhizobiales	Xanthobacteraceae	Azorhizobium	Azorhizobium doebereinerae	UFLA1100	581759	50.53	386794
Bacteria	Proteobacteria	Gammaproteobacteria	Alteromonadales	Alteromonadaceae	Alteromonas	Alteromonas australica	H17	4308833	15.00	850428
Bacteria	Proteobacteria	Gammaproteobacteria	Alteromonadales	Colwelliaceae	Colwellia	Colwellia sp.	MT41	253088	206.61	688034
Bacteria	Proteobacteria	Gammaproteobacteria	Alteromonadales	Ferrimonadaceae	Ferrimonas	Ferrimonas senticii	DSM18821	763770	60.13	604322
Bacteria	Proteobacteria	Gammaproteobacteria	Alteromonadales	Idiomarinaceae	Idiomarina	Idiomarina baltica	OS145	281428	29.28	108424
Bacteria	Proteobacteria	Gammaproteobacteria	Alteromonadales	Pseudoalteromonadaceae	Pseudoalteromonas	Pseudoalteromonas atlantica	T6c	5187005	20.29	1384794
Bacteria	Proteobacteria	Gammaproteobacteria	Alteromonadales	Psychromonadaceae	Psychromonas	Psychromonas sp.	CNPT3	3052410	70.69	2839142
Bacteria	Proteobacteria	Gammaproteobacteria	Alteromonadales	Shewanellaceae	Shewanella	Shewanella loihica	PV4	4602594	24.57	1488272
Bacteria	Firmicutes	Bacilli	Bacillales	Bacillaceae	Bacillus	Bacillus thuringiensis	AlHakam	5257091	28.10	1944016
Bacteria	Firmicutes	Bacilli	Bacillales	Listeriaceae	Listeria	Listeria innocua	Clip11262	3011208	19.04	754386
Bacteria	Firmicutes	Bacilli	Bacillales	Paenibacillaceae	Brevibacillus	Brevibacillus laterosporus	DSM25	253088	27.95	93076
Bacteria	Firmicutes	Bacilli	Bacillales	Alicyclobacillaceae	Alicyclobacillus	Alicyclobacillus macrosporangiidus	CPP55	4073483	17.45	935562

Table S9 : Taxonomic profile of the family level training dataset for the family target assembly dataset.

Domain	Phylum	Class	Order	Family	Genus	Species	Strain	Length	Coverage	# read pairs
Bacteria	Firmicutes	Bacilli	Bacillales	Bacillaceae	Anoxybacillus	Anoxybacillus flavithermus	WK1	2846746	62.42	2338078
Bacteria	Proteobacteria	Alphaproteobacteria	Rhizobiales	Beijerinckiaceae	Methylocella	Methylocella silvestris	BL2	4305430	15.00	849756
Bacteria	Proteobacteria	Alphaproteobacteria	Rhizobiales	Bradyrhizobiaceae	Oligotropha	Oligotropha carboxidovorans	OM5	3595748	15.00	709688
Bacteria	Proteobacteria	Alphaproteobacteria	Rhizobiales	Brucellaceae	Brucella	Ochrobactrum anthropi	ATCC49188	2887297	15.00	569862
Bacteria	Proteobacteria	Alphaproteobacteria	Rhizobiales	Hyphomicrobiaceae	Rhodomicrobium	Rhodomicrobium vannielii	ATCC17100	4014469	15.08	796820
Bacteria	Proteobacteria	Alphaproteobacteria	Rhizobiales	Methylobacteriaceae	Microvirga	Microvirga sp.	WSM3557	2401580	242.37	7658828
Bacteria	Proteobacteria	Alphaproteobacteria	Rhizobiales	Phyllobacteriaceae	Parvibaculum	Parvibaculum Lavamentivorans	DS1	3914745	73.67	3794728
Bacteria	Proteobacteria	Alphaproteobacteria	Rhizobiales	Rhizobiaceae	Agrobacterium	Agrobacterium vitis	S4	3726375	15.59	764642
Bacteria	Proteobacteria	Alphaproteobacteria	Rhizobiales	Xanthobacteraceae	Starkeya	Starkeya novella	DSM506	4765023	50.53	3168114
Bacteria	Proteobacteria	Gammaproteobacteria	Alteromonadales	Alteromonadaceae	Marinobacter	Marinobacter aquaeolei	VT8	4326849	15.00	853984
Bacteria	Firmicutes	Bacilli	Bacillales	Bacillaceae	Exiguobacterium	Exiguobacterium sp.	GIC31	2918587	206.61	7934332
Bacteria	Firmicutes	Bacilli	Bacillales	Bacillaceae	Gracilibacillus	Gracilibacillus lacisalsi	DSM19029	513257	60.13	406108
Bacteria	Proteobacteria	Alphaproteobacteria	Rhizobiales	Rhizobiaceae	Candidatus liberibacter	Candidatus liberibacter asiaticus	Psy62	1227328	29.28	496454
Bacteria	Proteobacteria	Alphaproteobacteria	Rhizobiales	Bradyrhizobiaceae	Oligotropha	Oligotropha carboxidovorans	OM4	3539447	20.29	997228
Bacteria	Proteobacteria	Alphaproteobacteria	Rhizobiales	Xanthobacteraceae	Xanthobacter	Xanthobacter autotrophicus	Py2	5308934	70.69	5223568
Bacteria	Proteobacteria	Gammaproteobacteria	Pseudomonadales	Pseudomonadaceae	Pseudomonas	Pseudomonas aeruginosa	NCGM2S1	6764661	24.57	2321500
Bacteria	Firmicutes	Bacilli	Bacillales	Bacillaceae	Amphibacillus	Amphibacillus xylanus	NBRC15112	2569486	28.10	950168
Bacteria	Proteobacteria	Gammaproteobacteria	Alteromonadales	Alteromonadaceae	Saccharophagus	Saccharophagus degradans	240	5057531	19.04	1377368
Bacteria	Firmicutes	Bacilli	Bacillales	Paenibacillaceae	Paenibacillus	Paenibacillus sp.	Y412MC10	7121665	27.95	2619086
Bacteria	Firmicutes	Bacilli	Bacillales	Alicyclobacillaceae	Kyrpidia	Kyrpidia tusciae	T2DSM2912	3384766	17.45	777382

Table S10 : Taxonomic profile of the ordo level training dataset for the family target assembly dataset.

Domain	Phylum	Class	Order	Family	Genus	Species	Strain	Length	Coverage	# read pairs
Bacteria	Proteobacteria	Gammaproteobacteria	Enterobacteriales	Enterobacteriaceae	Escherichia	Escherichia albertii	TW0762	781636	62.42	641970
Bacteria	Proteobacteria	Alphaproteobacteria	Rhodospirillales	Rhodospirillaceae	Azospirillum	Azospirillum brasilense	Az39	3064393	15.00	604814
Bacteria	Proteobacteria	Gammaproteobacteria	Vibrionales	Vibrionaceae	Vibrio	Vibrio ordalii	FF93	540137	15.00	106606
Bacteria	Proteobacteria	Gammaproteobacteria	Pseudomonadales	Pseudomonadaceae	Pseudomonas	Pseudomonas entomophila	L48	5888780	15.00	1162260
Bacteria	Proteobacteria	Gammaproteobacteria	Oceanospirillales	Halomonadaceae	Halomonas	Halomonas elongata	DSM2581	4061296	15.08	806114
Bacteria	Proteobacteria	Gammaproteobacteria	Pasteurellales	Pasteurellaceae	Pasteurella	Pasteurella dagmatis	ATCC43325	308287	242.37	983152
Bacteria	Firmicutes	Bacilli	Bacillales	Staphylococcaceae	Staphylococcus	Staphylococcus aureus	MSSA476	2799802	73.67	2831132
Bacteria	Proteobacteria	Gammaproteobacteria	Aeromonadales	Aeromonadaceae	Aeromonas	Aeromonas salmonicida	A449	4702402	15.59	964920
Bacteria	Proteobacteria	Alphaproteobacteria	Caulobacterales	Caulobacteraceae	Caulobacter	Caulobacter segnis	ATCC21756	4655622	50.53	3095376
Bacteria	Firmicutes	Bacilli	Bacillales	Staphylococcaceae	Staphylococcus	Staphylococcus carnosus	TM300	2566424	15.00	527610
Bacteria	Proteobacteria	Gammaproteobacteria	Thiotrichales	Piscirickettsiaceae	Thiomicrospira	Thiomicrospira kuenenii	DSM12350	1421009	206.61	3863088
Bacteria	Proteobacteria	Gammaproteobacteria	Methylococcales	Methylococcaceae	Methylococcus	Methylomicrobium agile	ATCC35068	3660293	60.13	2896158
Bacteria	Proteobacteria	Gammaproteobacteria	Legionellales	Legionellaceae	Legionella	Legionella longbeachae	NSW150	4077332	29.28	1570846
Bacteria	Firmicutes	Bacilli	Bacillales	Staphylococcaceae	Staphylococcus	Staphylococcus haemolyticus	JCSC1435	2685015	20.29	747504
Bacteria	Firmicutes	Bacilli	Bacillales	Staphylococcaceae	Firmicutes	Macrococcus caseolyticus	JCSC5402	2102324	24.57	679798
Bacteria	Proteobacteria	Alphaproteobacteria	Sphingomonadales	Erythrobacteraceae	Erythrobacter	Erythrobacter sp.	NAP1	3265065	28.10	1207386
Bacteria	Proteobacteria	Alphaproteobacteria	Rhizobiales	Methylocystaceae	Methylocystis	Methylocystis sp.	SC2	3773444	19.04	945348
Bacteria	Proteobacteria	Gammaproteobacteria	Xanthomonadales	Xanthomonadaceae	Xanthomonas	Xanthomonas axonopodis	Xac291	5153455	27.95	1895250
Bacteria	Proteobacteria	Gammaproteobacteria	Acidithiobacillales	Acidithiobacillaceae	Acidithiobacillus	Acidithiobacillus caldus	ATCC51756	2777717	17.45	637962

Table S11 : Taxonomic profile of the genus level training dataset for the genus target assembly dataset.

Domain	Phylum	Class	Order	Family	Genus	Species	Strain	Length	Coverage	# read pairs
Bacteria	Proteobacteria	Gammaproteobacteria	Enterobacteriales	Enterobacteriaceae	Yersinia	Yersinia pseudotuberculosis	IP32953	4744671	390.28	24365134
Bacteria	Proteobacteria	Gammaproteobacteria	Enterobacteriales	Enterobacteriaceae	Candidatus korarchaeum	Candidatus korarchaeum cryptofilum	OPF8	1590757	243.89	5104864
Bacteria	Proteobacteria	Gammaproteobacteria	Enterobacteriales	Enterobacteriaceae	Sodalis	Sodalis sp.	HS1	3938516	92.53	4795144
Bacteria	Proteobacteria	Gammaproteobacteria	Enterobacteriales	Enterobacteriaceae	Citrobacter	Citrobacter rodentium	ICC168	5346659	30.00	2110524
Bacteria	Proteobacteria	Gammaproteobacteria	Enterobacteriales	Enterobacteriaceae	Cronobacter	Cronobacter turicensis	Z3032	4384463	30.00	1730710
Bacteria	Proteobacteria	Gammaproteobacteria	Enterobacteriales	Enterobacteriaceae	Dickeya	Dickeya zeae	Ech1591	4813854	166.48	10544874
Bacteria	Proteobacteria	Gammaproteobacteria	Enterobacteriales	Enterobacteriaceae	Edwardsiella	Edwardsiella tarda	EIB202	3760463	81.42	4028644
Bacteria	Proteobacteria	Gammaproteobacteria	Enterobacteriales	Enterobacteriaceae	Enterobacter	Enterobacter sp.	MGH14	3495166	72.73	3344782
Bacteria	Proteobacteria	Gammaproteobacteria	Enterobacteriales	Enterobacteriaceae	Erwinia	Erwinia amylovora	CFBP1430	3805573	30.00	1502200
Bacteria	Proteobacteria	Gammaproteobacteria	Enterobacteriales	Enterobacteriaceae	Escherichia	Escherichia albertii	TW0762	781636	81.31	836248
Bacteria	Proteobacteria	Gammaproteobacteria	Enterobacteriales	Enterobacteriaceae	Klebsiella	Klebsiella pneumoniae	342	5641239	30.00	2226804
Bacteria	Proteobacteria	Gammaproteobacteria	Enterobacteriales	Enterobacteriaceae	Pantoea	Pantoea ananatis	LMG20103	4703373	241.81	14964772
Bacteria	Proteobacteria	Gammaproteobacteria	Enterobacteriales	Enterobacteriaceae	Pectobacterium	Pectobacterium wasabiae	WPP163	5063892	30.00	1998904
Bacteria	Proteobacteria	Gammaproteobacteria	Enterobacteriales	Enterobacteriaceae	Photorhabdus	Photorhabdus luminescens laumondii	TTO1	5688987	63.75	4772012
Bacteria	Proteobacteria	Gammaproteobacteria	Enterobacteriales	Enterobacteriaceae	Proteus	Proteus mirabilis	HI4320	4063606	83.65	4472640
Bacteria	Proteobacteria	Gammaproteobacteria	Enterobacteriales	Enterobacteriaceae	Wigglesworthia	Wigglesworthia glossinidia	brevipalpis	697724	57.65	529260
Bacteria	Proteobacteria	Gammaproteobacteria	Enterobacteriales	Enterobacteriaceae	Salmonella	Salmonella enterica serovar agona	SL483	4798660	30.00	1894208
Bacteria	Proteobacteria	Gammaproteobacteria	Enterobacteriales	Enterobacteriaceae	Serratia	Serratia plymuthica	4Rx13	5328010	96.20	6744138
Bacteria	Proteobacteria	Gammaproteobacteria	Enterobacteriales	Enterobacteriaceae	Shigella	Shigella boydii	CDC308394	4615997	80.21	4871698
Bacteria	Proteobacteria	Gammaproteobacteria	Enterobacteriales	Enterobacteriaceae	Xenorhabdus	Xenorhabdus nematophila	ATCC19061	4432590	182.67	10653964

Table S12 : Taxonomic profile of the family level training dataset for the genus target assembly dataset.

Domain	Phylum	Class	Order	Family	Genus	Species	Strain	Length	Coverage	# read pairs
Bacteria	Proteobacteria	Gammaproteobacteria	Enterobacteriales	Enterobacteriaceae	Candidatus blochmannia	Candidatus blochmannia chromaiodes	640	791219	390.28	4063118
Bacteria	Proteobacteria	Gammaproteobacteria	Enterobacteriales	Enterobacteriaceae	Candidatus blochmannia	Candidatus blochmannia floridanus		705557	243.89	2264188
Bacteria	Proteobacteria	Gammaproteobacteria	Enterobacteriales	Enterobacteriaceae	Buchnera	Buchnera aphidicola	Sg	641454	92.53	780970
Bacteria	Proteobacteria	Gammaproteobacteria	Enterobacteriales	Enterobacteriaceae	Candidatus blochmannia	Candidatus blochmannia pennsylvanicus	BPEN	791654	30.00	312496
Bacteria	Proteobacteria	Gammaproteobacteria	Enterobacteriales	Enterobacteriaceae	Candidatus blochmannia	Candidatus blochmannia vafer	BVAF	722593	30.00	285234
Bacteria	Proteobacteria	Gammaproteobacteria	Enterobacteriales	Enterobacteriaceae	Hamiltonella	Hamiltonella defensa	5AT	2110331	166.48	4622736
Bacteria	Proteobacteria	Gammaproteobacteria	Enterobacteriales	Enterobacteriaceae	Candidatus riesia	Candidatus riesia pediculicola	USDA	574390	81.42	615354
Bacteria	Proteobacteria	Gammaproteobacteria	Enterobacteriales	Enterobacteriaceae	Morganella	Morganella morganii	KT	3799539	72.73	3636058
Bacteria	Proteobacteria	Gammaproteobacteria	Enterobacteriales	Enterobacteriaceae	Buchnera	Buchnera aphidicola	5A	642122	30.00	253470
Bacteria	Proteobacteria	Gammaproteobacteria	Enterobacteriales	Enterobacteriaceae	Raoultella	Raoultella ornithinolytica	B6	5398151	81.31	5775312
Bacteria	Proteobacteria	Gammaproteobacteria	Enterobacteriales	Enterobacteriaceae	Providencia	Providencia stuartii	MRSN2154	4402109	30.00	1737674
Bacteria	Proteobacteria	Gammaproteobacteria	Enterobacteriales	Enterobacteriaceae	Wigglesworthia	Wigglesworthia glossinidia	morsitans	719535	241.81	2289352
Bacteria	Proteobacteria	Gammaproteobacteria	Enterobacteriales	Enterobacteriaceae	Arsenophonus	Arsenophonus melophagi	ArM	1155312	30.00	456044
Bacteria	Proteobacteria	Gammaproteobacteria	Enterobacteriales	Enterobacteriaceae	Arsenophonus	Arsenophonus sp.	ArN	3575339	63.75	2999050
Bacteria	Proteobacteria	Gammaproteobacteria	Enterobacteriales	Enterobacteriaceae	Arsenophonus	Arsenophonus triatominarum	ArT	2619384	83.65	2883046
Bacteria	Proteobacteria	Gammaproteobacteria	Enterobacteriales	Enterobacteriaceae	Baumannia	Baumannia cicadellinicola	HC	686194	57.65	520514
Bacteria	Proteobacteria	Gammaproteobacteria	Enterobacteriales	Enterobacteriaceae	Wigglesworthia	Wigglesworthia glossinidia	brevipalpis	697724	30.00	275418
Bacteria	Proteobacteria	Gammaproteobacteria	Enterobacteriales	Enterobacteriaceae	Buchnera	Buchnera aphidicola	G002	643517	96.20	814558
Bacteria	Proteobacteria	Gammaproteobacteria	Enterobacteriales	Enterobacteriaceae	Buchnera	Buchnera aphidicola	JF98	641771	80.21	67732
Bacteria	Proteobacteria	Gammaproteobacteria	Enterobacteriales	Enterobacteriaceae	Buchnera	Buchnera aphidicola	Ua	615380	182.67	1479098

Table S13 : Taxonomic profile of the ordo level training dataset for the genus target assembly dataset.

Domain	Phylum	Class	Order	Family	Genus	Species	Strain	Length	Coverage	# read pairs
Bacteria	Proteobacteria	Gammaproteobacteria	Alteromonadales	Idiomarinaceae	Idiomarina	Idiomarina loihiensis	L2TR	2839318	390.28	14580646
Bacteria	Proteobacteria	Gammaproteobacteria	Aeromonadales	Aeromonadaceae	Aeromonas	Aeromonas salmonicida	A449	4702402	243.89	15090380
Bacteria	Proteobacteria	Gammaproteobacteria	Alteromonadales	Psychromonadaceae	Psychromonas	Psychromonas ingrahamii	37	4559598	92.53	5551310
Bacteria	Proteobacteria	Gammaproteobacteria	Aeromonadales	Aeromonadaceae	Aeromonas	Aeromonas hydrophila subsp. hydrophila	ATCC 7966	4744448	30.00	1872808
Bacteria	Proteobacteria	Gammaproteobacteria	Alteromonadales	Shewanellaceae	Shewanella	Shewanella oneidensis	MR-1	4969803	30.00	1961768
Bacteria	Proteobacteria	Gammaproteobacteria	Pasteurellales	Pasteurellaceae	Pasteurella	Pasteurella dagmatis	ATCC43325	308287	166.48	675310
Bacteria	Proteobacteria	Gammaproteobacteria	Vibrionales	Vibrionaceae	Vibrio	Vibrio ordalii	FF93	540137	81.42	578658
Bacteria	Proteobacteria	Gammaproteobacteria	Alteromonadales	Alteromonadaceae	Alteromonas	Alteromonas australica	H17	4308833	72.73	4123440
Bacteria	Proteobacteria	Gammaproteobacteria	Alteromonadales	Pseudoalteromonadac eae	Pseudoalteromonas	Pseudoalteromonas atlantica	T6c	5187005	30.00	2047502
Bacteria	Proteobacteria	Gammaproteobacteria	Alteromonadales	Idiomarinaceae	Idiomarina	Idiomarina baltica	OS145	281428	81.31	301090
Bacteria	Proteobacteria	Gammaproteobacteria	Alteromonadales	Shewanellaceae	Shewanella	Shewanella loihica	PV4	4602594	30.00	1816814
Bacteria	Proteobacteria	Gammaproteobacteria	Alteromonadales	Psychromonadaceae	Psychromonas	Psychromonas sp.	CNPT3	3052410	241.81	9711886
Bacteria	Proteobacteria	Gammaproteobacteria	Pseudomonadale s	Pseudomonadaceae	Pseudomonas	Pseudomonas entomophila	L48	5888780	30.00	2324518
Bacteria	Proteobacteria	Gammaproteobacteria	Pasteurellales	Pasteurellaceae	Pasteurella	Pasteurella multocida subsp. multocida	Pm70	2257487	63.75	1893616
Bacteria	Proteobacteria	Gammaproteobacteria	Pseudomonadale s	Pseudomonadaceae	Pseudomonas	Pseudomonas putida	W619	5774330	83.65	6355562
Bacteria	Proteobacteria	Gammaproteobacteria	Vibrionales	Vibrionaceae	Vibrio	Vibrio cholerae O1 biovar eltor	N16961	2961149	57.65	2246188
Bacteria	Proteobacteria	Gammaproteobacteria	Alteromonadales	Colwelliaceae	Colwellia	Colwellia psychrerythraea	34H	5373180	30.00	2120992
Bacteria	Proteobacteria	Gammaproteobacteria	Alteromonadales	Alteromonadaceae	Alteromonas	Alteromonas macleodii	Deep ecotype	4412282	96.20	5631472
Bacteria	Proteobacteria	Gammaproteobacteria	Alteromonadales	Ferrimonadaceae	Ferrimonas	Ferrimonas balearica	DSM 9799	4279159	80.21	4516202
Bacteria	Proteobacteria	Gammaproteobacteria	Alteromonadales	Pseudoalteromonadac eae	Pseudoalteromonas	Pseudoalteromonas haloplanktis	TAC125	3214944	182.67	7727288

Table S14 : Taxonomic profile of the genus level training dataset for the species target assembly dataset.

Domain	Phylum	Class	Order	Family	Genus	Species	Strain	Length	Coverage	# read pairs
Bacteria	Firmicutes	Bacilli	Bacillales	Bacillaceae	Bacillus	Bacillus aerophilus	KACC16563	302985	92.04	366930
Bacteria	Firmicutes	Bacilli	Bacillales	Bacillaceae	Bacillus	Bacillus alcalophilus	ATCC27647	66087	270.68	235374
Bacteria	Firmicutes	Bacilli	Bacillales	Bacillaceae	Bacillus	Bacillus acidiproducens	DSM23148	176059	30.04	69590
Bacteria	Firmicutes	Bacilli	Bacillales	Bacillaceae	Bacillus	Bacillus aurantiacus	DSM18675	1812115	100.35	2392708
Bacteria	Firmicutes	Bacilli	Bacillales	Bacillaceae	Bacillus	Bacillus aidingensis	DSM18341	442710	31.46	183258
Bacteria	Firmicutes	Bacilli	Bacillales	Bacillaceae	Bacillus	Bacillus altitudinis	41KF2b	776827	114.47	1170044
Bacteria	Firmicutes	Bacilli	Bacillales	Bacillaceae	Bacillus	Bacillus sp.	B14905	311732	215.27	882980
Bacteria	Firmicutes	Bacilli	Bacillales	Bacillaceae	Bacillus	Bacillus azotoformans	LMG9581	275569	93.45	338842
Bacteria	Firmicutes	Bacilli	Bacillales	Bacillaceae	Bacillus	Bacillus bogoriensis	ATCCBAA922	529031	47.38	329810
Bacteria	Firmicutes	Bacilli	Bacillales	Bacillaceae	Bacillus	Bacillus chagannorensis	DSM18086	496250	123.48	806276
Bacteria	Firmicutes	Bacilli	Bacillales	Bacillaceae	Bacillus	Bacillus bataviensis	LMG21833	234467	30.00	92552
Bacteria	Firmicutes	Bacilli	Bacillales	Bacillaceae	Bacillus	Bacillus firmus	DS1	469348	303.41	1873748
Bacteria	Firmicutes	Bacilli	Bacillales	Bacillaceae	Bacillus	Bacillus mycoides	DSM2048	231697	30.00	91460
Bacteria	Firmicutes	Bacilli	Bacillales	Bacillaceae	Bacillus	Bacillus fordii	DSM16014	763255	161.57	1622620
Bacteria	Firmicutes	Bacilli	Bacillales	Bacillaceae	Bacillus	Bacillus coahuilensis	M4-4	256263	30.00	101156
Bacteria	Firmicutes	Bacilli	Bacillales	Bacillaceae	Bacillus	Lysinibacillus fusiformis	H1k	138825	240.41	439144
Bacteria	Firmicutes	Bacilli	Bacillales	Bacillaceae	Bacillus	Bacillus pseudomycoides	DSM12442	466454	30.00	184126
Bacteria	Firmicutes	Bacilli	Bacillales	Bacillaceae	Bacillus	Bacillus timonensis	MM10403188	261647	30.00	103282
Bacteria	Firmicutes	Bacilli	Bacillales	Bacillaceae	Geobacillus	Lysinibacillus sphaericus	C341	4125813	30.00	1628610
Bacteria	Firmicutes	Bacilli	Bacillales	Bacillaceae	Geobacillus	Bacillus subtilis	NattoBEST195	3320579	42.95	1876564

Table S15 : Taxonomic profile of the family level training dataset for the species target assembly dataset.

Domain	Phylum	Class	Order	Family	Genus	Species	Strain	Length	Coverage	# read pairs
Bacteria	Firmicutes	Bacilli	Bacillales	Bacillaceae	Lysinibacillus	Lysinibacillus sp.	GY32	4662822	92.04	5646922
Bacteria	Firmicutes	Bacilli	Bacillales	Bacillaceae	Anoxybacillus	Anoxybacillus flavithermus	WK1	2846746	270.68	10138910
Bacteria	Firmicutes	Bacilli	Bacillales	Bacillaceae	Amphibacillus	Amphibacillus xylanus	NBRC15112	2569486	30.04	1015624
Bacteria	Firmicutes	Bacilli	Bacillales	Bacillaceae	Exiguobacterium	Exiguobacterium sp.	GIC31	2918587	100.35	3853686
Bacteria	Firmicutes	Bacilli	Bacillales	Bacillaceae	Paucisalibacillus	Paucisalibacillus globulus	DSM18846	670977	31.46	277750
Bacteria	Firmicutes	Bacilli	Bacillales	Bacillaceae	Oceanobacillus	Oceanobacillus iheyensis	HTE831	3630528	114.47	5468244
Bacteria	Firmicutes	Bacilli	Bacillales	Bacillaceae	Exiguobacterium	Exiguobacterium	MH3	3164195	215.27	8962582
Bacteria	Firmicutes	Bacilli	Bacillales	Bacillaceae	Lysinibacillus	Lysinibacillus sphaericus	C341	4639821	93.45	14677096
Bacteria	Firmicutes	Bacilli	Bacillales	Bacillaceae	Gracilibacillus	Gracilibacillus lacisalsi	DSM19029	513257	47.38	319974
Bacteria	Firmicutes	Bacilli	Bacillales	Bacillaceae	Pontibacillus	Pontibacillus halophilus	DSM19796	513663	123.48	834566
Bacteria	Firmicutes	Bacilli	Bacillales	Bacillaceae	Halalkalibacillus	Halalkalibacillus halophilus	DSM18494	568519	30.00	224416
Bacteria	Firmicutes	Bacilli	Bacillales	Bacillaceae	Salimicrobium	Salimicrobium sp.	MJ3	271263	303.41	1082946
Bacteria	Firmicutes	Bacilli	Bacillales	Bacillaceae	Halobacillus	Halobacillus halophilus	DSM2266	4150632	30.00	1638408
Bacteria	Firmicutes	Bacilli	Bacillales	Bacillaceae	Salsuginibacillus	Salsuginibacillus kocurii	DSM18087	886923	161.57	1885528
Bacteria	Firmicutes	Bacilli	Bacillales	Bacillaceae	Halobacillus	Halobacillus kuroshimensis	DSM18393	1160998	30.00	458288
Bacteria	Firmicutes	Bacilli	Bacillales	Bacillaceae	Lysinibacillus	Lysinibacillus sphaericus	C341	4639821	240.41	14677096
Bacteria	Firmicutes	Bacilli	Bacillales	Bacillaceae	Lentibacillus	Lentibacillus sp.	Grbi	673304	30.00	265778
Bacteria	Firmicutes	Bacilli	Bacillales	Bacillaceae	Virgibacillus	Virgibacillus sp.	CM4	1627377	30.00	642386
Bacteria	Firmicutes	Bacilli	Bacillales	Bacillaceae	Lysinibacillus	Lysinibacillus fusiformis	RB21	4830949	30.00	1906954
Bacteria	Firmicutes	Bacilli	Bacillales	Bacillaceae	Lysinibacillus	Lysinibacillus fusiformis	ZC1	362838	42.95	820208

Table S16 : Taxonomic profile of the order level training dataset for the species target assembly dataset.

Domain	Phylum	Class	Order	Family	Genus	Species	Strain	Length	Coverage	# read pairs
Bacteria	Firmicutes	Bacilli	Bacillales	Alicyclobacillaceae	Alicyclobacillus	Alicyclobacillus acidocaldarius	Tc41	3124048	92.04	3932122
Bacteria	Firmicutes	Bacilli	Bacillales	Paenibacillaceae	Brevibacillus	Brevibacillus brevis	NBRC 100599	6296436	270.68	104766
Bacteria	Firmicutes	Bacilli	Bacillales	Alicyclobacillaceae	Alicyclobacillus	Alicyclobacillus acidocaldarius subsp. acidocaldarius	DSM 446	3018755	30.04	3932122
Bacteria	Firmicutes	Bacilli	Bacillales	Alicyclobacillaceae	Alicyclobacillus	Alicyclobacillus macrosporangiidus	CPP55	4073483	100.35	5378606
Bacteria	Firmicutes	Bacilli	Bacillales	Paenibacillaceae	Brevibacillus	Brevibacillus laterosporus	DSM25	253088	31.46	104766
Bacteria	Firmicutes	Bacilli	Bacillales	Listeriaceae	Listeria	Listeria innocua	Clip11262	3011208	114.47	4535434
Bacteria	Firmicutes	Bacilli	Bacillales	Listeriaceae	Listeria	Listeria ivanovii	PAM55	2928879	215.27	8296050
Bacteria	Firmicutes	Bacilli	Bacillales	Paenibacillaceae	Paenibacillus	Paenibacillus sp.	Y412MC10	7121665	93.45	8756836
Bacteria	Proteobacteria	Gammaproteobacteria	Alteromonadales	Alteromonadaceae	Saccharophagus	Saccharophagus degradans	240	5057531	47.38	3427518
Bacteria	Firmicutes	Bacilli	Bacillales	Listeriaceae	Listeria	Listeria seeligeri	SerovarSLCC3954	2797636	123.48	4790988
Bacteria	Firmicutes	Bacilli	Bacillales	Listeriaceae	Listeria	Listeria monocytogenes serotype 4b	F2365	2905187	30.00	1146784
Bacteria	Firmicutes	Bacilli	Bacillales	Listeriaceae	Listeria	Listeria welshimeri	Serovar6bSLCC5334	2814130	303.41	11790306
Bacteria	Firmicutes	Bacilli	Bacillales	Alicyclobacillaceae	Kyrpidia	Kyrpidia tusciae	T2DSM2912	3384766	30.00	1336092
Bacteria	Firmicutes	Bacilli	Bacillales	Listeriaceae	Listeria	Listeria monocytogenes	L312	2912346	161.57	6191418
Bacteria	Firmicutes	Bacilli	Bacillales	Paenibacillaceae	Paenibacillus	Paenibacillus polymyxa	CR1	6024666	30.00	2498596
Bacteria	Firmicutes	Bacilli	Bacillales	Paenibacillaceae	Paenibacillus	Paenibacillus sp.	JDR2	7184930	240.41	24031692
Bacteria	Firmicutes	Bacilli	Bacillales	Listeriaceae	Listeria	Listeria monocytogenes	M7	2976163	30.00	1235572
Bacteria	Firmicutes	Bacilli	Bacillales	Paenibacillaceae	Paenibacillus	Paenibacillus terrae	HPL003	6083395	30.00	2525250
Bacteria	Firmicutes	Bacilli	Bacillales	Paenibacillaceae	Paenibacillus	Paenibacillus sabinae	T27	5270569	30.00	2173614
Bacteria	Firmicutes	Bacilli	Bacillales	Paenibacillaceae	Paenibacillus	Paenibacillus mucilaginosus	K02	8819200	42.95	4984008

Table S17 : The taxonomic profile predicted by MetaPhlAn for the order target assembly dataset

No	Species	Coverage
1.	Acidithiobacillus ferrooxidans	295.12
2.	Xanthomonas campestris	212.08
3.	Dinoroseobacter shibae	180.12
4.	Methylococcus capsulatus	100.45
5.	Escherichia coli	88.54
6.	Pasteurella multocida	83.76
7.	Vibrio cholerae	81.47
8.	Caulobacter crescentus	78.10
9.	Thiomicrospira crunogena	73.09
10.	Parvularcula bermudensis	42.37
11.	Dichelobacter nodosus	33.56
12.	Aeromonas hydrophila	24.76
13.	Legionella pneumophila	24.65
14.	Chromohalobacter salexigens	24.29
15.	Alkalilimnicola ehrlichei	23.59
16.	Erythrobacter litoralis	22.55
17.	Pseudomonas putida	10.26
18.	Alteromonas macleodii	5.21

Table S18 : The taxonomic profile predicted by MetaPhlAn for the family target assembly dataset

No	Species	Coverage
1.	Methylocella silvestris	351.47
2.	Nitrobacter hamburgensis	188.13
3.	Mesorhizobium loti	124.20
4.	Bartonella bacilliformis	110.20
5.	Colwellia psychrerythraea	109.76
6.	Bacillus subtilis	93.82
7.	Ferrimonas balearica	93.18
8.	Azorhizobium caulinodans	48.50
9.	Idiomarina loihiensis	44.20
10.	Shewanella oneidensis	42.79
11.	Brevibacillus brevis	41.38
12.	Pseudoalteromonas haloplanktis	33.16
13.	Listeria monocytogenes	28.33
14.	Psychromonas ingrahamii	24.38
15.	Methylobacterium chloromethanicum	24.12
16.	Hyphomicrobium denitrificans	23.14
17.	Alicyclobacillus alicyclobacillus	22.39
18.	Brucella canis	18.31
19.	Brucella abortus	15.22
20.	Brucella melitensi	5.30

Table S19 : The taxonomic profile predicted by MetaPhlAn for the genus target assembly dataset

No	Species	Coverage
1.	Yersinia pestis	200.93
2.	Xenorhabdus bovienii	122.89
3.	Sodalis glossinidius	70.47
4.	Serratia proteamaculans	61.73
5.	Proteus mirabilis	60.21
6.	Pantoea vagans	58.79
7.	Edwardsiella ictaluri	52.77
8.	Shigella flexneri	50.43
9.	Escherichia coli	42.10
10.	Photorhabdus asymbiotica	40.80
11.	Dickeya dadantii	23.03
12.	Citrobacter koseri	21.00
13.	Pectobacterium carotovora	19.22
14.	Cronobacter sakazakii	19.15
15.	Klebsiella variicola	18.31
16.	Erwinia billingiae	12.00
17.	Salmonella enterica	2.32
18.	Enterobacter cloacae	2.19

Table S20 : The taxonomic profile predicted by MetaPhlAn for the species target assembly dataset

No	Species	Coverage
1.	Bacillus anthracis	313.61
2.	Bacillus cereus	303.63
3.	Bacillus coagulans	231.67
4.	Bacillus subtilis	205.40
5.	Bacillus thuringiensis	186.69
6.	Bacillus licheniformis	123.48
7.	Bacillus clausii	117.86
8.	Bacillus amyloliquefaciens	116.37
9.	Bacillus cellulosilyticus	106.58
10.	Bacillus halodurans	48.32
11.	Geobacillus thermoglucosidasius	39.64
12.	Bacillus megaterium	36.40
13.	Bacillus atrophaeus	35.47
14.	Bacillus pseudofirmus	32.72
15.	Bacillus pumilus	32.49
16.	Geobacillus kaustophilus	30.53
17.	Bacillus selenitireducens	30.40
18.	Bacillus weihenstephanensis	8.21
19.	Anoxybacillus flavithermus	0.02

Table S21 : The identification of chimeric nodes by MetaVelvet-SL using classification models gerated from the taxonomy profile inferred by MetaPhlAn for real datasets of human gut microbial data.

Dataset	Positive (+)		Negative (-)
	Class 1	Class 2	Class 3
MH0006	6896	0	64391
MH0012	3805	14	46244
MH0047	1905	242	6413
SRS017227	58564	3427	123746
SRS018661	9466	0	37863

Table S22 : The taxonomic profile predicted by MetaPhlAn for the MH0006 dataset

No.	Species	Coverage (%)
1	Actinomyces odontolyticus	0.00083
2	Mycobacterium unclassified	0.01007
3	Bifidobacterium adolescentis	0.01411
4	Bifidobacterium longum	0.02092
5	Bifidobacterium unclassified	0.0135
6	Collinsella aerofaciens	0.19865
7	Collinsella stercoris	0.00024
8	Gordonibacter pamelaeae	0.00014
9	Slackia unclassified	0.05366
10	Bacteroides caccae	0.10729
11	Bacteroides coprocola	0.00116
12	Bacteroides coprophilus	0.00061
13	Bacteroides dorei	0.02719
14	Bacteroides eggerthii	2.01184
15	Bacteroides finegoldii	0.00215
16	Bacteroides helcogenes	0.00241
17	Bacteroides ovatus	0.00433
18	Bacteroides pectinophilus	0.00094
19	Bacteroides plebeius	0.00136
20	Bacteroides salanitronis	0.00149
21	Bacteroides stercoris	0.04667
22	Bacteroides thetaiotaomicron	0.01719
23	Bacteroides unclassified	3.75135
24	Bacteroides vulgatus	1.20529
25	Bacteroides xylanisolvens	0.11776
26	Odoribacter splanchnicus	0.60652
27	Parabacteroides distasonis	0.0021
28	Parabacteroides johnsonii	0.03426
29	Parabacteroides merdae	0.13668
30	Parabacteroides unclassified	0.20743
31	Prevotella bergensis	0.00399
32	Prevotella bivia	0.0003
33	Prevotella buccae	0.01313
34	Prevotella buccalis	0.00188
35	Prevotella copri	54.23561
36	Prevotella disiens	0.0072
37	Prevotella marshii	0.00148

38	Prevotella multiformis	0.00259
39	Prevotella oralis	0.00041
40	Prevotella oris	0.00012
41	Prevotella ruminicola	0.00104
42	Alistipes putredinis	6.92968
43	Alistipes shahii	0.57223
44	Enterococcus unclassified	0.00085
45	Lactobacillus ruminis	0.05968
46	Streptococcus parasanguinis	0.01198
47	Streptococcus salivarius	0.00946
48	Clostridium asparagiforme	0.00176
49	Clostridium bolteae	0.02018
50	Clostridium cf	0.00779
51	Clostridium hathewayi	0.00035
52	Clostridium leptum	0.00743
53	Clostridium methylpentosum	0.00039
54	Clostridium nexile	0.00091
55	Clostridium saccharolyticum	0.00023
56	Clostridium scindens	0.00103
57	Clostridium symbiosum	0.0015
58	Pseudoflavonifractor capillosus	0.0156
59	Anaerococcus unclassified	0.00083
60	Peptoniphilus unclassified	0.00339
61	Blautia hansenii	0.0003
62	Blautia hydrogenotrophica	0.00011
63	Blautia unclassified	0.46581
64	Eubacterium eligens	0.89791
65	Eubacterium hallii	0.46406
66	Eubacterium rectale	0.04896
67	Eubacterium siraeum	0.00753
68	Eubacterium ventriosum	0.12054
69	Butyrivibrio crossotus	9.96091
70	Coprococcus catus	0.15195
71	Coprococcus comes	0.56243
72	Dorea formicigenerans	0.2435
73	Dorea longicatena	0.43978
74	Marvinbryantia formatexigens	0.00071
75	Oribacterium sinus	0.00046
76	Roseburia intestinalis	0.34135
77	Roseburia inulinivorans	1.04753

78	Anaerotruncus colihominis	0.00414
79	Faecalibacterium cf	0.25602
80	Faecalibacterium prausnitzii	4.18124
81	Faecalibacterium unclassified	0.64993
82	Ruminococcus albus	0.00756
83	Ruminococcus bromii	2.69545
84	Ruminococcus gnavus	0.02897
85	Ruminococcus lactaris	0.00417
86	Ruminococcus obeum	0.21542
87	Ruminococcus torques	0.07772
88	Subdoligranulum variabile	0.07944
89	Catenibacterium mitsuokai	0.3744
90	Coprobacillus bacterium	0.00098
91	Holdemania filiformis	0.00813
92	Phascolarctobacterium unclassified	5.10549
93	Veillonella atypica	0.00013
94	Veillonella dispar	0.00064
95	Veillonella unclassified	0.00498
96	Victivallis vadensis	0.066
97	Candidatus Zinderia unclassified	0.00015
98	Bilophila wadsworthia	0.08293
99	Desulfovibrio desulfuricans	0.28546
100	Desulfovibrio piger	0.46813
101	Escherichia coli	0.0036
102	Escherichia unclassified	0.0003
103	Haemophilus parainfluenzae	0.00494
104	Akkermansia muciniphila	0.01344

Table S23 : The taxonomic profile predicted by MetaPhlAn for the MH0012 dataset

No.	Species	Coverage (%)
1	Methanobrevibacter smithii	0.79152
2	Methanobrevibacter unclassified	0.00764
3	Actinomyces odontolyticus	0.01588
4	Rothia mucilaginosa	0.00807
5	Rothia unclassified	0.00384
6	Mycobacterium unclassified	0.04043
7	Bifidobacterium adolescentis	2.87375
8	Bifidobacterium angulatum	0.00485
9	Bifidobacterium bifidum	0.06723
10	Bifidobacterium breve	0.01385
11	Bifidobacterium catenulatum	0.00179
12	Bifidobacterium dentium	0.02203
13	Bifidobacterium gallicum	0.00081
14	Bifidobacterium longum	2.36531
15	Bifidobacterium pseudocatenulatum	0.00206
16	Collinsella aerofaciens	0.77142
17	Collinsella stercoris	0.00056
18	Eggerthella lenta	0.01367
19	Gordonibacter pamelaeae	0.00217
20	Slackia unclassified	0.05594
21	Bacteroides caccae	0.01968
22	Bacteroides cellulosilyticus	8.56915
23	Bacteroides coprocola	0.00125
24	Bacteroides coprophilus	0.0007
25	Bacteroides dorei	0.00448
26	Bacteroides eggerthii	0.0083
27	Bacteroides finegoldii	0.0013
28	Bacteroides fragilis	0.00097
29	Bacteroides helcogenes	0.01655
30	Bacteroides intestinalis	0.89081
31	Bacteroides pectinophilus	0.00143
32	Bacteroides plebeius	0.00279
33	Bacteroides salanitronis	0.00145
34	Bacteroides stercoris	0.00748
35	Bacteroides thetaiotaomicron	0.20746
36	Bacteroides unclassified	14.95133
37	Bacteroides uniformis	0.00243
38	Bacteroides vulgatus	0.09977
39	Bacteroides xylanisolvens	0.00294
40	Odoribacter splanchnicus	2.95047

41	Parabacteroides distasonis	0.05494
42	Parabacteroides johnsonii	0.17964
43	Parabacteroides merdae	0.88973
44	Parabacteroides unclassified	0.43404
45	Porphyromonas asaccharolytica	0.01641
46	Porphyromonas uenonis	0.00124
47	Prevotella bivia	0.00352
48	Prevotella buccalis	0.00061
49	Prevotella copri	0.00388
50	Prevotella melaninogenica	0.01374
51	Prevotella multiformis	0.00152
52	Prevotella timonensis	0.01115
53	Alistipes putredinis	13.19879
54	Alistipes shahii	2.16517
55	Enterococcus unclassified	0.00213
56	Lactobacillus crispatus	0.00357
57	Leuconostoc mesenteroides	0.00055
58	Lactococcus lactis	0.00818
59	Streptococcus parasanguinis	0.00658
60	Streptococcus salivarius	0.01094
61	Clostridium asparagiforme	0.00585
62	Clostridium bartlettii	0.0838
63	Clostridium bolteae	0.02177
64	Clostridium cf	0.12036
65	Clostridium hathewayi	0.00127
66	Clostridium leptum	0.03763
67	Clostridium methylpentosum	0.00391
68	Clostridium nexile	0.00244
69	Clostridium scindens	0.00558
70	Clostridium symbiosum	0.00054
71	Pseudoflavonifractor capillosus	0.08103
72	Anaerococcus unclassified	0.0019
73	Peptoniphilus unclassified	0.01563
74	Blautia hansenii	0.00041
75	Blautia unclassified	0.67013
76	Eubacterium eligens	2.19713
77	Eubacterium hallii	0.44597
78	Eubacterium rectale	1.76344
79	Eubacterium siraeum	0.20007
80	Eubacterium ventriosum	0.06096
81	Anaerostipes caccae	0.00247
82	Butyrivibrio crossotus	0.01679

83	Butyrivibrio unclassified	0.17857
84	Coprococcus catus	0.15243
85	Coprococcus comes	1.06772
86	Dorea formicigenerans	0.27046
87	Dorea longicatena	1.6051
88	Marvinbryantia formatexigens	0.00336
89	Roseburia intestinalis	0.06859
90	Roseburia inulinivorans	0.06367
91	Anaerotruncus colihominis	0.0135
92	Ethanoligenens harbinense	0.00073
93	Faecalibacterium cf	1.69417
94	Faecalibacterium prausnitzii	2.93174
95	Faecalibacterium unclassified	2.4757
96	Ruminococcus albus	0.00629
97	Ruminococcus bromii	12.92122
98	Ruminococcus gnavus	0.00236
99	Ruminococcus lactaris	0.38497
100	Ruminococcus obeum	0.41698
101	Ruminococcus torques	0.30669
102	Subdoligranulum variabile	0.22113
103	Catenibacterium mitsuokai	1.6573
104	Coprobacillus bacterium	0.00655
105	Holdemania filiformis	0.25814
106	Solobacterium moorei	0.00363
107	Turicibacter unclassified	0.00244
108	Acidaminococcus fermentans	0.00131
109	Phascolarctobacterium unclassified	0.002
110	Megasphaera micronuciformis	0.00207
111	Mitsuokella multacida a	3.8412
112	Selenomonas sputigen	0.00044
113	Selenomonas unclassified	0.03973
114	Veillonella atypica	0.00834
115	Veillonella dispar	0.00265
116	Veillonella parvula	0.02108
117	Veillonella unclassified	0.02653
118	Victivallis vadensis	0.01735
119	Burkholderia unclassified	0.00991
120	Candidatus Zinderia unclassified	0.0076
121	Oxalobacter formigenes	0.08099
122	Sutterella wadsworthensis	0.99217
123	Bilophila wadsworthia	0.17234
124	Desulfovibrio desulfuricans	0.35862

125	Campylobacter hominis	0.00454
126	Succinatimonas hippei	0.62084
127	Candidatus Carsonella ruddii	0.00164
128	Citrobacter unclassified	0.0025
129	Escherichia coli	0.88315
130	Haemophilus parainfluenzae	0.15741
131	Pseudomonas unclassified	0.00319
132	Deinococcus unclassified	0.00237
133	Akkermansia muciniphila	7.87018

Table S24 : The taxonomic profile predicted by MetaPhlAn for the MH0047 dataset

No.	Species	Coverage (%)
1	Bifidobacterium adolescentis	1.42269
2	Bifidobacterium longum	0.17281
3	Collinsella aerofaciens	0.13269
4	Bacteroides caccae	1.91235
5	Bacteroides cellulosilyticus	0.04845
6	Bacteroides coprocola	0.00405
7	Bacteroides coprophilus	0.00723
8	Bacteroides dorei	0.02975
9	Bacteroides eggerthii	1.44989
10	Bacteroides finegoldii	0.00424
11	Bacteroides fragilis	0.03245
12	Bacteroides ovatus	0.12096
13	Bacteroides plebeius	0.49986
14	Bacteroides salanitronis	8.06418
15	Bacteroides stercoris	0.23805
16	Bacteroides unclassified	14.26997
17	Bacteroides uniformis	0.2608
18	Bacteroides vulgatus	2.92952
19	Bacteroides xylanisolvens	0.34885
20	Odoribacter splanchnicus	1.35725
21	Parabacteroides distasonis	0.24968
22	Parabacteroides johnsonii	0.05548
23	Parabacteroides merdae	0.65483
24	Parabacteroides unclassified	0.29175
25	Alistipes putredinis	13.10953
26	Alistipes shahii	1.44786
27	Streptococcus mutans	0.01654
28	Streptococcus parasanguinis	0.05671
29	Streptococcus salivarius	0.07287
30	Clostridium asparagiforme	0.02126
31	Clostridium bartlettii	0.61844
32	Clostridium cf	0.22457
33	Clostridium leptum	0.11801
34	Clostridium nexile	11.45715
35	Pseudoflavonifractor capillosus	0.35284
36	Blautia unclassified	0.681
37	Eubacterium hallii	0.32805

38	Eubacterium rectale	2.51839
39	Eubacterium siraeum	4.6827
40	Butyrivibrio unclassified	0.00916
41	Coprococcus catus	0.05056
42	Coprococcus comes	0.23222
43	Dorea formicigenerans	0.08341
44	Dorea unclassified	0.01655
45	Roseburia intestinalis	2.32058
46	Roseburia inulinivorans	0.04956
47	Anaerotruncus colihominis	0.01297
48	Faecalibacterium cf	0.88555
49	Faecalibacterium prausnitzii	0.83598
50	Faecalibacterium unclassified	0.28332
51	Ruminococcus bromii	5.96385
52	Ruminococcus gnavus	7.67142
53	Ruminococcus obeum	0.34787
54	Ruminococcus torques	0.67722
55	Subdoligranulum variabile	0.0862
56	Coprobacillus bacterium	1.7778
57	Holdemania filiformis	0.12442
58	Phascolarctobacterium unclassified	6.40517
59	Veillonella atypica	0.04381
60	Veillonella dispar	0.04371
61	Veillonella unclassified	0.17813
62	Victivallis vadensis	0.03391
63	Bilophila wadsworthia	0.49395
64	Desulfovibrio desulfuricans	0.33353
65	Desulfovibrio piger	0.35435
66	Escherichia coli	0.09425
67	Prevotella buccae	0.005

Table S25 : The taxonomic profile predicted by MetaPhlAn for the SRS017227 dataset

No.	Species	Coverage (%)
1	Lautropia mirabilis	14.70585
2	Corynebacterium matruchotii	12.87123
3	Neisseria elongata	7.89805
4	Capnocytophaga sputigena	7.19035
5	Capnocytophaga gingivalis	4.51523
6	Neisseria flavescens	4.16504
7	Aggregatibacter segnis	4.12745
8	Aggregatibacter aphrophilus	3.97433
9	Cardiobacterium hominis	3.34601
10	Haemophilus parainfluenzae	2.7273
11	Neisseria sicca	2.68083
12	Capnocytophaga ochracea	1.98778
13	Neisseria mucosa	1.98358
14	Fusobacterium nucleatum	1.74169
15	Campylobacter showae	1.62199
16	Kingella denitrificans	1.57152
17	Campylobacter gracilis	1.45685
18	Veillonella parvula	1.44265
19	Eikenella corrodens	1.34903
20	Treponema vincentii	1.31729
21	Prevotella oris	1.19069
22	Prevotella tannerae	1.07383
23	Leptotrichia unclassified	0.88596
24	Selenomonas noxia	0.85609
25	Selenomonas sputigena	0.85004
26	Porphyromonas endodontalis	0.77862
27	Veillonella unclassified	0.75352
28	Leptotrichia hofstadii	0.65234
29	Actinomyces viscosus	0.62298
30	Leptotrichia buccalis	0.61686
31	Streptococcus sanguinis	0.58799
32	Catonella morbi	0.58137
33	Streptococcus mitis	0.5789
34	Dialister invisus	0.53868
35	Granulicatella adiacens	0.41977
36	Gemella moribillum	0.36168
37	Actinomyces oris	0.31598
38	Selenomonas artemidis	0.31059
39	Prevotella marshii	0.30738
40	Campylobacter rectus	0.29366

41	Neisseria subflava	0.17734
42	Eubacterium saburreum	0.14372
43	Rothia dentocariosa	0.14158
44	Streptococcus oralis	0.13944
45	Streptococcus cristatus	0.13615
46	Campylobacter concisus	0.12499
47	Prevotella veroralis	0.10961
48	Actinomyces odontolyticus	0.09761
49	Granulicatella elegans	0.0863
50	Leptotrichia goodfellowii	0.08582
51	Atopobium rimae	0.07269
52	Haemophilus influenzae	0.06659
53	Neisseria meningitidis	0.0606
54	Fusobacterium periodonticum	0.05745
55	Treponema denticola	0.04746
56	Peptoniphilus unclassified	0.04201
57	Gemella haemolysans	0.03466
58	Streptococcus infantis	0.02494
59	Megasphaera micronuciformis	0.02045
60	Selenomonas flueggei	0.01722
61	Rothia mucilaginosa	0.014
62	Neisseria cinerea	0.01315
63	Atopobium parvulum	0.00925
64	Bacteroides vulgatus	0.00874
65	Bacteroides unclassified	0.00861
66	Neisseria gonorrhoeae	0.00859
67	Oribacterium sinus	0.00738
68	Streptococcus australis	0.00651
69	Streptococcus gordonii	0.00583
70	Alistipes putredinis	0.00476
71	Streptococcus salivarius	0.00475
72	Solobacterium moorei	0.00437
73	Neisseria polysaccharea	0.00389
74	Veillonella dispar	0.00138
75	Prevotella salivae	0.00101
76	Prevotella copri	0.00099
77	Bacteroides caccae	0.00084
78	Propionibacterium acnes	0.00081
79	Actinomyces urogenitalis	0.00053
80	Veillonella atypica	0.00049
81	Slackia exigua	0.00047
82	Streptococcus parasanguinis	0.00047

83	Parabacteroides merdae	0.00036
84	Bacteroides ovatus	0.00035
85	Streptococcus peroris	0.00019
86	Aggregatibacter actinomycetemcomitans	9e-05

Table S26 : The taxonomic profile predicted by MetaPhlAn for the SRS018661 dataset

No.	Species	Coverage (%)
1	Streptococcus mitis	31.48217
2	Haemophilus parainfluenzae	24.47842
3	Streptococcus sanguinis	4.61514
4	Neisseria mucosa	3.59988
5	Abiotrophia defectiva	3.54552
6	Streptococcus infantis	3.43131
7	Gemella haemolysans	2.92807
8	Veillonella parvula	2.21204
9	Neisseria sicca	2.02929
10	Rothia dentocariosa	1.98869
11	Veillonella unclassified	1.90907
12	Streptococcus oralis	1.72239
13	Prevotella melaninogenica	1.58671
14	Rothia mucilaginosa	1.4698
15	Neisseria flavescens	1.33677
16	Haemophilus influenzae	1.29628
17	Streptococcus gordonii	1.15805
18	Actinomyces odontolyticus	0.74638
19	Gemella moribillum	0.70625
20	Actinomyces oris	0.6702
21	Fusobacterium nucleatum	0.50795
22	Granulicatella elegans	0.42218
23	Streptococcus cristatus	0.40701
24	Actinomyces viscosus	0.37065
25	Streptococcus australis	0.33662
26	Granulicatella adiacens	0.33506
27	Veillonella atypica	0.31405
28	Campylobacter concisus	0.2806
29	Capnocytophaga sputigena	0.26398
30	Streptococcus parasanguinis	0.23954
31	Oribacterium sinus	0.23174
32	Veillonella dispar	0.21931
33	Acidovorax unclassified	0.21516
34	Aggregatibacter segnis	0.21107
35	Streptococcus peroris	0.1985
36	Capnocytophaga ochracea	0.19732
37	Prevotella tannerae	0.1707
38	Capnocytophaga gingivalis	0.16015
39	Megasphaera micronuciformis	0.12368
40	Neisseria elongata	0.11335

41	Streptococcus salivarius	0.10814
42	Kingella oralis	0.10379
43	Atopobium parvulum	0.09092
44	Prevotella salivae	0.08996
45	Solobacterium moorei	0.08367
46	Corynebacterium matruchotii	0.07413
47	Acinetobacter junii	0.07112
48	Neisseria subflava	0.06826
49	Eikenella corrodens	0.05846
50	Leptotrichia buccalis	0.05378
51	Leptotrichia unclassified	0.05241
52	Prevotella oris	0.0498
53	Campylobacter showae	0.04522
54	Parvimonas micros	0.03866
55	Leptotrichia hofstadii	0.03665
56	Eubacterium saburreum	0.0359
57	Cardiobacterium hominis	0.0338
58	Streptococcus pneumoniae	0.02831
59	Peptostreptococcus stomatis	0.02787
60	Prevotella veroralis	0.02707
61	Lautropia mirabilis	0.02552
62	Aggregatibacter aphrophilus	0.02469
63	Dialister invisus	0.02373
64	Propionibacterium acnes	0.02113
65	Fusobacterium periodonticum	0.02103
66	Prevotella multiformis	0.01983
67	Selenomonas unclassified	0.01735
68	Campylobacter rectus	0.01589
69	Atopobium rimae	0.0117
70	Selenomonas noxia	0.01148
71	Streptococcus thermophilus	0.01115
72	Treponema vincentii	0.00866
73	Treponema denticola	0.00837
74	Escherichia unclassified	0.00549
75	Campylobacter gracilis	0.00524
76	Peptoniphilus unclassified	0.00468
77	Porphyromonas endodontalis	0.00441
78	Eubacterium saphenum	0.00436
79	Bifidobacterium dentium	0.00397
80	Streptococcus anginosus	0.00363
81	Selenomonas sputigena	0.0029
82	Selenomonas artemidis	0.00285

83	Propionibacterium unclassified	0.00212
84	Anaerococcus unclassified	0.00201
85	Neisseria gonorrhoeae	0.002
86	Catonella morbi	0.00198
87	Escherichia coli	0.00135
88	Alicycliphilus denitrificans	0.00102
89	Selenomonas flueggei	0.00095

Table S27 : The taxonomic profile predicted from the assembly results of MetaVelvet-SL using BLAST for the MH0006 dataset.

No	Species
1	Actinomyces odontolyticus
2	Akkermansia muciniphila
3	Alistipes putredinis
4	Alistipes shahii
5	Anaerococcus unclassified
6	Anaerotruncus colihominis
7	Bacteroides caccae
8	Bacteroides coprocola
9	Bacteroides coprophilus
10	Bacteroides dorei
11	Bacteroides eggerthii
12	Bacteroides finegoldii
13	Bacteroides helcogenes
14	Bacteroides ovatus
15	Bacteroides pectinophilus
16	Bacteroides plebeius
17	Bacteroides salanitronis
18	Bacteroides stercoris
19	Bacteroides thetaiotaomicron
20	Bacteroides unclassified
21	Bacteroides vulgatus
22	Bacteroides xylanisolvens
23	Bifidobacterium adolescentis
24	Bifidobacterium longum
25	Bifidobacterium unclassified
26	Bilophila wadsworthia
27	Blautia hansenii
28	Blautia hydrogenotrophica
29	Blautia unclassified
30	Butyrivibrio crossotus
31	Clostridium asparagiforme
32	Clostridium bolteae
33	Clostridium cf
34	Clostridium hathewayi
35	Clostridium leptum
36	Clostridium methylpentosum
37	Clostridium nexile
38	Clostridium saccharolyticum
39	Clostridium scindens
40	Clostridium symbiosum
41	Collinsella aerofaciens
42	Collinsella stercoris
43	Coprococcus catus
44	Coprococcus comes
45	Desulfovibrio desulfuricans
46	Desulfovibrio piger
47	Dorea formicigenerans
48	Dorea longicatena
49	Enterococcus unclassified
50	Escherichia coli
51	Escherichia unclassified
52	Eubacterium eligens
53	Eubacterium hallii
54	Eubacterium rectale
55	Eubacterium siraeum
56	Eubacterium ventriosum
57	Faecalibacterium cf
58	Faecalibacterium prausnitzii
59	Faecalibacterium unclassified
60	Gordonibacter pamelaeae
61	Haemophilus parainfluenzae
62	Holdemania filiformis
63	Lactobacillus ruminis
64	Mycobacterium unclassified
65	Odoribacter splanchnicus
66	Oribacterium sinus
67	Parabacteroides distasonis
68	Parabacteroides johnsonii
69	Parabacteroides merdae
70	Parabacteroides unclassified
71	Peptoniphilus unclassified
72	Phascolarctobacterium unclassified
73	Prevotella bergensis
74	Prevotella bivia
75	Prevotella buccae
76	Prevotella buccalis
77	Prevotella copri
78	Prevotella disiens
79	Prevotella marshii
80	Prevotella multiformis
81	Prevotella oralis
82	Prevotella oris
83	Prevotella ruminicola
84	Pseudoflavonifractor capillosus
85	Roseburia intestinalis
86	Roseburia inulinivorans
87	Ruminococcus albus
88	Ruminococcus bromii
89	Ruminococcus gnavus
90	Ruminococcus lactaris
91	Ruminococcus obeum
92	Ruminococcus torques
93	Slackia unclassified
94	Streptococcus parasanguinis
95	Streptococcus salivarius
96	Subdoligranulum variabile
97	Veillonella dispar
98	Veillonella unclassified
99	Victivallis vadensis
100	'Nostoc azollae'
101	[Bacillus] schlegelii
102	[Clostridium] aerotolerans
103	[Clostridium] alkalicellulosi
104	[Clostridium] aminophilum
105	[Clostridium] aminovalericum
106	[Clostridium] bifermentans
107	[Clostridium] cellobioparum
108	[Clostridium] cellulosi
109	[Clostridium] clostridioforme
110	[Clostridium] glycyrrhizinilyticum

111	[Clostridium] indolis
112	[Clostridium] josui
113	[Clostridium] lactatifermentans
114	[Clostridium] mangenotii
115	[Clostridium] methoxybenzovorans
116	[Clostridium] neopropionicum
117	[Clostridium] papyrosolvens
118	[Clostridium] populeti
119	[Clostridium] propionicum
120	[Clostridium] saccharogumia
121	[Clostridium] sordellii
122	[Clostridium] sticklandii,
123	[Clostridium] termitidis
124	[Clostridium] thermosuccinogenes
125	[Clostridium] viride
126	[Eubacterium] cellulosolvens
127	[Pseudomonas] geniculata
128	[Scytonema hofmanni]
129	Abiotrophia defectiva
130	Acanthamoeba castellanii
131	Acanthisitta chloris
132	Acaricomes phytoseiuli
133	Acaryochloris sp.
134	Acetanaerobacterium elongatum
135	Acetatifactor muris
136	Acetivibrio cellulolyticus
137	Acetobacter aceti
138	Acetobacter nitrogenifigens
139	Acetobacter pasteurianus
140	Acetobacter sp.
141	Acetobacteraceae bacterium
142	Acetobacterium dehalogenans
143	Acetobacterium woodii
144	Acetohalobium arabaticum
145	Acetomicrobium faecale
146	Acholeplasma axanthum
147	Acholeplasma equifetale
148	Acholeplasma granularum
149	Acholeplasma laidlawii
150	Acholeplasma modicum
151	Acholeplasma multilocale
152	Acholeplasma sp.
153	Achromobacter piechaudii
154	Achromobacter xylosoxidans
155	Acidaminococcus fermentans
156	Acidaminococcus intestini
157	Acidaminococcus sp.
158	Acidimicrobidae bacterium
159	Acidiphilium angustum
160	Acidiphilium cryptum
161	Acidiphilium multivorum
162	Acidiphilium sp.
163	Acidithiobacillus caldus
164	Acidithiobacillus ferrivorans
165	Acidithiobacillus ferrooxidans
166	Acidithiobacillus thiooxidans
167	Acidobacteria bacterium
168	Acidobacteriaceae bacterium
169	Acidobacterium capsulatum
170	Acidobacterium sp.
171	Acidocella facilis
172	Acidocella sp.
173	Acidothermus cellulolyticus
174	Acidovorax avenae
175	Acidovorax citrulli
176	Acidovorax ebreus
177	Acidovorax oryzae
178	Acidovorax radicis
179	Acidovorax sp.
180	Acinetobacter baumannii
181	Acinetobacter bereziniae
182	Acinetobacter gerneri
183	Acinetobacter gyllenbergii
184	Acinetobacter haemolyticus
185	Acinetobacter johnsonii
186	Acinetobacter junii
187	Acinetobacter lwoffii
188	Acinetobacter pittii
189	Acinetobacter radioresistens
190	Acinetobacter sp.
191	Acinetobacter tandoii
192	Acinetobacter ursingii
193	Actinoalloteichus cyanogriseus
194	Actinobacillus capsulatus
195	Actinobacillus pleuropneumoniae
196	Actinobacillus succinogenes
197	Actinobacterium LLX17
198	Actinobacterium SCGC
199	Actinobaculum massiliae
200	Actinobaculum schaalii
201	Actinobaculum sp.
202	Actinocatenispora sera
203	Actinokineospora enzanensis
204	Actinokineospora inagensis
205	Actinomadura atramentaria
206	Actinomadura flavalba
207	Actinomadura madurae
208	Actinomadura oligospora
209	Actinomadura rifamycini
210	Actinomyces cardiffensis
211	Actinomyces dentalis
212	Actinomyces europaeus
213	Actinomyces georgiae
214	Actinomyces gerencseriae
215	Actinomyces graevenitzii
216	Actinomyces johnsonii
217	Actinomyces massiliensis
218	Actinomyces neuii
219	Actinomyces oris
220	Actinomyces slackii
221	Actinomyces sp.
222	Actinomyces timonensis
223	Actinomyces turicensis
224	Actinomyces urogenitalis
225	Actinomyces viscosus
226	Actinomycetospora chiangmaiensis

227	Actinoplanes friuliensis
228	Actinoplanes globisporus
229	Actinoplanes missouriensis
230	Actinoplanes sp.
231	Actinoplanes subtropicus
232	Actinopolymorpha alba
233	Actinopolyspora mortivallis
234	Actinosynnema mirum
235	Acyrthosiphon pisum
236	Adlercreutzia equolifaciens
237	Advenella kashmirensis
238	Aedes aegypti
239	Aequorivita capsosiphonis
240	Aequorivita sublithincola
241	Aerococcus urinae
242	Aeromicrobium marinum
243	Aeromonas caviae
244	Aeromonas enteropelogenes
245	Aeromonas hydrophila
246	Aeromonas salmonicida
247	Aeromonas veronii
248	Afifella pfennigii
249	Afipia broomeae
250	Afipia clevelandensis
251	Afipia sp.
252	Aggregatibacter actinomycetemcomitans
253	Aggregatibacter aphrophilus
254	Agrobacterium fabrum
255	Agrobacterium radiobacter
256	Agrobacterium sp.
257	Agrobacterium tumefaciens
258	Agrobacterium vitis
259	Agrococcus lahaulensis
260	Agrococcus pavilionensis
261	Agromonas oligotrophica
262	Agromyces italicus
263	Agromyces subbeticus
264	Ahrensia kielensis
265	Ailuropoda melanoleuca
266	Ajellomyces capsulatus
267	Ajellomyces dermatitidis
268	Akkermansia sp.
269	Alcaligenes faecalis
270	Alcaligenes sp.
271	Alcanivorax borkumensis
272	Alcanivorax dieselolei
273	Alcanivorax sp.
274	Algicola sagamiensis
275	Algoriphagus mannitolivorans
276	Algoriphagus marincola
277	Algoriphagus sp.
278	Algoriphagus terrigena
279	Aliagarivorans marinus
280	Alicycliphilus denitrificans
281	Alicyclobacillus acidocaldarius
282	Alicyclobacillus acidoterrestris
283	Alicyclobacillus contaminans
284	Alicyclobacillus herbarius

285	Alicyclobacillus macrosporangiidus
286	Alicyclobacillus pohliae
287	Alicyclobacillus pomorum
288	Aliihoeflea sp.
289	Aliivibrio fischeri
290	Aliivibrio salmonicida
291	Alistipes finegoldii
292	Alistipes indistinctus
293	Alistipes onderdonkii
294	Alistipes senegalensis
295	Alistipes sp.
296	Alistipes timonensis
297	Alkalibacillus haloalkaliphilus
298	Alkaliflexus imshenetskii
299	Alkalilimnicola ehrlichii
300	Alkaliphilus metalliredigens
301	Alkaliphilus oremlandii
302	Alkaliphilus transvaalensis
303	Alligator mississippiensis
304	Alligator sinensis
305	Allobaculum stercoricanis
306	Allochromatium vinosum
307	Allofustis seminis
308	Alloiococcus otitis
309	Allokutzneria albata
310	Alloprevotella rava
311	Alloscardovia omnicolens
312	Alpha proteobacterium
313	Amborella trichopoda
314	Aminicenantes bacterium
315	Aminiphilus circumscriptus
316	Aminobacter sp.
317	Aminobacterium colombiense
318	Aminomonas paucivorans
319	Ammonifex degensii
320	Amorphus coralli
321	Amphibacillus jilinensis
322	Amphibacillus xylanus
323	Amphimedon queenslandica
324	Amycolatopsis alba
325	Amycolatopsis balhimycina
326	Amycolatopsis benzoatilytica
327	Amycolatopsis mediterranei
328	Amycolatopsis methanolica
329	Amycolatopsis nigrescens
330	Amycolatopsis orientalis
331	Amycolatopsis sp.
332	Amycolatopsis taiwanensis
333	Amycolatopsis thermoflava
334	Anabaena sp.
335	Anabaena variabilis
336	Anaeroarcus burkinensis
337	Anaerobaculum hydrogeniformans
338	Anaerobaculum mobile
339	Anaerobiospirillum succiniciproducens
340	Anaerofilum agile
341	Anaerofilum pentosovorans
342	Anaerofustis stercorihominis

343	Anaeroglobus geminatus
344	Anaerolinea thermophila
345	Anaeromusa acidaminophila
346	Anaeromyxobacter dehalogenans
347	Anaeromyxobacter sp.
348	Anaerophaga thermohalophila
349	Anaerosporobacter mobilis
350	Anaerostipes caccae
351	Anaerostipes hadrus
352	Anaerostipes sp.
353	Anaerotruncus sp.
354	Anaerovibrio lipolyticus
355	Anaerovibrio sp.
356	Anaerovorax odorimutans
357	Anaplasma phagocytophilum
358	Anas platyrhynchos
359	Aneurinibacillus aneurinilyticus
360	Aneurinibacillus terranovensis
361	Anolis carolinensis
362	Anopheles gambiae
363	Anoxybacillus flavithermus
364	Anoxybacillus kamchatkensis
365	Anoxybacillus tepidamans
366	Aphanizomenon flos-aquae
367	Apis dorsata
368	Apis florea
369	Apis mellifera
370	Aplysia californica
371	Aptenodytes forsteri
372	Aquifex aeolicus
373	Aquimarina agarilytica
374	Aquimarina macrocephali
375	Aquimarina muelleri
376	Arabidopsis lyrata
377	Arabidopsis thaliana
378	Arcanobacterium haemolyticum
379	Arcobacter butzleri
380	Arcobacter cibarius
381	Arcobacter nitrofigilis
382	Arcobacter sp.
383	Arenibacter certesii
384	Arenibacter latericius
385	Arenimonas composti
386	Arenimonas oryziterrae
387	Arenitalea lutea
388	Arhodomonas aquaeolei
389	Aromatoleum aromaticum
390	Arsenicicoccus bolidensis
391	Arthrobacter arilaitensis
392	Arthrobacter castelli
393	Arthrobacter chlorophenolicus
394	Arthrobacter phenanthrenivorans
395	Arthrobacter sanguinis
396	Arthrobacter sp.
397	Arthroderma gypseum
398	Arthrospira platensis
399	Asaia prunellae
400	Asaia sp.

401	Aspergillus clavatus
402	Aspergillus nidulans
403	Aspergillus niger
404	Aspergillus oryzae
405	Aspergillus terreus
406	Asticcacaulis benevestitus
407	Asticcacaulis biprosthecum
408	Asticcacaulis sp.
409	Astyanax mexicanus
410	Atopobacter phocae
411	Atopobium fossor
412	Atopobium minutum
413	Atopobium parvulum
414	Atopobium sp.
415	Atopobium vaginae
416	Atopococcus tabaci
417	Atribacteria bacterium
418	Aurantimonas coralicida
419	Aurantimonas manganoxydans
420	Aureimonas ureilytica
421	Aureococcus anophagefferens
422	Auricularia delicata
423	Azoarcus sp.
424	Azoarcus toluclasticus
425	Azohydromonas australica
426	Azonexus hydrophilus
427	Azorhizobium caulinodans
428	Azorhizobium doebereinerae
429	Azospirillum brasilense
430	Azospirillum halopraeferens
431	Azospirillum lipoferum
432	Azospirillum sp.
433	Azotobacter vinelandii
434	Azovibrio restrictus
435	Babesia bovis
436	Bacillus acidiproducens
437	Bacillus aidingensis
438	Bacillus amyloliquefaciens
439	Bacillus anthracis
440	Bacillus atrophaeus
441	Bacillus aurantiacus
442	Bacillus bogoriensis
443	Bacillus cellulosilyticus
444	Bacillus cereus
445	Bacillus chagannorensis
446	Bacillus coagulans
447	Bacillus coahuilensis
448	Bacillus cytotoxicus
449	Bacillus endophyticus
450	Bacillus flexus
451	Bacillus fordii
452	Bacillus gelatini
453	Bacillus ginsengihumi
454	Bacillus halodurans
455	Bacillus kribbensis
456	Bacillus licheniformis
457	Bacillus mannanilyticus
458	Bacillus marmarensis

459	Bacillus massilioanorexius
460	Bacillus massiliosenegalensis
461	Bacillus megaterium
462	Bacillus mojavensis
463	Bacillus oceanisediminis
464	Bacillus panaciterrae
465	Bacillus phage
466	Bacillus pseudofirmus
467	Bacillus pseudomycoides
468	Bacillus pumilus
469	Bacillus selenitireducens
470	Bacillus siamensis
471	Bacillus smithii
472	Bacillus sonorensis
473	Bacillus sp.
474	Bacillus subtilis
475	Bacillus tequilensis
476	Bacillus thuringiensis
477	Bacillus timonensis
478	Bacillus toyonensis
479	Bacillus vallismortis
480	Bacillus weihenstephanensis
481	Bacteriovorax marinus
482	Bacterium JKG1
483	Bacteroidales bacterium
484	Bacteroidetes oral
485	Balaenoptera acutorostrata
486	Balneatrix alpica
487	Balneimonas flocculans
488	Balneola vulgaris
489	Barnesiella intestinihominis
490	Bartonella birtlesii
491	Bartonella elizabethae
492	Bartonella grahamii
493	Bartonella henselae
494	Bartonella quintana
495	Baudoinia compniacensis
496	Bavariicoccus seileri
497	Bdellovibrio bacteriovorus
498	Beauveria bassiana
499	Belliella baltica
500	Beta proteobacterium
501	Beutenbergia cavernae
502	Bibersteinia trehalosi
503	Bilophila sp.
504	Bipolaris oryzae
505	Bipolaris zeicola
506	Blastocatella fastidiosa
507	Blastococcus saxobsidens
508	Blastococcus sp.
509	Blastomonas sp.
510	Blastopirellula marina
511	Blattabacterium sp.
512	Bombus impatiens
513	Bombus terrestris
514	Bombyx mori
515	Bordetella avium
516	Bordetella bronchiseptica
517	Bordetella hinzii
518	Bordetella petrii
519	Bordetella sp.
520	Bordetella trematum
521	Borrelia miyamotoi
522	Bos mutus
523	Bos taurus
524	Bosea sp.
525	Brachybacterium faecium
526	Brachybacterium muris
527	Brachybacterium paraconglomeratum
528	Brachybacterium squillarum
529	Brachymonas chironomi
530	Brachypodium distachyon
531	Brachyspira alvinipulli
532	Brachyspira hyodysenteriae
533	Brachyspira innocens
534	Brachyspira intermedia
535	Brachyspira murdochii
536	Brachyspira pilosicoli
537	Brachyspira sp.
538	Bradyrhizobiaceae bacterium
539	Bradyrhizobium elkanii
540	Bradyrhizobium genosp.
541	Bradyrhizobium japonicum
542	Bradyrhizobium sp.
543	Branchiostoma floridae
544	Brassica rapa
545	BRC1 bacterium
546	Brenneria sp.
547	Brevibacillus agri
548	Brevibacillus borstelensis
549	Brevibacillus brevis
550	Brevibacillus laterosporus
551	Brevibacillus massiliensis,
552	Brevibacillus panacihumi
553	Brevibacillus thermoruber
554	Brevibacterium album
555	Brevibacterium linens
556	Brevibacterium massiliense
557	Brevibacterium senegalense,
558	Brevibacterium sp.
559	Brevundimonas aveniformis
560	Brevundimonas bacteroides
561	Brevundimonas diminuta
562	Brevundimonas naejangsanensis
563	Brevundimonas sp.
564	Brevundimonas subvibrioides
565	Brucella abortus
566	Brucella melitensis
567	Brucella sp.
568	Bryobacter aggregatus
569	Bubalus bubalis
570	Buchnera aphidicola
571	Burkholderia acidipaludis
572	Burkholderia andropogonis
573	Burkholderia bannensis
574	Burkholderia bryophila

575	Burkholderia caledonica
576	Burkholderia cenocepacia
577	Burkholderia cepacia
578	Burkholderia dilworthii
579	Burkholderia dolosa
580	Burkholderia ferrariae
581	Burkholderia fungorum
582	Burkholderia gladioli
583	Burkholderia glumae
584	Burkholderia kururiensis
585	Burkholderia mallei
586	Burkholderia mimosarum
587	Burkholderia multivorans
588	Burkholderia nodosa
589	Burkholderia oxyphila
590	Burkholderia phymatum
591	Burkholderia phytofirmans
592	Burkholderia pseudomallei
593	Burkholderia pyrrocinia
594	Burkholderia rhizoxinica
595	Burkholderia sordidicola
596	Burkholderia sp.
597	Burkholderia sprentiae
598	Burkholderia thailandensis
599	Burkholderia ubonensis
600	Burkholderia vietnamiensis
601	Burkholderia xenovorans
602	Burkholderiales bacterium
603	Butyrate-producing bacterium
604	Butyricicoccus pullicaecorum
605	Butyricimonas synergistica
606	Butyricimonas virosa
607	Butyrivibrio fibrisolvens
608	Butyrivibrio hungatei
609	Butyrivibrio proteoclasticus
610	Butyrivibrio sp.
611	Caenorhabditis briggsae
612	Caenorhabditis elegans
613	Caenorhabditis remanei
614	Caldanaerobius polysaccharolyticus
615	Caldibacillus debilis
616	Caldicellulosiruptor bescii
617	Caldicellulosiruptor hydrothermalis
618	Caldicellulosiruptor kronotskyensis
619	Caldicellulosiruptor obsidiansis
620	Caldicellulosiruptor owensensis
621	Caldicellulosiruptor saccharolyticus
622	Caldicoprobacter guelmensis
623	Caldicoprobacter oshimai
624	Caldilinea aerophila
625	Caldimonas manganoxidans
626	Caldisphaera lagunensis
627	Calditerrivibrio nitroreducens
628	Caldithrix abyssi
629	Callicebus moloch
630	Callithrix jacchus
631	Callorhinchus milii
632	Caloramator fervidus

633	Caloramator sp.
634	Calothrix sp.
635	Calypte anna
636	Camelus ferus
637	Campylobacter coli
638	Campylobacter concisus
639	Campylobacter cuniculorum
640	Campylobacter curvus
641	Campylobacter fetus
642	Campylobacter jejuni
643	Campylobacter sp.
644	Campylobacter ureolyticus
645	Campylobacterales bacterium
646	Candida albicans
647	Candida maltosa
648	Candida orthopsilosis
649	Candida tropicalis
650	Candidate division
651	Candidatus Accumulibacter
652	Candidatus Alistipes
653	Candidatus Arthromitus
654	Candidatus Azobacteroides
655	Candidatus Blochmannia
656	Candidatus Burkholderia
657	Candidatus Carsonella
658	Candidatus Chloracidobacterium
659	Candidatus Cloacamonas
660	Candidatus Desulforudis
661	Candidatus Hydrogenedens
662	Candidatus Koribacter
663	Candidatus Latescibacter
664	Candidatus Methanomethylophilus
665	Candidatus Microthrix
666	Candidatus Nitrososphaera
667	Candidatus Nitrospira
668	Candidatus Pelagibacter
669	Candidatus Phytoplasma
670	Candidatus Poribacteria
671	Candidatus Portiera
672	Candidatus Regiella
673	Candidatus Schmidhempelia
674	Candidatus Solibacter
675	Candidatus Stoquefichus
676	Candidatus Sulcia
677	Candidatus Synechococcus
678	Canis lupus
679	Capnocytophaga canimorsus
680	Capnocytophaga cynodegmi
681	Capnocytophaga granulosa
682	Capnocytophaga ochracea
683	Capnocytophaga sp.
684	Capra hircus
685	Capsaspora owczarzaki
686	Capsella rubella
687	Carboxydibrachium pacificum
688	Carboxydothermus hydrogenoformans
689	Cardiobacterium hominis
690	Cardiobacterium valvarum

691	Carnimonas nigrificans
692	Carnobacterium jeotgali
693	Carnobacterium maltaromaticum
694	Carnobacterium sp.
695	Catabacter hongkongensis
696	Catellibacterium nectariphilum
697	Catelliglobosispora koreensis
698	Catenibacterium sp.
699	Catenovulum agarivorans
700	Catenulispora acidiphila
701	Catonella morbi
702	Caulobacter crescentus
703	Caulobacter segnis
704	Caulobacter sp.
705	Caulobacteraceae bacterium
706	Cavia porcellus
707	Cedecea davisae
708	Cellulomonas fimi
709	Cellulomonas flavigena
710	Cellulomonas massiliensis
711	Cellulomonas sp.
712	Cellulophaga algicola
713	Cellulophaga baltica
714	Cellulosimicrobium cellulans
715	Cellvibrio gilvus
716	Cellvibrio japonicus
717	Cellvibrio sp.
718	Centipeda periodontii
719	Cephalotaxus oliveri
720	Ceratitis capitata
721	Ceratotherium simum
722	Cetobacterium somerae
723	Chaetomium globosum
724	Chaetomium thermophilum
725	Chamaesiphon minutus
726	Chelativorans sp.
727	Chelatococcus sp.
728	Chelonia mydas
729	Chinchilla lanigera
730	Chitinibacter tainanensis
731	Chitinilyticum aquatile
732	Chitinilyticum litopenaei
733	Chitinimonas koreensis
734	Chitiniphilus shinanonensis
735	Chitinophaga pinensis
736	Chitinophaga sp.
737	Chlamydia psittaci
738	Chlamydomonas reinhardtii
739	Chlamydophila pneumoniae
740	Chlorella variabilis
741	Chlorobaculum parvum
742	Chlorobium chlorochromatii
743	Chlorobium limicola
744	Chlorobium luteolum
745	Chlorobium phaeobacteroides
746	Chlorobium tepidum
747	Chlorocebus aethiops
748	Chlorocebus sabaeus

749	Chloroflexi bacterium
750	Chloroflexus aggregans
751	Chloroflexus sp.
752	Chloroherpeton thalassium
753	Christensenella minuta
754	Chromobacterium violaceum
755	Chromohalobacter salexigens
756	Chrysemys picta
757	Chryseobacterium daeguense
758	Chryseobacterium gleum
759	Chryseobacterium gregarium
760	Chryseobacterium hispalense
761	Chryseobacterium sp.
762	Chryseobacterium taeanense
763	Chrysiogenes arsenatis
764	Chrysochloris asiatica
765	Chthonomonas calidirosea
766	Cicer arietinum
767	Ciona intestinalis
768	Citreicella sp.
769	Citricoccus sp.
770	Citrobacter freundii
771	Citrobacter koseri
772	Citrobacter rodentium
773	Citrobacter sp.
774	Citrobacter youngae
775	Citromicrobium bathyomarinum
776	Citromicrobium sp.
777	Citrus clementina
778	Cladophialophora carrionii
779	Clavibacter michiganensis
780	Clavispora lusitaniae
781	Cloacimonetes bacterium
782	Clostridiales bacterium
783	Clostridiales genomosp.
784	Clostridiisalibacter paucivorans
785	Clostridium acetobutylicum
786	Clostridium acidurici
787	Clostridium akagii
788	Clostridium algidicarnis
789	Clostridium arbusti
790	Clostridium autoethanogenum
791	Clostridium bartlettii
792	Clostridium beijerinckii
793	Clostridium botulinum
794	Clostridium butyricum
795	Clostridium cadaveris
796	Clostridium carboxidivorans
797	Clostridium celatum
798	Clostridium cellobioparum
799	Clostridium cellulolyticum
800	Clostridium cellulovorans
801	Clostridium citroniae
802	Clostridium clariflavum
803	Clostridium clostridioforme
804	Clostridium colicanis
805	Clostridium difficile
806	Clostridium glycolicum

807	Clostridium hiranonis	865	Coprobacter fastidiosus
808	Clostridium hydrogeniformans	866	Coprococcus eutactus
809	Clostridium hylemonae	867	Coprococcus sp.
810	Clostridium innocuum	868	Coprothermobacter proteolyticus
811	Clostridium intestinale	869	Coraliomargarita akajimensis
812	Clostridium kluyveri	870	Coraliomargarita sp.
813	Clostridium lentocellum	871	Corallococcus coralloides
814	Clostridium limosum	872	Corallococcus sp.
815	Clostridium ljungdahlii	873	Cordyceps militaris
816	Clostridium lundense	874	Coriobacteriaceae bacterium
817	Clostridium novyi	875	Coriobacterium glomerans
818	Clostridium paraputrificum	876	Corvus brachyrhynchos
819	Clostridium pasteurianum	877	Corynebacterium accolens
820	Clostridium perfringens	878	Corynebacterium argentoratense
821	Clostridium phage	879	Corynebacterium aurimucosum
822	Clostridium phytofermentans	880	Corynebacterium bovis
823	Clostridium ramosum	881	Corynebacterium crenatum
824	Clostridium saccharobutylicum	882	Corynebacterium diphtheriae
825	Clostridium saccharoperbutylacetonicum	883	Corynebacterium doosanense
826	Clostridium scatologenes	884	Corynebacterium durum
827	Clostridium senegalense	885	Corynebacterium efficiens
828	Clostridium sp.	886	Corynebacterium genitalium
829	Clostridium spiroforme	887	Corynebacterium glucuronolyticum
830	Clostridium sporogenes	888	Corynebacterium glutamicum
831	Clostridium sporosphaeroides	889	Corynebacterium halotolerans
832	Clostridium stercorarium	890	Corynebacterium jeikeium
833	Clostridium tetani	891	Corynebacterium lipophiloflavum
834	Clostridium thermocellum	892	Corynebacterium lubricantis
835	Clostridium thermopalmarium	893	Corynebacterium maris
836	Clostridium tunisiense	894	Corynebacterium massiliense
837	Clostridium tyrobutyricum	895	Corynebacterium mastitidis
838	Clostridium ultunense	896	Corynebacterium matruchotii
839	Clostridium viride	897	Corynebacterium nuruki
840	Coccidioides immitis	898	Corynebacterium pilosum
841	Cohnella laeviribosi	899	Corynebacterium propinquum
842	Cohnella panacarvi	900	Corynebacterium pseudodiphtheriticum
843	Cohnella phaseoli	901	Corynebacterium pseudotuberculosis
844	Cohnella thermotolerans	902	Corynebacterium pyruviciproducens
845	Colletotrichum gloeosporioides	903	Corynebacterium resistens
846	Collimonas fungivorans	904	Corynebacterium sp.
847	Collinsella intestinalis	905	Corynebacterium sputi
848	Collinsella sp.	906	Corynebacterium striatum
849	Collinsella tanakaei	907	Corynebacterium terpenotabidum
850	Columba livia	908	Corynebacterium ulcerans
851	Comamonadaceae bacterium	909	Corynebacterium ulceribovis
852	Comamonas badia	910	Corynebacterium urealyticum
853	Comamonas composti	911	Corynebacterium variabile
854	Comamonas sp.	912	Corynebacterium-like bacterium
855	Comamonas testosteroni	913	Cricetulus griseus
856	complete chromosome	914	Crinalium epipsammum
857	Conchiformibius kuhniae	915	Cronobacter pulveris
858	Conchiformibius steedae	916	Cronobacter sakazakii
859	Condylura cristata	917	Cronobacter sp.
860	Conexibacter woesei	918	Cronobacter turicensis
861	Congregibacter litoralis	919	Cronobacter zurichensis
862	Coniophora puteana	920	Cryptobacterium curtum
863	Coniosporium apollinis	921	Cryptobacterium sp.
864	Coprobacillus sp.	922	Cryptococcus neoformans

923	Cryptosporidium muris
924	Cucumibacter marinus
925	Cucumis melo
926	Cucumis sativus
927	Culex pipiens
928	Cupriavidus metallidurans
929	Cupriavidus necator
930	Cupriavidus sp.
931	Cupriavidus taiwanensis
932	Curtobacterium flaccumfaciens
933	Curtobacterium sp.
934	Curvibacter gracilis
935	Curvibacter lanceolatus
936	Cyanidioschyzon merolae
937	Cyanobacterium stanieri
938	Cyanobium gracile
939	Cyanobium sp.
940	Cyanothece sp.
941	Cyclobacterium marinum
942	Cynoglossus semilaevis
943	Cytophaga fermentans
944	Cytophagaceae bacterium
945	Cytophagales bacterium
946	Dactylococcopsis salina
947	Danio rerio
948	Dasypus novemcinctus
949	Dechloromonas agitata
950	Dechloromonas aromatica
951	Dechlorosoma suillum
952	Deefgea rivuli
953	Deferribacter desulfuricans
954	Deferrisoma camini
955	Dehalobacter sp.
956	Dehalococcoides ethenogenes
957	Dehalococcoides mccartyi
958	Dehalogenimonas lykanthroporepellens
959	Deinococcus apachensis
960	Deinococcus aquatilis
961	Deinococcus deserti
962	Deinococcus ficus
963	Deinococcus frigens
964	Deinococcus geothermalis
965	Deinococcus gobiensis
966	Deinococcus maricopensis
967	Deinococcus marmoris
968	Deinococcus murrayi
969	Deinococcus peraridilitoris
970	Deinococcus pimensis
971	Deinococcus proteolyticus
972	Deinococcus radiodurans
973	Deinococcus sp.
974	Deinococcus wulumuqiensis
975	Delftia acidovorans
976	Delftia sp.
977	Delta proteobacterium
978	Demetria terragena
979	Denitrobacterium detoxificans
980	Denitrovibrio acetiphilus

981	Dermabacter sp.
982	Derxia gummosa
983	Desmospora sp.
984	Desulfarculus baarsii
985	Desulfatibacillum aliphaticivorans
986	Desulfatibacillum alkenivorans
987	Desulfatirhabdium butyrativorans
988	Desulfitibacter alkalitolerans
989	Desulfitobacterium dehalogenans
990	Desulfitobacterium dichloroeliminans
991	Desulfitobacterium hafniense
992	Desulfitobacterium sp.
993	Desulfobacca acetoxidans
994	Desulfobacter curvatus
995	Desulfobacter postgatei
996	Desulfobacterium autotrophicum
997	Desulfobacula sp.
998	Desulfobulbus elongatus
999	Desulfobulbus japonicus
1000	Desulfobulbus mediterraneus
1001	Desulfobulbus propionicus
1002	Desulfocapsa sulfexigens
1003	Desulfococcus oleovorans
1004	Desulfocurvus vexinensis
1005	Desulfohalobium retbaense
1006	Desulfomicrobium baculatum
1007	Desulfomicrobium escambiense
1008	Desulfomonile tiedjei
1009	Desulfonatronovibrio hydrogenovorans
1010	Desulfonatronum lacustre
1011	Desulfonatronum thiodismutans
1012	Desulforegula conservatrix
1013	Desulfosarcina sp.
1014	Desulfospira joergensenii
1015	Desulfosporosinus acidiphilus
1016	Desulfosporosinus meridiei
1017	Desulfosporosinus orientis
1018	Desulfosporosinus youngiae
1019	Desulfotalea psychrophila
1020	Desulfotignum balticum
1021	Desulfotomaculum acetoxidans
1022	Desulfotomaculum alcoholivorax
1023	Desulfotomaculum alkaliphilum
1024	Desulfotomaculum carboxydivorans
1025	Desulfotomaculum gibsoniae
1026	Desulfotomaculum halophilum
1027	Desulfotomaculum kuznetsovii
1028	Desulfotomaculum nigrificans
1029	Desulfotomaculum reducens
1030	Desulfotomaculum ruminis
1031	Desulfotomaculum thermocisternum
1032	Desulfovermiculus halophilus
1033	Desulfovibrio aespoeensis
1034	Desulfovibrio africanus
1035	Desulfovibrio alaskensis
1036	Desulfovibrio alcoholivorans
1037	Desulfovibrio aminophilus
1038	Desulfovibrio bastinii

1039	Desulfovibrio cf.
1040	Desulfovibrio cuneatus
1041	Desulfovibrio frigidus
1042	Desulfovibrio gigas
1043	Desulfovibrio hydrothermalis
1044	Desulfovibrio inopinatus
1045	Desulfovibrio longus
1046	Desulfovibrio magneticus
1047	Desulfovibrio oxyclinae
1048	Desulfovibrio putealis
1049	Desulfovibrio salexigens
1050	Desulfovibrio sp.
1051	Desulfovibrio vulgaris
1052	Desulfovibrio zosterae
1053	Desulfovirgula thermocuniculi
1054	Desulfurispirillum indicum
1055	Desulfurispora thermophila
1056	Desulfurivibrio alkaliphilus
1057	Desulfurobacterium sp.
1058	Desulfurobacterium thermolithotrophum
1059	Desulfuromonas sp.
1060	Dialister invisus
1061	Dialister micraerophilus
1062	Dialister sp.
1063	Dialister succinatiphilus
1064	Diaphorina citri
1065	Dichomitus squalens
1066	Dickeya chrysanthemi
1067	Dickeya dadantii
1068	Dickeya dianthicola
1069	Dickeya paradisiaca
1070	Dickeya solani
1071	Dickeya zeae
1072	Dictyoglomus thermophilum
1073	Dictyoglomus turgidum
1074	Dictyostelium discoideum
1075	Dictyostelium fasciculatum
1076	Dielma fastidiosa
1077	Dietzia alimentaria
1078	Dietzia sp.
1079	Dinoroseobacter shibae
1080	Dolichospermum circinale
1081	Donghicola xiamenensis
1082	Dongia sp.
1083	Dorea sp.
1084	Drosophila ananassae
1085	Drosophila erecta
1086	Drosophila grimshawi
1087	Drosophila melanogaster
1088	Drosophila mojavensis
1089	Drosophila persimilis
1090	Drosophila sechellia
1091	Drosophila simulans
1092	Drosophila virilis
1093	Drosophila willistoni
1094	Drosophila yakuba
1095	Duganella violaceinigra
1096	Duganella zoogloeoides

1097	Dyadobacter alkalitolerans
1098	Dyadobacter beijingensis
1099	Dyadobacter crusticola
1100	Dyadobacter fermentans
1101	Dyella ginsengisoli
1102	Dyella japonica
1103	Dysgonomonas capnocytophagoides
1104	Dysgonomonas gadei
1105	Dysgonomonas mossii
1106	Echinicola vietnamensis
1107	Echinops telfairi
1108	Edwardsiella tarda
1109	Eggerthella lenta
1110	Eggerthella sp.
1111	Eggerthia catenaformis
1112	Ehrlichia muris
1113	Eikenella corrodens
1114	Elephantulus edwardii
1115	Elioraea tepidiphila
1116	Elizabethkingia meningoseptica
1117	Elusimicrobium minutum
1118	Emiliania huxleyi
1119	Emticicia oligotrophica
1120	Endozoicomonas elysicola
1121	Enorma massiliensis
1122	Ensifer adhaerens
1123	Ensifer sp.
1124	Entamoeba dispar
1125	Entamoeba histolytica
1126	Entamoeba invadens
1127	Entamoeba nuttalli
1128	Enterobacter aerogenes
1129	Enterobacter asburiae
1130	Enterobacter cancerogenus
1131	Enterobacter cloacae
1132	Enterobacter hormaechei
1133	Enterobacter mori
1134	Enterobacter radicincitans
1135	Enterobacter sp.
1136	Enterobacteriaceae bacterium
1137	Enterocytozoon bieneusi
1138	Enterorhabdus caecimuris
1139	Enterorhabdus mucosicola
1140	Enterovibrio calviensis
1141	Enterovibrio norvegicus
1142	Entomoplasma lucivorax
1143	Entomoplasma luminosum
1144	Entomoplasma melaleucae
1145	Entomoplasma somnilux
1146	Epilithonimonas tenax
1147	Eptesicus fuscus
1148	Epulopiscium sp.
1149	Equus caballus
1150	Equus przewalskii
1151	Eremococcus coleocola
1152	Erinaceus europaeus
1153	Erwinia amylovora
1154	Erwinia billingiae

1155	Erwinia phage
1156	Erwinia toletana
1157	Erwinia tracheiphila
1158	Erysipelothrix rhusiopathiae
1159	Erysipelothrix tonsillarum
1160	Erysipelotrichaceae bacterium
1161	Erythrobacter litoralis
1162	Erythrobacter sp.
1163	Ethanoligenens harbinense
1164	Eubacteriaceae bacterium
1165	Eubacterium biforme
1166	Eubacterium brachy
1167	Eubacterium cellulosolvens
1168	Eubacterium coprostanoligenes
1169	Eubacterium cylindroides
1170	Eubacterium desmolans
1171	Eubacterium dolichum
1172	Eubacterium infirmum
1173	Eubacterium limosum
1174	Eubacterium plexicaudatum
1175	Eubacterium ramulus
1176	Eubacterium saburreum
1177	Eubacterium saphenum
1178	Eubacterium sp.
1179	Eubacterium xylanophilum
1180	Eubacterium yurii
1181	Eudoraea adriatica
1182	Eutrema salsugineum
1183	Eutypa lata
1184	Exiguobacterium acetylicum
1185	Exiguobacterium antarcticum
1186	Exiguobacterium aurantiacum
1187	Exiguobacterium oxidotolerans
1188	Exiguobacterium pavilionensis
1189	Exiguobacterium sibiricum
1190	Exiguobacterium sp.
1191	Exiguobacterium undae
1192	Facklamia hominis
1193	Facklamia ignava
1194	Facklamia languida
1195	Falco cherrug
1196	Falco peregrinus
1197	Felis catus
1198	Ferrimonas balearica
1199	Ferrimonas futtsuensis
1200	Ferrimonas kyonanensis
1201	Ferrimonas senticii
1202	Ferrovum myxofaciens
1203	Fibrella aestuarina
1204	Fibrobacter succinogenes
1205	Ficedula albicollis
1206	Filifactor alocis
1207	Finegoldia magna
1208	Firmicutes bacterium
1209	Fischerella muscicola
1210	Fischerella sp.
1211	Fischerella thermalis
1212	Flavobacteriaceae bacterium

1213	Flavobacterium branchiophilum
1214	Flavobacterium chungangense
1215	Flavobacterium denitrificans
1216	Flavobacterium frigidarium
1217	Flavobacterium gelidilacus
1218	Flavobacterium indicum
1219	Flavobacterium johnsoniae
1220	Flavobacterium rivuli
1221	Flavobacterium sasangense
1222	Flavobacterium soli
1223	Flavobacterium sp.
1224	Flavobacterium subsaxonicum
1225	Flavobacterium succinicans
1226	Flavobacterium suncheonense
1227	Flavonifractor plautii
1228	Flectobacillus major
1229	Flexibacter roseolus
1230	Flexistipes sinusarabici
1231	Flexithrix dorotheae
1232	Fluviicola taffensis
1233	Fodinicurvata fenggangensis
1234	Fodinicurvata sediminis
1235	Fragaria vesca
1236	Francisella cf.
1237	Francisella sp.
1238	Frankia alni
1239	Frankia sp.
1240	Frankia symbiont
1241	Frateuria aurantia
1242	Fructobacillus fructosus
1243	Fusicatenibacter saccharivorans
1244	Fusobacterium gonidiaformans
1245	Fusobacterium mortiferum
1246	Fusobacterium necrophorum
1247	Fusobacterium nucleatum
1248	Fusobacterium perfoetens
1249	Fusobacterium periodonticum
1250	Fusobacterium russii
1251	Fusobacterium sp.
1252	Fusobacterium ulcerans
1253	Fusobacterium varium
1254	Gaeumannomyces graminis
1255	Galeopterus variegatus
1256	Gallibacterium anatis
1257	Gallus gallus
1258	Gamma proteobacterium
1259	gamma proteobacterium
1260	Gardnerella vaginalis
1261	Gayadomonas joobiniege
1262	Geitlerinema sp.
1263	Gelidibacter mesophilus
1264	Gemella bergeriae
1265	Gemella cuniculi
1266	Gemella haemolysans
1267	Gemella moribillum
1268	Gemella sanguinis
1269	Geminicoccus roseus
1270	Geminocystis herdmanii

1271	Gemmata obscuriglobus
1272	Gemmatimonadetes bacterium
1273	Gemmatimonas aurantiaca
1274	Gemmatimonas sp.
1275	Gemmiger formicilis
1276	Gemmobacter nectariphilus
1277	Geobacillus caldoxylosilyticus
1278	Geobacillus kaustophilus
1279	Geobacillus sp.
1280	Geobacillus thermocatenulatus
1281	Geobacillus thermodenitrificans
1282	Geobacillus thermoglucosidans
1283	Geobacillus vulcani
1284	Geobacter bemidjiensis
1285	Geobacter bremensis
1286	Geobacter daltonii
1287	Geobacter lovleyi
1288	Geobacter metallireducens
1289	Geobacter sp.
1290	Geobacter sulfurreducens
1291	Geobacter uraniireducens
1292	Geodermatophilaceae bacterium
1293	Geodermatophilus obscurus
1294	Geopsychrobacter electrodiphilus
1295	Geospiza fortis
1296	Geothrix fermentans
1297	Geovibrio sp.
1298	Gillisia limnaea
1299	Gillisia sp.
1300	Gilvimarinus chinensis
1301	Glaciecola nitratireducens
1302	Glaciecola sp.
1303	Glaciibacter superstes
1304	Glarea lozoyensis
1305	Gloeobacter kilaueensis
1306	Gloeobacter violaceus
1307	Gloeocapsa sp.
1308	Glomeribacter sp.
1309	Gluconacetobacter diazotrophicus
1310	Gluconacetobacter europaeus
1311	Gluconacetobacter hansenii
1312	Gluconacetobacter sp.
1313	Gluconacetobacter xylinus
1314	Gluconobacter frateurii
1315	Gluconobacter oxydans
1316	Glycine max
1317	Glycomyces arizonensis
1318	Glycomyces sp.
1319	Gordonia amicalis
1320	Gordonia bronchialis
1321	Gordonia hirsuta
1322	Gordonia kroppenstedtii
1323	Gordonia polyisoprenivorans
1324	Gordonia shandongensis
1325	Gorilla gorilla
1326	Gracilaria salicornia
1327	Gracilibacillus lacisalsi
1328	Gracilibacter thermotolerans

1329	Gracilimonas tropica
1330	Gramella forsetii
1331	Gramella portivictoriae
1332	Granulibacter bethesdensis
1333	Granulicatella adiacens
1334	Granulicatella elegans
1335	Granulicella mallensis
1336	Granulicella tundricola
1337	Granulicoccus phenolivorans
1338	Gryllotalpicola ginsengisoli
1339	Guillardia theta
1340	Gulosibacter molinativorax
1341	Haemophilus influenzae
1342	Haemophilus parasuis
1343	Haemophilus sputorum
1344	Hafnia alvei
1345	Hahella chejuensis
1346	Hahella ganghwensis
1347	Haladaptatus paucihalophilus
1348	Halanaerobium hydrogeniformans
1349	Haliangium ochraceum
1350	Haliscomenobacter hydrossis
1351	Hallella seregens
1352	Haloarcula hispanica
1353	Haloarcula marismortui
1354	Halobacillus halophilus
1355	Halobacillus kuroshimensis
1356	Halobacterium salinarum
1357	Halobacteroides halobius
1358	Halobiforma lacisalsi
1359	Halococcus hamelinensis
1360	Haloferax mediterranei
1361	Haloferax volcanii
1362	Haloglycomyces albus
1363	Halomicrobium katesii
1364	Halomonas anticariensis
1365	Halomonas boliviensis
1366	Halomonas elongata
1367	Halomonas halocynthiae
1368	Halomonas halodenitrificans
1369	Halomonas jeotgali
1370	Halomonas smyrnensis
1371	Halomonas sp.
1372	Halomonas stevensii
1373	Halomonas zhanjiangensis
1374	Halomonas zincidurans
1375	Halonatronum saccharophilum
1376	halophilic archaeon
1377	Halopiger xanaduensis
1378	Haloplasma contractile
1379	Haloquadratum walsbyi
1380	Halorhodospira halochloris
1381	Halorhodospira halophila
1382	Halorubrum lacusprofundi
1383	Halotalea alkalilenta
1384	Haloterrigena turkmenica
1385	Halothermothrix orenii
1386	Halothiobacillus neapolitanus

1387	Halovivax ruber
1388	Hamadaea tsunoensis
1389	Hammondia hammondi
1390	Haplochromis burtoni
1391	Helcococcus kunzii
1392	Helcococcus sueciensis
1393	Helicobacter bilis
1394	Helicobacter bizzozeronii
1395	Helicobacter canadensis
1396	Helicobacter cetorum
1397	Helicobacter felis
1398	Helicobacter hepaticus
1399	Helicobacter pullorum
1400	Helicobacter pylori
1401	Helicobacter rodentium
1402	Helicobacter winghamensis
1403	Heliobacterium modesticaldum
1404	Hellea balneolensis
1405	Helobdella robusta
1406	Henriciella marina
1407	Herbaspirillum lusitanum
1408	Herbaspirillum massiliense
1409	Herbaspirillum rubrisubalbicans
1410	Herbaspirillum seropedicae
1411	Herbidospora cretacea
1412	Herminiimonas sp.
1413	Herpetosiphon aurantiacus
1414	Heterocephalus glaber
1415	Hippea alviniae
1416	Hippea sp.
1417	Hirschia baltica
1418	Hoeflea phototrophica
1419	Hoeflea sp.
1420	Holophaga foetida
1421	Homo sapiens
1422	Howardella ureilytica
1423	Humibacter albus
1424	Hydra magnipapillata
1425	Hymenobacter aerophilus
1426	Hymenobacter norwichensis
1427	Hyphomicrobium denitrificans
1428	Hyphomicrobium nitrativorans
1429	Hyphomicrobium sp.
1430	Hyphomicrobium zavarzinii
1431	Hyphomonas neptunium
1432	Ichthyophthirius multifiliis
1433	Ideonella sp.
1434	Idiomarina loihiensis
1435	Idiomarina sediminum
1436	Ignatzschineria larvae
1437	Ignavibacterium album
1438	Ignicoccus hospitalis
1439	Ilyobacter polytropus
1440	Inquilinus limosus
1441	Intestinimonas butyriciproducens
1442	Intrasporangiaceae bacterium
1443	Intrasporangium calvum
1444	Isoptericola variabilis

1445	Isosphaera pallida
1446	Ixodes scapularis
1447	Jaculus jaculus
1448	Jakoba bahamiensis
1449	Janibacter sp.
1450	Jannaschia sp.
1451	Janthinobacterium lividum
1452	Janthinobacterium sp.
1453	Jeotgalicoccus marinus
1454	Jeotgalicoccus psychrophilus
1455	Jiangella gansuensis
1456	Johnsonella ignava
1457	Jonesia quinghaiensis
1458	Jonquetella anthropi
1459	Joostella marina
1460	Kaistia adipata
1461	Kaistia granuli
1462	Kamptonema formosum
1463	Kandleria vitulina
1464	Kangiella aquimarina
1465	Ketogulonigenium vulgarum
1466	Kineococcus radiotolerans
1467	Kingella denitrificans
1468	Kingella oralis
1469	Kitasatospora setae
1470	Kitasatospora sp.
1471	Klebsiella oxytoca
1472	Klebsiella pneumoniae
1473	Klebsiella sp.
1474	Klebsiella variicola
1475	Kocuria atrinae
1476	Kocuria rhizophila
1477	Kocuria sp.
1478	Kordia algicida
1479	Kordiimonas gwangyangensis
1480	Kosmotoga olearia
1481	Kozakia baliensis
1482	Kribbella catacumbae
1483	Kribbella flavida
1484	Kurthia huakuii
1485	Kurthia massiliensis
1486	Kurthia sp.
1487	Kushneria aurantia
1488	Kutzneria albida
1489	Kyrpidia tusciae
1490	Labrenzia alexandrii
1491	Labrenzia sp.
1492	Lachnobacterium bovis
1493	Lachnoclostridium phytofermentans
1494	Lachnospira multipara
1495	Lachnospira pectinoschiza
1496	Lachnospiraceae bacterium
1497	Lachnospiraceae oral
1498	Lacinutrix sp.
1499	Lacticigenium naphtae
1500	Lactobacillus acidipiscis
1501	Lactobacillus acidophilus
1502	Lactobacillus amylovorus

1503	Lactobacillus antri
1504	Lactobacillus apodemi
1505	Lactobacillus buchneri
1506	Lactobacillus casei
1507	Lactobacillus ceti
1508	Lactobacillus coryniformis
1509	Lactobacillus delbrueckii
1510	Lactobacillus equi
1511	Lactobacillus farciminis
1512	Lactobacillus fermentum
1513	Lactobacillus gasseri
1514	Lactobacillus hamsteri
1515	Lactobacillus harbinensis
1516	Lactobacillus hayakitensis
1517	Lactobacillus helveticus
1518	Lactobacillus hilgardii
1519	Lactobacillus iners
1520	Lactobacillus jensenii
1521	Lactobacillus johnsonii
1522	Lactobacillus kefiranofaciens
1523	Lactobacillus kitasatonis
1524	Lactobacillus mali
1525	Lactobacillus namurensis
1526	Lactobacillus nodensis
1527	Lactobacillus parabrevis
1528	Lactobacillus paracasei
1529	Lactobacillus parafarraginis
1530	Lactobacillus paralimentarius
1531	Lactobacillus plantarum
1532	Lactobacillus pobuzihii
1533	Lactobacillus psittaci
1534	Lactobacillus reuteri
1535	Lactobacillus rhamnosus
1536	Lactobacillus rossiae
1537	Lactobacillus sakei
1538	Lactobacillus salivarius
1539	Lactobacillus sanfranciscensis
1540	Lactobacillus shenzhenensis
1541	Lactobacillus sp.
1542	Lactobacillus suebicus
1543	Lactobacillus versmoldensis
1544	Lactobacillus zeae
1545	Lactococcus garvieae
1546	Lactococcus lactis
1547	Lactococcus phage
1548	Lactonifactor longoviformis
1549	Lamprocystis purpurea
1550	Laribacter hongkongensis
1551	Latimeria chalumnae
1552	Lautropia mirabilis
1553	Leadbetterella byssophila
1554	Lebetimonas sp.
1555	Lechevalieria aerocolonigenes
1556	Leeia oryzae
1557	Leeuwenhoekiella blandensis
1558	Leeuwenhoekiella sp.
1559	Legionella cherrii
1560	Legionella drancourtii
1561	Legionella geestiana
1562	Legionella longbeachae
1563	Legionella pneumophila
1564	Leifsonia aquatica
1565	Leifsonia sp.
1566	Leifsonia xyli
1567	Leishmania braziliensis
1568	Leishmania infantum
1569	Leishmania mexicana
1570	Leisingera aquimarina
1571	Leisingera methylohalidivorans
1572	Leisingera nanhaiensis
1573	Leminorella grimontii
1574	Lentibacillus jeotgali
1575	Lentisphaerae bacterium
1576	Lentzea albidocapillata
1577	Lepisosteus oculatus
1578	Leptolyngbya boryana
1579	Leptolyngbya sp.
1580	Leptonema illini
1581	Leptonychotes weddellii
1582	Leptosphaeria maculans
1583	Leptospira borgpetersenii
1584	Leptospira kirschneri
1585	Leptospirillum ferriphilum
1586	Leptothrix cholodnii
1587	Leptotrichia goodfellowii
1588	Leptotrichia hofstadii
1589	Leptotrichia shahii
1590	Leptotrichia sp.
1591	Leptotrichia trevisanii
1592	Leptotrichia wadei
1593	Leucobacter chironomi
1594	Leucobacter chromiiresistens
1595	Leucobacter salsicius
1596	Leucobacter sp.
1597	Leuconostoc citreum
1598	Leuconostoc fallax
1599	Leuconostoc gelidum
1600	Leuconostoc inhae
1601	Leuconostoc kimchii
1602	Leuconostoc lactis
1603	Leuconostoc pseudomesenteroides
1604	Leucothrix mucor
1605	Lewinella cohaerens
1606	Lewinella persica
1607	Liberibacter crescens
1608	Limnohabitans sp.
1609	Lipotes vexillifer
1610	Listeria grayi
1611	Listeria innocua
1612	Listeria monocytogenes
1613	Listeria welshimeri
1614	Listonella anguillarum
1615	Lithodesmium undulatum
1616	Loa loa
1617	Loktanella hongkongensis
1618	Loktanella vestfoldensis

1619	Longispora albida
1620	Lonsdalea quercina
1621	Lottia gigantea
1622	Loxodonta africana
1623	Luteimonas huabeiensis
1624	Luteimonas mephitis
1625	Luteimonas sp.
1626	Lyngbya majuscula
1627	Lysinibacillus boronitolerans
1628	Lysinibacillus fusiformis
1629	Lysinibacillus massiliensis
1630	Lysinibacillus sphaericus
1631	Lysobacter antibioticus
1632	Lysobacter sp.
1633	Macaca fascicularis
1634	Macaca mulatta
1635	Macrococcus caseolyticus
1636	Magnetococcus marinus
1637	Magnetospirillum gryphiswaldense
1638	Magnetospirillum magneticum
1639	Mahella australiensis
1640	Malus x
1641	Manacus vitellinus
1642	Mannheimia haemolytica
1643	Maribacter antarcticus
1644	Maribacter sp.
1645	Maricaulis maris
1646	Maricaulis sp.
1647	marine gamma
1648	Marinilabilia salmonicolor
1649	Marinimicrobia bacterium
1650	Marinimicrobium agarilyticum
1651	Marinimicrobium sp.
1652	Marinithermus hydrothermalis
1653	Marinitoga piezophila
1654	Marinobacter aquaeolei
1655	Marinobacter daepoensis
1656	Marinobacter lipolyticus
1657	Marinobacter nanhaiticus
1658	Marinobacter sp.
1659	Marinobacterium jannaschii
1660	Marinobacterium litorale
1661	Marinobacterium rhizophilum
1662	Marinobacterium stanieri
1663	Marinococcus halotolerans
1664	Marinomonas mediterranea
1665	Marinomonas posidonica
1666	Marinomonas sp.
1667	Marinospirillum insulare
1668	Marinospirillum minutulum
1669	Mariprofundus ferrooxydans
1670	Maritalea myrionectae
1671	Maritimibacter alkaliphilus
1672	Marivirga tractuosa
1673	Marmoricola aequoreus
1674	Marmoricola sp.
1675	Marssonina brunnea
1676	Martelella mediterranea

1677	Martelella sp.
1678	Massilia alkalitolerans
1679	Massilia niastensis
1680	Massilia timonae
1681	Mastigocladopsis repens
1682	Mastigocoleus testarum
1683	Maylandia zebra
1684	Medicago truncatula
1685	Megachile rotundata
1686	Megamonas funiformis
1687	Megamonas hypermegale
1688	Meganema perideroedes
1689	Megasphaera elsdenii
1690	Megasphaera micronuciformis
1691	Meiothermus cerbereus
1692	Meiothermus chliarophilus
1693	Meiothermus ruber
1694	Meiothermus rufus
1695	Meiothermus silvanus
1696	Meiothermus taiwanensis
1697	Meiothermus timidus
1698	Melampsora larici-populina
1699	Meleagris gallopavo
1700	Melissococcus plutonius
1701	Melitea salexigens
1702	Melopsittacus undulatus
1703	Merops nubicus
1704	Mesocricetus auratus
1705	Mesoplasma chauliocola
1706	Mesoplasma florum
1707	Mesoplasma grammopterae
1708	Mesoplasma syrphidae
1709	Mesorhizobium australicum
1710	Mesorhizobium ciceri
1711	Mesorhizobium loti
1712	Mesorhizobium sp.
1713	Mesotoga prima
1714	Metarhizium acridum
1715	Metarhizium anisopliae
1716	Metascardovia criceti
1717	Metaseiulus occidentalis
1718	Methanobacterium sp.
1719	Methanobrevibacter smithii
1720	Methanobrevibacter sp.
1721	Methanocaldococcus fervens
1722	Methanocaldococcus jannaschii
1723	Methanocella arvoryzae
1724	Methanocella paludicola
1725	Methanococcoides burtonii
1726	Methanococcus aeolicus
1727	Methanococcus maripaludis
1728	Methanococcus voltae
1729	Methanocorpusculum labreanum
1730	Methanoculleus bourgensis
1731	Methanoculleus marisnigri
1732	Methanoculleus sp.
1733	Methanofollis liminatans
1734	Methanolobus psychrophilus

1735	Methanomassiliicoccus luminyensis
1736	Methanomassiliicoccus sp.
1737	Methanomethylovorans hollandica
1738	Methanoplanus limicola
1739	Methanoplanus petrolearius
1740	Methanoregula boonei
1741	Methanoregula formicicum
1742	Methanosaeta harundinacea
1743	Methanosaeta thermophila
1744	Methanosalsum zhilinae
1745	Methanosarcina barkeri
1746	Methanosarcina mazei
1747	Methanosphaera stadtmanae
1748	Methanospirillum hungatei
1749	Methanothermobacter thermautotrophicus
1750	Methanothermococcus okinawensis
1751	Methylacidiphilum infernorum
1752	Methylibium petroleiphilum
1753	Methylobacillus flagellatus
1754	Methylobacillus glycogenes
1755	Methylobacter luteus
1756	Methylobacter marinus
1757	Methylobacter tundripaludum
1758	Methylobacterium extorquens
1759	Methylobacterium nodulans
1760	Methylobacterium populi
1761	Methylobacterium radiotolerans
1762	Methylobacterium sp.
1763	Methylocaldum szegediense
1764	Methylocella silvestris
1765	Methylococcus capsulatus
1766	Methylocystis parvus
1767	Methylocystis rosea
1768	Methyloferula stellata
1769	Methylohalobius crimeensis
1770	Methylomarinum vadi
1771	Methylomicrobium agile
1772	Methylomicrobium buryatense
1773	Methylomonas methanica
1774	Methylomonas sp.
1775	Methylophilus sp.
1776	Methylopila sp.
1777	Methylosarcina fibrata
1778	Methylosarcina lacus
1779	Methylosinus sp.
1780	Methylosinus trichosporium
1781	Methylotenera mobilis
1782	Methyloversatilis sp.
1783	Methyloversatilis universalis
1784	Methylovorus glucosetrophus
1785	Methylovorus sp.
1786	Methylovulum miyakonense
1787	Micavibrio aeruginosavorus
1788	Microbacterium barkeri
1789	Microbacterium gubbeenense
1790	Microbacterium indicum
1791	Microbacterium luticocti
1792	Microbacterium maritypicum

1793	Microbacterium paraoxydans
1794	Microbacterium sp.
1795	Microbacterium testaceum
1796	Microbacterium yannicii
1797	Microbispora rosea
1798	Microbulbifer agarilyticus
1799	Microbulbifer variabilis
1800	Micrococcus luteus
1801	Microcoleus sp.
1802	Microcystis aeruginosa
1803	Microlunatus ginsengisoli
1804	Microlunatus phosphovorus
1805	Micromonas pusilla
1806	Micromonospora chokoriensis
1807	Micromonospora globosa
1808	Micromonospora parva
1809	Micromonospora purpureochromogenes
1810	Micromonospora sp.
1811	Microplitis demolitor
1812	Microtetraspora glauca
1813	Microtus ochrogaster
1814	Microvirga sp.
1815	Microvirgula aerodenitrificans
1816	Millerozyma farinosa
1817	Mimivirus terra2
1818	Mitsuokella multacida
1819	Mitsuokella sp.
1820	Mobiluncus curtisii
1821	Mobiluncus mulieris
1822	Modestobacter marinus,
1823	Mogibacterium neglectum
1824	Mogibacterium pumilum
1825	Moniliophthora perniciosa
1826	Monodelphis domestica
1827	Monosiga brevicollis
1828	Moorella thermoacetica
1829	Moraxella boevrei
1830	Moraxella caprae
1831	Morganella morganii
1832	Moritella marina
1833	Mucilaginibacter paludis
1834	Mucispirillum schaedleri
1835	Muricauda ruestringensis
1836	Mus musculus
1837	Musca domestica
1838	Mustela putorius
1839	Myceliophthora thermophila
1840	Mycetocola saprophilus
1841	Mycoplasma agalactiae
1842	Mycoplasma crocodyli
1843	Mycoplasma gallinarum
1844	Mycoplasma gallisepticum
1845	Mycoplasma hyopneumoniae
1846	Mycoplasma hyorhinis
1847	Mycoplasma lipofaciens
1848	Mycoplasma moatsii
1849	Mycoplasma mobile
1850	Mycoplasma molare

1851	Mycoplasma mycoides
1852	Mycoplasma orale
1853	Mycoplasma penetrans
1854	Mycoplasma pirum
1855	Mycoplasma pulmonis
1856	Mycoplasma putrefaciens
1857	Mycoplasma salivarium
1858	Mycoplasma sp.
1859	Mycoplasma spumans
1860	Mycoplasma synoviae
1861	Myotis brandtii
1862	Myotis davidii
1863	Myotis lucifugus
1864	Myroides injenensis
1865	Myroides odoratimimus
1866	Myxococcus fulvus
1867	Myxococcus stipitatus
1868	Myxococcus xanthus
1869	Naegleria gruberi
1870	Nakamurella lactea
1871	Nakamurella multipartita
1872	Nannospalax galili
1873	Nasonia vitripennis
1874	Natranaerobius thermophilus
1875	Natranaerovirga pectinivora
1876	Natrialba magadii
1877	Natrinema pellirubrum
1878	Natronobacterium gregoryi
1879	Natronococcus occultus
1880	Natronomonas moolapensis
1881	Naumovozyma dairenensis
1882	Nautilia profundicola
1883	Nectria haematococca
1884	Neisseria bacilliformis
1885	Neisseria gonorrhoeae
1886	Neisseria lactamica
1887	Neisseria macacae
1888	Neisseria meningitidis
1889	Neisseria polysaccharea
1890	Neisseria shayeganii
1891	Neisseria sicca
1892	Neisseria sp.
1893	Neisseria wadsworthii
1894	Nematostella vectensis
1895	Neolamprologus brichardi
1896	Neosartorya fischeri
1897	Neptunomonas japonica
1898	Nesiotobacter exalbescens
1899	Nesterenkonia alba
1900	Nesterenkonia sp.
1901	Nevskia ramosa
1902	Nevskia soli
1903	Niabella aurantiaca
1904	Niastella koreensis
1905	Nisaea denitrificans
1906	Nitella hyalina
1907	Nitratifractor salsuginis
1908	Nitratireductor aquibiodomus

1909	Nitrobacter hamburgensis
1910	Nitrobacter sp.
1911	Nitrococcus mobilis
1912	Nitrosomonas cryotolerans
1913	Nitrosomonas europaea
1914	Nitrosopumilus maritimus
1915	Nitrosospira briensis
1916	Nitrosospira multiformis
1917	Nitrospina gracilis
1918	Nitrospina sp.
1919	Nitrospirillum amazonense
1920	Niveispirillum irakense
1921	Nocardia brasiliensis
1922	Nocardia cyriacigeorgica
1923	Nocardia farcinica
1924	Nocardia otitidiscaviarum
1925	Nocardia rhamnosiphila
1926	Nocardia sp.
1927	Nocardioidaceae bacterium
1928	Nocardioides alkalitolerans
1929	Nocardioides halotolerans
1930	Nocardioides insulae
1931	Nocardioides sp.
1932	Nocardiopsis alba
1933	Nocardiopsis baichengensis
1934	Nocardiopsis chromatogenes
1935	Nocardiopsis dassonvillei
1936	Nocardiopsis ganjiahuensis
1937	Nocardiopsis gilva
1938	Nocardiopsis halophila
1939	Nocardiopsis lucentensis
1940	Nocardiopsis potens
1941	Nocardiopsis prasina
1942	Nocardiopsis salina
1943	Nocardiopsis sp.
1944	Nocardiopsis synnemataformans
1945	Nocardiopsis valliformis
1946	Nocardiopsis xinjiangensis
1947	Nodosilinea nodulosa
1948	Nomascus leucogenys
1949	Nonomuraea coxensis
1950	Nostoc punctiforme
1951	Nostoc sp.
1952	Novispirillum itersonii
1953	Novosphingobium acidiphilum
1954	Novosphingobium aromaticivorans
1955	Novosphingobium lindaniclasticum
1956	Novosphingobium nitrogenifigens
1957	Novosphingobium sp.
1958	Oceanicaulis alexandrii
1959	Oceanicola batsensis
1960	Oceanicola granulosus
1961	Oceanicola nanhaiensis
1962	Oceanicola sp.
1963	Oceanimonas smirnovii
1964	Oceanimonas sp.
1965	Oceanithermus profundus
1966	Oceanobacillus iheyensis

1967	Oceanobacillus kimchii
1968	Oceanobacter kriegii
1969	Oceanospirillum beijerinckii
1970	Ochotona princeps
1971	Ochrobactrum anthropi
1972	Ochrobactrum intermedium
1973	Ochrobactrum rhizosphaerae
1974	Ochrobactrum sp.
1975	Octadecabacter antarcticus
1976	Octadecabacter arcticus
1977	Octodon degus
1978	Odobenus rosmarus
1979	Odoribacter laneus
1980	Odoribacter sp.
1981	Oenococcus kitaharae
1982	Oerskovia turbata
1983	Oligella urethralis
1984	Oligotropha carboxidovorans
1985	Olivibacter sitiensis
1986	Olleya sp.
1987	Olsenella sp.
1988	Olsenella uli
1989	Opisthorchis viverrini
1990	Opitutaceae bacterium
1991	Opitutus terrae
1992	Orcinus orca
1993	Orenia marismortui
1994	Oreochromis niloticus
1995	Oribacterium sp.
1996	Ornithinibacillus scapharcae
1997	Ornithinimicrobium pekingense
1998	Ornithoctonus huwena
1999	Ornithorhynchus anatinus
2000	Orycteropus afer
2001	Oryctolagus cuniculus
2002	Oryza brachyantha
2003	Oryza sativa
2004	Oryzias latipes
2005	Oryzias latipes,
2006	Oscillatoria nigro-viridis
2007	Oscillatoria sp.
2008	Oscillatoriales cyanobacterium
2009	Oscillibacter sp.
2010	Oscillibacter valericigenes
2011	Oscillochloris trichoides
2012	Oscillospiraceae bacterium
2013	Ostreococcus lucimarinus
2014	Ostreococcus tauri
2015	Otolemur garnettii
2016	Ottowia thiooxydans
2017	Ovis aries
2018	Oxalobacter formigenes
2019	Oxalobacteraceae bacterium
2020	Paenibacillus alginolyticus
2021	Paenibacillus assamensis
2022	Paenibacillus azotofixans
2023	Paenibacillus barengoltzii
2024	Paenibacillus daejeonensis

2025	Paenibacillus ehimensis
2026	Paenibacillus elgii
2027	Paenibacillus fonticola
2028	Paenibacillus forsythiae
2029	Paenibacillus ginsengihumi
2030	Paenibacillus graminis
2031	Paenibacillus harenae
2032	Paenibacillus larvae
2033	Paenibacillus massiliensis
2034	Paenibacillus mucilaginosus
2035	Paenibacillus panacisoli
2036	Paenibacillus pasadenensis
2037	Paenibacillus peoriae
2038	Paenibacillus pinihumi
2039	Paenibacillus polymyxa
2040	Paenibacillus pueri
2041	Paenibacillus rigui
2042	Paenibacillus sanguinis
2043	Paenibacillus senegalensis
2044	Paenibacillus sp.
2045	Paenibacillus taiwanensis
2046	Paenibacillus terrae
2047	Paenibacillus terrigena
2048	Paenibacillus zanthoxyli
2049	Paenisporosarcina sp.
2050	Paludibacter propionicigenes
2051	Paludibacterium yongneupense
2052	Pan paniscus
2053	Pan troglodytes
2054	Pandoraea sp.
2055	Pannonibacter phragmitetus
2056	Panthera tigris
2057	Pantholops hodgsonii
2058	Pantoea agglomerans
2059	Pantoea ananatis
2060	Pantoea dispersa
2061	Pantoea sp.
2062	Pantoea vagans
2063	Papillibacter cinnamivorans
2064	Papio anubis
2065	Paracoccidioides brasiliensis
2066	Paracoccus aminophilus
2067	Paracoccus denitrificans
2068	Paracoccus pantotrophus
2069	Paracoccus sp.
2070	Paracoccus yeei
2071	Paracoccus zeaxanthinifaciens
2072	Paraeggerthella hongkongensis
2073	Paramecium tetraurelia
2074	Paraoerskovia marina
2075	Paraprevotella clara
2076	Paraprevotella xylaniphila
2077	Parascardovia denticolens
2078	Parcubacteria bacterium
2079	Parvibaculum lavamentivorans
2080	Parvimonas micra
2081	Parvularcula oceani
2082	Pasteurella multocida

2083	Patulibacter americanus
2084	Patulibacter medicamentivorans
2085	Patulibacter minatonensis
2086	Paucisalibacillus globulus
2087	Paulinella chromatophora
2088	Pectobacterium atrosepticum
2089	Pectobacterium carotovorum
2090	Pectobacterium sp.
2091	Pectobacterium wasabiae
2092	Pediculus humanus
2093	Pediococcus claussenii
2094	Pediococcus pentosaceus
2095	Pedobacter agri
2096	Pedobacter borealis
2097	Pedobacter glucosidilyticus
2098	Pedobacter heparinus
2099	Pedobacter oryzae
2100	Pedobacter saltans
2101	Pedobacter sp.
2102	Pelagibaca bermudensis
2103	Pelagibacterium halotolerans
2104	Pelistega sp.
2105	Pelobacter carbinolicus
2106	Pelobacter propionicus
2107	Pelobacter seleniigenes
2108	Pelodiscus sinensis
2109	Pelosinus sp.
2110	Pelotomaculum thermopropionicum
2111	Peptoclostridium difficile
2112	Peptococcus niger
2113	Peptostreptococcaceae bacterium
2114	Peptostreptococcus anaerobius
2115	Perkinsus marinus
2116	Perlucidibaca piscinae
2117	Peromyscus maniculatus
2118	Persephonella marina
2119	Persephonella sp.
2120	Pestalotiopsis fici
2121	Phaeobacter arcticus
2122	Phaeobacter caeruleus
2123	Phaeobacter daeponensis
2124	Phaeobacter gallaeciensis
2125	Phaeobacter inhibens
2126	Phaeocystis globosa
2127	Phaeodactylum tricornutum
2128	Phanerochaete carnosa
2129	Phenylobacterium zucineum
2130	Phoenix dactylifera
2131	Photobacterium halotolerans
2132	Photobacterium profundum
2133	Photorhabdus temperata
2134	Phycicoccus jejuensis
2135	Phycisphaera mikurensis
2136	Phyllobacterium sp.
2137	Physcomitrella patens
2138	Physeter catodon
2139	Phytophthora parasitica
2140	Pichia pastoris

2141	Pilimelia anulata
2142	Pirellula staleyi
2143	Piscirickettsia salmonis
2144	Planctomyces brasiliensis
2145	Planktothrix agardhii
2146	Planktothrix prolifica
2147	Planktothrix rubescens
2148	Plasmodium chabaudi
2149	Plasmodium cynomolgi
2150	Plasmodium falciparum
2151	Plasmodium knowlesi
2152	Plasmodium vinckei
2153	Plasmodium yoelii
2154	Plautia stali
2155	Pleomorphomonas koreensis
2156	Pleomorphomonas oryzae
2157	Pleurocapsa sp.
2158	Poecilia formosa
2159	Poecilia reticulata
2160	Poinsettia branch-inducing
2161	Polaribacter irgensii
2162	Polaribacter sp.
2163	Polaromonas glacialis
2164	Polaromonas naphthalenivorans
2165	Polaromonas sp.
2166	Polycyclovorans algicola
2167	Polymorphum gilvum
2168	Polynucleobacter necessarius
2169	Pongo abelii
2170	Pontibacillus halophilus
2171	Pontibacillus marinus
2172	Pontibacter actiniarum
2173	Pontibacter roseus
2174	Ponticaulis koreensis
2175	Populus trichocarpa
2176	Poribacteria bacterium
2177	Porphyrobacter cryptus
2178	Porphyrobacter sp.
2179	Porphyromonas asaccharolytica
2180	Porphyromonas bennonis
2181	Porphyromonas cangingivalis
2182	Porphyromonas catoniae
2183	Porphyromonas gingivalis
2184	Porphyromonas gulae
2185	Porphyromonas levii
2186	Porphyromonas macacae
2187	Porphyromonas somerae
2188	Porphyromonas sp.
2189	Porphyromonas uenonis
2190	Prauserella rugosa
2191	Prevotella albensis
2192	Prevotella amnii
2193	Prevotella aurantiaca
2194	Prevotella baroniae
2195	Prevotella brevis
2196	Prevotella bryantii
2197	Prevotella corporis
2198	Prevotella dentalis

2199	Prevotella dentasini
2200	Prevotella denticola
2201	Prevotella enoeca
2202	Prevotella falsenii
2203	Prevotella fusca
2204	Prevotella histicola
2205	Prevotella intermedia
2206	Prevotella loescheii
2207	Prevotella maculosa
2208	Prevotella melaninogenica
2209	Prevotella multisaccharivorax
2210	Prevotella nanceiensis
2211	Prevotella nigrescens
2212	Prevotella oulorum
2213	Prevotella pallens
2214	Prevotella paludivivens
2215	Prevotella pleuritidis
2216	Prevotella saccharolytica
2217	Prevotella salivae
2218	Prevotella scopos
2219	Prevotella shahii
2220	Prevotella sp.
2221	Prevotella stercorea
2222	Prevotella tannerae
2223	Prevotella timonensis
2224	Prevotella veroralis
2225	Procavia capensis
2226	Prochlorococcus marinus
2227	Prochlorococcus sp.
2228	Prochlorothrix hollandica
2229	Prolixibacter bellariivorans
2230	Promicromonospora sukumoe
2231	Propionibacteriaceae bacterium
2232	Propionibacterium acidifaciens
2233	Propionibacterium acidipropionici
2234	Propionibacterium acnes
2235	Propionibacterium avidum
2236	Propionibacterium jensenii
2237	Propionibacterium propionicum
2238	Propionibacterium sp.
2239	Propionicicella superfundia
2240	Propionimicrobium lymphophilum
2241	Prosthecochloris aestuarii
2242	Proteiniclasticum ruminis
2243	Proteiniphilum acetatigenes
2244	Proteobacteria bacterium
2245	Proteocatella sphenisci
2246	Proteus mirabilis
2247	Proteus penneri
2248	Providencia rettgeri
2249	Providencia rustigianii
2250	Providencia sneebia
2251	Prunus mume
2252	Pseudaminobacter salicylatoxidans
2253	Pseudanabaena sp.
2254	Pseudoalteromonas atlantica
2255	Pseudoalteromonas haloplanktis
2256	Pseudoalteromonas sp.
2257	Pseudobutyrivibrio ruminis
2258	Pseudobutyrivibrio sp.
2259	Pseudocercospora fijiensis
2260	Pseudochrobactrum sp.
2261	Pseudoclavibacter soli
2262	Pseudoduganella violaceinigra
2263	Pseudogulbenkiania ferrooxidans
2264	Pseudogulbenkiania sp.
2265	Pseudomonas aeruginosa
2266	Pseudomonas agarici
2267	Pseudomonas alcaligenes
2268	Pseudomonas azotifigens
2269	Pseudomonas caeni
2270	Pseudomonas chloritidismutans
2271	Pseudomonas chlororaphis
2272	Pseudomonas corrugata
2273	Pseudomonas cremoricolorata
2274	Pseudomonas denitrificans
2275	Pseudomonas entomophila
2276	Pseudomonas fluorescens
2277	Pseudomonas fragi
2278	Pseudomonas fulva
2279	Pseudomonas fuscovaginae
2280	Pseudomonas luteola
2281	Pseudomonas mandelii
2282	Pseudomonas mendocina
2283	Pseudomonas monteilii
2284	Pseudomonas mosselii
2285	Pseudomonas nitroreducens
2286	Pseudomonas oleovorans
2287	Pseudomonas parafulva
2288	Pseudomonas plecoglossicida
2289	Pseudomonas poae
2290	Pseudomonas psychrophila
2291	Pseudomonas putida
2292	Pseudomonas resinovorans
2293	Pseudomonas savastanoi
2294	Pseudomonas sp.
2295	Pseudomonas stutzeri
2296	Pseudomonas syringae
2297	Pseudomonas taeanensis
2298	Pseudomonas taiwanensis
2299	Pseudomonas thermotolerans
2300	Pseudomonas umsongensis
2301	Pseudomonas veronii
2302	Pseudomonas viridiflava
2303	Pseudomonas vranovensis
2304	Pseudonocardia acaciae
2305	Pseudonocardia asaccharolytica
2306	Pseudonocardia dioxanivorans
2307	Pseudonocardia sp.
2308	Pseudonocardia spinosispora
2309	Pseudopodoces humilis
2310	Pseudoramibacter alactolyticus
2311	Pseudorhodobacter ferrugineus
2312	Pseudovibrio sp.
2313	Pseudoxanthomonas sp.
2314	Pseudoxanthomonas spadix

2315	Pseudoxanthomonas suwonensis
2316	Pseudozyma flocculosa
2317	Psychrilyobacter atlanticus
2318	Psychrobacillus psychrodurans
2319	Psychrobacter arcticus
2320	Psychrobacter lutiphocae
2321	Psychrobacter sp.
2322	Psychroflexus torquis
2323	Psychroflexus tropicus
2324	Psychromonas aquimarina
2325	Psychromonas arctica
2326	Psychromonas hadalis
2327	Psychromonas ingrahamii
2328	Psychromonas ossibalaenae
2329	Pteropus alecto
2330	Puccinia graminis
2331	Pundamilia nyererei
2332	Pusillimonas noertemannii
2333	Pyrenophora tritici-repentis
2334	Pyrobaculum neutrophilum
2335	Pyrobaculum oguniense
2336	Pyrococcus sp.
2337	Pyrococcus yayanosii
2338	Python bivittatus
2339	Rahnella aquatilis
2340	Rahnella sp.
2341	Ralstonia eutropha
2342	Ralstonia pickettii
2343	Ralstonia solanacearum
2344	Ralstonia sp.
2345	Ramlibacter tataouinensis
2346	Rathayibacter toxicus
2347	Rattus norvegicus
2348	Reinekea sp.
2349	Rhizobiales bacterium
2350	Rhizobium etli
2351	Rhizobium giardinii
2352	Rhizobium larrymoorei
2353	Rhizobium leguminosarum
2354	Rhizobium leucaenae
2355	Rhizobium mesoamericanum
2356	Rhizobium mongolense
2357	Rhizobium phaseoli
2358	Rhizobium selenitireducens
2359	Rhizobium sp.
2360	Rhizobium sullae
2361	Rhizobium tropici
2362	Rhizobium undicola
2363	Rhodanobacter sp.
2364	Rhodobacter capsulatus
2365	Rhodobacter sp.
2366	Rhodobacter sphaeroides
2367	Rhodobacteraceae bacterium
2368	Rhodobacterales bacterium
2369	Rhodococcus defluvii
2370	Rhodococcus equi
2371	Rhodococcus erythropolis
2372	Rhodococcus jostii

2373	Rhodococcus opacus
2374	Rhodococcus phage
2375	Rhodococcus pyridinivorans
2376	Rhodococcus rhodochrous
2377	Rhodococcus ruber
2378	Rhodococcus sp.
2379	Rhodoferax ferrireducens
2380	Rhodoferax saidenbachensis
2381	Rhodomicrobium vannielii
2382	Rhodonellum psychrophilum
2383	Rhodopirellula baltica
2384	Rhodopseudomonas palustris
2385	Rhodopseudomonas sp.
2386	Rhodospirillales bacterium
2387	Rhodospirillum centenum
2388	Rhodospirillum photometricum
2389	Rhodospirillum rubrum
2390	Rhodothermus marinus
2391	Rhodovibrio salinarum
2392	Ricinus communis
2393	Rickettsia bellii
2394	Rickettsia felis
2395	Rickettsia montanensis
2396	Rickettsia prowazekii
2397	Rickettsia sibirica
2398	Rickettsia slovaca
2399	Riemerella anatipestifer
2400	Riemerella columbina
2401	Rikenella microfusus
2402	Rivularia sp.
2403	Robiginitalea biformata
2404	Robinsoniella sp.
2405	Romboutsia lituseburensis
2406	Roseburia faecis
2407	Roseburia hominis
2408	Roseburia sp.
2409	Roseiflexus castenholzii
2410	Roseiflexus sp.
2411	Roseobacter denitrificans
2412	Roseobacter sp.
2413	Roseomonas cervicalis
2414	Roseomonas gilardii
2415	Roseomonas sp.
2416	Roseovarius nubinhibens
2417	Roseovarius sp.
2418	Rothia mucilaginosa
2419	Ruania albidiflava
2420	Rubellimicrobium thermophilum
2421	Rubritalea marina
2422	Rubritepida flocculans
2423	Rubrivivax benzoatilyticus
2424	Rubrivivax gelatinosus
2425	Rubrobacter xylanophilus
2426	Rudaea cellulosilytica
2427	Ruegeria conchae
2428	Ruegeria pomeroyi
2429	Ruegeria sp.
2430	Ruminobacter sp.

2431	Ruminococcaceae bacterium
2432	Ruminococcus callidus
2433	Ruminococcus champanellensis
2434	Ruminococcus flavefaciens
2435	Ruminococcus gauvreauii
2436	Ruminococcus sp.
2437	Runella limosa
2438	Runella slithyformis
2439	Runella zeae
2440	Saccharibacillus kuerlensis
2441	Saccharomonospora azurea
2442	Saccharomonospora cyanea
2443	Saccharomonospora glauca
2444	Saccharomonospora marina
2445	Saccharomonospora paurometabolica
2446	Saccharomonospora saliphila
2447	Saccharomonospora sp.
2448	Saccharomonospora viridis
2449	Saccharomonospora xinjiangensis
2450	Saccharomyces cerevisiae
2451	Saccharophagus degradans
2452	Saccharopolyspora erythraea
2453	Saccharopolyspora rectivirgula
2454	Saccharopolyspora spinosa
2455	Saccharothrix espanaensis
2456	Saccoglossus kowalevskii
2457	Saimiri boliviensis
2458	Salinarchaeum sp.
2459	Salinarimonas rosea
2460	Salinibacter ruber
2461	Salinicoccus albus
2462	Salinicoccus carnicancri
2463	Salinicoccus luteus
2464	Salinimicrobium terrae
2465	Salinimicrobium xinjiangense
2466	Salinimonas chungwhensis
2467	Salinispora arenicola
2468	Salinispora pacifica
2469	Salinispora tropica
2470	Salinivibrio costicola
2471	Salinivibrio socompensis
2472	Salipiger mucosus
2473	Salisaeta longa
2474	Salmonella bongori
2475	Salmonella enterica
2476	Salpingoeca sp.
2477	Salsuginibacillus kocurii
2478	Sandarakinorhabdus limnophila
2479	Sandarakinorhabdus sp.
2480	Sanguibacter keddieii
2481	Saprolegnia diclina
2482	Saprospira grandis
2483	SAR324 cluster
2484	Sarcophilus harrisii
2485	Saxeibacter lacteus
2486	Scardovia wiggsiae
2487	Schizophyllum commune
2488	Schlesneria paludicola

2489	Sciscionella marina
2490	Sciscionella sp.
2491	Scytonema hofmanni
2492	Sebaldella termitidis
2493	Secondary endosymbiont
2494	Sedimenticola selenatireducens
2495	Sediminibacillus halophilus
2496	Sediminibacterium salmoneum
2497	Sediminibacterium sp.
2498	Segetibacter koreensis
2499	Segniliparus rotundus
2500	Segniliparus rugosus
2501	Selaginella moellendorffii
2502	Selenomonas artemidis
2503	Selenomonas bovis
2504	Selenomonas flueggei
2505	Selenomonas infelix
2506	Selenomonas noxia
2507	Selenomonas ruminantium
2508	Selenomonas sp.
2509	Selenomonas sputigena
2510	Senegalimassilia anaerobia
2511	Serinicoccus marinus
2512	Serinicoccus profundi
2513	Serinus canaria
2514	Serpula lacrymans
2515	Serratia fonticola
2516	Serratia liquefaciens
2517	Serratia marcescens
2518	Serratia odorifera
2519	Serratia plymuthica
2520	Serratia sp.
2521	Serratia symbiotica
2522	Setaria italica
2523	Setosphaeria turcica
2524	Shewanella amazonensis
2525	Shewanella baltica
2526	Shewanella colwelliana
2527	Shewanella fidelis
2528	Shewanella loihica
2529	Shewanella marina
2530	Shewanella oneidensis
2531	Shewanella putrefaciens
2532	Shewanella sediminis
2533	Shewanella sp.
2534	Shewanella violacea
2535	Shimazuella kribbensis
2536	Shinella sp.
2537	Shuttleworthia satelles
2538	Sideroxydans lithotrophicus
2539	Silanimonas lenta
2540	Silicibacter lacuscaerulensis
2541	Silicibacter sp.
2542	Simiduia agarivorans
2543	Simkania negevensis
2544	Simonsiella muelleri
2545	Simplicispira psychrophila
2546	Singularimonas variicoloris

2547	Singulisphaera acidiphila
2548	Sinobacter flavus
2549	Sinorhizobium arboris
2550	Sinorhizobium fredii
2551	Sinorhizobium meliloti
2552	Smaragdicoccus niigatensis
2553	Sneathiella glossodoripedis
2554	Sodalis glossinidius
2555	Soehngenia saccharolytica
2556	Solanum lycopersicum
2557	Solanum tuberosum
2558	Solibacillus silvestris
2559	Solimonas flava
2560	Solimonas soli
2561	Solirubrobacter soli
2562	Solirubrobacter sp.
2563	Solirubrobacterales bacterium
2564	Solitalea canadensis
2565	Solobacterium moorei
2566	Sorangium cellulosum
2567	Sorex araneus
2568	Sorghum bicolor
2569	Spermophilus tridecemlineatus
2570	Sphaerobacter thermophilus
2571	Sphaerochaeta pleomorpha
2572	Sphingobacterium sp.
2573	Sphingobacterium spiritivorum
2574	Sphingobium baderi
2575	Sphingobium chlorophenolicum
2576	Sphingobium japonicum
2577	Sphingobium quisquiliarum
2578	Sphingobium sp.
2579	Sphingobium ummariense
2580	Sphingobium xenophagum
2581	Sphingomonas astaxanthinifaciens
2582	Sphingomonas echinoides
2583	Sphingomonas elodea
2584	Sphingomonas melonis
2585	Sphingomonas phyllosphaerae
2586	Sphingomonas sp.
2587	Sphingomonas wittichii
2588	Sphingomonas-like bacterium
2589	Sphingopyxis alaskensis
2590	Spiribacter salinus
2591	Spiribacter sp.
2592	Spirillospora albida
2593	Spirochaeta africana
2594	Spirochaeta alkalica
2595	Spirochaeta bajacaliforniensis
2596	Spirochaeta caldaria
2597	Spirochaeta cellobiosiphila
2598	Spirochaeta coccoides
2599	Spirochaeta smaragdinae
2600	Spirochaeta sp.
2601	Spirochaeta thermophila
2602	Spiroplasma apis
2603	Spiroplasma chrysopicola
2604	Spiroplasma diminutum

2605	Spiroplasma syrphidicola
2606	Spirosoma luteum
2607	Spirosoma panaciterrae
2608	Spirosoma spitsbergense
2609	Spongiibacterium flavum
2610	Sporichthya polymorpha
2611	Sporobacter termitidis
2612	Sporocytophaga myxococcoides
2613	Sporolactobacillus inulinus
2614	Sporolactobacillus laevolacticus
2615	Sporolactobacillus terrae
2616	Sporolactobacillus vineae
2617	Sporomusa malonica
2618	Sporomusa ovata
2619	Sporosarcina newyorkensis
2620	Sporosarcina sp.
2621	Sporosarcina ureae
2622	Stackebrandtia nassauensis
2623	Stanieria cyanosphaera
2624	Staphylococcus aureus
2625	Staphylococcus capitis
2626	Staphylococcus caprae
2627	Staphylococcus carnosus
2628	Staphylococcus epidermidis
2629	Staphylococcus haemolyticus
2630	Staphylococcus hominis
2631	Staphylococcus intermedius
2632	Staphylococcus lentus
2633	Staphylococcus massiliensis
2634	Staphylococcus pasteuri
2635	Staphylococcus phage
2636	Staphylococcus pseudintermedius
2637	Staphylococcus saprophyticus
2638	Staphylococcus sciuri
2639	Staphylococcus simulans
2640	Staphylococcus sp.
2641	Staphylococcus vitulinus
2642	Staphylococcus warneri
2643	Staphylococcus xylosus
2644	Stappia stellulata
2645	Starkeya novella
2646	Stegastes partitus
2647	Stenotrophomonas maltophilia
2648	Stereum hirsutum
2649	Stigmatella aurantiaca
2650	strain 284/09
2651	Streptacidiphilus jeojiense
2652	Streptobacillus moniliformis
2653	Streptococcus agalactiae
2654	Streptococcus anginosus
2655	Streptococcus australis
2656	Streptococcus caballi
2657	Streptococcus castoreus
2658	Streptococcus constellatus
2659	Streptococcus cristatus
2660	Streptococcus devriesei
2661	Streptococcus didelphis
2662	Streptococcus equi

2663	Streptococcus equinus
2664	Streptococcus ferus
2665	Streptococcus gallolyticus
2666	Streptococcus gordonii
2667	Streptococcus henryi
2668	Streptococcus hyovaginalis
2669	Streptococcus infantarius
2670	Streptococcus infantis
2671	Streptococcus intermedius
2672	Streptococcus lutetiensis
2673	Streptococcus macedonicus
2674	Streptococcus marimammalium
2675	Streptococcus massiliensis
2676	Streptococcus merionis
2677	Streptococcus mitis
2678	Streptococcus mutans
2679	Streptococcus oligofermentans
2680	Streptococcus oralis
2681	Streptococcus orisratti
2682	Streptococcus ovis
2683	Streptococcus parauberis
2684	Streptococcus pasteurianus
2685	Streptococcus peroris
2686	Streptococcus pleomorphus
2687	Streptococcus plurextorum
2688	Streptococcus pneumoniae
2689	Streptococcus porci
2690	Streptococcus pseudopneumoniae
2691	Streptococcus pyogenes
2692	Streptococcus sanguinis
2693	Streptococcus sobrinus
2694	Streptococcus sp.
2695	Streptococcus suis
2696	Streptococcus thermophilus
2697	Streptococcus thoraltensis
2698	Streptococcus tigurinus
2699	Streptococcus uberis
2700	Streptococcus urinalis
2701	Streptomyces achromogenes
2702	Streptomyces acidiscabies
2703	Streptomyces afghaniensis
2704	Streptomyces albus
2705	Streptomyces auratus
2706	Streptomyces aureocirculatus
2707	Streptomyces avellaneus
2708	Streptomyces avermitilis
2709	Streptomyces bicolor
2710	Streptomyces bingchenggensis
2711	Streptomyces canus
2712	Streptomyces catenulae
2713	Streptomyces cattleya
2714	Streptomyces cellulosae
2715	Streptomyces clavuligerus
2716	Streptomyces collinus
2717	Streptomyces davawensis
2718	Streptomyces durhamensis
2719	Streptomyces exfoliatus
2720	Streptomyces flavidovirens

2721	Streptomyces flavochromogenes
2722	Streptomyces flavotricini
2723	Streptomyces flavovariabilis
2724	Streptomyces fulvissimus
2725	Streptomyces fulvoviolaceus
2726	Streptomyces globisporus
2727	Streptomyces griseoflavus
2728	Streptomyces griseoluteus
2729	Streptomyces griseus
2730	Streptomyces halstedii
2731	Streptomyces hygroscopicus
2732	Streptomyces katrae
2733	Streptomyces lavendulae
2734	Streptomyces lavenduligriseus
2735	Streptomyces leeuwenhoekii
2736	Streptomyces mediolani
2737	Streptomyces megasporus
2738	Streptomyces monomycini
2739	Streptomyces natalensis
2740	Streptomyces niveus
2741	Streptomyces ochraceiscleroticus
2742	Streptomyces olivaceus
2743	Streptomyces peruviensis
2744	Streptomyces peucetius
2745	Streptomyces pristinaespiralis
2746	Streptomyces prunicolor
2747	Streptomyces purpeofuscus
2748	Streptomyces purpureus
2749	Streptomyces rapamycinicus
2750	Streptomyces resistomycificus
2751	Streptomyces rimosus
2752	Streptomyces roseochromogenus
2753	Streptomyces roseosporus
2754	Streptomyces roseoverticillatus
2755	Streptomyces ruber
2756	Streptomyces scabiei
2757	Streptomyces scabrisporus
2758	Streptomyces sclerotialus
2759	Streptomyces scopuliridis
2760	Streptomyces seoulensis
2761	Streptomyces sp.
2762	Streptomyces sulphureus
2763	Streptomyces sviceus
2764	Streptomyces thermolilacinus
2765	Streptomyces varsoviensis
2766	Streptomyces venezuelae
2767	Streptomyces violaceoruber
2768	Streptomyces violaceusniger
2769	Streptomyces violens
2770	Streptomyces virginiae
2771	Streptomyces viridochromogenes
2772	Streptomyces vitaminophilus
2773	Streptomyces xanthophaeus
2774	Streptomycetaceae bacterium
2775	Streptosporangium amethystogenes
2776	Streptosporangium roseum
2777	Strongylocentrotus purpuratus
2778	Subdoligranulum sp.

2779	Succinatimonas hippei
2780	Succinatimonas sp.
2781	Succinimonas amylolytica
2782	Succinispira mobilis
2783	Succinivibrio dextrinosolvens
2784	Succinivibrionaceae bacterium
2785	Sulfitobacter guttiformis
2786	Sulfitobacter sp.
2787	Sulfobacillus acidophilus
2788	Sulfobacillus thermosulfidooxidans
2789	Sulfolobus acidocaldarius
2790	Sulfuricella denitrificans
2791	Sulfurihydrogenibium azorense
2792	Sulfurimonas autotrophica
2793	Sulfurospirillum arcachonense
2794	Sus scrofa
2795	Sutterella parvirubra
2796	Sutterella sp.
2797	Sutterella wadsworthensis
2798	Symbiobacterium thermophilum
2799	Syncytium symbiont
2800	Synechococcus elongatus
2801	Synechococcus sp.
2802	Synechocystis sp.
2803	Synergistes sp.
2804	Synergistetes bacterium
2805	Syntrophobacter fumaroxidans
2806	Syntrophobotulus glycolicus
2807	Syntrophococcus sucromutans
2808	Syntrophomonas erecta
2809	Syntrophomonas wolfei
2810	Syntrophorhabdus aromaticivorans
2811	Syntrophothermus lipocalidus
2812	Syntrophus aciditrophicus
2813	Taeniopygia guttata
2814	Takifugu rubripes
2815	Tannerella forsythia
2816	Tannerella sp.
2817	Tarsius syrichta
2818	Tatumella ptyseos
2819	Tatumella sp.
2820	Taylorella equigenitalis
2821	Tenacibaculum ovolyticum
2822	Tenacibaculum sp.
2823	Tepidanaerobacter acetatoxydans
2824	Tepidiphilus margaritifer
2825	Terasakiella pusilla
2826	Teredinibacter turnerae
2827	Terracoccus sp.
2828	Terriglobus roseus
2829	Terriglobus saanensis
2830	Terrimonas ferruginea
2831	Terrisporobacter glycolicus
2832	Tetragenococcus halophilus
2833	Tetragenococcus muriaticus
2834	Tetrahymena thermophila
2835	Tetrapisispora phaffii
2836	Thalassobacillus devorans

2837	Thalassobacter arenae
2838	Thalassobaculum salexigens
2839	Thalassolituus oleivorans
2840	Thalassospira lucentensis
2841	Thauera linaloolentis
2842	Thauera sp.
2843	Thauera terpenica
2844	Theobroma cacao
2845	Thermacetogenium phaeum
2846	Thermaerobacter marianensis
2847	Thermaerobacter subterraneus
2848	Thermanaerovibrio acidaminovorans
2849	Thermanaerovibrio velox
2850	Thermicanus aegyptius
2851	Thermincola potens
2852	Thermithiobacillus tepidarius
2853	Thermoanaerobacter brockii
2854	Thermoanaerobacter italicus
2855	Thermoanaerobacter mathranii
2856	Thermoanaerobacter sp.
2857	Thermoanaerobacter thermocopriae
2858	Thermoanaerobacterium saccharolyticum
2859	Thermoanaerobacterium thermosaccharolyticum
2860	Thermoanaerobacterium xylanolyticum
2861	Thermobacillus composti
2862	Thermobaculum terrenum
2863	Thermobispora bispora
2864	Thermobrachium celere
2865	Thermococcus gammatolerans
2866	Thermococcus kodakarensis
2867	Thermococcus sibiricus
2868	Thermococcus sp.
2869	Thermocrispum agreste
2870	Thermocrispum municipale
2871	Thermodesulfatator atlanticus
2872	Thermodesulfobacterium hveragerdense
2873	Thermodesulfovibrio thiophilus
2874	Thermodesulfovibrio yellowstonii
2875	Thermoflavimicrobium dichotomicum
2876	Thermomicrobium roseum
2877	Thermomonospora curvata
2878	Thermophagus xiamenensis
2879	Thermoplasmatales archaeon
2880	Thermosediminibacter oceani
2881	Thermosipho africanus
2882	Thermosipho melanesiensis
2883	Thermosynechococcus elongatus
2884	Thermosynechococcus sp.
2885	Thermotoga hypogea
2886	Thermotoga lettingae
2887	Thermotoga maritima
2888	Thermotoga neapolitana
2889	Thermus igniterrae
2890	Thermus oshimai
2891	Thermus scotoductus
2892	Thermus sp.
2893	Thermus thermophilus

2894	Thielavia terrestris
2895	Thioalkalivibrio nitratireducens
2896	Thioalkalivibrio sp.
2897	Thioalkalivibrio sulfidophilus
2898	Thioalkalivibrio thiocyanoxidans
2899	Thiobacillus denitrificans
2900	Thiobacillus thioparus
2901	Thiocystis violascens
2902	Thioflavicoccus mobilis
2903	Thiomicrospira arctica
2904	Thiomicrospira chilensis
2905	Thiomicrospira pelophila
2906	Thiomicrospira sp.
2907	Thiomonas intermedia
2908	Thiomonas sp.
2909	Thiorhodovibrio sp.
2910	Thiothrix disciformis
2911	Thiothrix lacustris
2912	Tistrella mobilis
2913	Togninia minima
2914	Tolumonas auensis
2915	Tolumonas sp.
2916	Tomitella biformata
2917	Trametes versicolor
2918	Trebouxiophyceae sp.
2919	Tremella mesenterica
2920	Treponema azotonutricium
2921	Treponema brennaborense
2922	Treponema bryantii
2923	Treponema denticola
2924	Treponema lecithinolyticum
2925	Treponema maltophilum
2926	Treponema medium
2927	Treponema pallidum
2928	Treponema pedis
2929	Treponema phagedenis
2930	Treponema primitia
2931	Treponema socranskii
2932	Treponema sp.
2933	Treponema succinifaciens
2934	Treponema vincentii
2935	Tribolium castaneum
2936	Trichechus manatus
2937	Trichoderma reesei
2938	Trichodesmium erythraeum
2939	Trichomonas vaginalis
2940	Tropheryma whipplei
2941	Truepera radiovictrix
2942	Trueperella pyogenes
2943	Trypanosoma cruzi
2944	Tsukamurella paurometabola
2945	Tuber melanosporum
2946	Tuberibacillus calidus
2947	Tupaia chinensis
2948	Turicella otitidis
2949	Turneriella parva
2950	Tursiops truncatus
2951	Uliginosibacterium gangwonense

2952	uncultured archaeon
2953	Uncultured bacterium
2954	Ureibacillus thermosphaericus
2955	Ursus maritimus
2956	Vanderwaltozyma polyspora
2957	Varibaculum cambriense
2958	Variovorax paradoxus
2959	Variovorax sp.
2960	Verminephrobacter aporrectodeae
2961	Verminephrobacter eiseniae
2962	Verrucomicrobia bacterium
2963	Verrucomicrobiae bacterium
2964	Verrucomicrobium spinosum
2965	Verrucosispora maris
2966	Verticillium albo-atrum
2967	Vibrio alginolyticus
2968	Vibrio angustum
2969	Vibrio breoganii
2970	Vibrio campbellii
2971	Vibrio cholerae
2972	Vibrio coralliilyticus
2973	Vibrio crassostreae
2974	Vibrio fischeri
2975	Vibrio furnissii
2976	Vibrio gazogenes
2977	Vibrio genomosp.
2978	Vibrio harveyi
2979	Vibrio kanaloae
2980	Vibrio nigripulchritudo
2981	Vibrio ordalii
2982	Vibrio parahaemolyticus
2983	Vibrio splendidus
2984	Vibrio tasmaniensis
2985	Vibrio vulnificus
2986	Vicugna pacos
2987	Virgibacillus alimentarius
2988	Virgibacillus halodenitrificans
2989	Virgibacillus sp.
2990	Vitis vinifera
2991	Vitreoscilla stercoraria
2992	Volvox carteri
2993	Wallemia sebi
2994	Weissella koreensis
2995	Weissella viridescens
2996	Wenxinia marina
2997	Wigglesworthia glossinidia
2998	Williamsia sp.
2999	Wolbachia endosymbiont
3000	Wolinella succinogenes
3001	Woodsholea maritima
3002	Xanthobacter autotrophicus
3003	Xanthobacter sp.
3004	Xanthobacteraceae bacterium
3005	Xanthomonadaceae bacterium
3006	Xanthomonas albilineans
3007	Xanthomonas arboricola
3008	Xanthomonas axonopodis
3009	Xanthomonas campestris

3010	Xanthomonas cassavae
3011	Xanthomonas citri
3012	Xanthomonas hortorum
3013	Xanthomonas sacchari
3014	Xanthomonas sp.
3015	Xanthomonas translucens
3016	Xanthomonas vasicola
3017	Xenopus (Silurana)
3018	Xiphophorus maculatus
3019	Xylanimonas cellulosilytica
3020	Xylella fastidiosa
3021	Yaniella halotolerans
3022	Yarrowia lipolytica
3023	Yersinia pestis
3024	Yokenella regensburgei
3025	Zea mays
3026	Zeta proteobacterium
3027	Zobellia galactanivorans
3028	Zonotrichia albicollis
3029	Zooshikella ganghwensis
3030	Zymomonas mobilis
3031	Zymophilus raffinosivorans

Table S28 : The taxonomic profile predicted from the assembly results of MetaVelvet-SL using BLAST for the MH0012 dataset.

No.	Species
1	Acidaminococcus fermentans
2	Actinomyces odontolyticus
3	Akkermansia muciniphila
4	Alistipes putredinis
5	Alistipes shahii
6	Anaerococcus unclassified
7	Anaerostipes caccae
8	Anaerotruncus colihominis
9	Bacteroides caccae
10	Bacteroides cellulosilyticus
11	Bacteroides coprocola
12	Bacteroides coprophilus
13	Bacteroides dorei
14	Bacteroides eggerthii
15	Bacteroides finegoldii
16	Bacteroides fragilis
17	Bacteroides helcogenes
18	Bacteroides intestinalis
19	Bacteroides pectinophilus
20	Bacteroides plebeius
21	Bacteroides salanitronis
22	Bacteroides stercoris
23	Bacteroides thetaiotaomicron
24	Bacteroides unclassified
25	Bacteroides uniformis
26	Bacteroides vulgatus
27	Bacteroides xylanisolvens
28	Bifidobacterium adolescentis
29	Bifidobacterium angulatum
30	Bifidobacterium bifidum
31	Bifidobacterium breve
32	Bifidobacterium dentium
33	Bifidobacterium longum
34	Bifidobacterium pseudocatenulatum
35	Bilophila wadsworthia
36	Blautia hansenii
37	Blautia unclassified
38	Burkholderia unclassified
39	Butyrivibrio crossotus
40	Butyrivibrio unclassified
41	Catenibacterium mitsuokai
42	Citrobacter unclassified
43	Clostridium asparagiforme
44	Clostridium bartlettii
45	Clostridium bolteae
46	Clostridium cf
47	Clostridium hathewayi
48	Clostridium leptum
49	Clostridium methylpentosum
50	Clostridium nexile
51	Clostridium scindens
52	Clostridium symbiosum
53	Collinsella stercoris
54	Coprococcus catus
55	Coprococcus comes
56	Deinococcus unclassified
57	Desulfovibrio desulfuricans
58	Dorea formicigenerans
59	Dorea longicatena
60	Eggerthella lenta
61	Enterococcus unclassified
62	Escherichia coli
63	Ethanoligenens harbinense
64	Eubacterium eligens
65	Eubacterium hallii
66	Eubacterium rectale
67	Eubacterium siraeum
68	Eubacterium ventriosum
69	Faecalibacterium cf
70	Faecalibacterium prausnitzii
71	Faecalibacterium unclassified
72	Gordonibacter pamelaeae
73	Haemophilus parainfluenzae
74	Holdemania filiformis
75	Lactobacillus crispatus
76	Lactococcus lactis
77	Leuconostoc mesenteroides
78	Megasphaera micronuciformis
79	Methanobrevibacter smithii
80	Methanobrevibacter unclassified
81	Mitsuokella multacida
82	Mycobacterium unclassified
83	Odoribacter splanchnicus
84	Oxalobacter formigenes
85	Parabacteroides distasonis
86	Parabacteroides johnsonii
87	Parabacteroides merdae
88	Parabacteroides unclassified
89	Peptoniphilus unclassified
90	Phascolarctobacterium unclassified
91	Porphyromonas asaccharolytica
92	Porphyromonas uenonis
93	Prevotella bivia
94	Prevotella buccalis
95	Prevotella copri
96	Prevotella melaninogenica
97	Prevotella multiformis
98	Prevotella timonensis
99	Pseudoflavonifractor capillosus
100	Pseudomonas unclassified
101	Roseburia intestinalis
102	Roseburia inulinivorans
103	Rothia mucilaginosa
104	Rothia unclassified
105	Ruminococcus albus
106	Ruminococcus bromii
107	Ruminococcus gnavus
108	Ruminococcus lactaris
109	Ruminococcus obeum
110	Ruminococcus torques

111	Selenomonas sputigena
112	Selenomonas unclassified
113	Slackia unclassified
114	Solobacterium moorei
115	Streptococcus parasanguinis
116	Streptococcus salivarius
117	Subdoligranulum variabile
118	Succinatimonas hippei
119	Sutterella wadsworthensis
120	Veillonella atypica
121	Veillonella dispar
122	Veillonella parvula
123	Veillonella unclassified
124	Victivallis vadensis
125	[Bacillus] schlegelii
126	[Clostridium] aerotolerans
127	[Clostridium] aldrichii
128	[Clostridium] algidixylanolyticum
129	[Clostridium] alkalicellulosi
130	[Clostridium] aminophilum
131	[Clostridium] bifermentans
132	[Clostridium] cellobioparum
133	[Clostridium] cellulosi
134	[Clostridium] clostridioforme
135	[Clostridium] glycyrrhizinilyticum
136	[Clostridium] herbivorans
137	[Clostridium] hungatei
138	[Clostridium] hylemonae
139	[Clostridium] indolis
140	[Clostridium] josui
141	[Clostridium] lactatifermentans
142	[Clostridium] lavalense
143	[Clostridium] mangenotii
144	[Clostridium] methoxybenzovorans
145	[Clostridium] papyrosolvens
146	[Clostridium] propionicum
147	[Clostridium] saccharogumia
148	[Clostridium] sordellii
149	[Clostridium] sporosphaeroides
150	[Clostridium] sticklandii,
151	[Clostridium] straminisolvens
152	[Clostridium] sufflavum
153	[Clostridium] termitidis
154	[Clostridium] thermosuccinogenes
155	[Clostridium] viride
156	[Clostridium] xylanolyticum
157	[Eubacterium] cellulosolvens
158	[Eubacterium] hallii
159	[Eubacterium] sulci
160	Abiotrophia defectiva
161	Acanthamoeba castellanii
162	Acanthamoeba polyphaga
163	Acanthisitta chloris
164	Acaricomes phytoseiuli
165	Acaryochloris marina
166	Acaryochloris sp,
167	Acetivibrio cellulolyticus
168	Acetivibrio ethanolgignens

169	Acetobacter aceti
170	Acetobacter nitrogenifigens
171	Acetobacter pasteurianus
172	Acetobacter sp,
173	Acetobacteraceae bacterium
174	Acetobacterium dehalogenans
175	Acetobacterium woodii
176	Acetohalobium arabaticum
177	Acholeplasma axanthum
178	Acholeplasma equifetale
179	Acholeplasma granularum
180	Acholeplasma laidlawii
181	Acholeplasma modicum
182	Acholeplasma multilocale
183	Acholeplasma sp,
184	Achromobacter piechaudii
185	Achromobacter xylosoxidans
186	Acidaminococcus intestini
187	Acidaminococcus sp,
188	Acidimicrobidae bacterium
189	Acidiphilium angustum
190	Acidiphilium cryptum
191	Acidiphilium multivorum
192	Acidiphilium sp,
193	Acidithiobacillus caldus
194	Acidithiobacillus ferrivorans
195	Acidithiobacillus ferrooxidans
196	Acidithiobacillus thiooxidans
197	Acidobacteria bacterium
198	Acidobacteriaceae bacterium
199	Acidobacterium capsulatum
200	Acidobacterium sp,
201	Acidocella facilis
202	Acidocella sp,
203	Acidomonas methanolica
204	Acidothermus cellulolyticus
205	Acidovorax avenae
206	Acidovorax citrulli
207	Acidovorax ebreus
208	Acidovorax oryzae
209	Acidovorax radicis
210	Acidovorax sp,
211	Aciduliprofundum sp,
212	Acinetobacter baumannii
213	Acinetobacter beijerinckii
214	Acinetobacter bereziniae
215	Acinetobacter bouvetii
216	Acinetobacter brisouii
217	Acinetobacter calcoaceticus
218	Acinetobacter gerneri
219	Acinetobacter gyllenbergii
220	Acinetobacter indicus
221	Acinetobacter johnsonii
222	Acinetobacter junii
223	Acinetobacter lwoffii
224	Acinetobacter nectaris
225	Acinetobacter nosocomialis
226	Acinetobacter pittii

227	Acinetobacter radioresistens
228	Acinetobacter soli
229	Acinetobacter sp,
230	Acinetobacter towneri
231	Acinetobacter ursingii
232	Actinoalloteichus cyanogriseus
233	Actinoalloteichus spitiensis
234	Actinobacillus pleuropneumoniae
235	Actinobacillus succinogenes
236	Actinobacterium LLX17
237	Actinobacterium SCGC
238	Actinobaculum massiliae
239	Actinobaculum schaalii
240	Actinobaculum sp,
241	Actinobaculum urinale
242	Actinocatenispora sera
243	Actinokineospora enzanensis
244	Actinokineospora inagensis
245	Actinomadura atramentaria
246	Actinomadura flavalba
247	Actinomadura madurae
248	Actinomadura oligospora
249	Actinomadura rifamycini
250	Actinomyces cardiffensis
251	Actinomyces dentalis
252	Actinomyces gerencseriae
253	Actinomyces graevenitzii
254	Actinomyces johnsonii
255	Actinomyces massiliensis
256	Actinomyces neuii
257	Actinomyces oris
258	Actinomyces slackii
259	Actinomyces sp,
260	Actinomyces suimastitidis
261	Actinomyces timonensis
262	Actinomyces turicensis
263	Actinomyces urogenitalis
264	Actinomyces vaccimaxillae
265	Actinomyces viscosus
266	Actinomycetospora chiangmaiensis
267	Actinoplanes friuliensis
268	Actinoplanes globisporus
269	Actinoplanes missouriensis
270	Actinoplanes sp,
271	Actinoplanes subtropicus
272	Actinopolymorpha alba
273	Actinopolyspora halophila
274	Actinopolyspora mortivallis
275	Actinosynnema mirum
276	Acyrthosiphon pisum
277	Adlercreutzia equolifaciens
278	Advenella kashmirensis
279	Aedes aegypti
280	Aequorivita sublithincola
281	Aeromonas caviae
282	Aeromonas enteropelogenes
283	Aeromonas hydrophila
284	Aeromonas salmonicida

285	Aeromonas sp,
286	Aeromonas veronii
287	Aerophobetes bacterium
288	Aestuariibacter salexigens
289	Aestuariimicrobium kwangyangense
290	Afifella pfennigii
291	Afipia broomeae
292	Afipia clevelandensis
293	Afipia felis
294	Afipia sp,
295	Aggregatibacter actinomycetemcomitans
296	Aggregatibacter segnis
297	Agrobacterium fabrum
298	Agrobacterium radiobacter
299	Agrobacterium sp,
300	Agrobacterium tumefaciens
301	Agrobacterium vitis
302	Agrococcus lahaulensis
303	Agrococcus pavilionensis
304	Agromonas oligotrophica
305	Agromyces italicus
306	Agromyces subbeticus
307	Ahrensia kielensis
308	Ahrensia sp,
309	Ailuropoda melanoleuca
310	Ajellomyces capsulatus
311	Akkermansia sp,
312	Alcaligenes sp,
313	Alcanivorax borkumensis
314	Alcanivorax dieselolei
315	Alcanivorax sp,
316	Algoriphagus marincola
317	Algoriphagus sp,
318	Aliagarivorans marinus
319	Alicycliphilus denitrificans
320	Alicyclobacillus acidocaldarius
321	Alicyclobacillus acidoterrestris
322	Alicyclobacillus contaminans
323	Alicyclobacillus herbarius
324	Alicyclobacillus macrosporangiidus
325	Alicyclobacillus pohliae
326	Alicyclobacillus pomorum
327	Aliihoeflea sp,
328	Aliivibrio fischeri
329	Aliivibrio salmonicida
330	Alistipes finegoldii
331	Alistipes indistinctus
332	Alistipes onderdonkii
333	Alistipes senegalensis
334	Alistipes sp,
335	Alistipes timonensis
336	Alkalibacillus haloalkaliphilus
337	Alkalibacter saccharofermentans
338	Alkalibaculum bacchi
339	Alkaliflexus imshenetskii
340	Alkalilimnicola ehrlichii
341	Alkaliphilus crotonatoxidans
342	Alkaliphilus metalliredigens

343	Alkaliphilus oremlandii
344	Alkaliphilus transvaalensis
345	Alkanindiges illinoisensis
346	Alligator mississippiensis
347	Alligator sinensis
348	Allisonella histaminiformans
349	Allobaculum stercoricanis
350	Allochromatium vinosum
351	Allofustis seminis
352	Allokutzneria albata
353	Alloprevotella rava
354	Alloscardovia omnicolens
355	Alpha proteobacterium
356	alpha proteobacterium
357	Alteromonas macleodii
358	Amborella trichopoda
359	Aminicenantes bacterium
360	Aminiphilus circumscriptus
361	Aminobacter sp,
362	Aminobacterium colombiense
363	Aminobacterium mobile
364	Aminomonas paucivorans
365	Ammonifex degensii
366	Amorphus coralli
367	Amphibacillus jilinensis
368	Amphibacillus xylanus
369	Amphritea japonica
370	Amycolatopsis alba
371	Amycolatopsis balhimycina
372	Amycolatopsis benzoatilytica
373	Amycolatopsis mediterranei
374	Amycolatopsis nigrescens
375	Amycolatopsis orientalis
376	Amycolatopsis sp,
377	Amycolatopsis taiwanensis
378	Anaeroarcus burkinensis
379	Anaerobacterium chartisolvens
380	Anaerobaculum hydrogeniformans
381	Anaerobaculum mobile
382	Anaerobiospirillum succiniciproducens
383	Anaerobranca horikoshii
384	Anaerofilum agile
385	Anaerofilum pentosovorans
386	Anaerofustis stercorihominis
387	Anaeroglobus geminatus
388	Anaerolinea thermophila
389	Anaeromusa acidaminophila
390	Anaeromyxobacter dehalogenans
391	Anaeromyxobacter sp,
392	Anaerophaga thermohalophila
393	Anaerorhabdus furcosa
394	Anaerosporobacter mobilis
395	Anaerostipes hadrus
396	Anaerostipes sp,
397	Anaerotruncus sp,
398	Anaerovibrio lipolyticus
399	Anaerovibrio sp,
400	Anaerovorax odorimutans

401	Anas platyrhynchos
402	Aneurinibacillus aneurinilyticus
403	Aneurinibacillus terranovensis
404	Anolis carolinensis
405	Anopheles gambiae
406	Anoxybacillus flavithermus
407	Anoxybacillus kamchatkensis
408	Anoxybacillus tengchongensis
409	Anoxybacillus tepidamans
410	Aotus nancymaae
411	Apis dorsata
412	Apis florea
413	Apis mellifera
414	Aplysia californica
415	Aptenodytes forsteri
416	Aquimarina agarilytica
417	Aquimarina latercula
418	Aquimarina sp,
419	Arabidopsis thaliana
420	Archaeoglobus fulgidus
421	Archaeoglobus veneficus
422	Arcobacter butzleri
423	Arcobacter cibarius
424	Arcobacter sp,
425	Arenibacter algicola
426	Arenimonas composti
427	Arenimonas oryziterrae
428	Arenitalea lutea
429	Arhodomonas aquaeolei
430	Aromatoleum aromaticum
431	Arsenicicoccus bolidensis
432	Arthrobacter arilaitensis
433	Arthrobacter castelli
434	Arthrobacter nicotinovorans
435	Arthrobacter phenanthrenivorans
436	Arthrobacter sanguinis
437	Arthrobacter sp,
438	Arthroderma otae
439	Arthrospira platensis
440	Asaia astilbes
441	Asaia platycodi
442	Asaia prunellae
443	Asaia sp,
444	Ashbya gossypii
445	Aspergillus clavatus
446	Aspergillus niger
447	Aspergillus terreus
448	Asticcacaulis benevestitus
449	Asticcacaulis biprosthecum
450	Asticcacaulis excentricus
451	Asticcacaulis sp,
452	Astyanax mexicanus
453	Atopobium minutum
454	Atopobium parvulum
455	Atopobium sp,
456	Atopobium vaginae
457	Atopococcus tabaci
458	Atribacteria bacterium

459	Aurantimonas coralicida
460	Aurantimonas manganoxydans
461	Aureimonas ureilytica
462	Aureococcus anophagefferens
463	Auricularia delicata
464	Azoarcus sp,
465	Azoarcus toluclasticus
466	Azohydromonas australica
467	Azonexus hydrophilus
468	Azorhizobium caulinodans
469	Azorhizobium doebereinerae
470	Azospirillum brasilense
471	Azospirillum halopraeferens
472	Azospirillum lipoferum
473	Azospirillum sp,
474	Azotobacter vinelandii
475	Azovibrio restrictus
476	Bacillus acidiproducens
477	Bacillus aidingensis
478	Bacillus amyloliquefaciens
479	Bacillus anthracis
480	Bacillus atrophaeus
481	Bacillus aurantiacus
482	Bacillus bogoriensis
483	Bacillus cellulosilyticus
484	Bacillus cereus
485	Bacillus chagannorensis
486	Bacillus clausii
487	Bacillus coagulans
488	Bacillus cytotoxicus
489	Bacillus endophyticus
490	Bacillus flexus
491	Bacillus fordii
492	Bacillus ginsengihumi
493	Bacillus halodurans
494	Bacillus infantis
495	Bacillus kribbensis
496	Bacillus licheniformis
497	Bacillus mannanilyticus
498	Bacillus marmarensis
499	Bacillus massilioanorexius
500	Bacillus massiliosenegalensis
501	Bacillus megaterium
502	Bacillus mojavensis
503	Bacillus oceanisediminis
504	Bacillus panaciterrae
505	Bacillus phage
506	Bacillus pseudofirmus
507	Bacillus pseudomycoides
508	Bacillus pumilus
509	Bacillus selenitireducens
510	Bacillus siamensis
511	Bacillus smithii
512	Bacillus sonorensis
513	Bacillus sp,
514	Bacillus subtilis
515	Bacillus tequilensis
516	Bacillus thuringiensis

517	Bacillus timonensis
518	Bacillus weihenstephanensis
519	Bacteriovorax marinus
520	Bacterium JKG1
521	Bacteroidales bacterium
522	Bacteroidetes bacterium
523	Bacteroidetes oral
524	Balaenoptera acutorostrata
525	Balneatrix alpica
526	Balneimonas flocculans
527	Balneola vulgaris
528	Barnesiella intestinihominis
529	Bartonella australis
530	Bartonella elizabethae
531	Bartonella quintana
532	Bartonella schoenbuchensis
533	Bartonella tamiae
534	Bartonella tribocorum
535	Bavariicoccus seileri
536	Bdellovibrio bacteriovorus
537	Bdellovibrio exovorus
538	Beauveria bassiana
539	Beggiatoa alba
540	Belliella baltica
541	Beta proteobacterium
542	Beutenbergia cavernae
543	Bifidobacterium animalis
544	Bifidobacterium asteroides
545	Bifidobacterium boum
546	Bifidobacterium choerinum
547	Bifidobacterium magnum
548	Bifidobacterium minimum
549	Bifidobacterium pseudolongum
550	Bifidobacterium ruminantium
551	Bifidobacterium sp,
552	Bifidobacterium subtile
553	Bifidobacterium thermacidophilum
554	Bifidobacterium thermophilum
555	Bifidobacterium tsurumiense
556	Bilophila sp,
557	Bipolaris oryzae
558	Blastocatella fastidiosa
559	Blastococcus saxobsidens
560	Blastococcus sp,
561	Blastomonas sp,
562	Blastopirellula marina
563	Bombus impatiens
564	Bombus terrestris
565	Bombyx mori
566	Bordetella avium
567	Bordetella bronchiseptica
568	Bordetella hinzii
569	Bordetella holmesii
570	Bordetella petrii
571	Bordetella sp,
572	Bordetella trematum
573	Borrelia afzelii
574	Borrelia miyamotoi

575	Bos mutus
576	Bos taurus
577	Bosea sp,
578	Brachybacterium faecium
579	Brachybacterium muris
580	Brachybacterium paraconglomeratum
581	Brachybacterium squillarum
582	Brachymonas chironomi
583	Brachypodium distachyon
584	Brachyspira alvinipulli
585	Brachyspira hyodysenteriae
586	Brachyspira intermedia
587	Brachyspira murdochii
588	Brachyspira pilosicoli
589	Brachyspira sp,
590	Brackiella oedipodis
591	Bradyrhizobiaceae bacterium
592	Bradyrhizobium elkanii
593	Bradyrhizobium genosp,
594	Bradyrhizobium japonicum
595	Bradyrhizobium sp,
596	Branchiostoma floridae
597	Brassica rapa
598	BRC1 bacterium
599	Brenneria sp,
600	Brevibacillus agri
601	Brevibacillus borstelensis
602	Brevibacillus brevis
603	Brevibacillus laterosporus
604	Brevibacillus massiliensis,
605	Brevibacillus panacihumi
606	Brevibacillus thermoruber
607	Brevibacterium album
608	Brevibacterium linens
609	Brevibacterium massiliense
610	Brevibacterium senegalense,
611	Brevibacterium sp,
612	Brevundimonas bacteroides
613	Brevundimonas diminuta
614	Brevundimonas naejangsanensis
615	Brevundimonas sp,
616	Brevundimonas subvibrioides
617	Brochothrix phage
618	Brucella abortus
619	Brucella ceti
620	Brucella inopinata
621	Brucella sp,
622	Brugia malayi
623	Bryobacter aggregatus
624	Bubalus bubalis
625	Buchnera aphidicola
626	Burkholderiales bacterium
627	Butyrate-producing bacterium
628	Butyricicoccus pullicaecorum
629	Butyricimonas synergistica
630	Butyricimonas virosa
631	Caenorhabditis briggsae
632	Caenorhabditis elegans

633	Caenorhabditis remanei
634	Caldanaerobius polysaccharolyticus
635	Caldibacillus debilis
636	Caldicellulosiruptor acetigenus
637	Caldicellulosiruptor hydrothermalis
638	Caldicellulosiruptor kristjanssonii
639	Caldicellulosiruptor kronotskyensis
640	Caldicellulosiruptor lactoaceticus
641	Caldicellulosiruptor obsidiansis
642	Caldicellulosiruptor owensensis
643	Caldicellulosiruptor saccharolyticus
644	Caldicoprobacter guelmensis
645	Caldicoprobacter oshimai
646	Caldilinea aerophila
647	Caldimonas manganoxidans
648	Caldithrix abyssi
649	Callithrix jacchus
650	Callorhinchus milii
651	Caloramator boliviensis
652	Caloramator sp,
653	Caloranaerobacter azorensis
654	Calothrix sp,
655	Calypte anna
656	Camelus ferus
657	Campylobacter concisus
658	Campylobacter cuniculorum
659	Campylobacter curvus
660	Campylobacter fetus
661	Campylobacter jejuni
662	Campylobacter lari
663	Campylobacter sp,
664	Campylobacter ureolyticus
665	Campylobacterales bacterium
666	Candida dubliniensis
667	Candida glabrata
668	Candida orthopsilosis
669	Candida tropicalis
670	Candidate division
671	Candidatus Accumulibacter
672	Candidatus Alistipes
673	Candidatus Ancillula
674	Candidatus Arthromitus
675	Candidatus Caldatribacterium
676	Candidatus Chloracidobacterium
677	Candidatus Cloacamonas
678	Candidatus Desulforudis
679	Candidatus Endolissoclinum
680	Candidatus Hamiltonella
681	Candidatus Hydrogenedens
682	Candidatus Koribacter
683	Candidatus Liberibacter
684	Candidatus Methanomethylophilus
685	Candidatus Methylomirabilis
686	Candidatus Microthrix
687	Candidatus Nitrosopumilus
688	Candidatus Nitrospira
689	Candidatus Omnitrophus
690	Candidatus Pelagibacter

691	Candidatus Phytoplasma
692	Candidatus Poribacteria
693	Candidatus Protochlamydia
694	Candidatus Puniceispirillum
695	Candidatus Regiella
696	Candidatus Ruthia
697	Candidatus Saccharibacteria
698	Candidatus Saccharimonas
699	Candidatus Schmidhempelia
700	Candidatus Solibacter
701	Candidatus Stoquefichus
702	Candidatus Sulcia
703	Canis lupus
704	Capnocytophaga cynodegmi
705	Capnocytophaga granulosa
706	Capnocytophaga ochracea
707	Capnocytophaga sp,
708	Capra hircus
709	Capsella rubella
710	Carboxydibrachium pacificum
711	Carboxydocella manganica
712	Carboxydothermus ferrireducens
713	Carboxydothermus hydrogenoformans
714	Cardiobacterium hominis
715	Cardiobacterium valvarum
716	Carnimonas nigrificans
717	Carnobacterium jeotgali
718	Carnobacterium maltaromaticum
719	Carnobacterium sp,
720	Catabacter hongkongensis
721	Catelliglobosispora koreensis
722	
723	Catenibacterium sp,
724	Catenovulum agarivorans
725	Catenulispora acidiphila
726	Catonella morbi
727	Caulobacter crescentus
728	Caulobacter segnis
729	Caulobacter sp,
730	Caulobacteraceae bacterium
731	Cavia porcellus
732	Cedecea davisae
733	Cellulomonas fimi
734	Cellulomonas massiliensis
735	Cellulomonas sp,
736	Cellulophaga algicola
737	Cellulophaga lytica
738	Cellulosimicrobium cellulans
739	Cellvibrio gilvus
740	Cellvibrio japonicus
741	Cellvibrio sp,
742	Centipeda periodontii
743	Ceratitis capitata
744	Ceratotherium simum
745	Cetobacterium somerae
746	Chaetomium globosum
747	Chaetomium thermophilum
748	Chamaesiphon minutus

749	Chelativorans sp,
750	Chelatococcus sp,
751	Chelonia mydas
752	Chinchilla lanigera
753	Chitinibacter tainanensis
754	Chitinilyticum aquatile
755	Chitinilyticum litopenaei
756	Chitinimonas koreensis
757	Chitiniphilus shinanonensis
758	Chitinophaga pinensis
759	Chitinophagaceae bacterium
760	Chlamydomonas reinhardtii
761	Chlorella variabilis
762	Chlorobaculum parvum
763	Chlorobium chlorochromatii
764	Chlorobium limicola
765	Chlorobium luteolum
766	Chlorobium phaeobacteroides
767	Chlorobium phaeovibrioides
768	Chlorobium tepidum
769	Chlorocebus aethiops
770	Chlorocebus sabaeus
771	Chloroflexi bacterium
772	Chloroflexus aggregans
773	Chloroflexus sp,
774	Chlorogloeopsis fritschii
775	Chloroherpeton thalassium
776	Choloepus hoffmanni
777	Christensenella minuta
778	Chromobacterium violaceum
779	Chromohalobacter salexigens
780	Chroococcidiopsis thermalis
781	Chrysemys picta
782	Chryseobacterium antarcticum
783	Chryseobacterium caeni
784	Chryseobacterium daeguense
785	Chryseobacterium gleum
786	Chryseobacterium gregarium
787	Chryseobacterium haifense
788	Chryseobacterium hispalense
789	Chryseobacterium taeanense
790	Chrysiogenes arsenatis
791	Chrysochloris asiatica
792	Chthonomonas calidirosea
793	Cicer arietinum
794	Citreicella sp,
795	Citricoccus sp,
796	Citromicrobium bathyomarinum
797	Citromicrobium sp,
798	Citrus clementina
799	Citrus sinensis
800	Cladophialophora carrionii
801	Clavibacter michiganensis
802	Clavispora lusitaniae
803	Cloacimonetes bacterium
804	Clostridiales bacterium
805	Clostridiales genomosp,
806	Clostridiisalibacter paucivorans

807	Clostridium aceticum
808	Clostridium acetobutylicum
809	Clostridium aciditolerans
810	Clostridium acidurici
811	Clostridium akagii
812	Clostridium algidicarnis
813	Clostridium arbusti
814	Clostridium aurantibutyricum
815	Clostridium autoethanogenum
816	Clostridium beijerinckii
817	Clostridium botulinum
818	Clostridium butyricum
819	Clostridium cadaveris
820	Clostridium carboxidivorans
821	Clostridium celatum
822	Clostridium cellobioparum
823	Clostridium cellulolyticum
824	Clostridium cellulovorans
825	Clostridium citroniae
826	Clostridium clariflavum
827	Clostridium clostridioforme
828	Clostridium colicanis
829	Clostridium difficile
830	Clostridium disporicum
831	Clostridium formicaceticum
832	Clostridium ganghwense
833	Clostridium glycolicum
834	Clostridium hiranonis
835	Clostridium hydrogeniformans
836	Clostridium hylemonae
837	Clostridium innocuum
838	Clostridium intestinale
839	Clostridium kluyveri
840	Clostridium lentocellum
841	Clostridium ljungdahlii
842	Clostridium lundense
843	Clostridium novyi
844	Clostridium paraputrificum
845	Clostridium pasteurianum
846	Clostridium perfringens
847	Clostridium phytofermentans
848	Clostridium ramosum
849	Clostridium saccharobutylicum
850	Clostridium saccharogumia
851	Clostridium saccharolyticum
852	Clostridium saccharoperbutylacetonicum
853	Clostridium scatologenes
854	Clostridium senegalense
855	Clostridium sp,
856	Clostridium spiroforme
857	Clostridium sporogenes
858	Clostridium sporosphaeroides
859	Clostridium stercorarium
860	Clostridium tertium
861	Clostridium tetani
862	Clostridium thermobutyricum
863	Clostridium thermocellum
864	Clostridium tunisiense
865	Clostridium tyrobutyricum
866	Clostridium ultunense
867	Clostridium viride
868	Cobetia crustatorum
869	Cohnella laeviribosi
870	Cohnella panacarvi
871	Cohnella thermotolerans
872	Colletotrichum gloeosporioides
873	Collimonas fungivorans
874	Collinsella intestinalis
875	Collinsella sp,
876	Collinsella tanakaei
877	Columba livia
878	Colwellia piezophila
879	Comamonadaceae bacterium
880	Comamonas badia
881	Comamonas composti
882	Comamonas sp,
883	Comamonas testosteroni
884	complete chromosome
885	Conchiformibius kuhniae
886	Conchiformibius steedae
887	Condylura cristata
888	Conexibacter woesei
889	Congregibacter litoralis
890	Coniophora puteana
891	Coprobacillus sp,
892	Coprobacter fastidiosus
893	Coprococcus comes
894	Coprococcus eutactus
895	Coprococcus sp,
896	Coprothermobacter proteolyticus
897	Coraliomargarita akajimensis
898	Coraliomargarita sp,
899	Corallococcus coralloides
900	Corallococcus sp,
901	Cordyceps militaris
902	Coriobacteriaceae bacterium
903	Coriobacterium glomerans
904	Corticium candelabrum
905	Corvus brachyrhynchos
906	Corynebacterium accolens
907	Corynebacterium ammoniagenes
908	Corynebacterium argentoratense
909	Corynebacterium aurimucosum
910	Corynebacterium bovis
911	Corynebacterium callunae
912	Corynebacterium capitovis
913	Corynebacterium ciconiae
914	Corynebacterium crenatum
915	Corynebacterium diphtheriae
916	Corynebacterium doosanense
917	Corynebacterium durum
918	Corynebacterium efficiens
919	Corynebacterium genitalium
920	Corynebacterium glucuronolyticum
921	Corynebacterium halotolerans
922	Corynebacterium jeikeium

923	Corynebacterium kroppenstedtii
924	Corynebacterium lipophiloflavum
925	Corynebacterium lubricantis
926	Corynebacterium maris
927	Corynebacterium massiliense
928	Corynebacterium mastitidis
929	Corynebacterium matruchotii
930	Corynebacterium pilosum
931	Corynebacterium propinquum
932	Corynebacterium pseudodiphtheriticum
933	Corynebacterium pseudogenitalium
934	Corynebacterium resistens
935	Corynebacterium sp,
936	Corynebacterium sputi
937	Corynebacterium striatum
938	Corynebacterium terpenotabidum
939	Corynebacterium ulcerans
940	Corynebacterium ulceribovis
941	Corynebacterium urealyticum
942	Corynebacterium variabile
943	Corynebacterium-like bacterium
944	Coxiella burnetii
945	Crenarchaeota archaeon
946	Cricetulus griseus
947	Croceibacter atlanticus
948	Cronobacter helveticus
949	Cronobacter pulveris
950	Cronobacter sakazakii
951	Cronobacter sp,
952	Cronobacter turicensis
953	Cryocola sp,
954	Cryptobacterium curtum
955	Cryptobacterium sp,
956	Cucumibacter marinus
957	Cucumis melo
958	Cucumis sativus
959	Culex pipiens
960	Cupriavidus metallidurans
961	Cupriavidus necator
962	Cupriavidus sp,
963	Cupriavidus taiwanensis
964	Curtobacterium flaccumfaciens
965	Curtobacterium sp,
966	Curvibacter gracilis
967	Curvibacter lanceolatus
968	Cyanobacterium PCC
969	Cyanobium gracile
970	Cyanobium sp,
971	Cyanothece sp,
972	Cynoglossus semilaevis
973	Cyphellophora europaea
974	Cytophaga aurantiaca
975	Cytophaga hutchinsonii
976	Cytophagaceae bacterium
977	Cytophagales bacterium
978	Dactylococcopsis salina
979	Danio rerio
980	Dasania marina

981	Dasypus novemcinctus
982	Dechloromonas agitata
983	Dechloromonas aromatica
984	Dechlorosoma suillum
985	Deferrisoma camini
986	Defluviitalea saccharophila
987	Dehalobacter restrictus
988	Dehalobacter sp,
989	Dehalococcoides ethenogenes
990	Dehalococcoides mccartyi
991	Dehalococcoides sp,
992	Dehalococcoidia bacterium
993	Dehalogenimonas lykanthroporepellens
994	Delftia acidovorans
995	Delftia sp,
996	Delta proteobacterium
997	Demetria terragena
998	Denitrobacterium detoxificans
999	Denitrovibrio acetiphilus
1000	Dermabacter sp,
1001	Dermatophilus congolensis
1002	Derxia gummosa
1003	Desmospora sp,
1004	Desulfarculus baarsii
1005	Desulfatibacillum aliphaticivorans
1006	Desulfatibacillum alkenivorans
1007	Desulfatirhabdium butyrativorans
1008	Desulfitibacter alkalitolerans
1009	Desulfitispora alkaliphila
1010	Desulfitobacterium dichloroeliminans
1011	Desulfitobacterium hafniense
1012	Desulfitobacterium sp,
1013	Desulfobacca acetoxidans
1014	Desulfobacter curvatus
1015	Desulfobacter postgatei
1016	Desulfobacterium anilini
1017	Desulfobacterium autotrophicum
1018	Desulfobacula toluolica
1019	Desulfobulbus elongatus
1020	Desulfobulbus japonicus
1021	Desulfobulbus mediterraneus
1022	Desulfobulbus propionicus
1023	Desulfocapsa sulfexigens
1024	Desulfococcus oleovorans
1025	Desulfocurvus vexinensis
1026	Desulfohalobium retbaense
1027	Desulfomicrobium baculatum
1028	Desulfomicrobium escambiense
1029	Desulfomonile tiedjei
1030	Desulfonatronovibrio hydrogenovorans
1031	Desulfonatronum lacustre
1032	Desulfonatronum thiodismutans
1033	Desulfonauticus sp,
1034	Desulforegula conservatrix
1035	Desulfosarcina sp,
1036	Desulfospira joergensenii
1037	Desulfosporosinus acidiphilus
1038	Desulfosporosinus meridiei

1039	Desulfosporosinus orientis
1040	Desulfosporosinus youngiae
1041	Desulfotalea psychrophila
1042	Desulfotignum balticum
1043	Desulfotomaculum acetoxidans
1044	Desulfotomaculum alcoholivorax
1045	Desulfotomaculum alkaliphilum
1046	Desulfotomaculum carboxydivorans
1047	Desulfotomaculum gibsoniae
1048	Desulfotomaculum intricatum
1049	Desulfotomaculum kuznetsovii
1050	Desulfotomaculum nigrificans
1051	Desulfotomaculum reducens
1052	Desulfotomaculum ruminis
1053	Desulfotomaculum thermocisternum
1054	Desulfovermiculus halophilus
1055	Desulfovibrio aespoeensis
1056	Desulfovibrio africanus
1057	Desulfovibrio alaskensis
1058	Desulfovibrio alcoholivorans
1059	Desulfovibrio aminophilus
1060	Desulfovibrio bastinii
1061	Desulfovibrio cf,
1062	Desulfovibrio cuneatus
1063	Desulfovibrio frigidus
1064	Desulfovibrio gigas
1065	Desulfovibrio hydrothermalis
1066	Desulfovibrio inopinatus
1067	Desulfovibrio intestinalis
1068	Desulfovibrio lacusfryxellense
1069	Desulfovibrio longus
1070	Desulfovibrio magneticus
1071	Desulfovibrio oxyclinae
1072	Desulfovibrio piezophilus
1073	Desulfovibrio piger
1074	Desulfovibrio putealis
1075	Desulfovibrio salexigens
1076	Desulfovibrio simplex
1077	Desulfovibrio sp,
1078	Desulfovibrio vulgaris
1079	Desulfovirgula thermocuniculi
1080	Desulfurispirillum indicum
1081	Desulfurispora thermophila
1082	Desulfurivibrio alkaliphilus
1083	Desulfurobacterium sp,
1084	Desulfuromonas sp,
1085	Dialister invisus
1086	Dialister micraerophilus
1087	Dialister propionicifaciens
1088	Dialister sp,
1089	Dialister succinatiphilus
1090	Diaphorina citri
1091	Dichomitus squalens
1092	Dickeya chrysanthemi
1093	Dickeya dadantii
1094	Dickeya dianthicola
1095	Dickeya paradisiaca
1096	Dickeya zeae
1097	Dictyoglomus thermophilum
1098	Dictyoglomus turgidum
1099	Dictyostelium discoideum
1100	Dictyostelium fasciculatum
1101	Dictyostelium purpureum
1102	Dielma fastidiosa
1103	Dietzia alimentaria
1104	Dietzia sp,
1105	Diplorickettsia massiliensis
1106	Dolichospermum circinale
1107	Donghicola xiamenensis
1108	Dongia sp,
1109	Dorea sp,
1110	Drosophila ananassae
1111	Drosophila erecta
1112	Drosophila grimshawi
1113	Drosophila melanogaster
1114	Drosophila mojavensis
1115	Drosophila persimilis
1116	Drosophila pseudoobscura
1117	Drosophila sechellia
1118	Drosophila simulans
1119	Drosophila virilis
1120	Drosophila willistoni
1121	Drosophila yakuba
1122	Duganella zoogloeoides
1123	Dyadobacter alkalitolerans
1124	Dyadobacter beijingensis
1125	Dyadobacter crusticola
1126	Dyadobacter fermentans
1127	Dyadobacter tibetensis
1128	Dyella ginsengisoli
1129	Dyella japonica
1130	Dysgonomonas capnocytophagoides
1131	Dysgonomonas gadei
1132	Echinicola vietnamensis
1133	Echinops telfairi
1134	Edwardsiella hoshinae
1135	Edwardsiella ictaluri
1136	Edwardsiella tarda
1137	Eggerthella sinensis
1138	Eggerthella sp,
1139	Eggerthia catenaformis
1140	Eikenella corrodens
1141	Elephantulus edwardii
1142	Elioraea tepidiphila
1143	Elusimicrobium minutum
1144	Emiliania huxleyi
1145	Empedobacter brevis
1146	Emticicia oligotrophica
1147	Endozoicomonas elysicola
1148	Ensifer adhaerens
1149	Ensifer sp,
1150	Entamoeba dispar
1151	Entamoeba histolytica
1152	Entamoeba invadens
1153	Enterobacter aerogenes
1154	Enterobacter asburiae

1155	Enterobacter cancerogenus
1156	Enterobacter cloacae
1157	Enterobacter hormaechei
1158	Enterobacter radicincitans
1159	Enterobacter sp,
1160	Enterobacteria phage
1161	Enterobacteriaceae bacterium
1162	Enterocytozoon bieneusi
1163	Enterorhabdus caecimuris
1164	Enterorhabdus mucosicola
1165	Enterovibrio calviensis
1166	Enterovibrio norvegicus
1167	Entomoplasma lucivorax
1168	Entomoplasma luminosum
1169	Entomoplasma melaleucae
1170	Eogystia hippophaecolus
1171	Epilithonimonas tenax
1172	Eptesicus fuscus
1173	Epulopiscium sp,
1174	Equus caballus
1175	Equus przewalskii
1176	Eremococcus coleocola
1177	Erinaceus europaeus
1178	Erwinia amylovora
1179	Erwinia billingiae
1180	Erwinia pyrifoliae
1181	Erwinia sp,
1182	Erwinia tasmaniensis
1183	Erwinia toletana
1184	Erwinia tracheiphila
1185	Erysipelothrix rhusiopathiae
1186	Erysipelothrix tonsillarum
1187	Erysipelotrichaceae bacterium
1188	Erythrobacter litoralis
1189	Erythrobacter sp,
1190	Escherichia albertii
1191	Escherichia blattae
1192	Escherichia fergusonii
1193	Escherichia sp,
1194	Eubacteriaceae bacterium
1195	Eubacterium biforme
1196	Eubacterium brachy
1197	Eubacterium cellulosolvens
1198	Eubacterium coprostanoligenes
1199	Eubacterium cylindroides
1200	Eubacterium desmolans
1201	Eubacterium dolichum
1202	Eubacterium infirmum
1203	Eubacterium limosum
1204	Eubacterium oxidoreducens
1205	Eubacterium plexicaudatum
1206	Eubacterium pyruvativorans
1207	Eubacterium ramulus
1208	Eubacterium ruminantium
1209	Eubacterium saburreum
1210	Eubacterium saphenum
1211	Eubacterium sp,
1212	Eubacterium uniforme

1213	Eubacterium xylanophilum
1214	Eubacterium yurii
1215	Euryarchaeote SCGC
1216	Eutrema salsugineum
1217	Eutypa lata
1218	Exiguobacterium acetylicum
1219	Exiguobacterium antarcticum
1220	Exiguobacterium aurantiacum
1221	Exiguobacterium marinum
1222	Exiguobacterium oxidotolerans
1223	Exiguobacterium pavilionensis
1224	Exiguobacterium sibiricum
1225	Exiguobacterium sp,
1226	Exiguobacterium undae
1227	Exophiala dermatitidis
1228	Facklamia hominis
1229	Facklamia ignava
1230	Facklamia languida
1231	Facklamia sourekii
1232	Falco cherrug
1233	Falco peregrinus
1234	Fangia hongkongensis
1235	Felis catus
1236	Ferrimonas balearica
1237	Ferrimonas futtsuensis
1238	Ferrimonas kyonanensis
1239	Ferrimonas senticii
1240	Ferrovum myxofaciens
1241	Fervidicella metallireducens
1242	Fibrella aestuarina
1243	Fibrobacter succinogenes
1244	Ficedula albicollis
1245	Fictibacillus phosphorivorans
1246	Filamentous cyanobacterium
1247	Filifactor alocis
1248	Filifactor villosus
1249	Finegoldia magna
1250	Firmicutes bacterium
1251	Fischerella sp,
1252	Flavobacteria bacterium
1253	Flavobacterium branchiophilum
1254	Flavobacterium daejeonense
1255	Flavobacterium johnsoniae
1256	Flavobacterium soli
1257	Flavobacterium sp,
1258	Flavobacterium subsaxonicum
1259	Flavobacterium tegetincola
1260	Flavonifractor plautii
1261	Flectobacillus major
1262	Flexibacter elegans
1263	Flexibacter litoralis
1264	Flexistipes sinusarabici
1265	Flexithrix dorotheae
1266	Fluviicola taffensis
1267	Fodinicurvata fenggangensis
1268	Fodinicurvata sediminis
1269	Fragaria vesca
1270	Fragaria virginiana

1271	Francisella sp,
1272	Frankia alni
1273	Frankia sp,
1274	Frankia symbiont
1275	Frateuria aurantia
1276	Fulvimarina pelagi
1277	Fusibacter paucivorans
1278	Fusibacter tunisiensis
1279	Fusicatenibacter saccharivorans
1280	Fusobacterium gonidiaformans
1281	Fusobacterium mortiferum
1282	Fusobacterium naviforme
1283	Fusobacterium necrophorum
1284	Fusobacterium nucleatum
1285	Fusobacterium perfoetens
1286	Fusobacterium periodonticum
1287	Fusobacterium russii
1288	Fusobacterium sp,
1289	Fusobacterium ulcerans
1290	Fusobacterium varium
1291	Gaetbulibacter saemankumensis
1292	Gaeumannomyces graminis
1293	Galdieria sulphuraria
1294	Galeopterus variegatus
1295	Gallibacterium anatis
1296	Gallionella sp,
1297	Gallus gallus
1298	Gamma proteobacterium
1299	gamma proteobacterium
1300	Gardnerella vaginalis
1301	Geitlerinema sp,
1302	Gelidibacter mesophilus
1303	Gemella bergeriae
1304	Gemella cuniculi
1305	Gemella haemolysans
1306	Gemella moribillum
1307	Gemella sanguinis
1308	Geminicoccus roseus
1309	Geminocystis herdmanii
1310	Gemmata obscuriglobus
1311	Gemmatimonadetes bacterium
1312	Gemmatimonas aurantiaca
1313	Gemmatimonas sp,
1314	Gemmiger formicilis
1315	Gemmobacter nectariphilus
1316	Geobacillus caldoxylosilyticus
1317	Geobacillus sp,
1318	Geobacillus thermocatenulatus
1319	Geobacillus thermodenitrificans
1320	Geobacillus thermoglucosidans
1321	Geobacillus thermoleovorans
1322	Geobacillus vulcani
1323	Geobacter bemidjiensis
1324	Geobacter bremensis
1325	Geobacter daltonii
1326	Geobacter lovleyi
1327	Geobacter metallireducens
1328	Geobacter psychrophilus

1329	Geobacter sp,
1330	Geobacter sulfurreducens
1331	Geobacter uraniireducens
1332	Geodermatophilaceae bacterium
1333	Geodermatophilus obscurus
1334	Geopsychrobacter electrodiphilus
1335	Georgenia ruanii
1336	Geospiza fortis
1337	Geothrix fermentans
1338	Geovibrio sp,
1339	Gibberella zeae
1340	Gillisia limnaea
1341	Gilvimarinus chinensis
1342	Glaciecola nitratireducens
1343	Glaciecola pallidula
1344	Glaciecola sp,
1345	Glaciibacter superstes
1346	Gloeobacter kilaueensis
1347	Gloeobacter violaceus
1348	Gloeocapsa sp,
1349	Gloeophyllum trabeum
1350	Gluconacetobacter diazotrophicus
1351	Gluconacetobacter europaeus
1352	Gluconacetobacter hansenii
1353	Gluconacetobacter oboediens
1354	Gluconacetobacter sp,
1355	Gluconacetobacter xylinus
1356	Gluconobacter oxydans
1357	Glycine max
1358	Glycomyces arizonensis
1359	Glycomyces sp,
1360	Glycomyces tenuis
1361	Gordonia amicalis
1362	Gordonia bronchialis
1363	Gordonia kroppenstedtii
1364	Gordonia polyisoprenivorans
1365	Gordonia shandongensis
1366	Gordonia sp,
1367	Gorilla gorilla
1368	Gracilibacillus lacisalsi
1369	Gracilibacter thermotolerans
1370	Gracilimonas tropica
1371	Gramella forsetii
1372	Granulibacter bethesdensis
1373	Granulicatella adiacens
1374	Granulicella mallensis
1375	Granulicella tundricola
1376	Granulicoccus phenolivorans
1377	Gryllotalpicola ginsengisoli
1378	Haemophilus aegyptius
1379	Haemophilus influenzae
1380	Haemophilus parasuis
1381	Haemophilus somnus
1382	Haemophilus sp,
1383	Hafnia alvei
1384	Hahella chejuensis
1385	Hahella ganghwensis
1386	Haladaptatus paucihalophilus

1387	Halalkalicoccus jeotgali
1388	Halanaerobium hydrogeniformans
1389	Halanaerobium praevalens
1390	Haliangium ochraceum
1391	Haliea salexigens
1392	Hallella seregens
1393	Haloarcula hispanica
1394	Haloarcula marismortui
1395	Halobacillus halophilus
1396	Halobacillus kuroshimensis
1397	Halobacterium salinarum
1398	Halobacteroides halobius
1399	Halobiforma lacisalsi
1400	Halococcus hamelinensis
1401	Haloferax mediterranei
1402	Haloferax volcanii
1403	Halogeometricum borinquense
1404	Haloglycomyces albus
1405	Halomicrobium katesii
1406	Halomicrobium mukohataei
1407	Halomonas anticariensis
1408	Halomonas boliviensis
1409	Halomonas elongata
1410	Halomonas halodenitrificans
1411	Halomonas jeotgali
1412	Halomonas lutea
1413	Halomonas smyrnensis
1414	Halomonas sp,
1415	Halomonas stevensii
1416	Halomonas zhanjiangensis
1417	Halomonas zincidurans
1418	Halonatronum saccharophilum
1419	Halopiger xanaduensis
1420	Halorhabdus tiamatea
1421	Halorhabdus utahensis
1422	Halorhodospira halochloris
1423	Halorhodospira halophila
1424	Halorubrum lacusprofundi
1425	Halorubrum sp,
1426	Halotalea alkalilenta
1427	Haloterrigena turkmenica
1428	Halothermothrix orenii
1429	Halothiobacillus neapolitanus
1430	Hamadaea tsunoensis
1431	Hammondia hammondi
1432	Haplochromis burtoni
1433	Helcococcus kunzii
1434	Helcococcus sueciensis
1435	Helicobacter bilis
1436	Helicobacter bizzozeronii
1437	Helicobacter canadensis
1438	Helicobacter cetorum
1439	Helicobacter felis
1440	Helicobacter hepaticus
1441	Helicobacter pullorum
1442	Helicobacter pylori
1443	Helicobacter rodentium
1444	Heliobacterium modesticaldum

1445	Helobdella robusta
1446	Henriciella marina
1447	Herbaspirillum lusitanum
1448	Herbaspirillum massiliense
1449	Herbaspirillum rubrisubalbicans
1450	Herbaspirillum seropedicae
1451	Herbidospora cretacea
1452	Herminiimonas arsenicoxydans
1453	Herminiimonas sp,
1454	Herpetosiphon aurantiacus
1455	Hespellia porcina
1456	Hespellia stercorisuis
1457	Heterocephalus glaber
1458	Hippea alviniae
1459	Hippea sp,
1460	Hoeflea phototrophica
1461	Hoeflea sp,
1462	Holdemania massiliensis
1463	Holophaga foetida
1464	Homo sapiens
1465	Howardella ureilytica
1466	Humibacter albus
1467	Hydra magnipapillata
1468	Hydrogenoanaerobacterium saccharovorans
1469	Hydrogenobacter thermophilus
1470	Hydrogenobaculum sp,
1471	Hymenobacter aerophilus
1472	Hymenobacter norwichensis
1473	Hyphomicrobium denitrificans
1474	Hyphomicrobium nitrativorans
1475	Hyphomicrobium sp,
1476	Hyphomicrobium zavarzinii
1477	Hyphomonas neptunium
1478	Ichthyophthirius multifiliis
1479	Ideonella sp,
1480	Idiomarina baltica
1481	Idiomarina loihiensis
1482	Idiomarina sediminum
1483	Ignavibacterium album
1484	Ilyobacter polytropus
1485	Inquilinus limosus
1486	Intestinimonas butyriciproducens
1487	Intrasporangiaceae bacterium
1488	Isoptericola variabilis
1489	Isosphaera pallida
1490	Ixodes scapularis
1491	Jaculus jaculus
1492	Janibacter sp,
1493	Jannaschia sp,
1494	Janthinobacterium lividum
1495	Janthinobacterium sp,
1496	Jeotgalicoccus psychrophilus
1497	Jiangella gansuensis
1498	Johnsonella ignava
1499	Jonquetella anthropi
1500	Kaistia adipata
1501	Kaistia granuli
1502	Kamptonema formosum

1503	Kandleria vitulina
1504	Kangiella koreensis
1505	Kazachstania africana
1506	Ketogulonigenium vulgarum
1507	Kineococcus radiotolerans
1508	Kingella denitrificans
1509	Kingella kingae
1510	Kingella oralis
1511	Kitasatospora setae
1512	Kitasatospora sp,
1513	Klebsiella oxytoca
1514	Klebsiella pneumoniae
1515	Klebsiella sp,
1516	Kocuria atrinae
1517	Kocuria rhizophila
1518	Kocuria sp,
1519	Kordia algicida
1520	Kordiimonas gwangyangensis
1521	Kosmotoga olearia
1522	Kozakia baliensis
1523	Kribbella catacumbae
1524	Kribbella flavida
1525	Krokinobacter sp,
1526	Kurthia huakuii
1527	Kurthia massiliensis
1528	Kushneria aurantia
1529	Kutzneria albida
1530	Kyrpidia tusciae
1531	Kytococcus sedentarius
1532	Labrenzia alexandrii
1533	Labrenzia sp,
1534	Laccaria bicolor
1535	Laceyella sacchari
1536	Lachnobacterium bovis
1537	Lachnoclostridium phytofermentans
1538	Lachnospira multipara
1539	Lachnospiraceae bacterium
1540	Lachnospiraceae oral
1541	Lacticigenium naphtae
1542	Lactobacillus acidipiscis
1543	Lactobacillus acidophilus
1544	Lactobacillus amylovorus
1545	Lactobacillus animalis
1546	Lactobacillus antri
1547	Lactobacillus apodemi
1548	Lactobacillus brevis
1549	Lactobacillus buchneri
1550	Lactobacillus casei
1551	Lactobacillus coleohominis
1552	Lactobacillus coryniformis
1553	Lactobacillus delbrueckii
1554	Lactobacillus equi
1555	Lactobacillus fabifermentans
1556	Lactobacillus farciminis
1557	Lactobacillus fermentum
1558	Lactobacillus fuchuensis
1559	Lactobacillus gallinarum
1560	Lactobacillus harbinensis
1561	Lactobacillus hayakitensis
1562	Lactobacillus helveticus
1563	Lactobacillus hilgardii
1564	Lactobacillus iners
1565	Lactobacillus johnsonii
1566	Lactobacillus malefermentans
1567	Lactobacillus namurensis
1568	Lactobacillus nodensis
1569	Lactobacillus paracasei
1570	Lactobacillus parafarraginis
1571	Lactobacillus paralimentarius
1572	Lactobacillus plantarum
1573	Lactobacillus pobuzihii
1574	Lactobacillus reuteri
1575	Lactobacillus rhamnosus
1576	Lactobacillus rogosae
1577	Lactobacillus rossiae
1578	Lactobacillus ruminis
1579	Lactobacillus saerimneri
1580	Lactobacillus sakei
1581	Lactobacillus salivarius
1582	Lactobacillus sanfranciscensis
1583	Lactobacillus shenzhenensis
1584	Lactobacillus sp,
1585	Lactobacillus ultunensis
1586	Lactobacillus vini
1587	Lactobacillus zeae
1588	Lactococcus garvieae
1589	Lactococcus phage
1590	Lactococcus raffinolactis
1591	Lactonifactor longoviformis
1592	Lamprocystis purpurea
1593	Laribacter hongkongensis
1594	Latescibacteria bacterium
1595	Latimeria chalumnae
1596	Lautropia mirabilis
1597	Lawsonia intracellularis
1598	Leadbetterella byssophila
1599	Lechevalieria aerocolonigenes
1600	Leeia oryzae
1601	Leeuwenhoekiella blandensis
1602	Leeuwenhoekiella sp,
1603	Legionella geestiana
1604	Legionella longbeachae
1605	Legionella pneumophila
1606	Legionella sainthelensi
1607	Legionella wadsworthii
1608	Leifsonia aquatica
1609	Leifsonia sp,
1610	Leifsonia xyli
1611	Leishmania braziliensis
1612	Leishmania donovani
1613	Leishmania infantum
1614	Leishmania major
1615	Leishmania mexicana
1616	Leisingera aquimarina
1617	Leisingera methylohalidivorans
1618	Leisingera nanhaiensis

1619	Leminorella grimontii
1620	Lentibacillus jeotgali
1621	Lentisphaerae bacterium
1622	Lentzea albidocapillata
1623	Lepisosteus oculatus
1624	Leptolyngbya sp,
1625	Leptonema illini
1626	Leptonychotes weddellii
1627	Leptosphaeria maculans
1628	Leptospira interrogans
1629	Leptospira licerasiae
1630	Leptospirillum ferriphilum
1631	Leptothrix cholodnii
1632	Leptothrix ochracea
1633	Leptotrichia buccalis
1634	Leptotrichia goodfellowii
1635	Leptotrichia hofstadii
1636	Leptotrichia shahii
1637	Leptotrichia sp,
1638	Leptotrichia trevisanii
1639	Leptotrichia wadei
1640	Leucobacter chromiiresistens
1641	Leucobacter salsicius
1642	Leucobacter sp,
1643	Leuconostoc argentinum
1644	Leuconostoc citreum
1645	Leuconostoc fallax
1646	Leuconostoc gelidum
1647	Leuconostoc kimchii
1648	Leuconostoc lactis
1649	Leuconostoc phage
1650	Leucothrix mucor
1651	Lewinella cohaerens
1652	Lewinella persica
1653	Limnohabitans sp,
1654	Lipotes vexillifer
1655	Listeria innocua
1656	Listeria ivanovii
1657	Listeria monocytogenes
1658	Listeria welshimeri
1659	Lithodesmium undulatum
1660	Litorimonas taeanensis
1661	Loa loa
1662	Loktanella hongkongensis
1663	Loktanella vestfoldensis
1664	Longispora albida
1665	Lonsdalea quercina
1666	Lottia gigantea
1667	Loxodonta africana
1668	Luteimonas huabeiensis
1669	Luteimonas mephitis
1670	Luteimonas sp,
1671	Lyngbya majuscula
1672	Lysinibacillus boronitolerans
1673	Lysinibacillus fusiformis
1674	Lysinibacillus sphaericus
1675	Lysobacter antibioticus
1676	Lysobacter sp,

1677	Macaca fascicularis
1678	Macaca mulatta
1679	Macrococcus caseolyticus
1680	Magnaporthe oryzae
1681	Magnetococcus marinus
1682	Magnetospirillum gryphiswaldense
1683	Magnetospirillum magneticum
1684	Mahella australiensis
1685	Malus x
1686	Manacus vitellinus
1687	Mannheimia granulomatis
1688	Mannheimia haemolytica
1689	Maribacter antarcticus
1690	Maribacter sp,
1691	Maricaulis maris
1692	Maricaulis sp,
1693	marine actinobacterium
1694	marine gamma
1695	Marinilabilia salmonicolor
1696	Marinimicrobia bacterium
1697	Marinimicrobium agarilyticum
1698	Marinimicrobium sp,
1699	Marinitoga piezophila
1700	Marinobacter adhaerens
1701	Marinobacter daepoensis
1702	Marinobacter lipolyticus
1703	Marinobacter nanhaiticus
1704	Marinobacter sp,
1705	Marinobacterium jannaschii
1706	Marinobacterium litorale
1707	Marinobacterium rhizophilum
1708	Marinococcus halotolerans
1709	Marinomonas mediterranea
1710	Marinomonas posidonica
1711	Marinomonas sp,
1712	Marinospirillum insulare
1713	Mariprofundus ferrooxydans
1714	Maritalea myrionectae
1715	Maritimibacter alkaliphilus
1716	Marivirga tractuosa
1717	Marmoricola aequoreus
1718	Marmoricola sp,
1719	Marssonina brunnea
1720	Martelella mediterranea
1721	Martelella sp,
1722	Massilia alkalitolerans
1723	Massilia niastensis
1724	Massilia timonae
1725	Mastigocladopsis repens
1726	Mastigocoleus testarum
1727	Maylandia zebra
1728	Medicago truncatula
1729	Megachile rotundata
1730	Megamonas funiformis
1731	Megamonas hypermegale
1732	Meganema perideroedes
1733	Megasphaera elsdenii
1734	Megasphaera paucivorans

1735	Megavirus lba
1736	Meiothermus cerbereus
1737	Meiothermus chliarophilus
1738	Meiothermus ruber
1739	Meiothermus rufus
1740	Meiothermus silvanus
1741	Meiothermus taiwanensis
1742	Meiothermus timidus
1743	Melampsora larici-populina
1744	Meleagris gallopavo
1745	Melioribacter roseus
1746	Melissococcus plutonius
1747	Melitea salexigens
1748	Melopsittacus undulatus
1749	Merops nubicus
1750	Mesocricetus auratus
1751	Mesoflavibacter zeaxanthinifaciens
1752	Mesoplasma chauliocola
1753	Mesoplasma grammopterae
1754	Mesoplasma lactucae
1755	Mesoplasma photuris
1756	Mesoplasma seiffertii
1757	Mesorhizobium australicum
1758	Mesorhizobium ciceri
1759	Mesorhizobium loti
1760	Mesorhizobium sp,
1761	Mesotoga prima
1762	Metarhizium anisopliae
1763	Metascardovia criceti
1764	Methanocaldococcus sp,
1765	Methanocaldococcus vulcanius
1766	Methanocella arvoryzae
1767	Methanocella paludicola
1768	Methanococcus maripaludis
1769	Methanocorpusculum labreanum
1770	Methanoculleus sp,
1771	Methanofollis liminatans
1772	Methanolobus psychrophilus
1773	Methanomassiliicoccus sp,
1774	Methanopyrus kandleri
1775	Methanoregula boonei
1776	Methanoregula formicicum
1777	Methanosaeta harundinacea
1778	Methanosalsum zhilinae
1779	Methanosarcina acetivorans
1780	Methanosphaera stadtmanae
1781	Methylacidiphilum infernorum
1782	Methylibium petroleiphilum
1783	Methylobacillus glycogenes
1784	Methylobacter luteus
1785	Methylobacter marinus
1786	Methylobacter tundripaludum
1787	Methylobacterium extorquens
1788	Methylobacterium nodulans
1789	Methylobacterium populi
1790	Methylobacterium sp,
1791	Methylocaldum szegediense
1792	Methylocella silvestris

1793	Methylococcus capsulatus
1794	Methylocystis parvus
1795	Methylocystis rosea
1796	Methylocystis sp,
1797	Methyloferula stellata
1798	Methylohalobius crimeensis
1799	Methyloligella solikamskensis
1800	Methylomarinum vadi
1801	Methylomicrobium agile
1802	Methylomicrobium alcaliphilum
1803	Methylomonas methanica
1804	Methylomonas sp,
1805	Methylophaga sp,
1806	Methylophilus methylotrophus
1807	Methylophilus sp,
1808	Methylopila sp,
1809	Methylosarcina fibrata
1810	Methylosarcina lacus
1811	Methylosinus sp,
1812	Methylosinus trichosporium
1813	Methylotenera mobilis
1814	Methylotenera sp,
1815	Methyloversatilis sp,
1816	Methyloversatilis universalis
1817	Methylovorus glucosetrophus
1818	Methylovorus sp,
1819	Methylovulum miyakonense
1820	Micavibrio aeruginosavorus
1821	Microbacterium barkeri
1822	Microbacterium gubbeenense
1823	Microbacterium luticocti
1824	Microbacterium maritypicum
1825	Microbacterium paraoxydans
1826	Microbacterium sp,
1827	Microbacterium yannicii
1828	Microbispora rosea
1829	Microbulbifer agarilyticus
1830	Microbulbifer variabilis
1831	Micrococcus luteus
1832	Microcoleus sp,
1833	Microcystis aeruginosa
1834	Microgenomates bacterium
1835	Microlunatus phosphovorus
1836	Micromonas pusilla
1837	Micromonas sp,
1838	Micromonospora chokoriensis
1839	Micromonospora globosa
1840	Micromonospora lupini
1841	Micromonospora parva
1842	Micromonospora purpureochromogenes
1843	Micromonospora sp,
1844	Microplitis demolitor
1845	Microtus ochrogaster
1846	Microvirga sp,
1847	Microvirgula aerodenitrificans
1848	Millerozyma farinosa
1849	Mitsuokella jalaludinii
1850	Mitsuokella sp,

1851	Mobiluncus curtisii
1852	Mobiluncus mulieris
1853	Modestobacter marinus,
1854	Mogibacterium diversum
1855	Mogibacterium neglectum
1856	Mogibacterium timidum
1857	Moniliophthora perniciosa
1858	Monodelphis domestica
1859	Monosiga brevicollis
1860	Moorella thermoacetica
1861	Moraxella caprae
1862	Morganella morganii
1863	Moritella dasanensis
1864	Mucilaginibacter paludis
1865	Mucispirillum schaedleri
1866	Muricauda ruestringensis
1867	Mus musculus
1868	Musca domestica
1869	Mustela putorius
1870	Myceliophthora thermophila
1871	Mycetocola saprophilus
1872	Mycobacterium yongonense
1873	Mycoplasma bovis
1874	Mycoplasma capricolum
1875	Mycoplasma cloacale
1876	Mycoplasma columbinum
1877	Mycoplasma cottewii
1878	Mycoplasma cricetuli
1879	Mycoplasma cynos
1880	Mycoplasma elephantis
1881	Mycoplasma fermentans
1882	Mycoplasma gallinarum
1883	Mycoplasma gallisepticum
1884	Mycoplasma glycophilum
1885	Mycoplasma hyorhinis
1886	Mycoplasma imitans
1887	Mycoplasma iners
1888	Mycoplasma leachii
1889	Mycoplasma leonicaptivi
1890	Mycoplasma moatsii
1891	Mycoplasma mobile
1892	Mycoplasma molare
1893	Mycoplasma mycoides
1894	Mycoplasma opalescens
1895	Mycoplasma orale
1896	Mycoplasma ovipneumoniae
1897	Mycoplasma ovis
1898	Mycoplasma pirum
1899	Mycoplasma pulmonis
1900	Mycoplasma putrefaciens
1901	Mycoplasma sp,
1902	Mycoplasma spumans
1903	Myotis brandtii
1904	Myotis davidii
1905	Myotis lucifugus
1906	Myroides injenensis
1907	Myxococcus fulvus
1908	Myxococcus stipitatus

1909	Myxococcus xanthus
1910	Nafulsella turpanensis
1911	Nakamurella lactea
1912	Nakamurella multipartita
1913	Nannochloropsis gaditana
1914	Nannospalax galili
1915	Nasonia vitripennis
1916	Natranaerovirga hydrolytica
1917	Natranaerovirga pectinivora
1918	Natrialba magadii
1919	Natrinema pellirubrum
1920	Natronobacterium gregoryi
1921	Natronococcus occultus
1922	Naumovozyma castellii
1923	Nectria haematococca
1924	Neisseria bacilliformis
1925	Neisseria gonorrhoeae
1926	Neisseria lactamica
1927	Neisseria meningitidis
1928	Neisseria mucosa
1929	Neisseria shayeganii
1930	Neisseria sicca
1931	Neisseria sp,
1932	Neisseria wadsworthii
1933	Neofusicoccum parvum
1934	Neolamprologus brichardi
1935	Neosartorya fischeri
1936	Neptunomonas japonica
1937	Nesiotobacter exalbescens
1938	Nesterenkonia alba
1939	Nesterenkonia sp,
1940	Nevskia ramosa
1941	Nevskia soli
1942	Niabella aurantiaca
1943	Nisaea denitrificans
1944	Nitratifractor salsuginis
1945	Nitratiruptor sp,
1946	Nitrobacter hamburgensis
1947	Nitrobacter winogradskyi
1948	Nitrococcus mobilis
1949	Nitrosomonas cryotolerans
1950	Nitrosomonas europaea
1951	Nitrosomonas sp,
1952	Nitrosospira briensis
1953	Nitrosospira multiformis
1954	Nitrospina gracilis
1955	Nitrospina sp,
1956	Nitrospirillum amazonense
1957	Nocardia brasiliensis
1958	Nocardia cyriacigeorgica
1959	Nocardia farcinica
1960	Nocardia otitidiscaviarum
1961	Nocardia rhamnosiphila
1962	Nocardia sp,
1963	Nocardioidaceae bacterium
1964	Nocardioides alkalitolerans
1965	Nocardioides halotolerans
1966	Nocardioides insulae

1967	Nocardioides sp,
1968	Nocardiopsis alba
1969	Nocardiopsis alkaliphila
1970	Nocardiopsis baichengensis
1971	Nocardiopsis chromatogenes
1972	Nocardiopsis dassonvillei
1973	Nocardiopsis ganjiahuensis
1974	Nocardiopsis gilva
1975	Nocardiopsis halophila
1976	Nocardiopsis halotolerans
1977	Nocardiopsis kunsanensis
1978	Nocardiopsis lucentensis
1979	Nocardiopsis potens
1980	Nocardiopsis prasina
1981	Nocardiopsis salina
1982	Nocardiopsis sp,
1983	Nocardiopsis valliformis
1984	Nocardiopsis xinjiangensis
1985	Nodosilinea nodulosa
1986	Nomascus leucogenys
1987	Nonomuraea coxensis
1988	Nostoc punctiforme
1989	Nostoc sp,
1990	Novispirillum itersonii
1991	Novosphingobium acidiphilum
1992	Novosphingobium aromaticivorans
1993	Novosphingobium lindaniclasticum
1994	Novosphingobium nitrogenifigens
1995	Novosphingobium sp,
1996	Number of
1997	Oceanicaulis alexandrii
1998	Oceanicola batsensis
1999	Oceanicola granulosus
2000	Oceanicola nanhaiensis
2001	Oceanicola sp,
2002	Oceanimonas sp,
2003	Oceanithermus profundus
2004	Oceanobacillus iheyensis
2005	Oceanobacillus kimchii
2006	Oceanobacter kriegii
2007	Oceanobacter sp,
2008	Oceanospirillales bacterium
2009	Oceanospirillum beijerinckii
2010	Oceanospirillum sp,
2011	Ochotona princeps
2012	Ochrobactrum anthropi
2013	Ochrobactrum intermedium
2014	Ochrobactrum rhizosphaerae
2015	Ochrobactrum sp,
2016	Octadecabacter antarcticus
2017	Octadecabacter arcticus
2018	Octodon degus
2019	Odobenus rosmarus
2020	Odoribacter laneus
2021	Odoribacter sp,
2022	Oedogonium cardiacum
2023	Oenococcus kitaharae
2024	Oenococcus oeni

2025	Oerskovia turbata
2026	Olivibacter terrae
2027	Olleya marilimosa
2028	Olleya sp,
2029	Olsenella sp,
2030	Olsenella uli
2031	Onion yellows
2032	Opitutaceae bacterium
2033	Opitutus terrae
2034	Orcinus orca
2035	Orenia marismortui
2036	Oreochromis niloticus
2037	Oribacterium asaccharolyticum
2038	Oribacterium sinus
2039	Oribacterium sp,
2040	Ornithinibacillus scapharcae
2041	Ornithobacterium rhinotracheale
2042	Ornithorhynchus anatinus
2043	Orycteropus afer
2044	Oryctolagus cuniculus
2045	Oryza brachyantha
2046	Oryza sativa
2047	Oryzias latipes
2048	Oryzias melastigma
2049	Oscillatoria sp,
2050	Oscillatoriales cyanobacterium
2051	Oscillibacter ruminantium
2052	Oscillibacter sp,
2053	Oscillibacter valericigenes
2054	Oscillochloris trichoides
2055	Oscillospiraceae bacterium
2056	Ostreococcus lucimarinus
2057	Ostreococcus tauri
2058	Otolemur garnettii
2059	Ottowia thiooxydans
2060	Ovis aries
2061	Owenweeksia hongkongensis
2062	Oxalobacteraceae bacterium
2063	Oxobacter pfennigii
2064	Paenibacillus alginolyticus
2065	Paenibacillus assamensis
2066	Paenibacillus azotofixans
2067	Paenibacillus barengoltzii
2068	Paenibacillus daejeonensis
2069	Paenibacillus ehimensis
2070	Paenibacillus elgii
2071	Paenibacillus fonticola
2072	Paenibacillus forsythiae
2073	Paenibacillus ginsengihumi
2074	Paenibacillus graminis
2075	Paenibacillus harenae
2076	Paenibacillus jilunlii
2077	Paenibacillus larvae
2078	Paenibacillus massiliensis
2079	Paenibacillus mucilaginosus
2080	Paenibacillus panacisoli
2081	Paenibacillus pasadenensis
2082	Paenibacillus peoriae

2083	Paenibacillus pinihumi
2084	Paenibacillus polymyxa
2085	Paenibacillus sanguinis
2086	Paenibacillus senegalensis
2087	Paenibacillus sp,
2088	Paenibacillus taiwanensis
2089	Paenibacillus terrae
2090	Paenibacillus terrigena
2091	Paenibacillus turicensis
2092	Paenibacillus zanthoxyli
2093	Paenisporosarcina sp,
2094	Paludibacter propionicigenes
2095	Paludibacterium yongneupense
2096	Pan paniscus
2097	Pan troglodytes
2098	Pandoraea pnomenusa
2099	Pandoraea sp,
2100	Pannonibacter phragmitetus
2101	Panthera tigris
2102	Pantholops hodgsonii
2103	Pantoea agglomerans
2104	Pantoea ananatis
2105	Pantoea dispersa
2106	Pantoea sp,
2107	Pantoea vagans
2108	Papillibacter cinnamivorans
2109	Papio anubis
2110	Paracoccidioides brasiliensis
2111	Paracoccus aminophilus
2112	Paracoccus denitrificans
2113	Paracoccus pantotrophus
2114	Paracoccus sp,
2115	Paracoccus yeei
2116	Paracoccus zeaxanthinifaciens
2117	Paramecium tetraurelia
2118	Paraoerskovia marina
2119	Paraprevotella clara
2120	Paraprevotella xylaniphila
2121	Parascardovia denticolens
2122	Parasporobacterium paucivorans
2123	Parasutterella excrementihominis
2124	Parcubacteria bacterium
2125	Parvibaculum lavamentivorans
2126	Parvimonas micra
2127	Parvularcula bermudensis
2128	Parvularcula oceani
2129	Pasteurella dagmatis
2130	Pasteurella pneumotropica
2131	Patulibacter americanus
2132	Patulibacter minatonensis
2133	Paucisalibacillus globulus
2134	Pectobacterium atrosepticum
2135	Pectobacterium carotovorum
2136	Pectobacterium sp,
2137	Pectobacterium wasabiae
2138	Pediculus humanus
2139	Pediococcus acidilactici
2140	Pediococcus claussenii
2141	Pediococcus pentosaceus
2142	Pedobacter borealis
2143	Pedobacter glucosidilyticus
2144	Pelagibaca bermudensis
2145	Pelagibacterium halotolerans
2146	Pelobacter carbinolicus
2147	Pelobacter propionicus
2148	Pelobacter seleniigenes
2149	Pelodictyon phaeoclathratiforme
2150	Pelodiscus sinensis
2151	Pelosinus sp,
2152	Pelospora glutarica
2153	Pelotomaculum thermopropionicum
2154	Penicillium chrysogenum
2155	Penicillium marneffei
2156	Peptoclostridium difficile
2157	Peptostreptococcaceae bacterium
2158	Peptostreptococcus anaerobius
2159	Periwinkle leaf
2160	Perlucidibaca piscinae
2161	Peromyscus maniculatus
2162	Persephonella marina
2163	Persephonella sp,
2164	Pestalotiopsis fici
2165	Petrotoga mobilis
2166	Phaeobacter arcticus
2167	Phaeobacter caeruleus
2168	Phaeobacter daeponensis
2169	Phaeobacter gallaeciensis
2170	Phaeobacter inhibens
2171	Phaeocystis globosa
2172	Phaeosphaeria nodorum
2173	Phanerochaete carnosa
2174	Phaseolus vulgaris
2175	Phenylobacterium zucineum
2176	Phocaeicola abscessus
2177	Phoenix dactylifera
2178	Photobacterium damselae
2179	Photobacterium halotolerans
2180	Phycicoccus jejuensis
2181	Phycisphaera mikurensis
2182	Physcomitrella patens
2183	Physeter catodon
2184	Phytophthora infestans
2185	Pilimelia anulata
2186	Pirellula staleyi
2187	Planctomyces brasiliensis
2188	Planctomyces limnophilus
2189	Planktothrix agardhii
2190	Planktothrix prolifica
2191	Plasmodium berghei
2192	Plasmodium falciparum
2193	Plasmodium vinckei
2194	Plasmodium vivax
2195	Plasmodium yoelii
2196	Pleomorphomonas koreensis
2197	Pleomorphomonas oryzae
2198	Plesiomonas shigelloides

2199	Pleurocapsa sp,
2200	Pleurozia purpurea
2201	Podospora anserina
2202	Poecilia formosa
2203	Poecilia reticulata
2204	Polaromonas glacialis
2205	Polaromonas naphthalenivorans
2206	Polaromonas sp,
2207	Polycyclovorans algicola
2208	Polymorphum gilvum
2209	Polynucleobacter necessarius
2210	Pongo abelii
2211	Pontibacillus halophilus
2212	Pontibacillus marinus
2213	Pontibacter actiniarum
2214	Pontibacter roseus
2215	Ponticaulis koreensis
2216	Populus trichocarpa
2217	Poribacteria bacterium
2218	Porphyrobacter cryptus
2219	Porphyrobacter sp,
2220	Porphyromonas bennonis
2221	Porphyromonas cangingivalis
2222	Porphyromonas gingivalis
2223	Porphyromonas gingivicanis
2224	Porphyromonas levii
2225	Porphyromonas macacae
2226	Porphyromonas sp,
2227	Postia placenta
2228	Prauserella rugosa
2229	Prevotella albensis
2230	Prevotella amnii
2231	Prevotella aurantiaca
2232	Prevotella baroniae
2233	Prevotella bergensis
2234	Prevotella brevis
2235	Prevotella corporis
2236	Prevotella dentalis
2237	Prevotella dentasini
2238	Prevotella denticola
2239	Prevotella disiens
2240	Prevotella enoeca
2241	Prevotella falsenii
2242	Prevotella fusca
2243	Prevotella intermedia
2244	Prevotella loescheii
2245	Prevotella maculosa
2246	Prevotella marshii
2247	Prevotella micans
2248	Prevotella multisaccharivorax
2249	Prevotella nanceiensis
2250	Prevotella nigrescens
2251	Prevotella oralis
2252	Prevotella oris
2253	Prevotella oulorum
2254	Prevotella paludivivens
2255	Prevotella pleuritidis
2256	Prevotella ruminicola
2257	Prevotella salivae
2258	Prevotella shahii
2259	Prevotella sp,
2260	Prevotella stercorea
2261	Prevotella tannerae
2262	Prevotella veroralis
2263	Prochlorococcus marinus
2264	Prochlorococcus sp,
2265	Prochlorothrix hollandica
2266	Promicromonospora sukumoe
2267	Propionibacteriaceae bacterium
2268	Propionibacterium acidifaciens
2269	Propionibacterium acidipropionici
2270	Propionibacterium acnes
2271	Propionibacterium freudenreichii
2272	Propionibacterium jensenii
2273	Propionibacterium propionicum
2274	Propionibacterium sp,
2275	Propionibacterium thoenii
2276	Propionicicella superfundia
2277	Propionimicrobium lymphophilum
2278	Prosthecochloris aestuarii
2279	Proteiniclasticum ruminis
2280	Proteiniphilum acetatigenes
2281	Proteobacteria bacterium
2282	Proteocatella sphenisci
2283	Proteus penneri
2284	Providencia alcalifaciens
2285	Providencia burhodogranariea
2286	Providencia stuartii
2287	Prunus mume
2288	Prunus persica
2289	Pseudaminobacter salicylatoxidans
2290	Pseudanabaena sp,
2291	Pseudoalteromonas atlantica
2292	Pseudoalteromonas haloplanktis
2293	Pseudoalteromonas sp,
2294	Pseudobacteroides cellulosolvens
2295	Pseudobutyrivibrio ruminis
2296	Pseudobutyrivibrio sp,
2297	Pseudochrobactrum sp,
2298	Pseudoclavibacter soli
2299	Pseudoduganella violaceinigra
2300	Pseudogulbenkiania ferrooxidans
2301	Pseudogulbenkiania sp,
2302	Pseudonocardia acaciae
2303	Pseudonocardia asaccharolytica
2304	Pseudonocardia dioxanivorans
2305	Pseudonocardia spinosispora
2306	Pseudophaeobacter arcticus
2307	Pseudopodoces humilis
2308	Pseudoramibacter alactolyticus
2309	Pseudorhodobacter ferrugineus
2310	Pseudovibrio sp,
2311	Pseudoxanthomonas sp,
2312	Pseudoxanthomonas spadix
2313	Pseudoxanthomonas suwonensis
2314	Pseudozyma flocculosa

2315	Psychrilyobacter atlanticus
2316	Psychrobacter phenylpyruvicus
2317	Psychrobacter sp,
2318	Psychromonas aquimarina
2319	Psychromonas arctica
2320	Psychromonas ingrahamii
2321	Psychromonas ossibalaenae
2322	Psychromonas sp,
2323	Psychroserpens burtonensis
2324	Pteropus alecto
2325	Puccinia graminis
2326	Pundamilia nyererei
2327	Pusillimonas noertemannii
2328	Pusillimonas sp,
2329	Pyramidobacter piscolens
2330	Pyrobaculum oguniense
2331	Pyropia haitanensis
2332	Python bivittatus
2333	Rahnella aquatilis
2334	Ralstonia eutropha
2335	Ralstonia pickettii
2336	Ralstonia solanacearum
2337	Ralstonia sp,
2338	Ramlibacter tataouinensis
2339	Raoultella ornithinolytica
2340	Rathayibacter toxicus
2341	Rattus norvegicus
2342	Renibacterium salmoninarum
2343	Rhazya stricta
2344	Rheinheimera perlucida
2345	Rheinheimera texasensis
2346	Rhizobium etli
2347	Rhizobium giardinii
2348	Rhizobium larrymoorei
2349	Rhizobium leguminosarum
2350	Rhizobium leucaenae
2351	Rhizobium mesoamericanum
2352	Rhizobium mongolense
2353	Rhizobium phaseoli
2354	Rhizobium selenitireducens
2355	Rhizobium sp,
2356	Rhizobium sullae
2357	Rhizobium tropici
2358	Rhizobium undicola
2359	Rhodanobacter sp,
2360	Rhodobacter capsulatus
2361	Rhodobacter sp,
2362	Rhodobacter sphaeroides
2363	Rhodobacteraceae bacterium
2364	Rhodobacterales bacterium
2365	Rhodococcus erythropolis
2366	Rhodococcus jostii
2367	Rhodococcus opacus
2368	Rhodococcus pyridinivorans
2369	Rhodococcus rhodochrous
2370	Rhodococcus ruber
2371	Rhodococcus sp,
2372	Rhodoferax ferrireducens

2373	Rhodoferax saidenbachensis
2374	Rhodomicrobium vannielii
2375	Rhodomonas salina
2376	Rhodonellum psychrophilum
2377	Rhodopirellula baltica
2378	Rhodopseudomonas palustris
2379	Rhodopseudomonas sp,
2380	Rhodospirillales bacterium
2381	Rhodospirillum centenum
2382	Rhodospirillum photometricum
2383	Rhodospirillum rubrum
2384	Rhodothermus marinus
2385	Rhodovibrio salinarum
2386	Ricinus communis
2387	Rickettsia bellii
2388	Riemerella anatipestifer
2389	Rikenella microfusus
2390	Rivularia sp,
2391	Robiginitalea biformata
2392	Robinsoniella sp,
2393	Romboutsia ilealis
2394	Roseburia faecis
2395	Roseburia hominis
2396	Roseburia sp,
2397	Roseibium sp,
2398	Roseiflexus castenholzii
2399	Roseobacter denitrificans
2400	Roseobacter litoralis
2401	Roseobacter sp,
2402	Roseomonas cervicalis
2403	Roseomonas gilardii
2404	Roseomonas mucosa
2405	Roseomonas sp,
2406	Roseovarius nubinhibens
2407	Roseovarius sp,
2408	Ruania albidiflava
2409	Rubellimicrobium thermophilum
2410	Rubritalea tangerina
2411	Rubritepida flocculans
2412	Rubrivivax benzoatilyticus
2413	Rubrivivax gelatinosus
2414	Rubrobacter xylanophilus
2415	Rudaea cellulosilytica
2416	Rudanella lutea
2417	Ruegeria conchae
2418	Ruegeria pomeroyi
2419	Ruegeria sp,
2420	Ruminobacter sp,
2421	Ruminococcaceae bacterium
2422	Ruminococcus callidus
2423	Ruminococcus champanellensis
2424	Ruminococcus faecis
2425	Ruminococcus flavefaciens
2426	Ruminococcus gauvreauii
2427	Ruminococcus sp,
2428	Runella limosa
2429	Runella slithyformis
2430	Runella zeae

2431	Saccharibacillus kuerlensis
2432	Saccharofermentans acetigenes
2433	Saccharomonospora azurea
2434	Saccharomonospora cyanea
2435	Saccharomonospora glauca
2436	Saccharomonospora halophila
2437	Saccharomonospora marina
2438	Saccharomonospora paurometabolica
2439	Saccharomonospora saliphila
2440	Saccharomonospora sp,
2441	Saccharomonospora xinjiangensis
2442	Saccharomyces cerevisiae
2443	Saccharophagus degradans
2444	Saccharopolyspora erythraea
2445	Saccharopolyspora rectivirgula
2446	Saccharopolyspora spinosa
2447	Saccharospirillum impatiens
2448	Saccharothrix espanaensis
2449	Saccoglossus kowalevskii
2450	Saimiri boliviensis
2451	Salana multivorans
2452	Salimesophilobacter vulgaris
2453	Salinarimonas rosea
2454	Salinibacter ruber
2455	Salinicoccus albus
2456	Salinicoccus carnicancri
2457	Salinicoccus luteus
2458	Salinimicrobium xinjiangense
2459	Salinispora arenicola
2460	Salinispora pacifica
2461	Salinispora tropica
2462	Salipiger mucosus
2463	Salisaeta longa
2464	Salmonella enterica
2465	Salmonella sp,
2466	Salpingoeca sp,
2467	Salsuginibacillus kocurii
2468	Sandarakinorhabdus limnophila
2469	Sandarakinorhabdus sp,
2470	Sanguibacter keddieii
2471	Saprolegnia diclina
2472	SAR324 cluster
2473	Sarcophilus harrisii
2474	Saxeibacter lacteus
2475	Scardovia inopinata
2476	Scardovia wiggsiae
2477	Schlesneria paludicola
2478	Sciscionella marina
2479	Sciscionella sp,
2480	Sclerotinia sclerotiorum
2481	Sebaldella termitidis
2482	Sedimenticola selenatireducens
2483	Sediminibacillus halophilus
2484	Sediminibacterium salmoneum
2485	Sediminibacterium sp,
2486	Sediminimonas qiaohouensis
2487	Segniliparus rugosus
2488	Serinus canaria

2489	Serratia fonticola
2490	Serratia liquefaciens
2491	Serratia marcescens
2492	Serratia odorifera
2493	Serratia plymuthica
2494	Serratia proteamaculans
2495	Serratia sp,
2496	Setaria italica
2497	Shewanella amazonensis
2498	Shewanella colwelliana
2499	Shewanella fidelis
2500	Shewanella loihica
2501	Shewanella marina
2502	Shewanella putrefaciens
2503	Shewanella sediminis
2504	Shewanella woodyi
2505	Shigella dysenteriae
2506	Shigella flexneri
2507	Shigella sonnei
2508	Shigella sp,
2509	Shimazuella kribbensis
2510	Shinella sp,
2511	Shuttleworthia satelles
2512	Sideroxydans lithotrophicus
2513	Silanimonas lenta
2514	Silicibacter lacuscaerulensis
2515	Silicibacter sp,
2516	Simiduia agarivorans
2517	Simplicispira psychrophila
2518	Singulisphaera acidiphila
2519	Sinobacter flavus
2520	Sinorhizobium arboris
2521	Sinorhizobium fredii
2522	Sinorhizobium meliloti
2523	Smaragdicoccus niigatensis
2524	Sneathiella glossodoripedis
2525	Sodalis glossinidius
2526	Solanum lycopersicum
2527	Solanum tuberosum
2528	Solibacillus silvestris
2529	Solimonas flava
2530	Solimonas soli
2531	Solirubrobacter soli
2532	Solirubrobacter sp,
2533	Solirubrobacterales bacterium
2534	Solitalea canadensis
2535	Sorangium cellulosum
2536	Sordaria macrospora
2537	Sorex araneus
2538	Sorghum bicolor
2539	Spermophilus tridecemlineatus
2540	Sphaerobacter thermophilus
2541	Sphaerochaeta pleomorpha
2542	Sphingobacterium sp,
2543	Sphingobacterium spiritivorum
2544	Sphingobium baderi
2545	Sphingobium chinhatense
2546	Sphingobium chlorophenolicum

2547	Sphingobium lactosutens
2548	Sphingobium quisquiliarum
2549	Sphingobium sp,
2550	Sphingobium ummariense
2551	Sphingobium xenophagum
2552	Sphingobium yanoikuyae
2553	Sphingomonas astaxanthinifaciens
2554	Sphingomonas echinoides
2555	Sphingomonas elodea
2556	Sphingomonas melonis
2557	Sphingomonas phyllosphaerae
2558	Sphingomonas sp,
2559	Sphingomonas wittichii
2560	Sphingomonas-like bacterium
2561	Sphingopyxis alaskensis
2562	Spiribacter sp,
2563	Spirillospora albida
2564	Spirochaeta africana
2565	Spirochaeta alkalica
2566	Spirochaeta caldaria
2567	Spirochaeta cellobiosiphila
2568	Spirochaeta coccoides
2569	Spirochaeta smaragdinae
2570	Spirochaeta sp,
2571	Spirochaeta thermophila
2572	Spiroplasma apis
2573	Spiroplasma chrysopicola
2574	Spiroplasma culicicola
2575	Spiroplasma diminutum
2576	Spiroplasma leptinotarsae
2577	Spiroplasma syrphidicola
2578	Spiroplasma taiwanense
2579	Spirosoma linguale
2580	Spirosoma luteum
2581	Spirosoma panaciterrae
2582	Spirosoma spitsbergense
2583	Spongiibacterium flavum
2584	Sporichthya polymorpha
2585	Sporobacter termitidis
2586	Sporobacterium olearium
2587	Sporocytophaga myxococcoides
2588	Sporolactobacillus inulinus
2589	Sporolactobacillus laevolacticus
2590	Sporolactobacillus terrae
2591	Sporolactobacillus vineae
2592	Sporomusa ovata
2593	Sporosarcina newyorkensis
2594	Sporosarcina pasteurii
2595	Sporosarcina sp,
2596	Stackebrandtia nassauensis
2597	Stanieria cyanosphaera
2598	Staphylococcus aureus
2599	Staphylococcus capitis
2600	Staphylococcus epidermidis
2601	Staphylococcus equorum
2602	Staphylococcus haemolyticus
2603	Staphylococcus hominis
2604	Staphylococcus intermedius

2605	Staphylococcus lentus
2606	Staphylococcus lugdunensis
2607	Staphylococcus massiliensis
2608	Staphylococcus pseudintermedius
2609	Staphylococcus saprophyticus
2610	Staphylococcus simulans
2611	Staphylococcus sp,
2612	Staphylococcus vitulinus
2613	Staphylococcus warneri
2614	Staphylococcus xylosus
2615	Stappia stellulata
2616	Starkeya novella
2617	Stegastes partitus
2618	Stenotrophomonas maltophilia
2619	Stenotrophomonas sp,
2620	Stigmatella aurantiaca
2621	Strawberry lethal
2622	Streptacidiphilus jeojiense
2623	Streptobacillus moniliformis
2624	Streptococcus agalactiae
2625	Streptococcus anginosus
2626	Streptococcus caballi
2627	Streptococcus castoreus
2628	Streptococcus constellatus
2629	Streptococcus devriesei
2630	Streptococcus dysgalactiae
2631	Streptococcus equi
2632	Streptococcus equinus
2633	Streptococcus ferus
2634	Streptococcus gallolyticus
2635	Streptococcus gordonii
2636	Streptococcus henryi
2637	Streptococcus hyovaginalis
2638	Streptococcus infantarius
2639	Streptococcus infantis
2640	Streptococcus iniae
2641	Streptococcus intermedius
2642	Streptococcus lutetiensis
2643	Streptococcus marimammalium
2644	Streptococcus massiliensis
2645	Streptococcus mitis
2646	Streptococcus mutans
2647	Streptococcus oligofermentans
2648	Streptococcus orisratti
2649	Streptococcus parauberis
2650	Streptococcus pasteurianus
2651	Streptococcus peroris
2652	Streptococcus phage
2653	Streptococcus pleomorphus
2654	Streptococcus plurextorum
2655	Streptococcus pneumoniae
2656	Streptococcus porci
2657	Streptococcus pseudopneumoniae
2658	Streptococcus pyogenes
2659	Streptococcus sanguinis
2660	Streptococcus sobrinus
2661	Streptococcus sp,
2662	Streptococcus suis

2663	Streptococcus thermophilus
2664	Streptococcus uberis
2665	Streptomyces acidiscabies
2666	Streptomyces afghaniensis
2667	Streptomyces albus
2668	Streptomyces anulatus
2669	Streptomyces auratus
2670	Streptomyces aureocirculatus
2671	Streptomyces avellaneus
2672	Streptomyces avermitilis
2673	Streptomyces bicolor
2674	Streptomyces bikiniensis
2675	Streptomyces bingchenggensis
2676	Streptomyces bottropensis
2677	Streptomyces canus
2678	Streptomyces catenulae
2679	Streptomyces cattleya
2680	Streptomyces cellulosae
2681	Streptomyces chartreusis
2682	Streptomyces clavuligerus
2683	Streptomyces collinus
2684	Streptomyces cyaneofuscatus
2685	Streptomyces davawensis
2686	Streptomyces erythrochromogenes
2687	Streptomyces exfoliatus
2688	Streptomyces flavidovirens
2689	Streptomyces flavochromogenes
2690	Streptomyces flavotricini
2691	Streptomyces flavovariabilis
2692	Streptomyces flavovirens
2693	Streptomyces fulvissimus
2694	Streptomyces fulvoviolaceus
2695	Streptomyces ghanaensis
2696	Streptomyces globisporus
2697	Streptomyces griseoflavus
2698	Streptomyces griseus
2699	Streptomyces halstedii
2700	Streptomyces hygroscopicus
2701	Streptomyces katrae
2702	Streptomyces lavendulae
2703	Streptomyces lavenduligriseus
2704	Streptomyces leeuwenhoekii
2705	Streptomyces mediolani
2706	Streptomyces megasporus
2707	Streptomyces monomycini
2708	Streptomyces natalensis
2709	Streptomyces niveus
2710	Streptomyces olivaceus
2711	Streptomyces peruviensis
2712	Streptomyces peucetius
2713	Streptomyces pristinaespiralis
2714	Streptomyces prunicolor
2715	Streptomyces purpeofuscus
2716	Streptomyces rapamycinicus
2717	Streptomyces resistomycificus
2718	Streptomyces rimosus
2719	Streptomyces roseochromogenus
2720	Streptomyces roseoverticillatus
2721	Streptomyces ruber
2722	Streptomyces scabiei
2723	Streptomyces scabrisporus
2724	Streptomyces sclerotialus
2725	Streptomyces scopuliridis
2726	Streptomyces seoulensis
2727	Streptomyces sp,
2728	Streptomyces sulphureus
2729	Streptomyces thermodiastaticus
2730	Streptomyces thermolilacinus
2731	Streptomyces varsoviensis
2732	Streptomyces violaceoruber
2733	Streptomyces violaceusniger
2734	Streptomyces virginiae
2735	Streptomyces viridochromogenes
2736	Streptomyces vitaminophilus
2737	Streptomyces xanthophaeus
2738	Streptomycetaceae bacterium
2739	Streptosporangium amethystogenes
2740	Streptosporangium roseum
2741	Strongylocentrotus purpuratus
2742	Subdoligranulum sp,
2743	Succinimonas amylolytica
2744	Succinispira mobilis
2745	Succinivibrio dextrinosolvens
2746	Succinivibrionaceae bacterium
2747	Sulfitobacter guttiformis
2748	Sulfitobacter mediterraneus
2749	Sulfitobacter sp,
2750	Sulfobacillus thermosulfidooxidans
2751	Sulfuricella denitrificans
2752	Sulfurihydrogenibium azorense
2753	Sulfurimonas denitrificans
2754	Sulfurospirillum barnesii
2755	Sulfurospirillum deleyianum
2756	Sulfurovum sp,
2757	Sus scrofa
2758	Sutterella parvirubra
2759	Sutterella sp,
2760	Symbiobacterium thermophilum
2761	Syncytium symbiont
2762	Synechococcus elongatus
2763	Synechococcus sp,
2764	Synechocystis sp,
2765	Synergistes sp,
2766	Synergistetes bacterium
2767	Syntrophobacter fumaroxidans
2768	Syntrophobotulus glycolicus
2769	Syntrophomonas palmitatica
2770	Syntrophomonas wolfei
2771	Syntrophorhabdus aromaticivorans
2772	Syntrophothermus lipocalidus
2773	Syntrophus aciditrophicus
2774	Taeniopygia guttata
2775	Takifugu rubripes
2776	Tannerella forsythia
2777	Tannerella sp,
2778	Tarsius syrichta

2779	Tatumella ptyseos
2780	Taylorella equigenitalis
2781	Tepidanaerobacter acetatoxydans
2782	Tepidibacter thalassicus
2783	Tepidiphilus margaritifer
2784	Terasakiella pusilla
2785	Teredinibacter turnerae
2786	Terracoccus sp,
2787	Terriglobus roseus
2788	Terriglobus saanensis
2789	Terrimonas ferruginea
2790	Terrisporobacter glycolicus
2791	Tetragenococcus halophilus
2792	Tetragenococcus muriaticus
2793	Tetrahymena thermophila
2794	Tetrasphaera elongata
2795	Thalassiobium sp,
2796	Thalassobacter arenae
2797	Thalassobaculum salexigens
2798	Thalassospira lucentensis
2799	Thauera linaloolentis
2800	Thauera sp,
2801	Thauera terpenica
2802	Theobroma cacao
2803	Thermacetogenium phaeum
2804	Thermaerobacter marianensis
2805	Thermaerobacter subterraneus
2806	Thermanaerovibrio acidaminovorans
2807	Thermanaerovibrio velox
2808	Thermicanus aegyptius
2809	Thermincola potens
2810	Thermithiobacillus tepidarius
2811	Thermoanaerobacter brockii
2812	Thermoanaerobacter indiensis
2813	Thermoanaerobacter italicus
2814	Thermoanaerobacter kivui
2815	Thermoanaerobacter mathranii
2816	Thermoanaerobacter siderophilus
2817	Thermoanaerobacter sp,
2818	Thermoanaerobacterium saccharolyticum
2819	Thermoanaerobacterium thermosaccharolyticum
2820	Thermoanaerobacterium xylanolyticum
2821	Thermobacillus composti
2822	Thermobaculum terrenum
2823	Thermobifida fusca
2824	Thermobispora bispora
2825	Thermobrachium celere
2826	Thermococcus gammatolerans
2827	Thermococcus onnurineus
2828	Thermococcus sp,
2829	Thermodesulfobacterium geofontis
2830	Thermodesulfobium narugense
2831	Thermodesulfovibrio yellowstonii
2832	Thermomicrobium roseum
2833	Thermomonas fusca
2834	Thermomonospora curvata
2835	Thermoplasmatales archaeon
2836	Thermoproteus tenax
2837	Thermosediminibacter oceani
2838	Thermosipho africanus
2839	Thermosipho melanesiensis
2840	Thermosynechococcus sp,
2841	Thermotalea metallivorans
2842	Thermotoga hypogea
2843	Thermotoga maritima
2844	Thermotoga naphthophila
2845	Thermotoga neapolitana
2846	Thermotoga petrophila
2847	Thermovirga lienii
2848	Thermus antranikianii
2849	Thermus igniterrae
2850	Thermus oshimai
2851	Thermus scotoductus
2852	Thermus sp,
2853	Thermus thermophilus
2854	Thielavia terrestris
2855	Thioalkalivibrio nitratireducens
2856	Thioalkalivibrio sp,
2857	Thioalkalivibrio sulfidophilus
2858	Thioalkalivibrio thiocyanodenitrificans
2859	Thioalkalivibrio thiocyanoxidans
2860	Thiobacillus denitrificans
2861	Thiobacillus thioparus
2862	Thioflavicoccus mobilis
2863	Thiomicrospira chilensis
2864	Thiomicrospira crunogena
2865	Thiomicrospira halophila
2866	Thiomicrospira pelophila
2867	Thiomicrospira sp,
2868	Thiomonas intermedia
2869	Thiomonas sp,
2870	Thiorhodovibrio sp,
2871	Thiothrix disciformis
2872	Thiothrix flexilis
2873	Thiothrix nivea
2874	Tistrella mobilis
2875	Tolumonas auensis
2876	Tolumonas sp,
2877	Tomitella biformata
2878	Torulaspora delbrueckii
2879	Toxoplasma gondii
2880	Treponema azotonutricium
2881	Treponema brennaborense
2882	Treponema bryantii
2883	Treponema denticola
2884	Treponema lecithinolyticum
2885	Treponema maltophilum
2886	Treponema medium
2887	Treponema pallidum
2888	Treponema pedis
2889	Treponema phagedenis
2890	Treponema primitia
2891	Treponema socranskii
2892	Treponema sp,
2893	Treponema succinifaciens

2894	Treponema vincentii
2895	Tribolium castaneum
2896	Trichechus manatus
2897	Trichinella spiralis
2898	Trichoderma reesei
2899	Trichodesmium erythraeum
2900	Trichomonas vaginalis
2901	Truepera radiovictrix
2902	Trueperella pyogenes
2903	Trypanosoma cruzi
2904	Tuberibacillus calidus
2905	Tumebacillus ginsengisoli
2906	Tupaia chinensis
2907	Turicella otitidis
2908	Turneriella parva
2909	Tursiops truncatus
2910	Uliginosibacterium gangwonense
2911	Uncinocarpus reesii
2912	uncultured archaeon
2913	Uncultured bacterium
2914	Uncultured Termite
2915	Ureaplasma canigenitalium
2916	Ureaplasma urealyticum
2917	Ureibacillus thermosphaericus
2918	Ursus maritimus
2919	Vanderwaltozyma polyspora
2920	Variovorax paradoxus
2921	Variovorax sp,
2922	Verminephrobacter aporrectodeae
2923	Verminephrobacter eiseniae
2924	Verrucomicrobia bacterium
2925	Verrucomicrobiae bacterium
2926	Verrucomicrobium sp,
2927	Verrucomicrobium spinosum
2928	Verrucosispora maris
2929	Verticillium albo-atrum
2930	Vibrio alginolyticus
2931	Vibrio angustum
2932	Vibrio cholerae
2933	Vibrio coralliilyticus
2934	Vibrio crassostreae
2935	Vibrio cyclitrophicus
2936	Vibrio furnissii
2937	Vibrio genomosp,
2938	Vibrio harveyi
2939	Vibrio litoralis
2940	Vibrio mimicus
2941	Vibrio nigripulchritudo
2942	VIbrio owensii
2943	Vibrio parahaemolyticus
2944	Vibrio sp,
2945	Vibrio splendidus
2946	Vibrio tasmaniensis
2947	Vibrio vulnificus
2948	Vicugna pacos
2949	Virgibacillus alimentarius
2950	Virgibacillus halodenitrificans
2951	Virgibacillus sp,

2952	Vitis rotundifolia
2953	Vitis vinifera
2954	Volvox carteri
2955	Waddlia chondrophila
2956	Weissella oryzae
2957	Wenxinia marina
2958	Williamsia sp,
2959	Wohlfahrtiimonas chitiniclastica
2960	Wolbachia endosymbiont
2961	Wolinella succinogenes
2962	Xanthobacter autotrophicus
2963	Xanthobacter sp,
2964	Xanthobacteraceae bacterium
2965	Xanthomonadaceae bacterium
2966	Xanthomonas albilineans
2967	Xanthomonas arboricola
2968	Xanthomonas axonopodis
2969	Xanthomonas campestris
2970	Xanthomonas cassavae
2971	Xanthomonas citri
2972	Xanthomonas fuscans
2973	Xanthomonas hortorum
2974	Xanthomonas oryzae
2975	Xanthomonas sacchari
2976	Xanthomonas sp,
2977	Xanthomonas translucens
2978	Xanthomonas vasicola
2979	Xenopus (Silurana)
2980	Xenorhabdus bovienii
2981	Xenorhabdus nematophila
2982	Xiphophorus maculatus
2983	Xylanimonas cellulosilytica
2984	Yersinia pestis
2985	Yokenella regensburgei
2986	Zavarzinella formosa
2987	Zea mays
2988	Zeta proteobacterium
2989	Zimmermannella faecalis
2990	Zonotrichia albicollis
2991	Zooshikella ganghwensis
2992	Zunongwangia profunda
2993	Zygnema circumcarinatum
2994	Zymobacter palmae
2995	Zymomonas mobilis
2996	Zymophilus raffinosivorans

Table S29 : The taxonomic profile predicted from the assembly results of MetaVelvet-SL using BLAST for the MH0047 dataset.

No	Species
1	Alistipes putredinis
2	Alistipes shahii
3	Anaerotruncus colihominis
4	Bacteroides caccae
5	Bacteroides cellulosilyticus
6	Bacteroides coprocola
7	Bacteroides coprophilus
8	Bacteroides dorei
9	Bacteroides eggerthii
10	Bacteroides finegoldii
11	Bacteroides fragilis
12	Bacteroides ovatus
13	Bacteroides plebeius
14	Bacteroides salanitronis
15	Bacteroides stercoris
16	Bacteroides unclassified
17	Bacteroides uniformis
18	Bacteroides vulgatus
19	Bacteroides xylanisolvens
20	Bifidobacterium adolescentis
21	Bifidobacterium longum
22	Bilophila wadsworthia
23	Blautia unclassified
24	Butyrivibrio unclassified
25	Clostridium asparagiforme
26	Clostridium bartlettii
27	Clostridium cf
28	Clostridium leptum
29	Clostridium nexile
30	Collinsella aerofaciens
31	Coprococcus catus
32	Coprococcus comes
33	Desulfovibrio desulfuricans
34	Desulfovibrio piger
35	Dorea formicigenerans
36	Dorea unclassified
37	Escherichia coli
38	Eubacterium hallii
39	Eubacterium rectale
40	Eubacterium siraeum
41	Faecalibacterium cf
42	Faecalibacterium prausnitzii
43	Faecalibacterium unclassified
44	Holdemania filiformis
45	Odoribacter splanchnicus
46	Parabacteroides distasonis
47	Parabacteroides johnsonii
48	Parabacteroides merdae
49	Parabacteroides unclassified
50	Phascolarctobacterium unclassified
51	Prevotella buccae
52	Pseudoflavonifractor capillosus
53	Roseburia intestinalis
54	Roseburia inulinivorans
55	Ruminococcus bromii
56	Ruminococcus gnavus
57	Ruminococcus obeum
58	Ruminococcus torques
59	Streptococcus mutans
60	Streptococcus parasanguinis
61	Streptococcus salivarius
62	Subdoligranulum variabile
63	Veillonella atypica
64	Veillonella dispar
65	Veillonella unclassified
66	[Bacillus] schlegelii
67	[Clostridium] aerotolerans
68	[Clostridium] aminophilum
69	[Clostridium] bifermentans
70	[Clostridium] cellobioparum
71	[Clostridium] citroniae
72	[Clostridium] clostridioforme
73	[Clostridium] indolis
74	[Clostridium] josui
75	[Clostridium] mangenotii
76	[Clostridium] methoxybenzovorans
77	[Clostridium] papyrosolvens
78	[Clostridium] saccharogumia
79	[Clostridium] sordellii
80	[Clostridium] sporosphaeroides
81	[Clostridium] sticklandii,
82	[Clostridium] termitidis
83	[Clostridium] thermosuccinogenes
84	[Clostridium] viride
85	[Eubacterium] cellulosolvens
86	[Eubacterium] minutum
87	Abiotrophia defectiva
88	Acanthamoeba castellanii
89	Acanthisitta chloris
90	Acaricomes phytoseiuli
91	Acetanaerobacterium elongatum
92	Acetivibrio cellulolyticus
93	Acetobacter nitrogenifigens
94	Acetobacter pasteurianus
95	Acetobacter sp.
96	Acetobacterium dehalogenans
97	Acetobacterium woodii
98	Acholeplasma axanthum
99	Acholeplasma modicum
100	Acholeplasma sp.
101	Achromobacter piechaudii
102	Achromobacter xylosoxidans
103	Acidaminococcus fermentans
104	Acidaminococcus sp.
105	Acidimicrobidae bacterium
106	Acidimicrobium ferrooxidans
107	Acidiphilium angustum
108	Acidiphilium multivorum
109	Acidiphilium sp.
110	Acidithiobacillus caldus

111	Acidithiobacillus ferrivorans
112	Acidithiobacillus ferrooxidans
113	Acidithiobacillus thiooxidans
114	Acidobacteria bacterium
115	Acidobacteriaceae bacterium
116	Acidobacterium capsulatum
117	Acidobacterium sp.
118	Acidocella facilis
119	Acidovorax avenae
120	Acidovorax citrulli
121	Acidovorax ebreus
122	Acidovorax oryzae
123	Acidovorax radicis
124	Acidovorax sp.
125	Acinetobacter baumannii
126	Acinetobacter bereziniae
127	Acinetobacter bouvetii
128	Acinetobacter gerneri
129	Acinetobacter guillouiae
130	Acinetobacter johnsonii
131	Acinetobacter lwoffii
132	Acinetobacter parvus
133	Acinetobacter sp.
134	Acinetobacter venetianus
135	Actinoalloteichus spitiensis
136	Actinobacillus succinogenes
137	Actinobacillus ureae
138	Actinobacterium SCGC
139	Actinobaculum massiliae
140	Actinobaculum schaalii
141	Actinocatenispora sera
142	Actinokineospora enzanensis
143	Actinokineospora inagensis
144	Actinomadura atramentaria
145	Actinomadura flavalba
146	Actinomyces cardiffensis
147	Actinomyces dentalis
148	Actinomyces georgiae
149	Actinomyces gerencseriae
150	Actinomyces johnsonii
151	Actinomyces massiliensis
152	Actinomyces neuii
153	Actinomyces sp.
154	Actinomyces timonensis
155	Actinomyces turicensis
156	Actinomyces vaccimaxillae
157	Actinoplanes missouriensis
158	Actinoplanes sp.
159	Actinoplanes subtropicus
160	Acyrthosiphon pisum
161	Adhaeribacter aquaticus
162	Adlercreutzia equolifaciens
163	Advenella kashmirensis
164	Aedes aegypti
165	Aequorivita sublithincola
166	Aerococcus urinae
167	Aerococcus viridans
168	Aeromonas caviae
169	Aeromonas enteropelogenes
170	Aeromonas hydrophila
171	Aeromonas salmonicida
172	Aeromonas sp.
173	Aeromonas veronii
174	Aestuariimicrobium kwangyangense
175	Afifella pfennigii
176	Afipia broomeae
177	Afipia clevelandensis
178	Afipia sp.
179	Aggregatibacter actinomycetemcomitans
180	Aggregatibacter aphrophilus
181	Aggregatibacter segnis
182	Agrobacterium fabrum
183	Agrobacterium radiobacter
184	Agrobacterium sp.
185	Agrobacterium tumefaciens
186	Agrobacterium vitis
187	Ahrensia sp.
188	Ailuropoda melanoleuca
189	Ajellomyces capsulatus
190	Akkermansia muciniphila
191	Akkermansia muciniphila,
192	Akkermansia sp.
193	Alcaligenes sp.
194	Alcanivorax dieselolei
195	Alcanivorax sp.
196	Algoriphagus mannitolivorans
197	Algoriphagus marincola
198	Algoriphagus terrigena
199	Aliagarivorans marinus
200	Alicycliphilus denitrificans
201	Alicyclobacillus acidocaldarius
202	Alicyclobacillus herbarius
203	Alicyclobacillus macrosporangiidus
204	Alicyclobacillus pohliae
205	Alicyclobacillus pomorum
206	Alistipes finegoldii
207	Alistipes indistinctus
208	Alistipes onderdonkii
209	Alistipes senegalensis
210	Alistipes sp.
211	Alistipes timonensis
212	Alkalibacillus haloalkaliphilus
213	Alkalibaculum bacchi
214	Alkaliflexus imshenetskii
215	Alkaliphilus metalliredigens
216	Alkaliphilus transvaalensis
217	Alkanindiges illinoisensis
218	Alligator sinensis
219	Allobaculum stercoricanis
220	Allochromatium vinosum
221	Allokutzneria albata
222	Alloprevotella rava
223	Alpha proteobacterium
224	Amborella trichopoda
225	Aminicenantes bacterium
226	Aminobacter sp.

227	Aminomonas paucivorans
228	Amorphus coralli
229	Amphibacillus jilinensis
230	Amphibacillus xylanus
231	Amphimedon queenslandica
232	Amphritea japonica
233	Amycolatopsis balhimycina
234	Amycolatopsis mediterranei
235	Amycolatopsis methanolica
236	Amycolatopsis orientalis
237	Amycolatopsis taiwanensis
238	Amycolatopsis thermoflava
239	Anaerobacterium chartisolvens
240	Anaerobaculum mobile
241	Anaerococcus obesiensis
242	Anaerococcus prevotii
243	Anaerococcus vaginalis
244	Anaerofilum agile
245	Anaerolinea thermophila
246	Anaeromusa acidaminophila
247	Anaeromyxobacter dehalogenans
248	Anaeromyxobacter sp.
249	Anaerophaga thermohalophila
250	Anaerostipes caccae
251	Anaerostipes hadrus
252	Anaerotruncus sp.
253	Anaerovibrio sp.
254	Anaerovorax odorimutans
255	Anas platyrhynchos
256	Anolis carolinensis
257	Anoxybacillus flavithermus
258	Anoxybacillus kamchatkensis
259	Anoxybacillus tepidamans
260	Apis dorsata
261	Apis florea
262	Aplysia californica
263	Aptenodytes forsteri
264	Aquifex aeolicus
265	Aquimarina agarilytica
266	Aquimarina megaterium
267	Aquimarina sp.
268	Archaeoglobus fulgidus
269	Arcobacter sp.
270	Arenimonas composti
271	Arhodomonas aquaeolei
272	Armatimonadetes bacterium
273	Aromatoleum aromaticum
274	Arthrobacter chlorophenolicus
275	Arthrobacter nicotinovorans
276	Arthrobacter phenanthrenivorans
277	Arthrobacter sp.
278	Asaia platycodi
279	Asaia prunellae
280	Ashbya gossypii
281	Asticcacaulis benevestitus
282	Asticcacaulis biprosthecum
283	Asticcacaulis excentricus
284	Asticcacaulis sp.
285	Astyanax mexicanus
286	Atopobium fossor
287	Atopobium minutum
288	Atopobium vaginae
289	Aurantimonas coralicida
290	Aureococcus anophagefferens
291	Azoarcus sp.
292	Azoarcus toluclasticus
293	Azohydromonas australica
294	Azonexus hydrophilus
295	Azorhizobium caulinodans
296	Azorhizobium doebereinerae
297	Azospirillum brasilense
298	Azospirillum halopraeferens
299	Azospirillum lipoferum
300	Azospirillum sp.
301	Azotobacter vinelandii
302	Azovibrio restrictus
303	Bacillus acidiproducens
304	Bacillus aidingensis
305	Bacillus amyloliquefaciens
306	Bacillus atrophaeus
307	Bacillus bogoriensis
308	Bacillus cereus
309	Bacillus chagannorensis
310	Bacillus coagulans
311	Bacillus coahuilensis
312	Bacillus cytotoxicus
313	Bacillus fordii
314	Bacillus gelatini
315	Bacillus kribbensis
316	Bacillus licheniformis
317	Bacillus marmarensis
318	Bacillus megaterium
319	Bacillus mojavensis
320	Bacillus oceanisediminis
321	Bacillus panaciterrae
322	Bacillus pumilus
323	Bacillus selenitireducens
324	Bacillus siamensis
325	Bacillus smithii
326	Bacillus sp.
327	Bacillus subtilis
328	Bacillus toyonensis
329	Bacillus vallismortis
330	Bacterium JKG1
331	Bacteroidales bacterium
332	Bacteroidetes oral
333	Balaenoptera acutorostrata
334	Balneatrix alpica
335	Barnesiella intestinihominis
336	Barnesiella viscericola
337	Bartonella bacilliformis
338	Bartonella clarridgeiae
339	Bartonella elizabethae
340	Bathycoccus prasinos
341	Baudoinia compniacensis
342	Bavariicoccus seileri

343	Beijerinckia indica
344	Belliella baltica
345	Beta proteobacterium
346	Beutenbergia cavernae
347	Bifidobacterium angulatum
348	Bifidobacterium animalis
349	Bifidobacterium asteroides
350	Bifidobacterium bifidum
351	Bifidobacterium boum
352	Bifidobacterium breve
353	Bifidobacterium dentium
354	Bifidobacterium minimum
355	Bifidobacterium pseudocatenulatum
356	Bifidobacterium pseudolongum
357	Bifidobacterium ruminantium
358	Bifidobacterium sp.
359	Bifidobacterium subtile
360	Bifidobacterium thermacidophilum
361	Bifidobacterium thermophilum
362	Bifidobacterium tsurumiense
363	Bilophila sp.
364	Blastococcus saxobsidens
365	Blastomonas sp.
366	Blastopirellula marina
367	Blattabacterium sp.
368	Bordetella avium
369	Bordetella bronchiseptica
370	Bordetella hinzii
371	Bordetella holmesii
372	Bordetella petrii
373	Bordetella sp.
374	Bordetella trematum
375	Bos mutus
376	Bos taurus
377	Botryotinia fuckeliana
378	Brachybacterium faecium
379	Brachybacterium muris
380	Brachymonas chironomi
381	Brachypodium distachyon
382	Brachyspira alvinipulli
383	Brachyspira intermedia
384	Brachyspira pilosicoli
385	Brachyspira sp.
386	Bradyrhizobium elkanii
387	Bradyrhizobium genosp.
388	Bradyrhizobium japonicum
389	Bradyrhizobium sp.
390	Branchiostoma floridae
391	Brassica rapa
392	BRC1 bacterium
393	Brevibacillus agri
394	Brevibacillus borstelensis
395	Brevibacillus brevis
396	Brevibacillus massiliensis,
397	Brevibacillus panacihumi
398	Brevibacillus thermoruber
399	Brevibacterium album
400	Brevibacterium sp.

401	Brevundimonas aveniformis
402	Brevundimonas diminuta
403	Brochothrix thermosphacta
404	Brucella abortus
405	Brucella pinnipedialis
406	Brucella sp.
407	Bryobacter aggregatus
408	Bubalus bubalis
409	Buchnera aphidicola
410	Burkholderia acidipaludis
411	Burkholderia andropogonis
412	Burkholderia bannensis
413	Burkholderia caledonica
414	Burkholderia cenocepacia
415	Burkholderia cepacia
416	Burkholderia dilworthii
417	Burkholderia dolosa
418	Burkholderia ferrariae
419	Burkholderia fungorum
420	Burkholderia gladioli
421	Burkholderia glumae
422	Burkholderia kururiensis
423	Burkholderia mallei
424	Burkholderia mimosarum
425	Burkholderia nodosa
426	Burkholderia oklahomensis
427	Burkholderia oxyphila
428	Burkholderia phenoliruptrix
429	Burkholderia phymatum
430	Burkholderia phytofirmans
431	Burkholderia pseudomallei
432	Burkholderia pyrrocinia
433	Burkholderia rhizoxinica
434	Burkholderia sordidicola
435	Burkholderia sp.
436	Burkholderia sprentiae
437	Burkholderia thailandensis
438	Burkholderia ubonensis
439	Burkholderia xenovorans
440	Burkholderiales bacterium
441	Butyrate-producing bacterium
442	Butyricicoccus pullicaecorum
443	Butyricimonas synergistica
444	Butyricimonas virosa
445	Caenorhabditis elegans
446	Caldanaerobacter subterraneus
447	Caldanaerobius polysaccharolyticus
448	Caldibacillus debilis
449	Caldicellulosiruptor lactoaceticus
450	Caldicellulosiruptor owensensis
451	Caldicoprobacter oshimai
452	Caldimonas manganoxidans
453	Calditerrivibrio nitroreducens
454	Caldithrix abyssi
455	Calescamantes bacterium
456	Callithrix jacchus
457	Callorhinchus milii
458	Caloramator sp.

459	Calothrix sp.
460	Calypte anna
461	Camelus ferus
462	Campylobacter coli
463	Campylobacter curvus
464	Campylobacter fetus
465	Campylobacter hominis
466	Campylobacter jejuni
467	Campylobacter upsaliensis
468	Campylobacterales bacterium
469	Candidatus Accumulibacter
470	Candidatus Alistipes
471	Candidatus Arthromitus
472	Candidatus Burkholderia
473	Candidatus Carsonella
474	Candidatus Chloracidobacterium
475	Candidatus Hydrogenedens
476	Candidatus Kinetoplastibacterium
477	Candidatus Methylomirabilis
478	Candidatus Nitrospira
479	Candidatus Pelagibacter
480	Candidatus Phytoplasma
481	Candidatus Regiella
482	Candidatus Riesia
483	Candidatus Saccharibacteria
484	Candidatus Solibacter
485	Candidatus Stoquefichus
486	Canis lupus
487	Capnocytophaga canimorsus
488	Capnocytophaga ochracea
489	Capnocytophaga sp.
490	Capra hircus
491	Capsicum annuum
492	Cardiobacterium hominis
493	Cardiobacterium valvarum
494	Carnobacterium jeotgali
495	Carnobacterium maltaromaticum
496	Catabacter hongkongensis
497	Catellibacterium nectariphilum
498	Catelliglobosispora koreensis
499	Catenibacterium sp.
500	Catenulispora acidiphila
501	Catonella morbi
502	Caulobacter segnis
503	Caulobacter sp.
504	Caulobacteraceae bacterium
505	Cavia porcellus
506	Cedecea davisae
507	Cellulomonas fimi
508	Cellulomonas flavigena
509	Cellulomonas massiliensis
510	Cellulomonas sp.
511	Cellulophaga algicola
512	Cellulophaga baltica
513	Cellvibrio japonicus
514	Centipeda periodontii
515	Ceratitis capitata
516	Ceratotherium simum

517	Chaetomium globosum
518	Chamaesiphon minutus
519	Chelativorans sp.
520	Chelatococcus sp.
521	Chelonia mydas
522	Chinchilla lanigera
523	Chitinibacter tainanensis
524	Chitinilyticum aquatile
525	Chitinilyticum litopenaei
526	Chitinimonas koreensis
527	Chitiniphilus shinanonensis
528	Chitinophaga pinensis
529	Chlamydomonas reinhardtii
530	Chlorella variabilis
531	Chlorobaculum parvum
532	Chlorobium limicola
533	Chlorobium luteolum
534	Chlorobium phaeobacteroides
535	Chlorobium tepidum
536	Chlorocebus sabaeus
537	Chloroflexi bacterium
538	Chloroflexus aggregans
539	Chloroflexus sp.
540	Chloroherpeton thalassium
541	Choloepus hoffmanni
542	Christensenella minuta
543	Chromobacterium violaceum
544	Chromohalobacter salexigens
545	Chroococcidiopsis thermalis
546	Chrysemys picta
547	Chryseobacterium caeni
548	Chryseobacterium daeguense
549	Chryseobacterium gregarium
550	Chryseobacterium haifense
551	Chryseobacterium taeanense
552	Chrysochloris asiatica
553	Cicer arietinum
554	Citreicella sp.
555	Citrobacter freundii
556	Citrobacter koseri
557	Citrobacter rodentium
558	Citrobacter sp.
559	Citromicrobium sp.
560	Citrus clementina
561	Clavibacter michiganensis
562	Clostridiales bacterium
563	Clostridiales genomosp.
564	Clostridium acetobutylicum
565	Clostridium algidicarnis
566	Clostridium autoethanogenum
567	Clostridium beijerinckii
568	Clostridium bolteae
569	Clostridium botulinum
570	Clostridium butyricum
571	Clostridium cadaveris
572	Clostridium carboxidivorans
573	Clostridium celatum
574	Clostridium cellulolyticum

575	Clostridium cellulovorans
576	Clostridium chartatabidum
577	Clostridium citroniae
578	Clostridium clariflavum
579	Clostridium clostridioforme
580	Clostridium colicanis
581	Clostridium difficile
582	Clostridium disporicum
583	Clostridium glycolicum
584	Clostridium hathewayi
585	Clostridium hiranonis
586	Clostridium hydrogeniformans
587	Clostridium hylemonae
588	Clostridium innocuum
589	Clostridium intestinale
590	Clostridium kluyveri
591	Clostridium lentocellum
592	Clostridium ljungdahlii
593	Clostridium lundense
594	Clostridium methylpentosum
595	Clostridium novyi
596	Clostridium paraputrificum
597	Clostridium pasteurianum
598	Clostridium perfringens
599	Clostridium phytofermentans
600	Clostridium ramosum
601	Clostridium saccharobutylicum
602	Clostridium saccharolyticum
603	Clostridium saccharoperbutylacetonicum
604	Clostridium scindens
605	Clostridium senegalense
606	Clostridium sp.
607	Clostridium spiroforme
608	Clostridium sporogenes
609	Clostridium sporosphaeroides
610	Clostridium stercorarium
611	Clostridium symbiosum
612	Clostridium tetani
613	Clostridium thermocellum
614	Clostridium tyrobutyricum
615	Cohnella laeviribosi
616	Cohnella panacarvi
617	Cohnella thermotolerans
618	Colletotrichum gloeosporioides
619	Collinsella intestinalis
620	Collinsella sp.
621	Collinsella stercoris
622	Collinsella tanakaei
623	Columba livia
624	Comamonadaceae bacterium
625	Comamonas badia
626	Comamonas composti
627	Comamonas testosteroni
628	complete chromosome
629	Conchiformibius kuhniae
630	Conchiformibius steedae
631	Condylura cristata
632	Conexibacter woesei

633	Congregibacter litoralis
634	Coprobacillus sp.
635	Coprobacter fastidiosus
636	Coprococcus eutactus
637	Coprococcus sp.
638	Coraliomargarita akajimensis
639	Coraliomargarita sp.
640	Corallococcus coralloides
641	Corallococcus sp.
642	Coriobacteriaceae bacterium
643	Coriobacterium glomerans
644	Corynebacterium bovis
645	Corynebacterium callunae
646	Corynebacterium doosanense
647	Corynebacterium durum
648	Corynebacterium jeikeium
649	Corynebacterium kroppenstedtii
650	Corynebacterium massiliense
651	Corynebacterium mastitidis
652	Corynebacterium matruchotii
653	Corynebacterium pseudodiphtheriticum
654	Corynebacterium sp.
655	Corynebacterium ulcerans
656	Cricetulus griseus
657	Cronobacter pulveris
658	Cronobacter sakazakii
659	Cronobacter sp.
660	Cronobacter turicensis
661	Cryptobacterium curtum
662	Cryptobacterium sp.
663	Cryptococcus gattii
664	Cryptococcus neoformans
665	Cucumibacter marinus
666	Cucumis melo
667	Culex pipiens
668	Cupriavidus metallidurans
669	Cupriavidus necator
670	Cupriavidus sp.
671	Cupriavidus taiwanensis
672	Curtobacterium sp.
673	Curvibacter gracilis
674	Curvibacter lanceolatus
675	Cyanothece sp.
676	Cynoglossus semilaevis
677	Cytophaga aurantiaca
678	Cytophaga fermentans
679	Cytophaga hutchinsonii
680	Cytophagaceae bacterium
681	Cytophagales bacterium
682	Danio rerio
683	Dasypus novemcinctus
684	Dechloromonas agitata
685	Dechloromonas aromatica
686	Dechlorosoma suillum
687	Deefgea rivuli
688	Deferrisoma camini
689	Defluviitalea saccharophila
690	Dehalobacter sp.

691	Dehalococcoides ethenogenes
692	Dehalococcoides mccartyi
693	Dehalogenimonas lykanthroporepellens
694	Deinococcus apachensis
695	Deinococcus aquatilis
696	Deinococcus ficus
697	Deinococcus frigens
698	Deinococcus gobiensis
699	Deinococcus maricopensis
700	Deinococcus marmoris
701	Deinococcus murrayi
702	Deinococcus peraridilitoris
703	Deinococcus pimensis
704	Deinococcus proteolyticus
705	Deinococcus sp.
706	Deinococcus wulumuqiensis
707	Delftia acidovorans
708	Delftia sp.
709	Delta proteobacterium
710	Derxia gummosa
711	Desmospora sp.
712	Desulfarculus baarsii
713	Desulfatibacillum aliphaticivorans
714	Desulfatibacillum alkenivorans
715	Desulfatirhabdium butyrativorans
716	Desulfitobacterium dichloroeliminans
717	Desulfitobacterium hafniense
718	Desulfitobacterium sp.
719	Desulfobacter curvatus
720	Desulfobacter postgatei
721	Desulfobacterium anilini
722	Desulfobacula sp.
723	Desulfobacula toluolica
724	Desulfobulbus elongatus
725	Desulfobulbus propionicus
726	Desulfocapsa sulfexigens
727	Desulfococcus oleovorans
728	Desulfocurvus vexinensis
729	Desulfohalobium retbaense
730	Desulfomicrobium escambiense
731	Desulfonatronum lacustre
732	Desulfonatronum thiodismutans
733	Desulfosarcina sp.
734	Desulfospira joergensenii
735	Desulfosporosinus acidiphilus
736	Desulfosporosinus meridiei
737	Desulfosporosinus youngiae
738	Desulfotignum balticum
739	Desulfotomaculum alcoholivorax
740	Desulfotomaculum alkaliphilum
741	Desulfotomaculum carboxydivorans
742	Desulfotomaculum gibsoniae
743	Desulfotomaculum kuznetsovii
744	Desulfotomaculum nigrificans
745	Desulfotomaculum ruminis
746	Desulfovibrio aespoeensis
747	Desulfovibrio africanus
748	Desulfovibrio alaskensis

749	Desulfovibrio alcoholivorans
750	Desulfovibrio aminophilus
751	Desulfovibrio cf.
752	Desulfovibrio cuneatus
753	Desulfovibrio gigas
754	Desulfovibrio hydrothermalis
755	Desulfovibrio inopinatus
756	Desulfovibrio longus
757	Desulfovibrio magneticus
758	Desulfovibrio oxyclinae
759	Desulfovibrio piezophilus
760	Desulfovibrio putealis
761	Desulfovibrio sp.
762	Desulfovibrio vulgaris
763	Desulfovibrio zosterae
764	Desulfovirgula thermocuniculi
765	Desulfurispirillum indicum
766	Desulfurispora thermophila
767	Desulfurivibrio alkaliphilus
768	Desulfurococcus fermentans
769	Desulfuromonas sp.
770	Dialister invisus
771	Dialister sp.
772	Dialister succinatiphilus
773	Dickeya dadantii
774	Dickeya dianthicola
775	Dickeya paradisiaca
776	Dickeya solani
777	Dielma fastidiosa
778	Dietzia alimentaria
779	Dietzia sp.
780	Dinoroseobacter shibae
781	Diplorickettsia massiliensis
782	Donghicola xiamenensis
783	Dongia sp.
784	Drosophila ananassae
785	Drosophila erecta
786	Drosophila grimshawi
787	Drosophila persimilis
788	Drosophila pseudoobscura
789	Drosophila sechellia
790	Drosophila virilis
791	Drosophila willistoni
792	Drosophila yakuba
793	Duganella violaceinigra
794	Duganella zoogloeoides
795	Dyadobacter alkalitolerans
796	Dyadobacter beijingensis
797	Dyadobacter fermentans
798	Dyadobacter tibetensis
799	Dyella ginsengisoli
800	Dyella japonica
801	Dysgonomonas capnocytophagoides
802	Dysgonomonas gadei
803	Dysgonomonas mossii
804	Echinicola pacifica
805	Echinicola vietnamensis
806	Echinops telfairi

807	Edwardsiella ictaluri
808	Edwardsiella tarda
809	Eggerthella lenta
810	Eggerthella sp.
811	Eggerthia catenaformis
812	Eikenella corrodens
813	Elephantulus edwardii
814	Elioraea tepidiphila
815	Elizabethkingia anophelis
816	Elizabethkingia meningoseptica
817	Elusimicrobium minutum
818	Emiliania huxleyi
819	Empedobacter brevis
820	Emticicia oligotrophica
821	Endozoicomonas elysicola
822	Ensifer adhaerens
823	Ensifer sp.
824	Entamoeba dispar
825	Entamoeba histolytica
826	Entamoeba invadens
827	Enterobacter aerogenes
828	Enterobacter asburiae
829	Enterobacter cancerogenus
830	Enterobacter cloacae
831	Enterobacter hormaechei
832	Enterobacter mori
833	Enterobacter radicincitans
834	Enterobacter sp.
835	Enterobacteriaceae bacterium
836	Enterococcus asini
837	Enterococcus caccae
838	Enterococcus cecorum
839	Enterococcus columbae
840	Enterococcus durans
841	Enterococcus faecalis
842	Enterococcus faecium
843	Enterococcus gilvus
844	Enterococcus haemoperoxidus
845	Enterococcus mundtii
846	Enterococcus pallens
847	Enterococcus phoeniculicola
848	Enterococcus raffinosus
849	Enterococcus villorum
850	Enterorhabdus caecimuris
851	Enterorhabdus mucosicola
852	Entomoplasma melaleucae
853	Epilithonimonas tenax
854	Eptesicus fuscus
855	Equus caballus
856	Equus przewalskii
857	Erinaceus europaeus
858	Erwinia billingiae
859	Erwinia pyrifoliae
860	Erwinia toletana
861	Erysipelotrichaceae bacterium
862	Erythrobacter litoralis
863	Erythrobacter sp.
864	Escherichia albertii

865	Escherichia fergusonii
866	Escherichia sp.
867	Ethanoligenens harbinense
868	Eubacteriaceae bacterium
869	Eubacterium biforme
870	Eubacterium brachy
871	Eubacterium cellulosolvens
872	Eubacterium coprostanoligenes
873	Eubacterium cylindroides
874	Eubacterium desmolans
875	Eubacterium dolichum
876	Eubacterium eligens
877	Eubacterium limosum
878	Eubacterium plexicaudatum
879	Eubacterium ramulus
880	Eubacterium saburreum
881	Eubacterium sp.
882	Eubacterium ventriosum
883	Eubacterium xylanophilum
884	Eubacterium yurii
885	Eutrema salsugineum
886	Eutypa lata
887	Exiguobacterium antarcticum
888	Exiguobacterium aurantiacum
889	Exiguobacterium pavilionensis
890	Exiguobacterium sibiricum
891	Exiguobacterium sp.
892	Exophiala dermatitidis
893	Facklamia ignava
894	Falco cherrug
895	Fangia hongkongensis
896	Felis catus
897	Ferrimonas balearica
898	Ferrimonas futtsuensis
899	Ferrimonas kyonanensis
900	Ferrimonas senticii
901	Ferroplasma acidarmanus
902	Ferrovum myxofaciens
903	Fervidibacteria bacterium
904	Fibrella aestuarina
905	Fibrobacter succinogenes
906	Ficedula albicollis
907	Finegoldia magna
908	Firmicutes bacterium
909	Flavobacteriaceae bacterium
910	Flavobacterium anhuiense
911	Flavobacterium antarcticum
912	Flavobacterium chungangense
913	Flavobacterium daejeonense
914	Flavobacterium denitrificans
915	Flavobacterium indicum
916	Flavobacterium psychrophilum
917	Flavobacterium soli
918	Flavobacterium sp.
919	Flavobacterium subsaxonicum
920	Flavobacterium succinicans
921	Flavonifractor plautii
922	Flectobacillus major

923	Flexibacter elegans
924	Flexibacter litoralis
925	Flexithrix dorotheae
926	Fluoribacter dumoffii
927	Francisella philomiragia
928	Frankia sp.
929	Frankia symbiont
930	Frateuria aurantia
931	Fusobacterium gonidiaformans
932	Fusobacterium mortiferum
933	Fusobacterium nucleatum
934	Fusobacterium perfoetens
935	Fusobacterium sp.
936	Fusobacterium ulcerans
937	Galeopterus variegatus
938	Gallibacterium anatis
939	Gallionella capsiferriformans
940	Gallus gallus
941	gamma proteobacterium
942	Gamma proteobacterium
943	Gardnerella vaginalis
944	Geitlerinema sp.
945	Gelidibacter mesophilus
946	Gemella bergeriae
947	Gemella moribillum
948	Gemella sanguinis
949	Geminicoccus roseus
950	Geminocystis herdmanii
951	Gemmata obscuriglobus
952	Gemmatimonadetes bacterium
953	Gemmatimonas aurantiaca
954	Gemmobacter nectariphilus
955	Geobacillus caldoxylosilyticus
956	Geobacillus sp.
957	Geobacillus vulcani
958	Geobacter bemidjiensis
959	Geobacter bremensis
960	Geobacter daltonii
961	Geobacter lovleyi
962	Geobacter metallireducens
963	Geobacter sp.
964	Geobacter sulfurreducens
965	Geobacter uraniireducens
966	Geodermatophilaceae bacterium
967	Geospiza fortis
968	Geothrix fermentans
969	Gilvimarinus chinensis
970	Glaciecola nitratireducens
971	Glaciibacter superstes
972	Gloeobacter kilaueensis
973	Glomeribacter sp.
974	Gluconacetobacter diazotrophicus
975	Gluconacetobacter europaeus
976	Gluconacetobacter hansenii
977	Gluconacetobacter sp.
978	Gluconacetobacter xylinus
979	Gluconobacter frateurii
980	Gluconobacter oxydans
981	Glycine max
982	Glycomyces sp.
983	Glycomyces tenuis
984	Gordonia bronchialis
985	Gordonia hirsuta
986	Gordonia kroppenstedtii
987	Gordonia polyisoprenivorans
988	Gordonibacter pamelaeae
989	Gorilla gorilla
990	Gracilibacillus lacisalsi
991	Gracilibacter thermotolerans
992	Gracilimonas tropica
993	Gramella echinicola
994	Gramella forsetii
995	Gramella portivictoriae
996	Granulicatella adiacens
997	Granulicella mallensis
998	Granulicella tundricola
999	Granulicoccus phenolivorans
1000	Guillardia theta
1001	Gulosibacter molinativorax
1002	Haemophilus influenzae
1003	Haemophilus parainfluenzae
1004	Haemophilus parasuis
1005	Haemophilus somnus
1006	Hafnia alvei
1007	Hahella chejuensis
1008	Halanaerobium hydrogeniformans
1009	Halanaerobium praevalens
1010	Haliangium ochraceum
1011	Haliea salexigens
1012	Haliscomenobacter hydrossis
1013	Hallella seregens
1014	Haloarcula marismortui
1015	Halobacteroides halobius
1016	Halobiforma lacisalsi
1017	Halococcus hamelinensis
1018	Haloferax volcanii
1019	Halogeometricum borinquense
1020	Halomicrobium mukohataei
1021	Halomonas alkaliantarctica
1022	Halomonas anticariensis
1023	Halomonas boliviensis
1024	Halomonas jeotgali
1025	Halomonas lutea
1026	Halomonas smyrnensis
1027	Halomonas sp.
1028	Halomonas stevensii
1029	Halomonas zincidurans
1030	Halonatronum saccharophilum
1031	Halophilic archaeon
1032	Halorhodospira halochloris
1033	Halorhodospira halophila
1034	Halorubrum sp.
1035	Halotalea alkalilenta
1036	Haloterrigena turkmenica
1037	Halothiobacillus neapolitanus
1038	Hamadaea tsunoensis

1039	Helcococcus sueciensis
1040	Helicobacter pylori
1041	Heliobacterium modesticaldum
1042	Helobdella robusta
1043	Herbaspirillum lusitanum
1044	Herbaspirillum massiliense
1045	Herbaspirillum rubrisubalbicans
1046	Herbaspirillum seropedicae
1047	Herbidospora cretacea
1048	Herminiimonas arsenicoxydans
1049	Herminiimonas sp.
1050	Heterocephalus glaber
1051	Hevea brasiliensis
1052	Hippea alviniae
1053	Hippea sp.
1054	Hoeflea phototrophica
1055	Hoeflea sp.
1056	Holophaga foetida
1057	Homo sapiens
1058	Humibacter albus
1059	Hydra magnipapillata
1060	Hymenobacter aerophilus
1061	Hymenobacter norwichensis
1062	Hyphomicrobium denitrificans
1063	Hyphomicrobium nitrativorans
1064	Hyphomicrobium zavarzinii
1065	Ichthyophthirius multifiliis
1066	Ideonella sp.
1067	Idiomarina sediminum
1068	Ignavibacterium album
1069	Ignavigranum ruoffiae
1070	Inquilinus limosus
1071	Intestinimonas butyriciproducens
1072	Isoptericola variabilis
1073	Isosphaera pallida
1074	Ixodes scapularis
1075	Jaculus jaculus
1076	Janthinobacterium lividum
1077	Janthinobacterium sp.
1078	Jeotgalicoccus marinus
1079	Jeotgalicoccus psychrophilus
1080	Johnsonella ignava
1081	Jonquetella anthropi
1082	Joostella marina
1083	Kaistia adipata
1084	Kaistia granuli
1085	Kandleria vitulina
1086	Kangiella aquimarina
1087	Kangiella koreensis
1088	Ketogulonigenium vulgarum
1089	Kiloniella laminariae
1090	Kineococcus radiotolerans
1091	Kingella denitrificans
1092	Kingella kingae
1093	Kingella oralis
1094	Kitasatospora setae
1095	Klebsiella oxytoca
1096	Klebsiella pneumoniae
1097	Klebsiella sp.
1098	Kocuria atrinae
1099	Kocuria rhizophila
1100	Kocuria sp.
1101	Kordia algicida
1102	Kordiimonas gwangyangensis
1103	Kozakia baliensis
1104	Kribbella catacumbae
1105	Kribbella flavida
1106	Kushneria aurantia
1107	Kytococcus sedentarius
1108	Labrenzia alexandrii
1109	Labrenzia sp.
1110	Laceyella sacchari
1111	Lachnobacterium bovis
1112	Lachnoclostridium phytofermentans
1113	Lachnospira multipara
1114	Lachnospiraceae bacterium
1115	Lachnospiraceae oral
1116	Lacinutrix sp.
1117	Lactobacillus casei
1118	Lactobacillus delbrueckii
1119	Lactobacillus fructivorans
1120	Lactobacillus gasseri
1121	Lactobacillus harbinensis
1122	Lactobacillus hayakitensis
1123	Lactobacillus helveticus
1124	Lactobacillus iners
1125	Lactobacillus jensenii
1126	Lactobacillus johnsonii
1127	Lactobacillus kisonensis
1128	Lactobacillus namurensis
1129	Lactobacillus reuteri
1130	Lactobacillus rhamnosus
1131	Lactobacillus ruminis
1132	Lactobacillus salivarius
1133	Lactobacillus shenzhenensis
1134	Lactobacillus suebicus
1135	Lactococcus lactis
1136	Lactococcus raffinolactis
1137	Lamprocystis purpurea
1138	Laribacter hongkongensis
1139	Latimeria chalumnae
1140	Lautropia mirabilis
1141	Lawsonia intracellularis
1142	Leadbetterella byssophila
1143	Lechevalieria aerocolonigenes
1144	Leeia oryzae
1145	Leeuwenhoekiella blandensis
1146	Leeuwenhoekiella sp.
1147	Legionella drancourtii
1148	Legionella wadsworthii
1149	Leifsonia aquatica
1150	Leisingera aquimarina
1151	Leisingera nanhaiensis
1152	Leminorella grimontii
1153	Lentzea albidocapillata
1154	Lepisosteus oculatus

1155	Leptonychotes weddellii
1156	Leptosphaeria maculans
1157	Leptospira interrogans
1158	Leptotrichia goodfellowii
1159	Leptotrichia sp.
1160	Leucobacter chromiiresistens
1161	Leucobacter salsicius
1162	Leucobacter sp.
1163	Leuconostoc carnosum
1164	Leuconostoc mesenteroides
1165	Leucothrix mucor
1166	Lewinella cohaerens
1167	Lewinella persica
1168	Limnohabitans sp.
1169	Lipotes vexillifer
1170	Listeria grayi
1171	Listeria innocua
1172	Listeria monocytogenes
1173	Listeria seeligeri
1174	Listeria welshimeri
1175	Loa loa
1176	Loktanella hongkongensis
1177	Longispora albida
1178	Lottia gigantea
1179	Loxodonta africana
1180	Luteimonas huabeiensis
1181	Luteimonas mephitis
1182	Lutispora thermophila
1183	Lyngbya majuscula
1184	Lysinibacillus boronitolerans
1185	Lysinibacillus sphaericus
1186	Lysobacter antibioticus
1187	Macaca mulatta
1188	Magnetococcus marinus
1189	Magnetospirillum gryphiswaldense
1190	Magnetospirillum magneticum
1191	Mahella australiensis
1192	Malus x
1193	Manacus vitellinus
1194	Manihot esculenta
1195	Mannheimia haemolytica
1196	marine actinobacterium
1197	marine gamma
1198	Marinilabilia salmonicolor
1199	Marinimicrobia bacterium
1200	Marinimicrobium agarilyticum
1201	Marinimicrobium sp.
1202	Marinitoga piezophila
1203	Marinobacter adhaerens
1204	Marinobacter daepoensis
1205	Marinobacter lipolyticus
1206	Marinobacter sp.
1207	Marinobacterium rhizophilum
1208	Marinobacterium stanieri
1209	Marinomonas sp.
1210	Mariprofundus ferrooxydans
1211	Maritimibacter alkaliphilus
1212	Marivirga tractuosa

1213	Marmoricola sp.
1214	Martelella mediterranea
1215	Martelella sp.
1216	Massilia alkalitolerans
1217	Massilia niastensis
1218	Massilia timonae
1219	Mastigocoleus testarum
1220	Maylandia zebra
1221	Megamonas funiformis
1222	Megamonas hypermegale
1223	Megamonas rupellensis
1224	Megasphaera elsdenii
1225	Meiothermus cerbereus
1226	Meiothermus chliarophilus
1227	Meiothermus ruber
1228	Meiothermus rufus
1229	Meiothermus taiwanensis
1230	Meiothermus timidus
1231	Meleagris gallopavo
1232	Melitea salexigens
1233	Melopsittacus undulatus
1234	Meniscus glaucopis
1235	Merops nubicus
1236	Mesocricetus auratus
1237	Mesoflavibacter zeaxanthinifaciens
1238	Mesonia mobilis
1239	Mesoplasma syrphidae
1240	Mesorhizobium australicum
1241	Mesorhizobium ciceri
1242	Mesorhizobium loti
1243	Mesorhizobium sp.
1244	Metascardovia criceti
1245	Metaseiulus occidentalis
1246	Methanobrevibacter ruminantium
1247	Methanobrevibacter smithii
1248	Methanococcus voltae
1249	Methanoculleus marisnigri
1250	Methanomassiliicoccus luminyensis
1251	Methanomassiliicoccus sp.
1252	Methanosarcina acetivorans
1253	Methanosphaera stadtmanae
1254	Methanothermus fervidus
1255	Methylacidiphilum infernorum
1256	Methylibium petroleiphilum
1257	Methylobacillus flagellatus
1258	Methylobacillus glycogenes
1259	Methylobacter luteus
1260	Methylobacter tundripaludum
1261	Methylobacterium extorquens
1262	Methylobacterium nodulans
1263	Methylobacterium radiotolerans
1264	Methylobacterium sp.
1265	Methylocaldum szegediense
1266	Methylocapsa acidiphila
1267	Methylocella silvestris
1268	Methylocystis parvus
1269	Methylocystis sp.
1270	Methylohalobius crimeensis

1271	Methylomarinum vadi
1272	Methylomicrobium agile
1273	Methylomicrobium buryatense
1274	Methylomonas methanica
1275	Methylophaga sp.
1276	Methylophilaceae bacterium
1277	Methylophilus methylotrophus
1278	Methylophilus sp.
1279	Methylopila sp.
1280	Methylosarcina fibrata
1281	Methylosarcina lacus
1282	Methylosinus sp.
1283	Methylotenera versatilis
1284	Methyloversatilis sp.
1285	Methyloversatilis universalis
1286	Methylovorus glucosetrophus
1287	Methylovorus sp.
1288	Methylovulum miyakonense
1289	Micavibrio aeruginosavorus
1290	Microbacterium gubbeenense
1291	Microbacterium indicum
1292	Microbacterium luticocti
1293	Microbacterium maritypicum
1294	Microbacterium paraoxydans
1295	Microbacterium sp.
1296	Microbacterium testaceum
1297	Microbacterium yannicii
1298	Microbulbifer agarilyticus
1299	Microbulbifer variabilis
1300	Micrococcus luteus
1301	Microcystis aeruginosa
1302	Micromonas sp.
1303	Micromonospora globosa
1304	Micromonospora parva
1305	Micromonospora purpureochromogenes
1306	Microplitis demolitor
1307	Microtetraspora glauca
1308	Microtus ochrogaster
1309	Microvirga sp.
1310	Microvirgula aerodenitrificans
1311	Mitsuokella multacida
1312	Mitsuokella sp.
1313	Mobiluncus curtisii
1314	Modestobacter marinus,
1315	Monodelphis domestica
1316	Moorella thermoacetica
1317	Moraxella boevrei
1318	Moraxella catarrhalis
1319	Morganella morganii
1320	Mucilaginibacter paludis
1321	Mucispirillum schaedleri
1322	Muricauda ruestringensis
1323	Mus musculus
1324	Musca domestica
1325	Mustela putorius
1326	Myceliophthora thermophila
1327	Mycobacterium abscessus
1328	Mycobacterium avium
1329	Mycobacterium canettii
1330	Mycobacterium chubuense
1331	Mycobacterium gilvum
1332	Mycobacterium hassiacum
1333	Mycobacterium iranicum
1334	Mycobacterium neoaurum
1335	Mycobacterium parascrofulaceum
1336	Mycobacterium smegmatis
1337	Mycobacterium sp.
1338	Mycobacterium ulcerans
1339	Mycoplasma bovis
1340	Mycoplasma capricolum
1341	Mycoplasma cynos
1342	Mycoplasma gallisepticum
1343	Mycoplasma glycophilum
1344	Mycoplasma hominis
1345	Mycoplasma moatsii
1346	Mycoplasma mycoides
1347	Mycoplasma penetrans
1348	Mycoplasma salivarium
1349	Mycoplasma simbae
1350	Mycoplasma sp.
1351	Mycoplasma synoviae
1352	Myotis brandtii
1353	Myotis davidii
1354	Myotis lucifugus
1355	Myroides injenensis
1356	Myroides odoratimimus
1357	Myxococcus fulvus
1358	Myxococcus stipitatus
1359	Myxococcus xanthus
1360	Nafulsella turpanensis
1361	Nakamurella lactea
1362	Nakamurella multipartita
1363	Nannospalax galili
1364	Nasonia vitripennis
1365	Natronobacterium gregoryi
1366	Natronomonas pharaonis
1367	Nautilia profundicola
1368	Nectria haematococca
1369	Neisseria bacilliformis
1370	Neisseria gonorrhoeae
1371	Neisseria lactamica
1372	Neisseria meningitidis
1373	Neisseria shayeganii
1374	Neisseria sicca
1375	Neisseria sp.
1376	Neisseria wadsworthii
1377	Nematostella vectensis
1378	Neofusicoccum parvum
1379	Neosartorya fischeri
1380	Nesterenkonia alba
1381	Neurospora crassa
1382	Nevskia ramosa
1383	Nevskia soli
1384	Niastella koreensis
1385	Nicotiana tabacum
1386	Nisaea denitrificans

1387	Nitratireductor aquibiodomus
1388	Nitrobacter hamburgensis
1389	Nitrobacter sp.
1390	Nitrobacter winogradskyi
1391	Nitrococcus mobilis
1392	Nitrosococcus oceani
1393	Nitrosococcus watsonii
1394	Nitrosomonas eutropha
1395	Nitrosomonas sp.
1396	Nitrosospira briensis
1397	Nitrospina gracilis
1398	Nitrospirillum amazonense
1399	Nocardia brasiliensis
1400	Nocardia farcinica
1401	Nocardia otitidiscaviarum
1402	Nocardia rhamnosiphila
1403	Nocardia sp.
1404	Nocardioidaceae bacterium
1405	Nocardioides halotolerans
1406	Nocardioides koreensis
1407	Nocardioides sp.
1408	Nocardiopsis alba
1409	Nocardiopsis dassonvillei
1410	Nocardiopsis gilva
1411	Nocardiopsis halotolerans
1412	Nocardiopsis kunsanensis
1413	Nocardiopsis potens
1414	Nocardiopsis prasina
1415	Nocardiopsis valliformis
1416	Nocardiopsis xinjiangensis
1417	Nodosilinea nodulosa
1418	Nomascus leucogenys
1419	Nonlabens tegetincola
1420	Nostoc sp.
1421	Novispirillum itersonii
1422	Novosphingobium acidiphilum
1423	Novosphingobium lindaniclasticum
1424	Oceanicaulis alexandrii
1425	Oceanicola granulosus
1426	Oceanicola nanhaiensis
1427	Oceanicola sp.
1428	Oceanimonas smirnovii
1429	Oceanimonas sp.
1430	Oceanithermus profundus
1431	Oceanobacter kriegii
1432	Oceanospirillum beijerinckii
1433	Oceanospirillum sp.
1434	Ochotona princeps
1435	Ochrobactrum rhizosphaerae
1436	Ochrobactrum sp.
1437	Octadecabacter antarcticus
1438	Octadecabacter arcticus
1439	Octodon degus
1440	Odobenus rosmarus
1441	Odoribacter laneus
1442	Odoribacter sp.
1443	Oenococcus kitaharae
1444	Oerskovia turbata

1445	Oligella ureolytica
1446	Oligotropha carboxidovorans
1447	Olleya sp.
1448	Olsenella sp.
1449	Olsenella uli
1450	Onion yellows
1451	Opisthorchis viverrini
1452	Opitutaceae bacterium
1453	Opitutus terrae
1454	Orcinus orca
1455	Oreochromis niloticus
1456	Oribacterium sinus
1457	Oribacterium sp.
1458	Ornithorhynchus anatinus
1459	Orycteropus afer
1460	Oryctolagus cuniculus
1461	Oryza brachyantha
1462	Oryzias latipes
1463	Oscillatoria sp.
1464	Oscillibacter ruminantium
1465	Oscillibacter sp.
1466	Oscillibacter valericigenes
1467	Oscillochloris trichoides
1468	Oscillospiraceae bacterium
1469	Ostreococcus lucimarinus
1470	Ostreococcus tauri
1471	Otolemur garnettii
1472	Ottowia thiooxydans
1473	Ovis aries
1474	Owenweeksia hongkongensis
1475	Oxalobacter formigenes
1476	Oxalobacteraceae bacterium
1477	Paenibacillus alginolyticus
1478	Paenibacillus azotofixans
1479	Paenibacillus barcinonensis
1480	Paenibacillus ehimensis
1481	Paenibacillus fonticola
1482	Paenibacillus forsythiae
1483	Paenibacillus ginsengihumi
1484	Paenibacillus graminis
1485	Paenibacillus larvae
1486	Paenibacillus massiliensis
1487	Paenibacillus mucilaginosus
1488	Paenibacillus panacisoli
1489	Paenibacillus pasadenensis
1490	Paenibacillus pinihumi
1491	Paenibacillus polymyxa
1492	Paenibacillus sp.
1493	Paenibacillus taiwanensis
1494	Paenibacillus terrae
1495	Paenibacillus terrigena
1496	Paenisporosarcina sp.
1497	Paludibacter propionicigenes
1498	Paludibacterium yongneupense
1499	Pandoraea pnomenusa
1500	Pandoraea sp.
1501	Pannonibacter phragmitetus
1502	Panthera tigris

1503	Pantholops hodgsonii
1504	Pantoea ananatis
1505	Pantoea dispersa
1506	Pantoea sp.
1507	Papio anubis
1508	Paracoccus aminophilus
1509	Paracoccus denitrificans
1510	Paracoccus pantotrophus
1511	Paracoccus sp.
1512	Paracoccus zeaxanthinifaciens
1513	Paramecium tetraurelia
1514	Paraprevotella clara
1515	Paraprevotella xylaniphila
1516	Parascardovia denticolens
1517	Parasutterella excrementihominis
1518	Parasutterella secunda
1519	Parcubacteria bacterium
1520	Parvibaculum lavamentivorans
1521	Parvimonas micra
1522	Parvularcula bermudensis
1523	Parvularcula oceani
1524	Pasteurella dagmatis
1525	Pectobacterium atrosepticum
1526	Pectobacterium carotovorum
1527	Pectobacterium sp.
1528	Pediococcus claussenii
1529	Pediococcus pentosaceus
1530	Pedobacter agri
1531	Pedobacter arcticus
1532	Pedobacter borealis
1533	Pelagibaca bermudensis
1534	Pelagibacterium halotolerans
1535	Pelobacter carbinolicus
1536	Pelobacter propionicus
1537	Pelobacter seleniigenes
1538	Pelodictyon phaeoclathratiforme
1539	Pelodiscus sinensis
1540	Pelomonas saccharophila
1541	Pelomonas sp.
1542	Peptoclostridium difficile
1543	Peptoniphilus duerdenii
1544	Peptoniphilus indolicus
1545	Peptoniphilus rhinitidis
1546	Peptoniphilus sp.
1547	Peptostreptococcaceae bacterium
1548	Peptostreptococcus anaerobius
1549	Perkinsus marinus
1550	Peromyscus maniculatus
1551	Phaeobacter arcticus
1552	Phaeobacter caeruleus
1553	Phaeobacter daeponensis
1554	Phaeobacter inhibens
1555	Phanerochaete carnosa
1556	Phaseolus vulgaris
1557	Phoenix dactylifera
1558	Photobacterium halotolerans
1559	Photobacterium profundum
1560	Photorhabdus luminescens

1561	Physcomitrella patens
1562	Phytophthora infestans
1563	Phytophthora parasitica
1564	Picea abies
1565	Pilimelia anulata
1566	Pirellula staleyi
1567	Piscirickettsia salmonis
1568	Planctomyces brasiliensis
1569	Planctomyces limnophilus
1570	Plasmodium cynomolgi
1571	Plasmodium falciparum
1572	Pleomorphomonas koreensis
1573	Pleomorphomonas oryzae
1574	Plesiomonas shigelloides
1575	Pleurocapsa sp.
1576	Podospora anserina
1577	Poecilia formosa
1578	Poecilia reticulata
1579	Polaribacter irgensii
1580	Polaromonas glacialis
1581	Polaromonas naphthalenivorans
1582	Polaromonas sp.
1583	Polycyclovorans algicola
1584	Polymorphum gilvum
1585	Pongo abelii
1586	Pontibacter actiniarum
1587	Pontibacter roseus
1588	Populus fremontii
1589	Populus trichocarpa
1590	Poribacteria bacterium
1591	Porphyrobacter cryptus
1592	Porphyrobacter sp.
1593	Porphyromonas bennonis
1594	Porphyromonas cangingivalis
1595	Porphyromonas catoniae
1596	Porphyromonas gingivalis
1597	Porphyromonas gingivicanis
1598	Porphyromonas gulae
1599	Porphyromonas levii
1600	Porphyromonas macacae
1601	Porphyromonas somerae
1602	Porphyromonas sp.
1603	Porphyromonas uenonis
1604	Postia placenta
1605	Prauserella rugosa
1606	Prevotella albensis
1607	Prevotella amnii
1608	Prevotella aurantiaca
1609	Prevotella baroniae
1610	Prevotella bergensis
1611	Prevotella bivia
1612	Prevotella brevis
1613	Prevotella bryantii
1614	Prevotella copri
1615	Prevotella corporis
1616	Prevotella dentalis
1617	Prevotella dentasini
1618	Prevotella denticola

1619	Prevotella disiens
1620	Prevotella enoeca
1621	Prevotella falsenii
1622	Prevotella fusca
1623	Prevotella histicola
1624	Prevotella intermedia
1625	Prevotella loescheii
1626	Prevotella maculosa
1627	Prevotella marshii
1628	Prevotella melaninogenica
1629	Prevotella micans
1630	Prevotella multiformis
1631	Prevotella multisaccharivorax
1632	Prevotella nanceiensis
1633	Prevotella nigrescens
1634	Prevotella oralis
1635	Prevotella oris
1636	Prevotella oulorum
1637	Prevotella pallens
1638	Prevotella paludivivens
1639	Prevotella pleuritidis
1640	Prevotella ruminicola
1641	Prevotella saccharolytica
1642	Prevotella salivae
1643	Prevotella scopos
1644	Prevotella shahii
1645	Prevotella sp.
1646	Prevotella stercorea
1647	Prevotella tannerae
1648	Prevotella timonensis
1649	Prevotella veroralis
1650	Prochlorococcus sp.
1651	Prochlorothrix hollandica
1652	Prolixibacter bellariivorans
1653	Promicromonospora sukumoe
1654	Propionibacterium acidifaciens
1655	Propionibacterium freudenreichii
1656	Propionibacterium jensenii
1657	Propionibacterium sp.
1658	Propionibacterium thoenii
1659	Propionicicella superfundia
1660	Propionimicrobium lymphophilum
1661	Proteiniphilum acetatigenes
1662	Proteobacteria bacterium
1663	Proteocatella sphenisci
1664	Providencia stuartii
1665	Prunus mume
1666	Prunus persica
1667	Pseudaminobacter salicylatoxidans
1668	Pseudanabaena sp.
1669	Pseudoalteromonas haloplanktis
1670	Pseudoalteromonas ruthenica
1671	Pseudobutyrivibrio ruminis
1672	Pseudobutyrivibrio sp.
1673	Pseudochrobactrum sp.
1674	Pseudoduganella violaceinigra
1675	Pseudogulbenkiania ferrooxidans
1676	Pseudogulbenkiania sp.

1677	Pseudomonas aeruginosa
1678	Pseudomonas agarici
1679	Pseudomonas alcaligenes
1680	Pseudomonas alcaliphila
1681	Pseudomonas azotifigens
1682	Pseudomonas brassicacearum
1683	Pseudomonas caeni
1684	Pseudomonas chloritidismutans
1685	Pseudomonas chlororaphis
1686	Pseudomonas cremoricolorata
1687	Pseudomonas denitrificans
1688	Pseudomonas entomophila
1689	Pseudomonas fluorescens
1690	Pseudomonas fragi
1691	Pseudomonas fuscovaginae
1692	Pseudomonas mendocina
1693	Pseudomonas monteilii
1694	Pseudomonas moraviensis
1695	Pseudomonas mosselii
1696	Pseudomonas nitroreducens
1697	Pseudomonas oleovorans
1698	Pseudomonas parafulva
1699	Pseudomonas plecoglossicida
1700	Pseudomonas poae
1701	Pseudomonas putida
1702	Pseudomonas resinovorans
1703	Pseudomonas sp.
1704	Pseudomonas stutzeri
1705	Pseudomonas syringae
1706	Pseudomonas thermotolerans
1707	Pseudomonas tolaasii
1708	Pseudomonas umsongensis
1709	Pseudomonas viridiflava
1710	Pseudonocardia acaciae
1711	Pseudonocardia asaccharolytica
1712	Pseudonocardia dioxanivorans
1713	Pseudopodoces humilis
1714	Pseudoramibacter alactolyticus
1715	Pseudorhodobacter ferrugineus
1716	Pseudovibrio sp.
1717	Pseudoxanthomonas sp.
1718	Pseudoxanthomonas spadix
1719	Pseudoxanthomonas suwonensis
1720	Psychrilyobacter atlanticus
1721	Psychrobacter lutiphocae
1722	Psychromonas sp.
1723	Psychroserpens burtonensis
1724	Pteropus alecto
1725	Pusillimonas noertemannii
1726	Pusillimonas sp.
1727	Python bivittatus
1728	Rahnella aquatilis
1729	Ralstonia eutropha
1730	Ralstonia pickettii
1731	Ralstonia solanacearum
1732	Ralstonia sp.
1733	Ramlibacter tataouinensis
1734	Raoultella ornithinolytica

1735	Rathayibacter toxicus
1736	Rattus norvegicus
1737	Rhazya stricta
1738	Rheinheimera texasensis
1739	Rhizobium etli
1740	Rhizobium gallicum
1741	Rhizobium giardinii
1742	Rhizobium leguminosarum
1743	Rhizobium leucaenae
1744	Rhizobium phaseoli
1745	Rhizobium selenitireducens
1746	Rhizobium sp.
1747	Rhizobium sullae
1748	Rhizobium tropici
1749	Rhizobium undicola
1750	Rhodanobacter sp.
1751	Rhodobacter capsulatus
1752	Rhodobacter sphaeroides
1753	Rhodobacteraceae bacterium
1754	Rhodobacterales bacterium
1755	Rhodococcus defluvii
1756	Rhodococcus equi
1757	Rhodococcus erythropolis
1758	Rhodococcus jostii
1759	Rhodococcus opacus
1760	Rhodococcus pyridinivorans
1761	Rhodococcus ruber
1762	Rhodococcus sp.
1763	Rhodoferax ferrireducens
1764	Rhodoferax saidenbachensis
1765	Rhodomicrobium vannielii
1766	Rhodonellum psychrophilum
1767	Rhodopirellula baltica
1768	Rhodopseudomonas palustris
1769	Rhodopseudomonas sp.
1770	Rhodospirillales bacterium
1771	Rhodospirillum centenum
1772	Rhodospirillum photometricum
1773	Rhodospirillum rubrum
1774	Rhodothermus marinus
1775	Rhodovibrio salinarum
1776	Ricinus communis
1777	Rickettsia canadensis
1778	Rickettsia felis
1779	Rickettsia montanensis
1780	Rickettsia prowazekii
1781	Rickettsia rickettsii
1782	Rikenella microfusus
1783	Robiginitalea biformata
1784	Robiginitomaculum antarcticum
1785	Robinsoniella sp.
1786	Roseburia hominis
1787	Roseburia sp.
1788	Roseibium sp.
1789	Roseiflexus castenholzii
1790	Roseobacter denitrificans
1791	Roseobacter sp.
1792	Roseomonas cervicalis

1793	Roseomonas sp.
1794	Ruania albidiflava
1795	Rubritalea marina
1796	Rubritepida flocculans
1797	Rubrivivax benzoatilyticus
1798	Rubrivivax gelatinosus
1799	Rubrobacter xylanophilus
1800	Rudaea cellulosilytica
1801	Rudanella lutea
1802	Ruegeria pomeroyi
1803	Ruegeria sp.
1804	Ruminococcaceae bacterium
1805	Ruminococcus albus
1806	Ruminococcus callidus
1807	Ruminococcus champanellensis
1808	Ruminococcus flavefaciens
1809	Ruminococcus gauvreauii
1810	Ruminococcus lactaris
1811	Ruminococcus sp.
1812	Runella limosa
1813	Runella slithyformis
1814	Runella zeae
1815	Saccharibacillus kuerlensis
1816	Saccharibacter floricola
1817	Saccharomonospora azurea
1818	Saccharomonospora marina
1819	Saccharomonospora paurometabolica
1820	Saccharomonospora saliphila
1821	Saccharomonospora sp.
1822	Saccharomonospora viridis
1823	Saccharomonospora xinjiangensis
1824	Saccharomyces cerevisiae
1825	Saccharopolyspora rectivirgula
1826	Saccharopolyspora spinosa
1827	Saccharospirillum impatiens
1828	Saccoglossus kowalevskii
1829	Saimiri boliviensis
1830	Salana multivorans
1831	Salinarimonas rosea
1832	Salinibacter ruber
1833	Salinicoccus albus
1834	Salinispora arenicola
1835	Salinispora pacifica
1836	Salisaeta longa
1837	Salmonella enterica
1838	Sandarakinorhabdus sp.
1839	Saprospira grandis
1840	SAR324 cluster
1841	Sarcophilus harrisii
1842	Schlesneria paludicola
1843	Sciscionella sp.
1844	Sedimenticola selenatireducens
1845	Sediminibacillus halophilus
1846	Sediminibacter sp.
1847	Sediminibacterium salmoneum
1848	Sediminibacterium sp.
1849	Segetibacter koreensis
1850	Segniliparus rotundus

1851	Selenomonas artemidis
1852	Selenomonas bovis
1853	Selenomonas infelix
1854	Selenomonas noxia
1855	Selenomonas ruminantium
1856	Selenomonas sp.
1857	Selenomonas sputigena
1858	Serinicoccus marinus
1859	Serinicoccus profundi
1860	Serpula lacrymans
1861	Serratia fonticola
1862	Serratia liquefaciens
1863	Serratia marcescens
1864	Serratia plymuthica
1865	Serratia proteamaculans
1866	Serratia sp.
1867	Setaria italica
1868	Shewanella amazonensis
1869	Shewanella baltica
1870	Shewanella halifaxensis
1871	Shewanella haliotis
1872	Shewanella loihica
1873	Shewanella pealeana
1874	Shewanella sp.
1875	Shewanella violacea
1876	Shinella sp.
1877	Shuttleworthia satelles
1878	Silanimonas lenta
1879	Silene conica
1880	Silicibacter lacuscaerulensis
1881	Silicibacter sp.
1882	Simiduia agarivorans
1883	Simonsiella muelleri
1884	Simplicispira psychrophila
1885	Singularimonas variicoloris
1886	Singulisphaera acidiphila
1887	Sinorhizobium fredii
1888	Sinorhizobium meliloti
1889	Slackia exigua
1890	Slackia heliotrinireducens
1891	Slackia piriformis
1892	Sodalis glossinidius
1893	Solanum tuberosum
1894	Solibacillus silvestris
1895	Solimonas flava
1896	Solimonas soli
1897	Solirubrobacter soli
1898	Solirubrobacter sp.
1899	Solirubrobacterales bacterium
1900	Solitalea canadensis
1901	Solobacterium moorei
1902	Sorangium cellulosum
1903	Sorex araneus
1904	Sorghum bicolor
1905	Spermophilus tridecemlineatus
1906	Sphaerobacter thermophilus
1907	Sphingobacterium sp.
1908	Sphingobacterium spiritivorum
1909	Sphingobacterium thalpophilum
1910	Sphingobium baderi
1911	Sphingobium lactosutens
1912	Sphingobium quisquiliarum
1913	Sphingobium sp.
1914	Sphingobium ummariense
1915	Sphingobium xenophagum
1916	Sphingobium yanoikuyae
1917	Sphingomonas astaxanthinifaciens
1918	Sphingomonas elodea
1919	Sphingomonas melonis
1920	Sphingomonas sp.
1921	Sphingomonas wittichii
1922	Sphingomonas-like bacterium
1923	Sphingopyxis alaskensis
1924	Spiribacter salinus
1925	Spirillospora albida
1926	Spirochaeta africana
1927	Spirochaeta alkalica
1928	Spirochaeta bajacaliforniensis
1929	Spirochaeta caldaria
1930	Spirochaeta sp.
1931	Spirochaeta thermophila
1932	Spiroplasma chrysopicola
1933	Spiroplasma taiwanense
1934	Spirosoma linguale
1935	Spirosoma luteum
1936	Spirosoma panaciterrae
1937	Spirosoma spitsbergense
1938	Spongiibacter tropicus
1939	Sporobacter termitidis
1940	Sporocytophaga myxococcoides
1941	Sporolactobacillus terrae
1942	Sporolactobacillus vineae
1943	Sporosarcina newyorkensis
1944	Sporosarcina sp.
1945	Stackebrandtia nassauensis
1946	Staphylococcus aureus
1947	Staphylococcus hominis
1948	Staphylococcus pasteuri
1949	Staphylococcus saprophyticus
1950	Staphylococcus simulans
1951	Staphylococcus sp.
1952	Staphylococcus warneri
1953	Staphylococcus xylosus
1954	Stappia stellulata
1955	Stegastes partitus
1956	Stenotrophomonas maltophilia
1957	Stenotrophomonas sp.
1958	Stereum hirsutum
1959	Stigmatella aurantiaca
1960	Streptobacillus moniliformis
1961	Streptococcus agalactiae
1962	Streptococcus anginosus
1963	Streptococcus caballi
1964	Streptococcus constellatus
1965	Streptococcus dysgalactiae
1966	Streptococcus entericus

1967	Streptococcus equinus
1968	Streptococcus ferus
1969	Streptococcus gordonii
1970	Streptococcus infantis
1971	Streptococcus intermedius
1972	Streptococcus mitis
1973	Streptococcus oralis
1974	Streptococcus orisratti
1975	Streptococcus pasteurianus
1976	Streptococcus pleomorphus
1977	Streptococcus pneumoniae
1978	Streptococcus pseudopneumoniae
1979	Streptococcus pyogenes
1980	Streptococcus sanguinis
1981	Streptococcus sobrinus
1982	Streptococcus sp.
1983	Streptococcus suis
1984	Streptococcus thermophilus
1985	Streptococcus uberis
1986	Streptococcus vestibularis
1987	Streptomyces achromogenes
1988	Streptomyces acidiscabies
1989	Streptomyces albus
1990	Streptomyces bicolor
1991	Streptomyces chartreusis
1992	Streptomyces davawensis
1993	Streptomyces erythrochromogenes
1994	Streptomyces exfoliatus
1995	Streptomyces flavochromogenes
1996	Streptomyces globisporus
1997	Streptomyces griseoflavus
1998	Streptomyces griseus
1999	Streptomyces hygroscopicus
2000	Streptomyces lavendulae
2001	Streptomyces lavenduligriseus
2002	Streptomyces leeuwenhoekii
2003	Streptomyces mediolani
2004	Streptomyces natalensis
2005	Streptomyces olivaceus
2006	Streptomyces peucetius
2007	Streptomyces pristinaespiralis
2008	Streptomyces prunicolor
2009	Streptomyces pyridomyceticus
2010	Streptomyces roseochromogenus
2011	Streptomyces ruber
2012	Streptomyces sclerotialus
2013	Streptomyces scopuliridis
2014	Streptomyces somaliensis
2015	Streptomyces sp.
2016	Streptomyces sulphureus
2017	Streptomyces sviceus
2018	Streptomyces varsoviensis
2019	Streptomyces violaceoruber
2020	Streptomyces violaceusniger
2021	Streptomyces viridochromogenes
2022	Streptomyces viridosporus
2023	Streptomyces xanthophaeus
2024	Streptosporangium amethystogenes

2025	Streptosporangium roseum
2026	Strongylocentrotus purpuratus
2027	Subdoligranulum sp.
2028	Succinatimonas hippei
2029	Succinatimonas sp.
2030	Succinimonas amylolytica
2031	Sulfitobacter sp.
2032	Sulfobacillus acidophilus
2033	Sulfobacillus thermosulfidooxidans
2034	Sulfolobus islandicus
2035	Sulfuricella denitrificans
2036	Sulfurihydrogenibium sp.
2037	Sulfurimonas sp.
2038	Sulfurovum sp.
2039	Sus scrofa
2040	Sutterella parvirubra
2041	Sutterella sp.
2042	Sutterella wadsworthensis
2043	Symbiobacterium thermophilum
2044	Synechococcus elongatus
2045	Synechococcus phage
2046	Synechococcus sp.
2047	Synergistes sp.
2048	Synergistetes bacterium
2049	Syntrophobacter fumaroxidans
2050	Syntrophobotulus glycolicus
2051	Syntrophomonas wolfei
2052	Syntrophorhabdus aromaticivorans
2053	Taeniopygia guttata
2054	Takifugu rubripes
2055	Tannerella forsythia
2056	Tannerella sp.
2057	Tarsius syrichta
2058	Tenacibaculum maritimum
2059	Tenacibaculum ovolyticum
2060	Tenacibaculum sp.
2061	Tepidanaerobacter acetatoxydans
2062	Tepidiphilus margaritifer
2063	Terasakiella pusilla
2064	Teredinibacter turnerae
2065	Terriglobus roseus
2066	Terrimonas ferruginea
2067	Terrisporobacter glycolicus
2068	Tetragenococcus halophilus
2069	Tetragenococcus muriaticus
2070	Tetrahymena thermophila
2071	Thalassiosira pseudonana
2072	Thalassobacter arenae
2073	Thalassobaculum salexigens
2074	Thalassospira lucentensis
2075	Thauera linaloolentis
2076	Thauera sp.
2077	Thauera terpenica
2078	Theobroma cacao
2079	Thermacetogenium phaeum
2080	Thermaerobacter marianensis
2081	Thermaerobacter subterraneus
2082	Thermanaerovibrio acidaminovorans

2083	Thermanaerovibrio velox
2084	Thermincola potens
2085	Thermithiobacillus tepidarius
2086	Thermoanaerobacter indiensis
2087	Thermoanaerobacter siderophilus
2088	Thermoanaerobacterium thermosaccharolyticum
2089	Thermoanaerobacterium xylanolyticum
2090	Thermobacillus composti
2091	Thermobispora bispora
2092	Thermococcus gammatolerans
2093	Thermococcus sp.
2094	Thermocrinis albus
2095	Thermodesulfobacterium hveragerdense
2096	Thermofilum sp.
2097	Thermomonas fusca
2098	Thermomonospora curvata
2099	Thermophagus xiamenensis
2100	Thermoplasmatales archaeon
2101	Thermosediminibacter oceani
2102	Thermus igniterrae
2103	Thermus islandicus
2104	Thermus oshimai
2105	Thermus thermophilus
2106	Thielavia terrestris
2107	Thioalkalivibrio sp.
2108	Thioalkalivibrio sulfidophilus
2109	Thioalkalivibrio thiocyanodenitrificans
2110	Thioalkalivibrio thiocyanoxidans
2111	Thiobacillus denitrificans
2112	Thiobacillus thioparus
2113	Thiocystis violascens
2114	Thiomicrospira chilensis
2115	Thiomicrospira halophila
2116	Thiomicrospira pelophila
2117	Thiomicrospira sp.
2118	Thiomonas intermedia
2119	Thiomonas sp.
2120	Thiothrix lacustris
2121	Thiothrix nivea
2122	Tistrella mobilis
2123	Tolumonas auensis
2124	Tomitella biformata
2125	Treponema azotonutricium
2126	Treponema brennaborense
2127	Treponema bryantii
2128	Treponema denticola
2129	Treponema lecithinolyticum
2130	Treponema maltophilum
2131	Treponema medium
2132	Treponema pedis
2133	Treponema phagedenis
2134	Treponema primitia
2135	Treponema socranskii
2136	Treponema sp.
2137	Treponema succinifaciens
2138	Treponema vincentii
2139	Tribolium castaneum

2140	Trichechus manatus
2141	Trichomonas vaginalis
2142	Triticum timopheevii
2143	Triticum urartu
2144	Tsukamurella paurometabola
2145	Tuber melanosporum
2146	Tuberibacillus calidus
2147	Tupaia chinensis
2148	Turicibacter sanguinis
2149	Turneriella parva
2150	Tyzzerella nexilis
2151	Uliginosibacterium gangwonense
2152	Uncultured bacterium
2153	Ursus maritimus
2154	Variovorax paradoxus
2155	Variovorax sp.
2156	Verminephrobacter aporrectodeae
2157	Verminephrobacter eiseniae
2158	Verrucomicrobia bacterium
2159	Verrucomicrobiae bacterium
2160	Verrucomicrobium spinosum
2161	Vibrio cholerae
2162	Vibrio cyclitrophicus
2163	Vibrio furnissii
2164	Vibrio litoralis
2165	Vibrio natriegens
2166	Vibrio parahaemolyticus
2167	Vibrio phage
2168	Vibrio rumoiensis
2169	Vibrio sp.
2170	Vibrio splendidus
2171	Vibrio tapetis
2172	Vicugna pacos
2173	Virgibacillus alimentarius
2174	Virgibacillus sp.
2175	Vitis vinifera
2176	Vitreoscilla stercoraria
2177	Volvox carteri
2178	Weeksella virosa
2179	Weissella paramesenteroides
2180	Woodsholea maritima
2181	Xanthobacter autotrophicus
2182	Xanthobacter sp.
2183	Xanthobacteraceae bacterium
2184	Xanthomonas arboricola
2185	Xanthomonas axonopodis
2186	Xanthomonas campestris
2187	Xanthomonas cassavae
2188	Xanthomonas citri
2189	Xanthomonas sp.
2190	Xanthomonas translucens
2191	Xenopus (Silurana)
2192	Xenorhabdus bovienii
2193	Xiphophorus maculatus
2194	Xylanibacter oryzae
2195	Xylanimonas cellulosilytica
2196	Xylella fastidiosa
2197	Yokenella regensburgei

2198	Zea mays
2199	Zeta proteobacterium
2200	Zonotrichia albicollis
2201	Zymobacter palmae
2202	Zymophilus raffinosivorans

Table S30 : The taxonomic profile predicted from the assembly results of MetaVelvet-SL using BLAST for the SRS017227 dataset.

No	Species
1	Actinomyces odontolyticus
2	Actinomyces oris
3	Actinomyces urogenitalis
4	Actinomyces viscosus
5	Aggregatibacter actinomycetemcomitans
6	Aggregatibacter aphrophilus
7	Aggregatibacter segnis
8	Alistipes putredinis
9	Atopobium parvulum
10	Atopobium rimae
11	Bacteroides caccae
12	Bacteroides ovatus
13	Bacteroides unclassified
14	Bacteroides vulgatus
15	Campylobacter concisus
16	Campylobacter gracilis
17	Campylobacter showae
18	Capnocytophaga gingivalis
19	Capnocytophaga ochracea
20	Capnocytophaga sputigena
21	Cardiobacterium hominis
22	Catonella morbi
23	Corynebacterium matruchotii
24	Dialister invisus
25	Eikenella corrodens
26	Eubacterium saburreum
27	Fusobacterium nucleatum
28	Fusobacterium periodonticum
29	Gemella haemolysans
30	Gemella moribillum
31	Granulicatella adiacens
32	Granulicatella elegans
33	Haemophilus influenzae
34	Haemophilus parainfluenzae
35	Kingella denitrificans
36	Lautropia mirabilis
37	Leptotrichia buccalis
38	Leptotrichia goodfellowii
39	Leptotrichia hofstadii
40	Leptotrichia unclassified
41	Megasphaera micronuciformis
42	Neisseria elongata
43	Neisseria gonorrhoeae
44	Neisseria meningitidis
45	Neisseria mucosa
46	Neisseria polysaccharea
47	Neisseria sicca
48	Neisseria subflava
49	Oribacterium sinus
50	Parabacteroides merdae
51	Peptoniphilus unclassified
52	Porphyromonas endodontalis
53	Prevotella copri
54	Prevotella marshii
55	Prevotella oris
56	Prevotella salivae
57	Prevotella tannerae
58	Prevotella veroralis
59	Propionibacterium acnes
60	Rothia dentocariosa
61	Rothia mucilaginosa
62	Selenomonas artemidis
63	Selenomonas flueggei
64	Selenomonas noxia
65	Selenomonas sputigena
66	Slackia exigua
67	Solobacterium moorei
68	Streptococcus australis
69	Streptococcus cristatus
70	Streptococcus gordonii
71	Streptococcus infantis
72	Streptococcus mitis
73	Streptococcus oralis
74	Streptococcus parasanguinis
75	Streptococcus peroris
76	Streptococcus salivarius
77	Streptococcus sanguinis
78	Treponema denticola
79	Treponema vincentii
80	Veillonella atypica
81	Veillonella dispar
82	Veillonella parvula
83	Veillonella unclassified
84	Nostoc azollae'
85	[Clostridium] aerotolerans
86	[Clostridium] aminophilum
87	[Clostridium] bifermentans
88	[Clostridium] cellobioparum
89	[Clostridium] clostridioforme
90	[Clostridium] indolis
91	[Clostridium] josui
92	[Clostridium] mangenotii
93	[Clostridium] methoxybenzovorans
94	[Clostridium] papyrosolvens
95	[Clostridium] saccharogumia
96	[Clostridium] sordellii
97	[Clostridium] sticklandii,
98	[Clostridium] termitidis
99	[Clostridium] viride
100	[Eubacterium] cellulosolvens
101	[Eubacterium] yurii
102	[Pseudomonas] geniculata
103	Abiotrophia defectiva
104	Acanthamoeba castellanii
105	Acanthisitta chloris
106	Acaricomes phytoseiuli
107	Acaryochloris marina
108	Acaryochloris sp.
109	Acetivibrio cellulolyticus
110	Acetobacter aceti

111	Acetobacter pasteurianus
112	Acetobacter sp.
113	Acetobacterium dehalogenans
114	Acetobacterium woodii
115	Acholeplasma axanthum
116	Acholeplasma equifetale
117	Acholeplasma granularum
118	Acholeplasma laidlawii
119	Acholeplasma modicum
120	Acholeplasma sp.
121	Achromobacter piechaudii
122	Achromobacter xylosoxidans
123	Acidaminococcus sp.
124	Acidimicrobidae bacterium
125	Acidimicrobium ferrooxidans
126	Acidiphilium angustum
127	Acidiphilium multivorum
128	Acidiphilium sp.
129	Acidithiobacillus caldus
130	Acidithiobacillus ferrivorans
131	Acidithiobacillus ferrooxidans
132	Acidithiobacillus thiooxidans
133	Acidobacteria bacterium
134	Acidobacteriaceae bacterium
135	Acidobacterium capsulatum
136	Acidobacterium sp.
137	Acidocella sp.
138	Acidothermus cellulolyticus
139	Acidovorax avenae
140	Acidovorax citrulli
141	Acidovorax ebreus
142	Acidovorax oryzae
143	Acidovorax radicis
144	Acidovorax sp.
145	Acinetobacter baumannii
146	Acinetobacter bereziniae
147	Acinetobacter bouvetii
148	Acinetobacter brisouii
149	Acinetobacter calcoaceticus
150	Acinetobacter gyllenbergii
151	Acinetobacter haemolyticus
152	Acinetobacter johnsonii
153	Acinetobacter junii
154	Acinetobacter lwoffii
155	Acinetobacter nectaris
156	Acinetobacter nosocomialis
157	Acinetobacter oleivorans
158	Acinetobacter parvus
159	Acinetobacter pittii
160	Acinetobacter radioresistens
161	Acinetobacter schindleri
162	Acinetobacter sp.
163	Acinetobacter towneri
164	Acinetobacter ursingii
165	Acinetobacter venetianus
166	Actinoalloteichus cyanogriseus
167	Actinoalloteichus spitiensis
168	Actinobacillus capsulatus

169	Actinobacillus pleuropneumoniae
170	Actinobacillus succinogenes
171	Actinobacillus suis
172	Actinobacillus ureae
173	Actinobacterium LLX17
174	Actinobacterium SCGC
175	Actinobaculum massiliae
176	Actinobaculum schaalii
177	Actinobaculum sp.
178	Actinobaculum urinale
179	Actinocatenispora sera
180	Actinokineospora enzanensis
181	Actinokineospora inagensis
182	Actinomadura atramentaria
183	Actinomadura flavalba
184	Actinomadura madurae
185	Actinomadura oligospora
186	Actinomadura rifamycini
187	Actinomyces cardiffensis
188	Actinomyces dentalis
189	Actinomyces europaeus
190	Actinomyces georgiae
191	Actinomyces gerencseriae
192	Actinomyces graevenitzii
193	Actinomyces israelii
194	Actinomyces johnsonii
195	Actinomyces massiliensis
196	Actinomyces meyeri
197	Actinomyces naeslundii
198	Actinomyces neuii
199	Actinomyces ruminicola
200	Actinomyces slackii
201	Actinomyces sp.
202	Actinomyces suimastitidis
203	Actinomyces timonensis
204	Actinomyces turicensis
205	Actinomyces vaccimaxillae
206	Actinomyces weissii
207	Actinomycetospora chiangmaiensis
208	Actinoplanes friuliensis
209	Actinoplanes globisporus
210	Actinoplanes missouriensis
211	Actinoplanes sp.
212	Actinoplanes subtropicus
213	Actinopolymorpha alba
214	Actinopolyspora halophila
215	Actinopolyspora mortivallis
216	Actinosynnema mirum
217	Acyrthosiphon pisum
218	Adhaeribacter aquaticus
219	Adlercreutzia equolifaciens
220	Advenella kashmirensis
221	Aedes aegypti
222	Aequorivita capsosiphonis
223	Aequorivita sublithincola
224	Aerococcus urinae
225	Aeromicrobium marinum
226	Aeromonas caviae

227	Aeromonas hydrophila
228	Aeromonas salmonicida
229	Aeromonas veronii
230	Aestuariibacter salexigens
231	Aestuariimicrobium kwangyangense
232	Afifella pfennigii
233	Afipia felis
234	Afipia sp.
235	Aggregatibacter phage
236	Aggregatibacter sp.
237	Agrobacterium fabrum
238	Agrobacterium radiobacter
239	Agrobacterium tumefaciens
240	Agrobacterium vitis
241	Agrococcus lahaulensis
242	Agrococcus pavilionensis
243	Agromonas oligotrophica
244	Agromyces italicus
245	Agromyces subbeticus
246	Ahrensia kielensis
247	Ailuropoda melanoleuca
248	Ajellomyces dermatitidis
249	Akkermansia muciniphila
250	Akkermansia muciniphila,
251	Akkermansia sp.
252	Alcaligenes sp.
253	Alcanivorax borkumensis
254	Alcanivorax dieselolei
255	Alcanivorax sp.
256	Algoriphagus mannitolivorans
257	Algoriphagus marincola
258	Algoriphagus sp.
259	Algoriphagus terrigena
260	Algoriphagus vanfongensis
261	Aliagarivorans marinus
262	Aliagarivorans taiwanensis
263	Alicycliphilus denitrificans
264	Alicyclobacillus acidocaldarius
265	Alicyclobacillus contaminans
266	Alicyclobacillus herbarius
267	Alicyclobacillus macrosporangiidus
268	Alicyclobacillus pomorum
269	Aliivibrio fischeri
270	Aliivibrio logei
271	Alistipes finegoldii
272	Alistipes indistinctus
273	Alistipes onderdonkii
274	Alistipes senegalensis
275	Alistipes shahii
276	Alistipes sp.
277	Alistipes timonensis
278	Alkalibacillus haloalkaliphilus
279	Alkaliflexus imshenetskii
280	Alkalilimnicola ehrlichii
281	Alkaliphilus metalliredigens
282	Alkaliphilus transvaalensis
283	Alkanindiges illinoisensis
284	Alligator mississippiensis
285	Alligator sinensis
286	Allisonella histaminiformans
287	Allochromatium vinosum
288	Allofustis seminis
289	Allokutzneria albata
290	Alloprevotella rava
291	Alloscardovia omnicolens
292	Alpha proteobacterium
293	alpha proteobacterium
294	Alteromonas macleodii
295	Alteromonas sp.
296	Alysiella filiformis
297	Amborella trichopoda
298	Aminicenantes bacterium
299	Aminobacter sp.
300	Aminomonas paucivorans
301	Amorphus coralli
302	Amphibacillus jilinensis
303	Amphimedon queenslandica
304	Amphritea japonica
305	Amycolatopsis alba
306	Amycolatopsis balhimycina
307	Amycolatopsis benzoatilytica
308	Amycolatopsis mediterranei
309	Amycolatopsis methanolica
310	Amycolatopsis nigrescens
311	Amycolatopsis orientalis
312	Amycolatopsis sp.
313	Amycolatopsis taiwanensis
314	Amycolatopsis thermoflava
315	Amycolicicoccus subflavus
316	Anabaena cylindrica
317	Anabaena sp.
318	Anabaena variabilis
319	Anaeroarcus burkinensis
320	Anaerococcus lactolyticus
321	Anaerococcus obesiensis
322	Anaerococcus prevotii
323	Anaerococcus sp.
324	Anaerococcus tetradius
325	Anaerococcus vaginalis
326	Anaerofustis stercorihominis
327	Anaeroglobus geminatus
328	Anaeromusa acidaminophila
329	Anaeromyxobacter dehalogenans
330	Anaeromyxobacter sp.
331	Anaerophaga thermohalophila
332	Anaerostipes caccae
333	Anaerostipes hadrus
334	Anaerotruncus colihominis
335	Anaerotruncus sp.
336	Anaerovibrio lipolyticus
337	Anaerovibrio sp.
338	Anaerovorax odorimutans
339	Anas platyrhynchos
340	Andalucia godoyi
341	Aneurinibacillus aneurinilyticus
342	Anolis carolinensis

343	Anopheles gambiae
344	Anoxybacillus flavithermus
345	Anoxybacillus tepidamans
346	Aotus nancymaae
347	Aphanizomenon flos-aquae
348	Aphanomyces invadans
349	Apis dorsata
350	Apis florea
351	Apis mellifera
352	Aplysia californica
353	Aptenodytes forsteri
354	Aquaspirillum serpens
355	Aquimarina agarilytica
356	Aquimarina latercula
357	Aquimarina macrocephali
358	Aquimarina megaterium
359	Aquimarina muelleri
360	Aquimarina sp.
361	Arabidopsis lyrata
362	Arabidopsis thaliana
363	Arcanobacterium haemolyticum
364	Arcobacter butzleri
365	Arcobacter cibarius
366	Arcobacter nitrofigilis
367	Arcobacter sp.
368	Arenibacter algicola
369	Arenibacter certesii
370	Arenibacter latericius
371	Arenimonas composti
372	Arenimonas oryziterrae
373	Arenitalea lutea
374	Arhodomonas aquaeolei
375	Aromatoleum aromaticum
376	Arsenicicoccus bolidensis
377	Arsenophonus nasoniae
378	Arthrobacter arilaitensis
379	Arthrobacter aurescens
380	Arthrobacter castelli
381	Arthrobacter chlorophenolicus
382	Arthrobacter nicotinovorans
383	Arthrobacter phenanthrenivorans
384	Arthrobacter sanguinis
385	Arthrobacter sp.
386	Arthroderma benhamiae
387	Arthroderma gypseum
388	Asaia astilbes
389	Asaia platycodi
390	Asaia prunellae
391	Asaia sp.
392	Asterionellopsis glacialis
393	Asticcacaulis benevestitus
394	Asticcacaulis biprosthecum
395	Asticcacaulis excentricus
396	Asticcacaulis sp.
397	Astyanax mexicanus
398	Atopobacter phocae
399	Atopobium fossor
400	Atopobium minutum

401	Atopobium sp.
402	Atopobium vaginae
403	Atopococcus tabaci
404	Atribacteria bacterium
405	Aurantimonas coralicida
406	Aureococcus anophagefferens
407	Avibacterium paragallinarum
408	Azoarcus sp.
409	Azoarcus toluclasticus
410	Azohydromonas australica
411	Azonexus hydrophilus
412	Azorhizobium caulinodans
413	Azorhizobium doebereinerae
414	Azospirillum brasilense
415	Azospirillum halopraeferens
416	Azospirillum irakense
417	Azospirillum lipoferum
418	Azospirillum sp.
419	Azotobacter vinelandii
420	Azovibrio restrictus
421	Bacillus acidiproducens
422	Bacillus amyloliquefaciens
423	Bacillus anthracis
424	Bacillus atrophaeus
425	Bacillus aurantiacus
426	Bacillus bogoriensis
427	Bacillus cellulosilyticus
428	Bacillus cereus
429	Bacillus chagannorensis
430	Bacillus coagulans
431	Bacillus coahuilensis
432	Bacillus cytotoxicus
433	Bacillus endophyticus
434	Bacillus flexus
435	Bacillus fordii
436	Bacillus ginsengihumi
437	Bacillus halochares
438	Bacillus kribbensis
439	Bacillus licheniformis
440	Bacillus mannanilyticus
441	Bacillus massilioanorexius
442	Bacillus massiliosenegalensis
443	Bacillus megaterium
444	Bacillus mycoides
445	Bacillus panaciterrae
446	Bacillus phage
447	Bacillus pumilus
448	Bacillus selenitireducens
449	Bacillus siamensis
450	Bacillus smithii
451	Bacillus sp.
452	Bacillus subtilis
453	Bacillus thuringiensis
454	Bacillus timonensis
455	Bacillus toyonensis
456	Bacillus vallismortis
457	Bacteriophage Aaphi23,
458	Bacteriovorax marinus

459	Bacterium JKG1
460	Bacteroidales bacterium
461	Balaenoptera acutorostrata
462	Balneola vulgaris
463	Barnesiella intestinihominis
464	Bartonella alsatica
465	Bartonella australis
466	Bartonella bacilliformis
467	Bartonella birtlesii
468	Bartonella bovis
469	Bartonella clarridgeiae
470	Bartonella henselae
471	Bartonella schoenbuchensis
472	Bartonella tamiae
473	Bartonella tribocorum
474	Baudoinia compniacensis
475	Bdellovibrio bacteriovorus
476	Bdellovibrio exovorus
477	Beijerinckia indica
478	Belliella baltica
479	Bergeyella zoohelcum
480	Beta proteobacterium
481	beta proteobacterium
482	Beutenbergia cavernae
483	Bibersteinia trehalosi
484	Bifidobacterium adolescentis
485	Bifidobacterium angulatum
486	Bifidobacterium animalis
487	Bifidobacterium asteroides
488	Bifidobacterium bifidum
489	Bifidobacterium breve
490	Bifidobacterium choerinum
491	Bifidobacterium dentium
492	Bifidobacterium longum
493	Bifidobacterium pseudocatenulatum
494	Bifidobacterium pseudolongum
495	Bifidobacterium sp.
496	Bifidobacterium subtile
497	Bifidobacterium thermacidophilum
498	Bilophila wadsworthia
499	Bipolaris zeicola
500	Blastococcus saxobsidens
501	Blastococcus sp.
502	Blastomonas sp.
503	Blattabacterium sp.
504	Blautia hansenii
505	Blautia hydrogenotrophica
506	Blautia producta
507	Blautia sp.
508	Blautia wexlerae
509	Bombus impatiens
510	Bombus terrestris
511	Bombyx mori
512	Bordetella bronchiseptica
513	Bordetella hinzii
514	Bordetella holmesii
515	Bordetella parapertussis
516	Bordetella pertussis

517	Bordetella petrii
518	Bordetella phage
519	Bordetella sp.
520	Bordetella trematum
521	Borrelia anserina
522	Borrelia bissettii
523	Borrelia burgdorferi
524	Borrelia coriaceae
525	Borrelia crocidurae
526	Borrelia hermsii
527	Borrelia hispanica
528	Borrelia persica
529	Borrelia turicatae
530	Bos mutus
531	Bos taurus
532	Bosea sp.
533	Botryotinia fuckeliana
534	Brachybacterium faecium
535	Brachybacterium muris
536	Brachybacterium paraconglomeratum
537	Brachybacterium squillarum
538	Brachymonas chironomi
539	Brachymonas denitrificans
540	Brachypodium distachyon
541	Brachyspira alvinipulli
542	Brachyspira hyodysenteriae
543	Brachyspira innocens
544	Brachyspira murdochii
545	Brachyspira pilosicoli
546	Brachyspira sp.
547	Bradyrhizobium elkanii
548	Bradyrhizobium japonicum
549	Bradyrhizobium sp.
550	Branchiostoma floridae
551	Brassica rapa
552	BRC1 bacterium
553	Brenneria sp.
554	Brevibacillus agri
555	Brevibacillus borstelensis
556	Brevibacillus brevis
557	Brevibacillus laterosporus
558	Brevibacillus massiliensis,
559	Brevibacillus panacihumi
560	Brevibacillus thermoruber
561	Brevibacterium album
562	Brevibacterium linens
563	Brevibacterium massiliense
564	Brevibacterium senegalense,
565	Brevibacterium sp.
566	Brevundimonas aveniformis
567	Brevundimonas diminuta
568	Brevundimonas naejangsanensis
569	Brevundimonas sp.
570	Brevundimonas subvibrioides
571	Brucella sp.
572	Brugia malayi
573	Bubalus bubalis
574	Buchnera aphidicola

575	Budvicia aquatica
576	Burkholderia ambifaria
577	Burkholderia andropogonis
578	Burkholderia bannensis
579	Burkholderia bryophila
580	Burkholderia caledonica
581	Burkholderia cenocepacia
582	Burkholderia cepacia
583	Burkholderia dilworthii
584	Burkholderia dolosa
585	Burkholderia ferrariae
586	Burkholderia fungorum
587	Burkholderia gladioli
588	Burkholderia glumae
589	Burkholderia kururiensis
590	Burkholderia mallei
591	Burkholderia mimosarum
592	Burkholderia multivorans
593	Burkholderia nodosa
594	Burkholderia oklahomensis
595	Burkholderia oxyphila
596	Burkholderia phenoliruptrix
597	Burkholderia phymatum
598	Burkholderia phytofirmans
599	Burkholderia pseudomallei
600	Burkholderia pyrrocinia
601	Burkholderia rhizoxinica
602	Burkholderia sordidicola
603	Burkholderia sp.
604	Burkholderia sprentiae
605	Burkholderia thailandensis
606	Burkholderia ubonensis
607	Burkholderia vietnamiensis
608	Burkholderia xenovorans
609	Burkholderiales bacterium
610	Butyrate-producing bacterium
611	Butyricicoccus pullicaecorum
612	Butyricimonas synergistica
613	Butyricimonas virosa
614	Butyrivibrio crossotus
615	Butyrivibrio fibrisolvens
616	Butyrivibrio hungatei
617	Butyrivibrio proteoclasticus
618	Butyrivibrio sp.
619	Buzura suppressaria
620	Caenorhabditis briggsae
621	Caenorhabditis elegans
622	Caenorhabditis remanei
623	Caldanaerobacter subterraneus
624	Caldanaerobius polysaccharolyticus
625	Caldicellulosiruptor bescii
626	Caldicellulosiruptor hydrothermalis
627	Caldicellulosiruptor lactoaceticus
628	Caldicellulosiruptor saccharolyticus
629	Caldicoprobacter oshimai
630	Caldilinea aerophila
631	Caldimonas manganoxidans
632	Calditerrivibrio nitroreducens

633	Caldithrix abyssi
634	Calescamantes bacterium
635	Callithrix jacchus
636	Callorhinchus milii
637	Caloramator sp.
638	Calothrix sp.
639	Calypte anna
640	Camelus ferus
641	Campylobacter coli
642	Campylobacter cuniculorum
643	Campylobacter curvus
644	Campylobacter fetus
645	Campylobacter hominis
646	Campylobacter jejuni
647	Campylobacter lari
648	Campylobacter sp.
649	Campylobacter upsaliensis
650	Campylobacter ureolyticus
651	Campylobacterales bacterium
652	Candida albicans
653	Candida dubliniensis
654	Candida tropicalis
655	Candidate division
656	Candidatus Accumulibacter
657	Candidatus Alistipes
658	Candidatus Amoebophilus
659	Candidatus Ancillula
660	Candidatus Arthromitus
661	Candidatus Blochmannia
662	Candidatus Burkholderia
663	Candidatus Caldatribacterium
664	Candidatus Caldiarchaeum
665	Candidatus Chloracidobacterium
666	Candidatus Cloacamonas
667	Candidatus Desulforudis
668	Candidatus Endolissoclinum
669	Candidatus Hamiltonella
670	Candidatus Hepatobacter
671	Candidatus Hydrogenedens
672	Candidatus Kinetoplastibacterium
673	Candidatus Koribacter
674	Candidatus Nasuia
675	Candidatus Nitrospira
676	Candidatus Omnitrophus
677	Candidatus Pelagibacter
678	Candidatus Poribacteria
679	Candidatus Protochlamydia
680	Candidatus Puniceispirillum
681	Candidatus Rickettsia
682	Candidatus Saccharibacteria
683	Candidatus Saccharimonas
684	Candidatus Schmidhempelia
685	Candidatus Solibacter
686	Candidatus Stoquefichus
687	Candidatus Sulcia
688	Candidatus Uzinura
689	Candidatus Zinderia
690	Canis lupus

691	Capnocytophaga canimorsus
692	Capnocytophaga cynodegmi
693	Capnocytophaga granulosa
694	Capnocytophaga haemolytica
695	Capnocytophaga leadbetteri
696	Capnocytophaga sp.
697	Capra hircus
698	Capsaspora owczarzaki
699	Capsella rubella
700	Carboxydibrachium pacificum
701	Carboxydothermus hydrogenoformans
702	Cardinium endosymbiont
703	Cardiobacterium valvarum
704	Carnobacterium jeotgali
705	Carnobacterium maltaromaticum
706	Carnobacterium sp.
707	Catelliglobosispora koreensis
708	Catenibacterium sp.
709	Catenovulum agarivorans
710	Catenulispora acidiphila
711	Caulobacter crescentus
712	Caulobacter segnis
713	Caulobacter sp.
714	Caulobacteraceae bacterium
715	Cavia porcellus
716	Cedecea davisae
717	Cellulomonas fimi
718	Cellulomonas flavigena
719	Cellulomonas massiliensis
720	Cellulomonas sp.
721	Cellulophaga algicola
722	Cellulophaga baltica
723	Cellulophaga lytica
724	Cellulosimicrobium cellulans
725	Cellvibrio gilvus
726	Cellvibrio japonicus
727	Centipeda periodontii
728	Ceratitis capitata
729	Ceratotherium simum
730	Cetobacterium somerae
731	Chamaesiphon minutus
732	Chelativorans sp.
733	Chelatococcus sp.
734	Chelonia mydas
735	Chinchilla lanigera
736	Chitinibacter tainanensis
737	Chitinilyticum aquatile
738	Chitinilyticum litopenaei
739	Chitinimonas koreensis
740	Chitiniphilus shinanonensis
741	Chitinophaga pinensis
742	Chitinophaga sp.
743	Chlamydia muridarum
744	Chlamydia psittaci
745	Chlamydomonas reinhardtii
746	Chlamydophila sp.
747	Chlorobaculum parvum
748	Chlorobium chlorochromatii

749	Chlorobium limicola
750	Chlorobium luteolum
751	Chlorobium phaeobacteroides
752	Chlorobium phaeovibrioides
753	Chlorocebus sabaeus
754	Chloroflexi bacterium
755	Chloroflexus aggregans
756	Chloroflexus sp.
757	Chlorogloeopsis fritschii
758	Chloroherpeton thalassium
759	Choloepus hoffmanni
760	Chromobacterium violaceum
761	Chromohalobacter salexigens
762	Chroococcidiopsis thermalis
763	Chrysemys picta
764	Chryseobacterium aquaticum
765	Chryseobacterium caeni
766	Chryseobacterium daeguense
767	Chryseobacterium gleum
768	Chryseobacterium gregarium
769	Chryseobacterium haifense
770	Chryseobacterium hispalense
771	Chryseobacterium palustre
772	Chryseobacterium sp.
773	Chryseobacterium taeanense
774	Chryseobacterium treverense
775	Chrysochloris asiatica
776	Chthonomonas calidirosea
777	Cicer arietinum
778	Ciona intestinalis
779	Citreicella sp.
780	Citricoccus sp.
781	Citrobacter freundii
782	Citrobacter koseri
783	Citrobacter rodentium
784	Citrobacter sp.
785	Citrobacter youngae
786	Citromicrobium bathyomarinum
787	Citrus clementina
788	Citrus sinensis
789	Cladophialophora carrionii
790	Clavibacter michiganensis
791	Cloacibacterium haliotis
792	Cloacimonetes bacterium
793	Clostridiales bacterium
794	Clostridiales genomosp.
795	Clostridiisalibacter paucivorans
796	Clostridium acetobutylicum
797	Clostridium acidurici
798	Clostridium akagii
799	Clostridium algidicarnis
800	Clostridium arbusti
801	Clostridium asparagiforme
802	Clostridium autoethanogenum
803	Clostridium bartlettii
804	Clostridium beijerinckii
805	Clostridium bolteae
806	Clostridium botulinum

807	Clostridium butyricum
808	Clostridium cadaveris
809	Clostridium carboxidivorans
810	Clostridium celatum
811	Clostridium cellulolyticum
812	Clostridium cellulovorans
813	Clostridium cf.
814	Clostridium citroniae
815	Clostridium clariflavum
816	Clostridium clostridioforme
817	Clostridium colicanis
818	Clostridium difficile
819	Clostridium glycolicum
820	Clostridium hathewayi
821	Clostridium hiranonis
822	Clostridium hydrogeniformans
823	Clostridium hylemonae
824	Clostridium innocuum
825	Clostridium intestinale
826	Clostridium kluyveri
827	Clostridium lentocellum
828	Clostridium leptum
829	Clostridium ljungdahlii
830	Clostridium lundense
831	Clostridium methylpentosum
832	Clostridium nexile
833	Clostridium novyi
834	Clostridium paraputrificum
835	Clostridium pasteurianum
836	Clostridium perfringens
837	Clostridium phage
838	Clostridium phytofermentans
839	Clostridium ramosum
840	Clostridium saccharobutylicum
841	Clostridium saccharolyticum
842	Clostridium saccharoperbutylacetonicum
843	Clostridium scatologenes
844	Clostridium scindens
845	Clostridium senegalense
846	Clostridium sp.
847	Clostridium spiroforme
848	Clostridium sporogenes
849	Clostridium sporosphaeroides
850	Clostridium stercorarium
851	Clostridium symbiosum
852	Clostridium tetani
853	Clostridium thermocellum
854	Clostridium tunisiense
855	Clostridium tyrobutyricum
856	Clostridium ultunense
857	Cobetia crustatorum
858	Cohnella laeviribosi
859	Cohnella panacarvi
860	Cohnella thermotolerans
861	Collimonas fungivorans
862	Collinsella intestinalis
863	Collinsella sp.
864	Collinsella stercoris

865	Collinsella tanakaei
866	Columba livia
867	Colwellia piezophila
868	Colwellia psychrerythraea
869	Comamonadaceae bacterium
870	Comamonas badia
871	Comamonas composti
872	Comamonas sp.
873	Comamonas testosteroni
874	complete chromosome
875	Conchiformibius kuhniae
876	Conchiformibius steedae
877	Condylura cristata
878	Conexibacter woesei
879	Congregibacter litoralis
880	Coprobacillus sp.
881	Coprobacter fastidiosus
882	Coprococcus catus
883	Coprococcus comes
884	Coprococcus eutactus
885	Coprococcus sp.
886	Coprothermobacter platensis
887	Coraliomargarita akajimensis
888	Corallococcus coralloides
889	Corallococcus sp.
890	Cordyceps militaris
891	Coriobacteriaceae bacterium
892	Corvus brachyrhynchos
893	Corynebacterium accolens
894	Corynebacterium argentoratense
895	Corynebacterium aurimucosum
896	Corynebacterium bovis
897	Corynebacterium capitovis
898	Corynebacterium caspium
899	Corynebacterium crenatum
900	Corynebacterium diphtheriae
901	Corynebacterium doosanense
902	Corynebacterium durum
903	Corynebacterium efficiens
904	Corynebacterium freiburgense
905	Corynebacterium genitalium
906	Corynebacterium glucuronolyticum
907	Corynebacterium glutamicum
908	Corynebacterium halotolerans
909	Corynebacterium jeikeium
910	Corynebacterium kroppenstedtii
911	Corynebacterium lipophiloflavum
912	Corynebacterium lubricantis
913	Corynebacterium maris
914	Corynebacterium mastitidis
915	Corynebacterium nuruki
916	Corynebacterium pilosum
917	Corynebacterium pseudodiphtheriticum
918	Corynebacterium pseudogenitalium
919	Corynebacterium pseudotuberculosis
920	Corynebacterium pyruviciproducens
921	Corynebacterium resistens
922	Corynebacterium sp.

923	Corynebacterium sputi
924	Corynebacterium striatum
925	Corynebacterium terpenotabidum
926	Corynebacterium ulcerans
927	Corynebacterium ulceribovis
928	Corynebacterium urealyticum
929	Corynebacterium variabile
930	Corynebacterium-like bacterium
931	Cricetulus griseus
932	Crinalium epipsammum
933	Croceibacter atlanticus
934	Crocinitomix catalasitica
935	Cronobacter helveticus
936	Cronobacter pulveris
937	Cronobacter sakazakii
938	Cronobacter sp.
939	Cronobacter turicensis
940	Cronobacter zurichensis
941	Cryocola sp.
942	Cryptobacterium curtum
943	Cryptosporidium muris
944	Cucumis melo
945	Cucumis sativus
946	Culex pipiens
947	Cupriavidus metallidurans
948	Cupriavidus necator
949	Cupriavidus sp.
950	Cupriavidus taiwanensis
951	Curtobacterium flaccumfaciens
952	Curtobacterium sp.
953	Curvibacter gracilis
954	Curvibacter lanceolatus
955	Cyanidioschyzon merolae
956	Cyanobacterium UCYN-A,
957	Cyanobium gracile
958	Cyanophage MED4-213,
959	Cyanothece sp.
960	Cyclobacterium marinum
961	Cycloclasticus pugetii
962	Cylindrospermum stagnale
963	Cynoglossus semilaevis
964	Cytophaga aurantiaca
965	Cytophaga fermentans
966	Cytophaga hutchinsonii
967	Cytophagales bacterium
968	Dactylococcopsis salina
969	Danio rerio
970	Dasypus novemcinctus
971	Debaryomyces hansenii
972	Dechloromonas agitata
973	Dechloromonas aromatica
974	Dechlorosoma suillum
975	Deefgea rivuli
976	Deferribacter desulfuricans
977	Dehalobacter sp.
978	Dehalococcoides ethenogenes
979	Dehalococcoides mccartyi
980	Dehalococcoidia bacterium

981	Deinococcus apachensis
982	Deinococcus aquatilis
983	Deinococcus deserti
984	Deinococcus ficus
985	Deinococcus frigens
986	Deinococcus geothermalis
987	Deinococcus gobiensis
988	Deinococcus maricopensis
989	Deinococcus murrayi
990	Deinococcus peraridilitoris
991	Deinococcus pimensis
992	Deinococcus radiodurans
993	Deinococcus sp.
994	Deinococcus wulumuqiensis
995	Delftia acidovorans
996	Delftia sp.
997	delta proteobacterium
998	Delta proteobacterium
999	Demetria terragena
1000	Dermabacter sp.
1001	Dermatophilus congolensis
1002	Derxia gummosa
1003	Desmospora sp.
1004	Desulfarculus baarsii
1005	Desulfatibacillum alkenivorans
1006	Desulfatirhabdium butyrativorans
1007	Desulfitobacterium dichloroeliminans
1008	Desulfitobacterium hafniense
1009	Desulfobacca acetoxidans
1010	Desulfobacter curvatus
1011	Desulfobacter postgatei
1012	Desulfobacterium autotrophicum
1013	Desulfobacula toluolica
1014	Desulfobulbus elongatus
1015	Desulfobulbus mediterraneus
1016	Desulfobulbus propionicus
1017	Desulfocapsa sulfexigens
1018	Desulfococcus oleovorans
1019	Desulfocurvus vexinensis
1020	Desulfomicrobium escambiense
1021	Desulfonatronum lacustre
1022	Desulfonatronum thiodismutans
1023	Desulforegula conservatrix
1024	Desulfosarcina sp.
1025	Desulfospira joergensenii
1026	Desulfosporosinus meridiei
1027	Desulfosporosinus orientis
1028	Desulfosporosinus youngiae
1029	Desulfotignum balticum
1030	Desulfotomaculum acetoxidans
1031	Desulfotomaculum alcoholivorax
1032	Desulfotomaculum alkaliphilum
1033	Desulfotomaculum gibsoniae
1034	Desulfotomaculum kuznetsovii
1035	Desulfotomaculum nigrificans
1036	Desulfotomaculum reducens
1037	Desulfotomaculum ruminis
1038	Desulfovermiculus halophilus

1039	Desulfovibrio aespoeensis
1040	Desulfovibrio africanus
1041	Desulfovibrio alaskensis
1042	Desulfovibrio alcoholivorans
1043	Desulfovibrio aminophilus
1044	Desulfovibrio cf.
1045	Desulfovibrio desulfuricans
1046	Desulfovibrio frigidus
1047	Desulfovibrio gigas
1048	Desulfovibrio inopinatus
1049	Desulfovibrio magneticus
1050	Desulfovibrio piezophilus
1051	Desulfovibrio piger
1052	Desulfovibrio putealis
1053	Desulfovibrio sp.
1054	Desulfovibrio vulgaris
1055	Desulfovirgula thermocuniculi
1056	Desulfurispirillum indicum
1057	Desulfurispora thermophila
1058	Desulfurivibrio alkaliphilus
1059	Desulfurobacterium thermolithotrophum
1060	Desulfuromonas sp.
1061	Dialister micraerophilus
1062	Dialister pneumosintes
1063	Dialister sp.
1064	Dialister succinatiphilus
1065	Diaphorina citri
1066	Dichelobacter nodosus
1067	Dickeya chrysanthemi
1068	Dickeya dadantii
1069	Dickeya dianthicola
1070	Dickeya paradisiaca
1071	Dickeya solani
1072	Dickeya zeae
1073	Dictyoglomus thermophilum
1074	Dictyoglomus turgidum
1075	Dictyostelium discoideum
1076	Dictyostelium fasciculatum
1077	Dictyostelium purpureum
1078	Dielma fastidiosa
1079	Dietzia sp.
1080	Dinoroseobacter shibae
1081	Dolichospermum circinale
1082	Dolosigranulum pigrum
1083	Dongia sp.
1084	Dorea formicigenerans
1085	Dorea longicatena
1086	Dorea sp.
1087	Drosophila ananassae
1088	Drosophila erecta
1089	Drosophila grimshawi
1090	Drosophila melanogaster
1091	Drosophila mojavensis
1092	Drosophila persimilis
1093	Drosophila sechellia
1094	Drosophila simulans
1095	Drosophila virilis
1096	Drosophila willistoni

1097	Drosophila yakuba
1098	Duganella violaceinigra
1099	Duganella zoogloeoides
1100	Durinskia baltica
1101	Dyadobacter alkalitolerans
1102	Dyadobacter beijingensis
1103	Dyadobacter crusticola
1104	Dyadobacter fermentans
1105	Dyadobacter tibetensis
1106	Dyella ginsengisoli
1107	Dyella japonica
1108	Dysgonomonas capnocytophagoides
1109	Dysgonomonas gadei
1110	Dysgonomonas mossii
1111	Echinicola pacifica
1112	Echinicola vietnamensis
1113	Echinops telfairi
1114	Edwardsiella hoshinae
1115	Edwardsiella tarda
1116	Eggerthella lenta
1117	Eggerthella sp.
1118	Eggerthia catenaformis
1119	Ehrlichia canis
1120	Ehrlichia chaffeensis
1121	Ehrlichia ruminantium
1122	Elephantulus edwardii
1123	Elioraea tepidiphila
1124	Elizabethkingia anophelis
1125	Elizabethkingia meningoseptica
1126	Elusimicrobium minutum
1127	Emiliania huxleyi
1128	Empedobacter brevis
1129	Emticicia oligotrophica
1130	Endozoicomonas elysicola
1131	Ensifer adhaerens
1132	Ensifer sp.
1133	Entamoeba dispar
1134	Entamoeba histolytica
1135	Entamoeba invadens
1136	Entamoeba nuttalli
1137	Enterobacter aerogenes
1138	Enterobacter asburiae
1139	Enterobacter cloacae
1140	Enterobacter hormaechei
1141	Enterobacter mori
1142	Enterobacter radicincitans
1143	Enterobacter sp.
1144	Enterobacteriaceae bacterium
1145	Enterococcus avium
1146	Enterococcus caccae
1147	Enterococcus casseliflavus
1148	Enterococcus cecorum
1149	Enterococcus columbae
1150	Enterococcus dispar
1151	Enterococcus durans
1152	Enterococcus faecalis
1153	Enterococcus faecium
1154	Enterococcus gallinarum

1155	Enterococcus gilvus
1156	Enterococcus haemoperoxidus
1157	Enterococcus italicus
1158	Enterococcus moraviensis
1159	Enterococcus pallens
1160	Enterococcus phoeniculicola
1161	Enterococcus raffinosus
1162	Enterococcus saccharolyticus
1163	Enterococcus sp.
1164	Enterococcus sulfureus
1165	Enterorhabdus caecimuris
1166	Enterorhabdus mucosicola
1167	Enterovibrio calviensis
1168	Enterovibrio norvegicus
1169	Entomoplasma lucivorax
1170	Entomoplasma melaleucae
1171	Entomoplasma somnilux
1172	Epilithonimonas tenax
1173	Eptesicus fuscus
1174	Epulopiscium sp.
1175	Equus caballus
1176	Equus przewalskii
1177	Eremococcus coleocola
1178	Erinaceus europaeus
1179	Erwinia amylovora
1180	Erwinia billingiae
1181	Erwinia pyrifoliae
1182	Erwinia sp.
1183	Erwinia tasmaniensis
1184	Erwinia toletana
1185	Erwinia tracheiphila
1186	Erysipelothrix rhusiopathiae
1187	Erysipelothrix tonsillarum
1188	Erysipelotrichaceae bacterium
1189	Erythrobacter litoralis
1190	Erythrobacter sp.
1191	Escherichia albertii
1192	Escherichia blattae
1193	Escherichia coli
1194	Escherichia fergusonii
1195	Escherichia sp.
1196	Ethanoligenens harbinense
1197	Eubacteriaceae bacterium
1198	Eubacterium biforme
1199	Eubacterium brachy
1200	Eubacterium cellulosolvens
1201	Eubacterium cylindroides
1202	Eubacterium desmolans
1203	Eubacterium dolichum
1204	Eubacterium eligens
1205	Eubacterium hallii
1206	Eubacterium infirmum
1207	Eubacterium limosum
1208	Eubacterium plexicaudatum
1209	Eubacterium ramulus
1210	Eubacterium rectale
1211	Eubacterium saphenum
1212	Eubacterium siraeum

1213	Eubacterium sp.
1214	Eubacterium ventriosum
1215	Eubacterium xylanophilum
1216	Eubacterium yurii
1217	Eudoraea adriatica
1218	Eutrema salsugineum
1219	Exiguobacterium acetylicum
1220	Exiguobacterium antarcticum
1221	Exiguobacterium pavilionensis
1222	Exiguobacterium sibiricum
1223	Exiguobacterium sp.
1224	Facklamia hominis
1225	Facklamia ignava
1226	Facklamia languida
1227	Facklamia sourekii
1228	Faecalibacterium cf.
1229	Faecalibacterium prausnitzii
1230	Faecalibacterium sp.
1231	Falco cherrug
1232	Falco peregrinus
1233	Fangia hongkongensis
1234	Felis catus
1235	Ferrimonas balearica
1236	Ferrimonas kyonanensis
1237	Ferrimonas senticii
1238	Ferrovum myxofaciens
1239	Fervidobacterium nodosum
1240	Fervidobacterium pennivorans
1241	Fibrella aestuarina
1242	Fibrobacter succinogenes
1243	Ficedula albicollis
1244	Filifactor alocis
1245	Finegoldia magna
1246	Firmicutes bacterium
1247	Fischerella sp.
1248	Fischerella thermalis
1249	Flavobacteria bacterium
1250	Flavobacteriaceae bacterium
1251	Flavobacterium antarcticum
1252	Flavobacterium beibuense
1253	Flavobacterium branchiophilum
1254	Flavobacterium chungangense
1255	Flavobacterium columnare
1256	Flavobacterium daejeonense
1257	Flavobacterium denitrificans
1258	Flavobacterium filum
1259	Flavobacterium frigidarium
1260	Flavobacterium gelidilacus
1261	Flavobacterium indicum
1262	Flavobacterium johnsoniae
1263	Flavobacterium psychrophilum
1264	Flavobacterium rivuli
1265	Flavobacterium sasangense
1266	Flavobacterium soli
1267	Flavobacterium sp.
1268	Flavobacterium subsaxonicum
1269	Flavobacterium succinicans
1270	Flavobacterium suncheonense

1271	Flavobacterium tegetincola
1272	Flectobacillus major
1273	Flexibacter elegans
1274	Flexibacter litoralis
1275	Flexibacter roseolus
1276	Flexistipes sinusarabici
1277	Flexithrix dorotheae
1278	Fluoribacter dumoffii
1279	Fluviicola taffensis
1280	Fodinicurvata fenggangensis
1281	Fodinicurvata sediminis
1282	Fomitiporia mediterranea
1283	Fragaria vesca
1284	Francisella cf.
1285	Francisella noatunensis
1286	Francisella philomiragia
1287	Francisella sp.
1288	Frankia alni
1289	Frankia sp.
1290	Frankia symbiont
1291	Frateuria aurantia
1292	Fructobacillus fructosus
1293	Fusobacterium gonidiaformans
1294	Fusobacterium mortiferum
1295	Fusobacterium necrophorum
1296	Fusobacterium perfoetens
1297	Fusobacterium russii
1298	Fusobacterium sp.
1299	Fusobacterium ulcerans
1300	Fusobacterium varium
1301	Gaetbulibacter saemankumensis
1302	Gaeumannomyces graminis
1303	Galeopterus variegatus
1304	Gallibacterium anatis
1305	Gallionella capsiferriformans
1306	Gallionella sp.
1307	Gallus gallus
1308	gamma proteobacterium
1309	Gamma proteobacterium
1310	Gammaproteobacteria bacterium
1311	Gardnerella vaginalis
1312	Geitlerinema sp.
1313	Gelidibacter mesophilus
1314	Gemella bergeriae
1315	Gemella cuniculi
1316	Gemella sanguinis
1317	Geminicoccus roseus
1318	Geminocystis herdmanii
1319	Gemmata obscuriglobus
1320	Gemmatimonadetes bacterium
1321	Gemmatimonas aurantiaca
1322	Gemmatimonas sp.
1323	Gemmobacter nectariphilus
1324	Geobacillus caldoxylosilyticus
1325	Geobacillus sp.
1326	Geobacillus thermodenitrificans
1327	Geobacillus thermoglucosidans
1328	Geobacillus vulcani

1329	Geobacter bemidjiensis
1330	Geobacter daltonii
1331	Geobacter lovleyi
1332	Geobacter sp.
1333	Geobacter uraniireducens
1334	Geodermatophilaceae bacterium
1335	Geodermatophilus obscurus
1336	Geopsychrobacter electrodiphilus
1337	Geospiza fortis
1338	Geothrix fermentans
1339	Gillisia limnaea
1340	Gillisia marina
1341	Gillisia sp.
1342	Gilvimarinus chinensis
1343	Glaciecola sp.
1344	Glaciibacter superstes
1345	Glarea lozoyensis
1346	Gloeobacter kilaueensis
1347	Gloeocapsa sp.
1348	Gloeophyllum trabeum
1349	Glomeribacter sp.
1350	Gluconacetobacter diazotrophicus
1351	Gluconacetobacter hansenii
1352	Gluconacetobacter oboediens
1353	Gluconacetobacter sp.
1354	Gluconacetobacter xylinus
1355	Gluconobacter oxydans
1356	Glycine max
1357	Glycomyces arizonensis
1358	Glycomyces sp.
1359	Glycomyces tenuis
1360	Gordonia amicalis
1361	Gordonia bronchialis
1362	Gordonia hirsuta
1363	Gordonia kroppenstedtii
1364	Gordonia polyisoprenivorans
1365	Gordonia sp.
1366	Gordonia westfalica
1367	Gordonibacter pamelaeae
1368	Gorilla gorilla
1369	Gracilibacillus lacisalsi
1370	Gracilimonas tropica
1371	Gramella echinicola
1372	Gramella forsetii
1373	Gramella portivictoriae
1374	Granulibacter bethesdensis
1375	Granulicella mallensis
1376	Granulicoccus phenolivorans
1377	Gryllotalpicola daejeonensis
1378	Gryllotalpicola ginsengisoli
1379	Guillardia theta
1380	Gulosibacter molinativorax
1381	Haemophilus aegyptius
1382	Haemophilus ducreyi
1383	Haemophilus haemolyticus
1384	Haemophilus paraphrohaemolyticus
1385	Haemophilus parasuis
1386	Haemophilus phage

1387	Haemophilus somnus
1388	Haemophilus sp.
1389	Haemophilus sputorum
1390	Hafnia alvei
1391	Hahella chejuensis
1392	Halanaerobium hydrogeniformans
1393	Halanaerobium praevalens
1394	Haliangium ochraceum
1395	Haliea salexigens
1396	Haliscomenobacter hydrossis
1397	Hallella seregens
1398	Halobacillus halophilus
1399	Halobacterium salinarum
1400	Halobacteroides halobius
1401	Haloferax volcanii
1402	Haloglycomyces albus
1403	Halomonas anticariensis
1404	Halomonas boliviensis
1405	Halomonas halocynthiae
1406	Halomonas halodenitrificans
1407	Halomonas jeotgali
1408	Halomonas sp.
1409	Halomonas stevensii
1410	Halomonas zhanjiangensis
1411	Halomonas zincidurans
1412	Halonatronum saccharophilum
1413	Halopiger xanaduensis
1414	Halorhabdus utahensis
1415	Halorhodospira halochloris
1416	Halorubrum lacusprofundi
1417	Halotalea alkalilenta
1418	Halothermothrix orenii
1419	Halothiobacillus neapolitanus
1420	Halyomorpha halys
1421	Hamadaea tsunoensis
1422	Hammondia hammondi
1423	Helcococcus kunzii
1424	Helcococcus sueciensis
1425	Helicobacter acinonychis
1426	Helicobacter bilis
1427	Helicobacter canadensis
1428	Helicobacter canis
1429	Helicobacter cetorum
1430	Helicobacter cinaedi
1431	Helicobacter felis
1432	Helicobacter hepaticus
1433	Helicobacter macacae
1434	Helicobacter mustelae
1435	Helicobacter pametensis
1436	Helicobacter pullorum
1437	Helicobacter pylori
1438	Helicobacter rodentium
1439	Helicobacter winghamensis
1440	Heliobacterium modesticaldum
1441	Heliophilum fasciatum
1442	Helobdella robusta
1443	Henriciella marina
1444	Herbaspirillum frisingense

1445	Herbaspirillum lusitanum
1446	Herbaspirillum massiliense
1447	Herbaspirillum rubrisubalbicans
1448	Herbaspirillum seropedicae
1449	Herbidospora cretacea
1450	Herminiimonas arsenicoxydans
1451	Herminiimonas sp.
1452	Herpetosiphon aurantiacus
1453	Heterocephalus glaber
1454	Hippea maritima
1455	Hippea sp.
1456	Hirschia baltica
1457	Hoeflea sp.
1458	Holdemania filiformis
1459	Holophaga foetida
1460	Homo sapiens
1461	Humibacter albus
1462	Hydra magnipapillata
1463	Hydrogenovibrio marinus
1464	Hymenobacter aerophilus
1465	Hymenobacter norwichensis
1466	Hyphomicrobium denitrificans
1467	Hyphomicrobium nitrativorans
1468	Hyphomicrobium sp.
1469	Hyphomicrobium zavarzinii
1470	Hyphomonas neptunium
1471	Ichthyophthirius multifiliis
1472	Ideonella sp.
1473	Idiomarina baltica
1474	Idiomarina sediminum
1475	Ignatzschineria larvae
1476	Ignavibacterium album
1477	Ignisphaera aggregans
1478	Ilyobacter polytropus
1479	Inquilinus limosus
1480	Intrasporangiaceae bacterium
1481	Intrasporangium calvum
1482	Isoptericola variabilis
1483	Isosphaera pallida
1484	Ixodes scapularis
1485	Jaculus jaculus
1486	Janibacter sp.
1487	Janthinobacterium lividum
1488	Janthinobacterium sp.
1489	Jeotgalicoccus psychrophilus
1490	Jiangella gansuensis
1491	Johnsonella ignava
1492	Jonesia denitrificans
1493	Jonesia quinghaiensis
1494	Jonquetella anthropi
1495	Joostella marina
1496	Kaistia adipata
1497	Kaistia granuli
1498	Kamptonema formosum
1499	Kandleria vitulina
1500	Kangiella aquimarina
1501	Kangiella koreensis
1502	Ketogulonigenium vulgarum

1503	Kiloniella laminariae
1504	Kineococcus radiotolerans
1505	Kingella kingae
1506	Kingella oralis
1507	Kingella potus
1508	Kitasatospora setae
1509	Kitasatospora sp.
1510	Klebsiella oxytoca
1511	Klebsiella pneumoniae
1512	Klebsiella sp.
1513	Klebsiella variicola
1514	Kocuria atrinae
1515	Kocuria rhizophila
1516	Kocuria sp.
1517	Kordia algicida
1518	Kordiimonas gwangyangensis
1519	Kozakia baliensis
1520	Kribbella catacumbae
1521	Kribbella flavida
1522	Krokinobacter sp.
1523	Kurthia huakuii
1524	Kurthia sp.
1525	Kushneria aurantia
1526	Kutzneria albida
1527	Kyrpidia tusciae
1528	Kytococcus sedentarius
1529	Labrenzia alexandrii
1530	Labrenzia sp.
1531	Laccaria bicolor
1532	Laceyella sacchari
1533	Lachnoanaerobaculum orale
1534	Lachnobacterium bovis
1535	Lachnoclostridium phytofermentans
1536	Lachnospira multipara
1537	Lachnospiraceae bacterium
1538	Lachnospiraceae oral
1539	Lacinutrix sp.
1540	Lacticigenium naphtae
1541	Lactobacillus acidipiscis
1542	Lactobacillus acidophilus
1543	Lactobacillus amylovorus
1544	Lactobacillus antri
1545	Lactobacillus brevis
1546	Lactobacillus buchneri
1547	Lactobacillus casei
1548	Lactobacillus ceti
1549	Lactobacillus coleohominis
1550	Lactobacillus coryniformis
1551	Lactobacillus crispatus
1552	Lactobacillus delbrueckii
1553	Lactobacillus equi
1554	Lactobacillus fabifermentans
1555	Lactobacillus fuchuensis
1556	Lactobacillus gallinarum
1557	Lactobacillus gasseri
1558	Lactobacillus hamsteri
1559	Lactobacillus hayakitensis
1560	Lactobacillus helveticus

1561	Lactobacillus iners
1562	Lactobacillus jensenii
1563	Lactobacillus johnsonii
1564	Lactobacillus kitasatonis
1565	Lactobacillus malefermentans
1566	Lactobacillus mali
1567	Lactobacillus murinus
1568	Lactobacillus namurensis
1569	Lactobacillus paralimentarius
1570	Lactobacillus perolens
1571	Lactobacillus phage
1572	Lactobacillus plantarum
1573	Lactobacillus reuteri
1574	Lactobacillus rhamnosus
1575	Lactobacillus ruminis
1576	Lactobacillus sanfranciscensis
1577	Lactobacillus shenzhenensis
1578	Lactobacillus sp.
1579	Lactobacillus suebicus
1580	Lactobacillus ultunensis
1581	Lactobacillus vaginalis
1582	Lactobacillus vini
1583	Lactococcus garvieae
1584	Lactococcus lactis
1585	Lamprocystis purpurea
1586	Laribacter hongkongensis
1587	Latimeria chalumnae
1588	Lawsonia intracellularis
1589	Leadbetterella byssophila
1590	Lebetimonas sp.
1591	Lechevalieria aerocolonigenes
1592	Leeia oryzae
1593	Leeuwenhoekiella blandensis
1594	Leeuwenhoekiella sp.
1595	Legionella drancourtii
1596	Legionella lansingensis
1597	Legionella longbeachae
1598	Legionella moravica
1599	Legionella pneumophila
1600	Legionella sainthelensi
1601	Legionella shakespearei
1602	Legionella tunisiensis
1603	Legionella wadsworthii
1604	Leifsonia aquatica
1605	Leifsonia sp.
1606	Leifsonia xyli
1607	Leishmania major
1608	Leisingera methylohalidivorans
1609	Leisingera nanhaiensis
1610	Leminorella grimontii
1611	Lentzea albidocapillata
1612	Lepisosteus oculatus
1613	Leptolyngbya boryana
1614	Leptolyngbya sp.
1615	Leptonema illini
1616	Leptonychotes weddellii
1617	Leptospira biflexa
1618	Leptospira borgpetersenii

1619	Leptospira interrogans
1620	Leptospira kirschneri
1621	Leptospira licerasiae
1622	Leptospira weilii
1623	Leptothrix cholodnii
1624	Leucobacter chironomi
1625	Leucobacter chromiiresistens
1626	Leucobacter salsicius
1627	Leucobacter sp.
1628	Leuconostoc citreum
1629	Leuconostoc inhae
1630	Leuconostoc lactis
1631	Leuconostoc pseudomesenteroides
1632	Leucothrix mucor
1633	Lewinella cohaerens
1634	Lewinella persica
1635	Limnohabitans sp.
1636	Lipotes vexillifer
1637	Listeria grayi
1638	Listeria innocua
1639	Listeria ivanovii
1640	Listeria monocytogenes
1641	Listeria seeligeri
1642	Listeria welshimeri
1643	Listonella anguillarum
1644	Loa loa
1645	Loktanella vestfoldensis
1646	Longispora albida
1647	Lonsdalea quercina
1648	Lottia gigantea
1649	Loxodonta africana
1650	Luteimonas huabeiensis
1651	Luteimonas mephitis
1652	Luteimonas sp.
1653	Lutispora thermophila
1654	Lysinibacillus boronitolerans
1655	Lysinibacillus fusiformis
1656	Lysinibacillus sphaericus
1657	Lysobacter antibioticus
1658	Lysobacter defluvii
1659	Macaca fascicularis
1660	Macaca mulatta
1661	Macrococcus caseolyticus
1662	Magnaporthe oryzae
1663	Magnetococcus marinus
1664	Magnetospirillum gryphiswaldense
1665	Magnetospirillum magneticum
1666	Mahella australiensis
1667	Malus x
1668	Manacus vitellinus
1669	Mannheimia granulomatis
1670	Mannheimia haemolytica
1671	Mannheimia succiniciproducens
1672	Marchantia polymorpha
1673	Maribacter antarcticus
1674	Maribacter sp.
1675	Maricaulis maris
1676	Maricaulis sp.

1677	marine actinobacterium
1678	marine gamma
1679	Marinilabilia salmonicolor
1680	Marinimicrobia bacterium
1681	Marinimicrobium agarilyticum
1682	Marinimicrobium sp.
1683	Marinitoga piezophila
1684	Marinobacter daepoensis
1685	Marinobacter lipolyticus
1686	Marinobacter nanhaiticus
1687	Marinobacter sp.
1688	Marinobacterium jannaschii
1689	Marinobacterium litorale
1690	Marinobacterium rhizophilum
1691	Marinobacterium stanieri
1692	Marinomonas mediterranea
1693	Marinomonas posidonica
1694	Marinomonas sp.
1695	Marinospirillum insulare
1696	Marinospirillum minutulum
1697	Mariprofundus ferrooxydans
1698	Maritalea myrionectae
1699	Marivirga tractuosa
1700	Marmoricola aequoreus
1701	Marmoricola sp.
1702	Marssonina brunnea
1703	Martelella mediterranea
1704	Martelella sp.
1705	Massilia alkalitolerans
1706	Massilia niastensis
1707	Massilia timonae
1708	Mastigocladopsis repens
1709	Mastigocoleus testarum
1710	Maylandia zebra
1711	Medicago truncatula
1712	Megachile rotundata
1713	Megamonas funiformis
1714	Megamonas hypermegale
1715	Meganema perideroedes
1716	Megasphaera elsdenii
1717	Megavirus terra1
1718	Meiothermus cerbereus
1719	Meiothermus chliarophilus
1720	Meiothermus ruber
1721	Meiothermus silvanus
1722	Meiothermus timidus
1723	Meleagris gallopavo
1724	Melioribacter roseus
1725	Melitea salexigens
1726	Melopsittacus undulatus
1727	Merops nubicus
1728	Mesocricetus auratus
1729	Mesoflavibacter zeaxanthinifaciens
1730	Mesonia mobilis
1731	Mesoplasma chauliocola
1732	Mesoplasma florum
1733	Mesoplasma grammopterae
1734	Mesoplasma photuris

1735	Mesoplasma seiffertii
1736	Mesorhizobium ciceri
1737	Mesorhizobium loti
1738	Mesorhizobium sp.
1739	Metarhizium anisopliae
1740	Metascardovia criceti
1741	Metaseiulus occidentalis
1742	Methanobrevibacter ruminantium
1743	Methanobrevibacter smithii
1744	Methanocaldococcus jannaschii
1745	Methanocaldococcus villosus
1746	Methanocella arvoryzae
1747	Methanococcus maripaludis
1748	Methanococcus voltae
1749	Methanoculleus marisnigri
1750	Methanohalobium evestigatum
1751	Methanohalophilus mahii
1752	Methanolobus psychrophilus
1753	Methanomassiliicoccus sp.
1754	Methanomethylovorans hollandica
1755	Methanopyrus kandleri
1756	Methanoregula formicicum
1757	Methanosaeta harundinacea
1758	Methanosalsum zhilinae
1759	Methanotorris igneus
1760	Methylibium petroleiphilum
1761	Methylobacillus flagellatus
1762	Methylobacillus glycogenes
1763	Methylobacter luteus
1764	Methylobacter marinus
1765	Methylobacter tundripaludum
1766	Methylobacterium extorquens
1767	Methylobacterium nodulans
1768	Methylobacterium populi
1769	Methylobacterium sp.
1770	Methylocaldum szegediense
1771	Methylococcus capsulatus
1772	Methylocystis parvus
1773	Methyloferula stellata
1774	Methylohalobius crimeensis
1775	Methylomarinum vadi
1776	Methylomicrobium agile
1777	Methylomicrobium album
1778	Methylomicrobium alcaliphilum
1779	Methylomicrobium buryatense
1780	Methylomonas methanica
1781	Methylomonas sp.
1782	Methylophaga sp.
1783	Methylophaga thiooxidans
1784	Methylophilaceae bacterium
1785	Methylophilus methylotrophus
1786	Methylophilus sp.
1787	Methylopila sp.
1788	Methylosarcina fibrata
1789	Methylosarcina lacus
1790	Methylosinus sp.
1791	Methylotenera mobilis
1792	Methylotenera sp.
1793	Methylotenera versatilis
1794	Methyloversatilis sp.
1795	Methyloversatilis universalis
1796	Methylovorus glucosetrophus
1797	Methylovorus sp.
1798	Methylovulum miyakonense
1799	Micavibrio aeruginosavorus
1800	Microbacterium barkeri
1801	Microbacterium gubbeenense
1802	Microbacterium indicum
1803	Microbacterium luticocti
1804	Microbacterium maritypicum
1805	Microbacterium paraoxydans
1806	Microbacterium sp.
1807	Microbacterium testaceum
1808	Microbacterium yannicii
1809	Microbispora rosea
1810	Microbispora sp.
1811	Microbulbifer agarilyticus
1812	Microbulbifer variabilis
1813	Microcebus murinus
1814	Micrococcus luteus
1815	Microcoleus chthonoplastes
1816	Microcoleus sp.
1817	Microcystis aeruginosa
1818	Microlunatus phosphovorus
1819	Micromonas pusilla
1820	Micromonas sp.
1821	Micromonospora chokoriensis
1822	Micromonospora globosa
1823	Micromonospora lupini
1824	Micromonospora parva
1825	Micromonospora purpureochromogenes
1826	Micromonospora sp.
1827	Microplitis demolitor
1828	Microtetraspora glauca
1829	Microtus ochrogaster
1830	Microvirga sp.
1831	Microvirgula aerodenitrificans
1832	Millerozyma farinosa
1833	Mimivirus terra2
1834	Mitsuokella multacida
1835	Mitsuokella sp.
1836	Mobiluncus curtisii
1837	Mobiluncus mulieris
1838	Modestobacter marinus,
1839	Mogibacterium neglectum
1840	Mogibacterium vescum
1841	Moniliophthora perniciosa
1842	Monodelphis domestica
1843	Moorella thermoacetica
1844	Moraxella boevrei
1845	Moraxella caprae
1846	Moraxella catarrhalis
1847	Morganella morganii
1848	Moritella dasanensis
1849	Moritella marina
1850	Mucilaginibacter paludis

1851	Mucispirillum schaedleri
1852	Muricauda ruestringensis
1853	Mus musculus
1854	Musca domestica
1855	Mustela putorius
1856	Mycetocola saprophilus
1857	Mycobacterium abscessus
1858	Mycobacterium avium
1859	Mycobacterium canettii
1860	Mycobacterium chubuense
1861	Mycobacterium fortuitum
1862	Mycobacterium genavense
1863	Mycobacterium gilvum
1864	Mycobacterium hassiacum
1865	Mycobacterium iranicum
1866	Mycobacterium kansasii
1867	Mycobacterium marinum
1868	Mycobacterium neoaurum
1869	Mycobacterium parascrofulaceum
1870	Mycobacterium rhodesiae
1871	Mycobacterium smegmatis
1872	Mycobacterium sp.
1873	Mycobacterium tusciae
1874	Mycobacterium ulcerans
1875	Mycobacterium vaccae
1876	Mycobacterium vanbaalenii
1877	Mycobacterium yongonense
1878	Mycoplasma bovis
1879	Mycoplasma capricolum
1880	Mycoplasma columbinum
1881	Mycoplasma conjunctivae
1882	Mycoplasma cricetuli
1883	Mycoplasma cynos
1884	Mycoplasma elephantis
1885	Mycoplasma fermentans
1886	Mycoplasma glycophilum
1887	Mycoplasma hominis
1888	Mycoplasma hyopneumoniae
1889	Mycoplasma hyorhinis
1890	Mycoplasma leachii
1891	Mycoplasma leonicaptivi
1892	Mycoplasma lipofaciens
1893	Mycoplasma moatsii
1894	Mycoplasma molare
1895	Mycoplasma mycoides
1896	Mycoplasma orale
1897	Mycoplasma ovipneumoniae
1898	Mycoplasma penetrans
1899	Mycoplasma primatum
1900	Mycoplasma pulmonis
1901	Mycoplasma putrefaciens
1902	Mycoplasma salivarium
1903	Mycoplasma sp.
1904	Mycoplasma synoviae
1905	Mycoplasma wenyonii
1906	Mycosphaerella graminicola
1907	Myotis brandtii
1908	Myotis davidii
1909	Myotis lucifugus
1910	Myroides injenensis
1911	Myroides odoratimimus
1912	Myroides odoratus
1913	Myxococcus fulvus
1914	Myxococcus stipitatus
1915	Myxococcus xanthus
1916	Naegleria gruberi
1917	Nafulsella turpanensis
1918	Nakamurella lactea
1919	Nakamurella multipartita
1920	Nannochloropsis gaditana
1921	Nannospalax galili
1922	Nanoarchaeota archaeon
1923	Nasonia vitripennis
1924	Natranaerobius thermophilus
1925	Natrinema pellirubrum
1926	Natrinema sp.
1927	Natronobacterium gregoryi
1928	Natronomonas pharaonis
1929	Naumovozyma dairenensis
1930	Nautilia profundicola
1931	Neisseria bacilliformis
1932	Neisseria lactamica
1933	Neisseria macacae
1934	Neisseria perflava
1935	Neisseria shayeganii
1936	Neisseria sp.
1937	Neisseria wadsworthii
1938	Nematostella vectensis
1939	Neolamprologus brichardi
1940	Neosartorya fischeri
1941	Neospora caninum
1942	Neptunomonas japonica
1943	Nesterenkonia alba
1944	Nesterenkonia sp.
1945	Neurospora crassa
1946	Nevskia ramosa
1947	Nevskia soli
1948	Niabella aurantiaca
1949	Niastella koreensis
1950	Nitratifractor salsuginis
1951	Nitratireductor aquibiodomus
1952	Nitratiruptor sp.
1953	Nitrobacter sp.
1954	Nitrococcus mobilis
1955	Nitrosococcus halophilus
1956	Nitrosococcus oceani
1957	Nitrosomonas cryotolerans
1958	Nitrosomonas europaea
1959	Nitrosomonas eutropha
1960	Nitrosomonas sp.
1961	Nitrosospira briensis
1962	Nitrosospira multiformis
1963	Nitrospirillum amazonense
1964	Nocardia brasiliensis
1965	Nocardia cyriacigeorgica
1966	Nocardia farcinica

1967	Nocardia otitidiscaviarum
1968	Nocardia rhamnosiphila
1969	Nocardia sp.
1970	Nocardioidaceae bacterium
1971	Nocardioides alkalitolerans
1972	Nocardioides halotolerans
1973	Nocardioides insulae
1974	Nocardioides sp.
1975	Nocardiopsis alba
1976	Nocardiopsis chromatogenes
1977	Nocardiopsis dassonvillei
1978	Nocardiopsis ganjiahuensis
1979	Nocardiopsis gilva
1980	Nocardiopsis halophila
1981	Nocardiopsis halotolerans
1982	Nocardiopsis kunsanensis
1983	Nocardiopsis lucentensis
1984	Nocardiopsis potens
1985	Nocardiopsis prasina
1986	Nocardiopsis salina
1987	Nocardiopsis sp.
1988	Nocardiopsis synnemataformans
1989	Nocardiopsis valliformis
1990	Nocardiopsis xinjiangensis
1991	Nodosilinea nodulosa
1992	Nomascus leucogenys
1993	Nonlabens dokdonensis
1994	Nonomuraea coxensis
1995	Nostoc sp.
1996	Novispirillum itersonii
1997	Novosphingobium lindaniclasticum
1998	Novosphingobium nitrogenifigens
1999	Novosphingobium sp.
2000	Oceanicaulis alexandrii
2001	Oceanicola batsensis
2002	Oceanicola granulosus
2003	Oceanicola sp.
2004	Oceanimonas smirnovii
2005	Oceanimonas sp.
2006	Oceanithermus profundus
2007	Oceanobacillus iheyensis
2008	Oceanobacter sp.
2009	Oceanospirillales bacterium
2010	Oceanospirillum maris
2011	Oceanospirillum sp.
2012	Ochotona princeps
2013	Ochrobactrum anthropi
2014	Ochrobactrum intermedium
2015	Ochrobactrum rhizosphaerae
2016	Ochrobactrum sp.
2017	Octadecabacter antarcticus
2018	Octadecabacter arcticus
2019	Octodon degus
2020	Odobenus rosmarus
2021	Odoribacter laneus
2022	Odoribacter sp.
2023	Odoribacter splanchnicus
2024	Oenococcus oeni

2025	Oerskovia turbata
2026	Oligella ureolytica
2027	Olivibacter sitiensis
2028	Olleya marilimosa
2029	Olleya sp.
2030	Olsenella sp.
2031	Olsenella uli
2032	Omnitrophica bacterium
2033	Opisthorchis viverrini
2034	Opitutaceae bacterium
2035	Opitutus terrae
2036	Orcinus orca
2037	Orenia marismortui
2038	Oreochromis niloticus
2039	Oribacterium sp.
2040	Orientia tsutsugamushi
2041	Ornithinimicrobium pekingense
2042	Ornithobacterium rhinotracheale
2043	Ornithorhynchus anatinus
2044	Orycteropus afer
2045	Oryctolagus cuniculus
2046	Oryza brachyantha
2047	Oryza sativa
2048	Oryzias latipes
2049	Oscillatoria acuminata
2050	Oscillatoria sp.
2051	Oscillatoriales cyanobacterium
2052	Oscillibacter sp.
2053	Oscillibacter valericigenes
2054	Oscillochloris trichoides
2055	Ostreococcus tauri
2056	Otolemur garnettii
2057	Ottowia thiooxydans
2058	Ovis aries
2059	Owenweeksia hongkongensis
2060	Oxalobacter formigenes
2061	Oxalobacteraceae bacterium
2062	Paenibacillus alginolyticus
2063	Paenibacillus assamensis
2064	Paenibacillus azotofixans
2065	Paenibacillus barengoltzii
2066	Paenibacillus daejeonensis
2067	Paenibacillus ehimensis
2068	Paenibacillus elgii
2069	Paenibacillus forsythiae
2070	Paenibacillus ginsengihumi
2071	Paenibacillus graminis
2072	Paenibacillus harenae
2073	Paenibacillus larvae
2074	Paenibacillus massiliensis
2075	Paenibacillus mucilaginosus
2076	Paenibacillus pasadenensis
2077	Paenibacillus peoriae
2078	Paenibacillus polymyxa
2079	Paenibacillus sp.
2080	Paenibacillus taiwanensis
2081	Paenibacillus terrae
2082	Paenibacillus zanthoxyli

2083	Paenisporosarcina sp.
2084	Paludibacter propionicigenes
2085	Paludibacterium yongneupense
2086	Pan paniscus
2087	Pan troglodytes
2088	Pandoraea pnomenusa
2089	Pandoraea sp.
2090	Pannonibacter phragmitetus
2091	Panthera tigris
2092	Pantholops hodgsonii
2093	Pantoea agglomerans
2094	Pantoea ananatis
2095	Pantoea dispersa
2096	Pantoea sp.
2097	Pantoea vagans
2098	Papio anubis
2099	Parabacteroides chartae
2100	Parabacteroides distasonis
2101	Parabacteroides goldsteinii
2102	Parabacteroides gordonii
2103	Parabacteroides johnsonii
2104	Parabacteroides sp.
2105	Parachlamydia acanthamoebae
2106	Paracoccidioides brasiliensis
2107	Paracoccus aminophilus
2108	Paracoccus denitrificans
2109	Paracoccus pantotrophus
2110	Paracoccus sp.
2111	Paracoccus yeei
2112	Paramecium tetraurelia
2113	Paraoerskovia marina
2114	Paraprevotella clara
2115	Paraprevotella xylaniphila
2116	Parasutterella excrementihominis
2117	Parcubacteria bacterium
2118	Parvibaculum lavamentivorans
2119	Parvimonas micra
2120	Parvularcula bermudensis
2121	Parvularcula oceani
2122	Pasteurella dagmatis
2123	Pasteurella multocida
2124	Pasteurella pneumotropica
2125	Patulibacter americanus
2126	Patulibacter minatonensis
2127	Paucisalibacillus globulus
2128	Pectobacterium carotovorum
2129	Pectobacterium sp.
2130	Pediculus humanus
2131	Pediococcus acidilactici
2132	Pediococcus pentosaceus
2133	Pedobacter agri
2134	Pedobacter arcticus
2135	Pedobacter borealis
2136	Pedobacter glucosidilyticus
2137	Pedobacter heparinus
2138	Pedobacter oryzae
2139	Pedobacter saltans
2140	Pelagibaca bermudensis

2141	Pelagibacterium halotolerans
2142	Pelistega sp.
2143	Pelobacter carbinolicus
2144	Pelobacter seleniigenes
2145	Pelodiscus sinensis
2146	Pelosinus sp.
2147	Penicillium marneffei
2148	Peptoclostridium difficile
2149	Peptococcus niger
2150	Peptostreptococcaceae bacterium
2151	Peptostreptococcus anaerobius
2152	Perlucidibaca piscinae
2153	Peromyscus maniculatus
2154	Persephonella marina
2155	Persephonella sp.
2156	Phaeobacter arcticus
2157	Phaeobacter caeruleus
2158	Phaeobacter gallaeciensis
2159	Phanerochaete carnosa
2160	Phascolarctobacterium sp.
2161	Phascolarctobacterium succinatutens
2162	Phaseolus vulgaris
2163	Phenylobacterium zucineum
2164	Phoenix dactylifera
2165	Photobacterium leiognathi
2166	Photobacterium profundum
2167	Photorhabdus asymbiotica,
2168	Photorhabdus luminescens
2169	Photorhabdus temperata
2170	Phycicoccus jejuensis
2171	Phycisphaera mikurensis
2172	Physcomitrella patens
2173	Physeter catodon
2174	Phytophthora infestans
2175	Phytophthora parasitica
2176	Pilimelia anulata
2177	Planctomyces limnophilus
2178	Planktothrix prolifica
2179	Plasmodium berghei
2180	Plasmodium chabaudi
2181	Plasmodium cynomolgi
2182	Plasmodium falciparum
2183	Plasmodium knowlesi
2184	Plasmodium vivax
2185	Plasmodium yoelii
2186	Platanus occidentalis
2187	Plautia stali
2188	Pleomorphomonas koreensis
2189	Pleomorphomonas oryzae
2190	Plesiomonas shigelloides
2191	Pleurocapsa sp.
2192	Poecilia formosa
2193	Poecilia reticulata
2194	Polaribacter irgensii
2195	Polaribacter sp.
2196	Polaromonas glacialis
2197	Polaromonas naphthalenivorans
2198	Polaromonas sp.

2199	Polycyclovorans algicola
2200	Polymorphum gilvum
2201	Polynucleobacter necessarius
2202	Pongo abelii
2203	Pontibacillus halophilus
2204	Pontibacillus marinus
2205	Pontibacter actiniarum
2206	Pontibacter roseus
2207	Ponticaulis koreensis
2208	Populus trichocarpa
2209	Porphyrobacter cryptus
2210	Porphyromonas asaccharolytica
2211	Porphyromonas bennonis
2212	Porphyromonas cangingivalis
2213	Porphyromonas catoniae
2214	Porphyromonas gingivalis
2215	Porphyromonas gingivicanis
2216	Porphyromonas gulae
2217	Porphyromonas levii
2218	Porphyromonas macacae
2219	Porphyromonas somerae
2220	Porphyromonas sp.
2221	Porphyromonas uenonis
2222	Prasinoderma coloniale
2223	Prauserella rugosa
2224	Prevotella albensis
2225	Prevotella amnii
2226	Prevotella aurantiaca
2227	Prevotella baroniae
2228	Prevotella bergensis
2229	Prevotella bivia
2230	Prevotella brevis
2231	Prevotella bryantii
2232	Prevotella buccae
2233	Prevotella corporis
2234	Prevotella dentalis
2235	Prevotella dentasini
2236	Prevotella denticola
2237	Prevotella disiens
2238	Prevotella enoeca
2239	Prevotella falsenii
2240	Prevotella fusca
2241	Prevotella histicola
2242	Prevotella intermedia
2243	Prevotella jejuni
2244	Prevotella loescheii
2245	Prevotella maculosa
2246	Prevotella melaninogenica
2247	Prevotella micans
2248	Prevotella multiformis
2249	Prevotella multisaccharivorax
2250	Prevotella nanceiensis
2251	Prevotella nigrescens
2252	Prevotella oralis
2253	Prevotella oulorum
2254	Prevotella pallens
2255	Prevotella paludivivens
2256	Prevotella pleuritidis

2257	Prevotella ruminicola
2258	Prevotella saccharolytica
2259	Prevotella scopos
2260	Prevotella shahii
2261	Prevotella sp.
2262	Prevotella stercorea
2263	Prevotella timonensis
2264	Procavia capensis
2265	Prochlorococcus marinus
2266	Prochlorococcus sp.
2267	Prolixibacter bellariivorans
2268	Promicromonospora sukumoe
2269	Propionibacteriaceae bacterium
2270	Propionibacterium acidifaciens
2271	Propionibacterium acidipropionici
2272	Propionibacterium avidum
2273	Propionibacterium freudenreichii
2274	Propionibacterium jensenii
2275	Propionibacterium propionicum
2276	Propionibacterium sp.
2277	Propionibacterium thoenii
2278	Propionicicella superfundia
2279	Propionigenium modestum
2280	Propionimicrobium lymphophilum
2281	Propionivibrio pelophilus
2282	Prosthecochloris aestuarii
2283	Proteiniclasticum ruminis
2284	Proteiniphilum acetatigenes
2285	Proteobacteria bacterium
2286	Proteocatella sphenisci
2287	Proteus mirabilis
2288	Proteus penneri
2289	Providencia alcalifaciens
2290	Providencia burhodogranariea
2291	Providencia rettgeri
2292	Providencia rustigianii
2293	Providencia sneebia
2294	Providencia stuartii
2295	Prunus mume
2296	Pseudaminobacter salicylatoxidans
2297	Pseudoalteromonas flavipulchra
2298	Pseudoalteromonas haloplanktis
2299	Pseudoalteromonas sp.
2300	Pseudobutyrivibrio ruminis
2301	Pseudobutyrivibrio sp.
2302	Pseudochrobactrum sp.
2303	Pseudoclavibacter soli
2304	Pseudoduganella violaceinigra
2305	Pseudogulbenkiania ferrooxidans
2306	Pseudogulbenkiania sp.
2307	Pseudomonas aeruginosa
2308	Pseudomonas agarici
2309	Pseudomonas alcaligenes
2310	Pseudomonas azotifigens
2311	Pseudomonas caeni
2312	Pseudomonas chloritidismutans
2313	Pseudomonas chlororaphis
2314	Pseudomonas corrugata

2315	Pseudomonas cremoricolorata
2316	Pseudomonas denitrificans
2317	Pseudomonas entomophila
2318	Pseudomonas flectens
2319	Pseudomonas fluorescens
2320	Pseudomonas fragi
2321	Pseudomonas fulva
2322	Pseudomonas fuscovaginae
2323	Pseudomonas mendocina
2324	Pseudomonas monteilii
2325	Pseudomonas moraviensis
2326	Pseudomonas mosselii
2327	Pseudomonas nitroreducens
2328	Pseudomonas oleovorans
2329	Pseudomonas pelagia
2330	Pseudomonas plecoglossicida
2331	Pseudomonas poae
2332	Pseudomonas protegens
2333	Pseudomonas psychrophila
2334	Pseudomonas putida
2335	Pseudomonas resinovorans
2336	Pseudomonas sp.
2337	Pseudomonas stutzeri
2338	Pseudomonas syringae
2339	Pseudomonas taeanensis
2340	Pseudomonas taiwanensis
2341	Pseudomonas thermotolerans
2342	Pseudomonas veronii
2343	Pseudomonas viridiflava
2344	Pseudomonas vranovensis
2345	Pseudonocardia acaciae
2346	Pseudonocardia asaccharolytica
2347	Pseudonocardia dioxanivorans
2348	Pseudonocardia sp.
2349	Pseudopodoces humilis
2350	Pseudoramibacter alactolyticus
2351	Pseudorhodobacter ferrugineus
2352	Pseudovibrio sp.
2353	Pseudoxanthomonas sp.
2354	Pseudoxanthomonas spadix
2355	Pseudoxanthomonas suwonensis
2356	Pseudozyma flocculosa
2357	Psychrilyobacter atlanticus
2358	Psychrobacter arcticus
2359	Psychrobacter cryohalolentis
2360	Psychrobacter lutiphocae
2361	Psychrobacter phenylpyruvicus
2362	Psychrobacter sp.
2363	Psychroflexus torquis
2364	Psychroflexus tropicus
2365	Psychromonas aquimarina
2366	Psychromonas arctica
2367	Psychromonas hadalis
2368	Psychromonas ingrahamii
2369	Psychromonas sp.
2370	Psychroserpens burtonensis
2371	Pteropus alecto
2372	Pteropus vampyrus

2373	Pundamilia nyererei
2374	Pusillimonas noertemannii
2375	Pusillimonas sp.
2376	Pyrenophora teres
2377	Pyrenophora tritici-repentis
2378	Python bivittatus
2379	Rahnella aquatilis
2380	Ralstonia eutropha
2381	Ralstonia pickettii
2382	Ralstonia solanacearum
2383	Ralstonia sp.
2384	Ramlibacter tataouinensis
2385	Rathayibacter toxicus
2386	Rattus norvegicus
2387	Reinekea sp.
2388	Renibacterium salmoninarum
2389	Rheinheimera baltica
2390	Rheinheimera perlucida
2391	Rheinheimera texasensis
2392	Rhizobiales bacterium
2393	Rhizobium etli
2394	Rhizobium giardinii
2395	Rhizobium leguminosarum
2396	Rhizobium leucaenae
2397	Rhizobium mesoamericanum
2398	Rhizobium mongolense
2399	Rhizobium phaseoli
2400	Rhizobium sp.
2401	Rhizobium sullae
2402	Rhizobium tropici
2403	Rhizobium undicola
2404	Rhodanobacter sp.
2405	Rhodobacter capsulatus
2406	Rhodobacter sphaeroides
2407	Rhodobacteraceae bacterium
2408	Rhodobacterales bacterium
2409	Rhodococcus equi
2410	Rhodococcus erythropolis
2411	Rhodococcus fascians
2412	Rhodococcus jostii
2413	Rhodococcus opacus
2414	Rhodococcus pyridinivorans
2415	Rhodococcus rhodochrous
2416	Rhodococcus ruber
2417	Rhodococcus sp.
2418	Rhodoferax ferrireducens
2419	Rhodoferax saidenbachensis
2420	Rhodonellum psychrophilum
2421	Rhodopirellula baltica
2422	Rhodopseudomonas palustris
2423	Rhodopseudomonas sp.
2424	Rhodospirillales bacterium
2425	Rhodospirillum centenum
2426	Rhodospirillum photometricum
2427	Rhodospirillum rubrum
2428	Rhodothermus marinus
2429	Rhodovibrio salinarum
2430	Ricinus communis

2431	Rickettsia australis
2432	Rickettsia canadensis
2433	Rickettsia montanensis
2434	Rickettsia typhi
2435	Rickettsiaceae bacterium
2436	Riemerella anatipestifer
2437	Riemerella columbina
2438	Rikenella microfusus
2439	Rivularia sp.
2440	Robiginitalea biformata
2441	Robinsoniella sp.
2442	Roseburia hominis
2443	Roseburia intestinalis
2444	Roseburia inulinivorans
2445	Roseburia sp.
2446	Roseiflexus castenholzii
2447	Roseiflexus sp.
2448	Roseobacter denitrificans
2449	Roseobacter litoralis
2450	Roseobacter sp.
2451	Roseomonas cervicalis
2452	Roseovarius sp.
2453	Rothia aeria
2454	Ruania albidiflava
2455	Rubellimicrobium thermophilum
2456	Rubritalea marina
2457	Rubritepida flocculans
2458	Rubrivivax benzoatilyticus
2459	Rubrivivax gelatinosus
2460	Rubrobacter xylanophilus
2461	Rudaea cellulosilytica
2462	Rudanella lutea
2463	Ruegeria conchae
2464	Ruegeria pomeroyi
2465	Ruegeria sp.
2466	Ruminobacter sp.
2467	Ruminococcaceae bacterium
2468	Ruminococcus albus
2469	Ruminococcus bromii
2470	Ruminococcus callidus
2471	Ruminococcus champanellensis
2472	Ruminococcus flavefaciens
2473	Ruminococcus gauvreauii
2474	Ruminococcus gnavus
2475	Ruminococcus lactaris
2476	Ruminococcus obeum
2477	Ruminococcus sp.
2478	Ruminococcus torques
2479	Runella limosa
2480	Runella slithyformis
2481	Runella zeae
2482	Saccharibacillus kuerlensis
2483	Saccharomonospora azurea
2484	Saccharomonospora cyanea
2485	Saccharomonospora glauca
2486	Saccharomonospora marina
2487	Saccharomonospora paurometabolica
2488	Saccharomonospora viridis
2489	Saccharomonospora xinjiangensis
2490	Saccharomyces cerevisiae
2491	Saccharopolyspora erythraea
2492	Saccharopolyspora rectivirgula
2493	Saccharopolyspora spinosa
2494	Saccharospirillum impatiens
2495	Saccharothrix espanaensis
2496	Saccoglossus kowalevskii
2497	Saimiri boliviensis
2498	Salana multivorans
2499	Salinarimonas rosea
2500	Salinibacter ruber
2501	Salinicoccus carnicancri
2502	Salinimicrobium terrae
2503	Salinimicrobium xinjiangense
2504	Salinimonas chungwhensis
2505	Salinispora arenicola
2506	Salinispora pacifica
2507	Salinivibrio socompensis
2508	Salipiger mucosus
2509	Salisaeta longa
2510	Salmonella enterica
2511	Salpingoeca sp.
2512	Salsuginibacillus kocurii
2513	Sandarakinorhabdus sp.
2514	Sanguibacter keddieii
2515	Saprolegnia diclina
2516	Saprospira grandis
2517	SAR324 cluster
2518	SAR86 cluster
2519	Sarcophilus harrisii
2520	Saxeibacter lacteus
2521	Scardovia inopinata
2522	Scardovia wiggsiae
2523	Schizosaccharomyces pombe
2524	Schwartzia succinivorans
2525	Sciscionella marina
2526	Sciscionella sp.
2527	Scytonema hofmanni
2528	Sebaldella termitidis
2529	Seculamonas ecuadoriensis
2530	Sedimenticola selenatireducens
2531	Sediminibacillus halophilus
2532	Sediminibacter sp.
2533	Sediminibacterium salmoneum
2534	Sediminibacterium sp.
2535	Segetibacter koreensis
2536	Segniliparus rugosus
2537	Selaginella moellendorffii
2538	Selenomonas bovis
2539	Selenomonas dianae
2540	Selenomonas infelix
2541	Selenomonas ruminantium
2542	Selenomonas sp.
2543	Serinicoccus marinus
2544	Serinicoccus profundi
2545	Serinus canaria
2546	Serratia fonticola

2547	Serratia liquefaciens
2548	Serratia marcescens
2549	Serratia odorifera
2550	Serratia plymuthica
2551	Serratia proteamaculans
2552	Serratia sp.
2553	Serratia symbiotica
2554	Setaria italica
2555	Shewanella baltica
2556	Shewanella colwelliana
2557	Shewanella denitrificans
2558	Shewanella fidelis
2559	Shewanella frigidimarina
2560	Shewanella loihica
2561	Shewanella marina
2562	Shewanella oneidensis
2563	Shewanella pealeana
2564	Shewanella piezotolerans
2565	Shewanella putrefaciens
2566	Shewanella sediminis
2567	Shewanella sp.
2568	Shigella sonnei
2569	Shimazuella kribbensis
2570	Shinella sp.
2571	Shuttleworthia satelles
2572	Sideroxydans lithotrophicus
2573	Silanimonas lenta
2574	Silicibacter lacuscaerulensis
2575	Simonsiella muelleri
2576	Simplicispira psychrophila
2577	Singularimonas variicoloris
2578	Singulisphaera acidiphila
2579	Sinomonas echigonensis
2580	Sinorhizobium arboris
2581	Sinorhizobium fredii
2582	Sinorhizobium meliloti
2583	Slackia heliotrinireducens
2584	Slackia piriformis
2585	Smaragdicoccus niigatensis
2586	Sneathia sanguinegens
2587	Sneathiella glossodoripedis
2588	Snodgrassella alvi
2589	Sodalis glossinidius
2590	Solanum lycopersicum
2591	Solanum tuberosum
2592	Solibacillus silvestris
2593	Solimonas flava
2594	Solimonas soli
2595	Solirubrobacter soli
2596	Solirubrobacter sp.
2597	Solirubrobacterales bacterium
2598	Solitalea canadensis
2599	Soonwooa buanensis
2600	Sorangium cellulosum
2601	Sordaria macrospora
2602	Sorex araneus
2603	Sorghum bicolor
2604	Spermophilus tridecemlineatus
2605	Sphaerobacter thermophilus
2606	Sphaerochaeta pleomorpha
2607	Sphingobacterium paucimobilis
2608	Sphingobacterium sp.
2609	Sphingobacterium spiritivorum
2610	Sphingobacterium thalpophilum
2611	Sphingobium baderi
2612	Sphingobium chinhatense
2613	Sphingobium chlorophenolicum
2614	Sphingobium japonicum
2615	Sphingobium lactosutens
2616	Sphingobium quisquiliarum
2617	Sphingobium sp.
2618	Sphingobium yanoikuyae
2619	Sphingomonas echinoides
2620	Sphingomonas phyllosphaerae
2621	Sphingomonas sp.
2622	Sphingomonas wittichii
2623	Sphingomonas-like bacterium
2624	Sphingopyxis alaskensis
2625	Spiribacter salinus
2626	Spirillospora albida
2627	Spirochaeta africana
2628	Spirochaeta bajacaliforniensis
2629	Spirochaeta caldaria
2630	Spirochaeta cellobiosiphila
2631	Spirochaeta smaragdinae
2632	Spirochaeta sp.
2633	Spirochaeta thermophila
2634	Spiroplasma apis
2635	Spiroplasma diminutum
2636	Spiroplasma syrphidicola
2637	Spiroplasma taiwanense
2638	Spirosoma linguale
2639	Spirosoma luteum
2640	Spirosoma panaciterrae
2641	Spirosoma spitsbergense
2642	Spongiibacter tropicus
2643	Sporichthya polymorpha
2644	Sporocytophaga myxococcoides
2645	Sporolactobacillus laevolacticus
2646	Sporolactobacillus vineae
2647	Sporomusa ovata
2648	Sporosarcina newyorkensis
2649	Sporosarcina sp.
2650	Stackebrandtia nassauensis
2651	Stanieria cyanosphaera
2652	Staphylococcus aureus
2653	Staphylococcus capitis
2654	Staphylococcus epidermidis
2655	Staphylococcus equorum
2656	Staphylococcus hominis
2657	Staphylococcus intermedius
2658	Staphylococcus lentus
2659	Staphylococcus lugdunensis
2660	Staphylococcus pasteuri
2661	Staphylococcus saprophyticus
2662	Staphylococcus simulans

2663	Staphylococcus sp.
2664	Staphylococcus vitulinus
2665	Staphylococcus warneri
2666	Staphylococcus xylosus
2667	Staphylothermus marinus
2668	Stappia stellulata
2669	Starkeya novella
2670	Stegastes partitus
2671	Stenotrophomonas maltophilia
2672	Stenotrophomonas sp.
2673	Stenoxybacter acetivorans
2674	Stigmatella aurantiaca
2675	Streptacidiphilus jeojiense
2676	Streptobacillus moniliformis
2677	Streptococcus agalactiae
2678	Streptococcus anginosus
2679	Streptococcus caballi
2680	Streptococcus castoreus
2681	Streptococcus constellatus
2682	Streptococcus devriesei
2683	Streptococcus didelphis
2684	Streptococcus dysgalactiae
2685	Streptococcus entericus
2686	Streptococcus equi
2687	Streptococcus equinus
2688	Streptococcus ferus
2689	Streptococcus gallolyticus
2690	Streptococcus henryi
2691	Streptococcus hyovaginalis
2692	Streptococcus infantarius
2693	Streptococcus iniae
2694	Streptococcus intermedius
2695	Streptococcus lutetiensis
2696	Streptococcus macedonicus
2697	Streptococcus marimammalium
2698	Streptococcus massiliensis
2699	Streptococcus merionis
2700	Streptococcus minor
2701	Streptococcus mutans
2702	Streptococcus oligofermentans
2703	Streptococcus orisratti
2704	Streptococcus ovis
2705	Streptococcus parauberis
2706	Streptococcus pasteurianus
2707	Streptococcus phage
2708	Streptococcus plurextorum
2709	Streptococcus pneumoniae
2710	Streptococcus porci
2711	Streptococcus prophage
2712	Streptococcus pseudopneumoniae
2713	Streptococcus pyogenes
2714	Streptococcus sobrinus
2715	Streptococcus sp.
2716	Streptococcus suis
2717	Streptococcus thermophilus
2718	Streptococcus thoraltensis
2719	Streptococcus tigurinus
2720	Streptococcus uberis

2721	Streptococcus vestibularis
2722	Streptomyces achromogenes
2723	Streptomyces acidiscabies
2724	Streptomyces afghaniensis
2725	Streptomyces albidoflavus
2726	Streptomyces albus
2727	Streptomyces auratus
2728	Streptomyces aureocirculatus
2729	Streptomyces aureofaciens
2730	Streptomyces avellaneus
2731	Streptomyces avermitilis
2732	Streptomyces baarnensis
2733	Streptomyces bicolor
2734	Streptomyces bikiniensis
2735	Streptomyces bingchenggensis
2736	Streptomyces bottropensis
2737	Streptomyces canus
2738	Streptomyces catenulae
2739	Streptomyces cattleya
2740	Streptomyces cellulosae
2741	Streptomyces chartreusis
2742	Streptomyces clavuligerus
2743	Streptomyces collinus
2744	Streptomyces cyaneofuscatus
2745	Streptomyces davawensis
2746	Streptomyces decoyicus
2747	Streptomyces durhamensis
2748	Streptomyces erythrochromogenes
2749	Streptomyces exfoliatus
2750	Streptomyces flavidovirens
2751	Streptomyces flavochromogenes
2752	Streptomyces flavotricini
2753	Streptomyces flavovariabilis
2754	Streptomyces flavovirens
2755	Streptomyces fulvissimus
2756	Streptomyces fulvoviolaceus
2757	Streptomyces globisporus
2758	Streptomyces griseus
2759	Streptomyces halstedii
2760	Streptomyces hygroscopicus
2761	Streptomyces lavendulae
2762	Streptomyces lavenduligriseus
2763	Streptomyces leeuwenhoekii
2764	Streptomyces lividans
2765	Streptomyces megasporus
2766	Streptomyces monomycini
2767	Streptomyces natalensis
2768	Streptomyces niveus
2769	Streptomyces ochraceiscleroticus
2770	Streptomyces olivaceus
2771	Streptomyces peruviensis
2772	Streptomyces peucetius
2773	Streptomyces pristinaespiralis
2774	Streptomyces prunicolor
2775	Streptomyces purpeofuscus
2776	Streptomyces purpureus
2777	Streptomyces pyridomyceticus
2778	Streptomyces rapamycinicus

2779	Streptomyces resistomycificus
2780	Streptomyces rimosus
2781	Streptomyces roseochromogenus
2782	Streptomyces roseosporus
2783	Streptomyces roseoverticillatus
2784	Streptomyces ruber
2785	Streptomyces scabiei
2786	Streptomyces scabrisporus
2787	Streptomyces sclerotialus
2788	Streptomyces seoulensis
2789	Streptomyces somaliensis
2790	Streptomyces sp.
2791	Streptomyces sulphureus
2792	Streptomyces sviceus
2793	Streptomyces thermolilacinus
2794	Streptomyces varsoviensis
2795	Streptomyces venezuelae
2796	Streptomyces violaceoruber
2797	Streptomyces violaceorubidus
2798	Streptomyces violaceusniger
2799	Streptomyces violens
2800	Streptomyces virginiae
2801	Streptomyces viridochromogenes
2802	Streptomyces viridosporus
2803	Streptomyces vitaminophilus
2804	Streptomyces xanthophaeus
2805	Streptomycetaceae bacterium
2806	Streptosporangium amethystogenes
2807	Streptosporangium roseum
2808	Strongylocentrotus purpuratus
2809	Subdoligranulum sp.
2810	Subdoligranulum variabile
2811	Succinatimonas sp.
2812	Succinispira mobilis
2813	Succinivibrio dextrinosolvens
2814	Succinivibrionaceae bacterium
2815	Sulfitobacter guttiformis
2816	Sulfitobacter mediterraneus
2817	Sulfitobacter sp.
2818	Sulfolobus islandicus
2819	Sulfuricella denitrificans
2820	Sulfuricurvum kujiense
2821	Sulfurimonas autotrophica
2822	Sulfurimonas denitrificans
2823	Sulfurimonas sp.
2824	Sulfurospirillum arcachonense
2825	Sulfurospirillum barnesii
2826	Sulfurospirillum deleyianum
2827	Sulfurovum sp.
2828	Sus scrofa
2829	Sutterella parvirubra
2830	Sutterella sp.
2831	Sutterella wadsworthensis
2832	Symbiobacterium thermophilum
2833	Syncytium symbiont
2834	Synechococcus phage
2835	Synechococcus sp.
2836	Synechocystis sp.

2837	Synergistetes bacterium
2838	Syntrophobacter fumaroxidans
2839	Syntrophobotulus glycolicus
2840	Syntrophorhabdus aromaticivorans
2841	Syntrophothermus lipocalidus
2842	Syntrophus aciditrophicus
2843	Taeniopygia guttata
2844	Takifugu rubripes
2845	Tannerella forsythia
2846	Tannerella sp.
2847	Tarsius syrichta
2848	Tatumella ptyseos
2849	Tatumella sp.
2850	Taylorella equigenitalis
2851	Tenacibaculum maritimum
2852	Tenacibaculum ovolyticum
2853	Tenacibaculum sp.
2854	Tepidanaerobacter acetatoxydans
2855	Tepidiphilus margaritifer
2856	Terasakiella pusilla
2857	Teredinibacter turnerae
2858	Terracoccus sp.
2859	Terriglobus saanensis
2860	Terrimonas ferruginea
2861	Terrisporobacter glycolicus
2862	Tetragenococcus halophilus
2863	Tetrahymena thermophila
2864	Tetrasphaera elongata
2865	Thalassiobium sp.
2866	Thalassobacillus devorans
2867	Thalassobacter arenae
2868	Thalassospira lucentensis
2869	Thauera linaloolentis
2870	Thauera sp.
2871	Thauera terpenica
2872	Theileria annulata
2873	Theobroma cacao
2874	Thermaerobacter marianensis
2875	Thermaerobacter subterraneus
2876	Thermanaerovibrio acidaminovorans
2877	Thermicanus aegyptius
2878	Thermincola potens
2879	Thermithiobacillus tepidarius
2880	Thermoanaerobacter brockii
2881	Thermoanaerobacter indiensis
2882	Thermoanaerobacter italicus
2883	Thermoanaerobacter mathranii
2884	Thermoanaerobacter siderophilus
2885	Thermoanaerobacter sp.
2886	Thermoanaerobacter tengcongensis
2887	Thermoanaerobacter wiegelii
2888	Thermoanaerobacterium saccharolyticum
2889	Thermoanaerobacterium thermosaccharolyticum
2890	Thermoanaerobacterium xylanolyticum
2891	Thermobacillus composti
2892	Thermobaculum terrenum
2893	Thermobifida fusca

2894	Thermobrachium celere
2895	Thermococcus barophilus
2896	Thermococcus zilligii
2897	Thermocrispum agreste
2898	Thermocrispum municipale
2899	Thermodesulfatator atlanticus
2900	Thermodesulfatator indicus
2901	Thermodesulfobacterium hveragerdense
2902	Thermodesulfobium narugense
2903	Thermodesulfovibrio thiophilus
2904	Thermodesulfovibrio yellowstonii
2905	Thermomicrobium roseum
2906	Thermomonas fusca
2907	Thermomonospora curvata
2908	Thermophagus xiamenensis
2909	Thermoplasmatales archaeon
2910	Thermosediminibacter oceani
2911	Thermosipho africanus
2912	Thermosipho melanesiensis
2913	Thermosynechococcus sp.
2914	Thermotoga elfii
2915	Thermotoga maritima
2916	Thermotoga thermarum
2917	Thermovibrio ammonificans
2918	Thermus igniterrae
2919	Thermus islandicus
2920	Thermus oshimai
2921	Thermus sp.
2922	Thermus thermophilus
2923	Thioalkalivibrio nitratireducens
2924	Thioalkalivibrio sp.
2925	Thioalkalivibrio sulfidophilus
2926	Thioalkalivibrio thiocyanodenitrificans
2927	Thioalkalivibrio thiocyanoxidans
2928	Thiobacillus denitrificans
2929	Thiobacillus thioparus
2930	Thiocystis violascens
2931	Thioflavicoccus mobilis
2932	Thiomicrospira arctica
2933	Thiomicrospira chilensis
2934	Thiomicrospira crunogena
2935	Thiomicrospira halophila
2936	Thiomicrospira pelophila
2937	Thiomicrospira sp.
2938	Thiomonas intermedia
2939	Thiomonas sp.
2940	Thioreductor micantisoli
2941	Thiorhodovibrio sp.
2942	Thiothrix disciformis
2943	Thiothrix flexilis
2944	Thiothrix lacustris
2945	Thiothrix nivea
2946	Tistrella mobilis
2947	Togninia minima
2948	Tolumonas sp.
2949	Tomitella biformata
2950	Toxoplasma gondii
2951	Tremella mesenterica

2952	Treponema brennaborense
2953	Treponema bryantii
2954	Treponema lecithinolyticum
2955	Treponema maltophilum
2956	Treponema medium
2957	Treponema pallidum
2958	Treponema pedis
2959	Treponema phagedenis
2960	Treponema primitia
2961	Treponema socranskii
2962	Treponema sp.
2963	Treponema succinifaciens
2964	Tribolium castaneum
2965	Trichechus manatus
2966	Trichinella spiralis
2967	Trichodesmium erythraeum
2968	Trichomonas vaginalis
2969	Trichoplax adhaerens
2970	Truepera radiovictrix
2971	Trueperella pyogenes
2972	Trypanosoma cruzi
2973	Tsukamurella paurometabola
2974	Tsukubamonas globosa
2975	Tuber melanosporum
2976	Tupaia chinensis
2977	Turicella otitidis
2978	Tursiops truncatus
2979	Uliginosibacterium gangwonense
2980	Uncultured bacterium
2981	Uncultured Sulfuricurvum
2982	Undibacterium oligocarboniphilum
2983	Ureaplasma canigenitalium
2984	Ureaplasma parvum
2985	Ureibacillus thermosphaericus
2986	Ursus maritimus
2987	Ustilago maydis
2988	Vaccinium witches'-broom
2989	Vanderwaltozyma polyspora
2990	Variovorax paradoxus
2991	Variovorax sp.
2992	Verminephrobacter aporrectodeae
2993	Verminephrobacter eiseniae
2994	Verrucomicrobia bacterium
2995	Verrucomicrobiae bacterium
2996	Verrucomicrobium sp.
2997	Verrucomicrobium spinosum
2998	Verrucosispora maris
2999	Verticillium albo-atrum
3000	Vibrio angustum
3001	Vibrio campbellii
3002	Vibrio cholerae
3003	Vibrio coralliilyticus
3004	Vibrio crassostreae
3005	Vibrio cyclitrophicus
3006	Vibrio fischeri
3007	Vibrio furnissii
3008	Vibrio genomosp.
3009	Vibrio harveyi

3010	Vibrio kanaloae
3011	Vibrio litoralis
3012	Vibrio mimicus
3013	Vibrio nigripulchritudo
3014	Vibrio ordalii
3015	Vibrio parahaemolyticus
3016	Vibrio rotiferianus
3017	Vibrio rumoiensis
3018	Vibrio shiloi
3019	Vibrio sp.
3020	Vibrio splendidus
3021	Vibrio vulnificus
3022	Vicugna pacos
3023	Virgibacillus halodenitrificans
3024	Vitis vinifera
3025	Vitreoscilla stercoraria
3026	Volvox carteri
3027	Waddlia chondrophila
3028	Wallemia sebi
3029	Weeksella virosa
3030	Weissella cibaria
3031	Weissella koreensis
3032	Weissella oryzae
3033	Weissella paramesenteroides
3034	Wigglesworthia glossinidia
3035	Wohlfahrtiimonas chitiniclastica
3036	Wolbachia sp.
3037	Wolinella succinogenes
3038	Xanthobacter autotrophicus
3039	Xanthobacter sp.
3040	Xanthobacteraceae bacterium
3041	Xanthomonadaceae bacterium
3042	Xanthomonas albilineans
3043	Xanthomonas arboricola
3044	Xanthomonas axonopodis
3045	Xanthomonas campestris
3046	Xanthomonas fuscans
3047	Xanthomonas hortorum
3048	Xanthomonas oryzae
3049	Xanthomonas phage
3050	Xanthomonas sacchari
3051	Xanthomonas sp.
3052	Xanthomonas translucens
3053	Xenopus (Silurana)
3054	Xenorhabdus bovienii
3055	Xenorhabdus nematophila
3056	Xiphophorus maculatus
3057	Xylanimonas cellulosilytica
3058	Xylella fastidiosa
3059	Yaniella halotolerans
3060	Yersinia enterocolitica
3061	Yersinia pestis
3062	Yersinia pseudotuberculosis
3063	Yokenella regensburgei
3064	Zavarzinella formosa
3065	Zea mays
3066	Zeta proteobacterium
3067	Zimmermannella faecalis
3068	Zobellia galactanivorans
3069	Zonotrichia albicollis
3070	Zoogloea oryzae
3071	Zooshikella ganghwensis
3072	Zunongwangia profunda
3073	Zygosaccharomyces rouxii
3074	Zymomonas mobilis
3075	Zymophilus raffinosivorans

Table S31 : The taxonomic profile predicted from the assembly results of MetaVelvet-SL using BLAST for the SRS018661 dataset.

No.	Species
1	Abiotrophia defectiva
2	Acidovorax unclassified
3	Acinetobacter junii
4	Actinomyces odontolyticus
5	Actinomyces oris
6	Actinomyces viscosus
7	Aggregatibacter aphrophilus
8	Aggregatibacter segnis
9	Alicycliphilus denitrificans
10	Anaerococcus unclassified
11	Atopobium parvulum
12	Campylobacter concisus
13	Campylobacter gracilis
14	Capnocytophaga gingivalis
15	Capnocytophaga ochracea
16	Capnocytophaga sputigena
17	Cardiobacterium hominis
18	Catonella morbi
19	Corynebacterium matruchotii
20	Dialister invisus
21	Eikenella corrodens
22	Escherichia coli
23	Escherichia unclassified
24	Eubacterium saburreum
25	Eubacterium saphenum
26	Fusobacterium nucleatum
27	Fusobacterium periodonticum
28	Gemella haemolysans
29	Gemella moribillum
30	Granulicatella adiacens
31	Granulicatella elegans
32	Haemophilus influenzae
33	Haemophilus parainfluenzae
34	Kingella oralis
35	Lautropia mirabilis
36	Leptotrichia buccalis
37	Leptotrichia hofstadii
38	Leptotrichia unclassified
39	Megasphaera micronuciformis
40	Neisseria gonorrhoeae
41	Neisseria mucosa
42	Neisseria sicca
43	Oribacterium sinus
44	Parvimonas micros
45	Peptoniphilus unclassified
46	Peptostreptococcus stomatis
47	Prevotella melaninogenica
48	Prevotella multiformis
49	Prevotella oris
50	Prevotella salivae
51	Prevotella tannerae
52	Prevotella veroralis
53	Propionibacterium acnes
54	Propionibacterium unclassified
55	Rothia dentocariosa
56	Rothia mucilaginosa
57	Selenomonas artemidis
58	Selenomonas flueggei
59	Selenomonas noxia
60	Selenomonas sputigena
61	Selenomonas unclassified
62	Solobacterium moorei
63	Streptococcus anginosus
64	Streptococcus australis
65	Streptococcus cristatus
66	Streptococcus gordonii
67	Streptococcus infantis
68	Streptococcus mitis
69	Streptococcus oralis
70	Streptococcus parasanguinis
71	Streptococcus peroris
72	Streptococcus pneumoniae
73	Streptococcus salivarius
74	Streptococcus sanguinis
75	Streptococcus thermophilus
76	Treponema denticola
77	Treponema vincentii
78	Veillonella atypica
79	Veillonella dispar
80	Veillonella parvula
81	Veillonella unclassified
1	'Nostoc azollae'
2	[Clostridium] aerotolerans
3	[Clostridium] bifermentans
4	[Clostridium] mangenotii
5	[Clostridium] methoxybenzovorans
6	[Clostridium] papyrosolvens
7	[Clostridium] saccharogumia
8	[Clostridium] sordellii
9	[Clostridium] sticklandii,
10	[Eubacterium] cellulosolvens
11	[Eubacterium] yurii
12	[Scytonema hofmanni]
13	Acanthamoeba polyphaga
14	Acetivibrio cellulolyticus
15	Acetobacter aceti
16	Acetobacter sp.
17	Acetobacterium woodii
18	Acholeplasma axanthum
19	Acholeplasma granularum
20	Acholeplasma sp.
21	Achromobacter piechaudii
22	Achromobacter xylosoxidans
23	Acidaminococcus sp.
24	Acidiphilium angustum
25	Acidithiobacillus ferrivorans
26	Acidobacteria bacterium
27	Acidobacteriaceae bacterium
28	Acidocella sp.
29	Acinetobacter baumannii

30	Acinetobacter bereziniae
31	Acinetobacter brisouii
32	Acinetobacter calcoaceticus
33	Acinetobacter genomosp.
34	Acinetobacter gerneri
35	Acinetobacter guillouiae
36	Acinetobacter gyllenbergii
37	Acinetobacter haemolyticus
38	Acinetobacter indicus
39	Acinetobacter johnsonii
40	Acinetobacter lwoffii
41	Acinetobacter nosocomialis
42	Acinetobacter parvus
43	Acinetobacter pittii
44	Acinetobacter radioresistens
45	Acinetobacter schindleri
46	Acinetobacter soli
47	Acinetobacter sp.
48	Acinetobacter tandoii
49	Acinetobacter tjernbergiae
50	Acinetobacter ursingii
51	Acinetobacter venetianus
52	Actinobacillus capsulatus
53	Actinobacillus hominis
54	Actinobacillus pleuropneumoniae
55	Actinobacillus porcinus
56	Actinobacillus succinogenes
57	Actinobacillus suis
58	Actinobacillus ureae
59	Actinobacterium LLX17
60	Actinobacterium SCGC
61	Actinobaculum massiliae
62	Actinobaculum sp.
63	Actinobaculum urinale
64	Actinomadura flavalba
65	Actinomyces cardiffensis
66	Actinomyces dentalis
67	Actinomyces europaeus
68	Actinomyces georgiae
69	Actinomyces gerencseriae
70	Actinomyces graevenitzii
71	Actinomyces johnsonii
72	Actinomyces massiliensis
73	Actinomyces naeslundii
74	Actinomyces neuii
75	Actinomyces slackii
76	Actinomyces sp.
77	Actinomyces suimastitidis
78	Actinomyces timonensis
79	Actinomyces turicensis
80	Actinomyces urogenitalis
81	Actinomyces vaccimaxillae
82	Actinoplanes friuliensis
83	Actinoplanes missouriensis
84	Actinoplanes subtropicus
85	Actinopolymorpha alba
86	Acyrthosiphon pisum
87	Adhaeribacter aquaticus
88	Aedes aegypti
89	Aequorivita capsosiphonis
90	Aequorivita sublithincola
91	Aerococcus urinae
92	Aeromicrobium marinum
93	Aeromonas hydrophila
94	Aestuariimicrobium kwangyangense
95	Agaricus bisporus
96	Aggregatibacter actinomycetemcomitans
97	Aggregatibacter phage
98	Aggregatibacter sp.
99	Agrococcus pavilionensis
100	Agromyces italicus
101	Ailuropoda melanoleuca
102	Alcaligenes faecalis
103	Algicola sagamiensis
104	Algoriphagus mannitolivorans
105	Algoriphagus marincola
106	Algoriphagus sp.
107	Algoriphagus terrigena
108	Algoriphagus vanfongensis
109	Aliagarivorans marinus
110	Alicyclobacillus acidoterrestris
111	Aliivibrio salmonicida
112	Alistipes finegoldii
113	Alistipes indistinctus
114	Alistipes onderdonkii
115	Alistipes putredinis
116	Alistipes senegalensis
117	Alistipes shahii
118	Alistipes sp.
119	Alistipes timonensis
120	Alkalibacillus haloalkaliphilus
121	Alkaliflexus imshenetskii
122	Alkaliphilus oremlandii
123	Alkaliphilus transvaalensis
124	Alkanindiges illinoisensis
125	Alligator mississippiensis
126	Allofustis seminis
127	Allokutzneria albata
128	Alloscardovia omnicolens
129	Alpha proteobacterium
130	alpha proteobacterium
131	Alteromonas sp.
132	Amborella trichopoda
133	Aminomonas paucivorans
134	Amphibacillus jilinensis
135	Amphimedon queenslandica
136	Amycolatopsis balhimycina
137	Amycolatopsis nigrescens
138	Amycolatopsis orientalis
139	Anabaena cylindrica
140	Anabaena sp.
141	Anabaena variabilis
142	Anaeroglobus geminatus
143	Anaeromusa acidaminophila
144	Anaerophaga thermohalophila
145	Anaerovibrio sp.

146	Anaerovorax odorimutans
147	Anas platyrhynchos
148	Aneurinibacillus aneurinilyticus
149	Anolis carolinensis
150	Anopheles gambiae
151	Aphanizomenon flos-aquae
152	Apis dorsata
153	Apis mellifera
154	Aplysia californica
155	Aptenodytes forsteri
156	Aquimarina agarilytica
157	Aquimarina latercula
158	Aquimarina macrocephali
159	Aquimarina megaterium
160	Aquimarina muelleri
161	Aquimarina sp.
162	Arcanobacterium haemolyticum
163	Arcobacter butzleri
164	Arcobacter cibarius
165	Arcobacter sp.
166	Arenibacter algicola
167	Arenibacter certesii
168	Arenibacter latericius
169	Arenimonas composti
170	Arenitalea lutea
171	Arsenophonus nasoniae
172	Arthrobacter sp.
173	Arthrospira platensis
174	Asaia platycodi
175	Asaia prunellae
176	Astyanax mexicanus
177	Atopobacter phocae
178	Atopobium sp.
179	Avibacterium paragallinarum
180	Azoarcus sp.
181	Azohydromonas australica
182	Azorhizobium doebereinerae
183	Azospirillum brasilense
184	Azospirillum sp.
185	Azovibrio restrictus
186	Bacillus acidiproducens
187	Bacillus aurantiacus
188	Bacillus bogoriensis
189	Bacillus cereus
190	Bacillus coagulans
191	Bacillus coahuilensis
192	Bacillus endophyticus
193	Bacillus flexus
194	Bacillus gelatini
195	Bacillus kribbensis
196	Bacillus mannanilyticus
197	Bacillus massilioanorexius
198	Bacillus massiliosenegalensis
199	Bacillus megaterium
200	Bacillus oceanisediminis
201	Bacillus pumilus
202	Bacillus sp.
203	Bacillus subtilis

204	Bacillus thuringiensis
205	Bacillus timonensis
206	Bacteriophage Aaphi23,
207	Bacteriovorax marinus
208	Bacteroidales bacterium
209	Bacteroides acidifaciens
210	Bacteroides barnesiae
211	Bacteroides cellulosilyticus
212	Bacteroides clarus
213	Bacteroides coprocola
214	Bacteroides coprophilus
215	Bacteroides dorei
216	Bacteroides eggerthii
217	Bacteroides faecichinchillae
218	Bacteroides faecis
219	Bacteroides finegoldii
220	Bacteroides fluxus
221	Bacteroides fragilis
222	Bacteroides gallinarum
223	Bacteroides graminisolvens
224	Bacteroides helcogenes
225	Bacteroides intestinalis
226	Bacteroides massiliensis
227	Bacteroides nordii
228	Bacteroides oleiciplenus
229	Bacteroides ovatus
230	Bacteroides paurosaccharolyticus
231	Bacteroides pectinophilus
232	Bacteroides plebeius
233	Bacteroides propionicifaciens
234	Bacteroides pyogenes
235	Bacteroides rodentium
236	Bacteroides salanitronis
237	Bacteroides salyersiae
238	Bacteroides sartorii
239	Bacteroides sp.
240	Bacteroides stercorirosoris
241	Bacteroides stercoris
242	Bacteroides uniformis
243	Bacteroides vulgatus
244	Bacteroides xylanisolvens
245	Bacteroidetes bacterium
246	Bacteroidetes oral
247	Balaenoptera acutorostrata
248	Barnesiella intestinihominis
249	Bartonella doshiae
250	Bartonella elizabethae
251	Bartonella henselae
252	Bartonella vinsonii
253	Bavariicoccus seileri
254	Bdellovibrio bacteriovorus
255	Belliella baltica
256	Bergeyella zoohelcum
257	Beutenbergia cavernae
258	Bibersteinia trehalosi
259	Bifidobacterium animalis
260	Bifidobacterium bifidum
261	Bifidobacterium breve

262	Bifidobacterium choerinum
263	Bifidobacterium longum
264	Bifidobacterium magnum
265	Bifidobacterium pseudolongum
266	Bifidobacterium ruminantium
267	Bifidobacterium sp.
268	Bifidobacterium thermophilum
269	Bilophila wadsworthia
270	Bisgaard taxon
271	Blastococcus saxobsidens
272	Blastococcus sp.
273	Blattabacterium sp.
274	Blautia hansenii
275	Blautia hydrogenotrophica
276	Blautia sp.
277	Bombyx mori
278	Bordetella bronchiseptica
279	Bordetella hinzii
280	Bordetella petrii
281	Borrelia crocidurae
282	Bos mutus
283	Bos taurus
284	Botryotinia fuckeliana
285	Brachybacterium faecium
286	Brachybacterium muris
287	Brachybacterium paraconglomeratum
288	Brachybacterium squillarum
289	Brachymonas chironomi
290	Brachypodium distachyon
291	Brachyspira innocens
292	Brachyspira sp.
293	Bradyrhizobium japonicum
294	Bradyrhizobium sp.
295	Branchiostoma floridae
296	Brassica rapa
297	Brevibacterium album
298	Brevibacterium linens
299	Brevibacterium senegalense,
300	Brevundimonas aveniformis
301	Brevundimonas diminuta
302	Brochothrix thermosphacta
303	Brucella sp.
304	Brugia malayi
305	Bubalus bubalis
306	Burkholderia andropogonis
307	Burkholderia cenocepacia
308	Burkholderia cepacia
309	Burkholderia pseudomallei
310	Burkholderia pyrrocinia
311	Burkholderia sordidicola
312	Burkholderia sp.
313	Burkholderia vietnamiensis
314	Butyricimonas synergistica
315	Butyrivibrio crossotus
316	Butyrivibrio fibrisolvens
317	Butyrivibrio proteoclasticus
318	Butyrivibrio sp.
319	Caenorhabditis elegans

320	Caldibacillus debilis
321	Caldicellulosiruptor obsidiansis
322	Caldicellulosiruptor owensensis
323	Caldimonas manganoxidans
324	Caldisericum exile
325	Callithrix jacchus
326	Calothrix sp.
327	Calypte anna
328	Camelus ferus
329	Campylobacter coli
330	Campylobacter curvus
331	Campylobacter hominis
332	Campylobacter jejuni
333	Campylobacter sp.
334	Campylobacter upsaliensis
335	Campylobacter ureolyticus
336	Campylobacterales bacterium
337	Candida glabrata
338	Candidate division
339	Candidatus Alistipes
340	Candidatus Arthromitus
341	Candidatus Cloacamonas
342	Candidatus Kinetoplastibacterium
343	Candidatus Liberibacter
344	Candidatus Odyssella
345	Candidatus Pelagibacter
346	Candidatus Poribacteria
347	Candidatus Puniceispirillum
348	Candidatus Ruthia
349	Candidatus Saccharibacteria
350	Candidatus Saccharimonas
351	Candidatus Schmidhempelia
352	Candidatus Stoquefichus
353	Canis familiaris
354	Canis lupus
355	Capnocytophaga canimorsus
356	Capnocytophaga cynodegmi
357	Capnocytophaga granulosa
358	Capnocytophaga sp.
359	Capra hircus
360	Cardiobacterium valvarum
361	Carnobacterium jeotgali
362	Carnobacterium maltaromaticum
363	Carnobacterium sp.
364	Caulobacter segnis
365	Caulobacter sp.
366	Cavia porcellus
367	Cellulomonas fimi
368	Cellulomonas flavigena
369	Cellulomonas sp.
370	Cellulophaga algicola
371	Cellulophaga baltica
372	Cellulophaga lytica
373	Cellulosimicrobium cellulans
374	Cellvibrio gilvus
375	Centipeda periodontii
376	Ceratitis capitata
377	Ceratotherium simum

378	Cetobacterium somerae
379	Chelativorans sp.
380	Chelonia mydas
381	Chinchilla lanigera
382	Chitinimonas koreensis
383	Chlamydomonas reinhardtii
384	Chlamydophila sp.
385	Chlorobium chlorochromatii
386	Chlorobium luteolum
387	Chlorocebus aethiops
388	Chlorocebus sabaeus
389	Chloroflexi bacterium
390	Chloroherpeton thalassium
391	Chroococcidiopsis thermalis
392	Chrysemys picta
393	Chryseobacterium caeni
394	Chryseobacterium daeguense
395	Chryseobacterium gleum
396	Chryseobacterium gregarium
397	Chryseobacterium haifense
398	Chryseobacterium hispalense
399	Chryseobacterium palustre
400	Chryseobacterium sp.
401	Chryseobacterium taeanense
402	Chrysochloris asiatica
403	Cicer arietinum
404	Ciona intestinalis
405	Citricoccus sp.
406	Citrobacter rodentium
407	Citrobacter youngae
408	Citrus clementina
409	Citrus sinensis
410	Clavibacter michiganensis
411	Cloacibacterium rupense
412	Cloacimonetes bacterium
413	Clostridiales bacterium
414	Clostridiales genomosp.
415	Clostridiisalibacter paucivorans
416	Clostridium acetobutylicum
417	Clostridium autoethanogenum
418	Clostridium bartlettii
419	Clostridium beijerinckii
420	Clostridium bolteae
421	Clostridium botulinum
422	Clostridium butyricum
423	Clostridium cadaveris
424	Clostridium celatum
425	Clostridium cellulolyticum
426	Clostridium cellulovorans
427	Clostridium cf.
428	Clostridium clariflavum
429	Clostridium clostridioforme
430	Clostridium colicanis
431	Clostridium difficile
432	Clostridium hathewayi
433	Clostridium hiranonis
434	Clostridium hydrogeniformans
435	Clostridium innocuum

436	Clostridium intestinale
437	Clostridium kluyveri
438	Clostridium lentocellum
439	Clostridium ljungdahlii
440	Clostridium lundense
441	Clostridium nexile
442	Clostridium paraputrificum
443	Clostridium pasteurianum
444	Clostridium perfringens
445	Clostridium phytofermentans
446	Clostridium saccharobutylicum
447	Clostridium saccharolyticum
448	Clostridium saccharoperbutylacetonicum
449	Clostridium scatologenes
450	Clostridium scindens
451	Clostridium senegalense
452	Clostridium sp.
453	Clostridium spiroforme
454	Clostridium sporogenes
455	Clostridium tetani
456	Clostridium thermocellum
457	Clostridium tyrobutyricum
458	Clostridium ultunense
459	Clostridium viride
460	Cobetia crustatorum
461	Collinsella sp.
462	Columba livia
463	Comamonadaceae bacterium
464	Comamonas badia
465	Comamonas sp.
466	Comamonas testosteroni
467	complete chromosome
468	Conchiformibius kuhniae
469	Conchiformibius steedae
470	Condylura cristata
471	Congregibacter litoralis
472	Coprobacillus sp.
473	Coprococcus catus
474	Coprococcus comes
475	Coprococcus eutactus
476	Coprococcus sp.
477	Coraliomargarita akajimensis
478	Corallococcus coralloides
479	Cordyceps militaris
480	Coriobacteriaceae bacterium
481	Corvus brachyrhynchos
482	Corynebacterium aurimucosum
483	Corynebacterium bovis
484	Corynebacterium ciconiae
485	Corynebacterium diphtheriae
486	Corynebacterium durum
487	Corynebacterium efficiens
488	Corynebacterium genitalium
489	Corynebacterium glucuronolyticum
490	Corynebacterium jeikeium
491	Corynebacterium lipophiloflavum
492	Corynebacterium lubricantis
493	Corynebacterium massiliense

494	Corynebacterium nuruki
495	Corynebacterium sp.
496	Corynebacterium sputi
497	Corynebacterium striatum
498	Corynebacterium ulcerans
499	Corynebacterium variabile
500	Corynebacterium-like bacterium
501	Crenarchaeota archaeon
502	Cricetulus griseus
503	Croceibacter atlanticus
504	Crocinitomix catalasitica
505	Cronobacter helveticus
506	Cronobacter sakazakii
507	Cucumibacter marinus
508	Cucumis melo
509	Cucumis sativus
510	Culex pipiens
511	Cupriavidus sp.
512	Curtobacterium flaccumfaciens
513	Curtobacterium sp.
514	Curvibacter lanceolatus
515	Cyanothece sp.
516	Cyclobacterium marinum
517	Cynoglossus semilaevis
518	Cytophaga fermentans
519	Cytophaga hutchinsonii
520	Danio rerio
521	Dasypus novemcinctus
522	Dechlorosoma suillum
523	Dehalobacter sp.
524	Dehalogenimonas lykanthroporepellens
525	Deinococcus aquatilis
526	Deinococcus geothermalis
527	Deinococcus gobiensis
528	Deinococcus peraridilitoris
529	Deinococcus wulumuqiensis
530	Delftia acidovorans
531	Delftia sp.
532	delta proteobacterium
533	Delta proteobacterium
534	Dermabacter sp.
535	Desulfitobacterium dichloroeliminans
536	Desulfobacterium anilini
537	Desulfobulbus propionicus
538	Desulfococcus oleovorans
539	Desulfosporosinus orientis
540	Desulfosporosinus youngiae
541	Desulfotomaculum acetoxidans
542	Desulfotomaculum alcoholivorax
543	Desulfotomaculum gibsoniae
544	Desulfovibrio aespoeensis
545	Desulfovibrio desulfuricans
546	Desulfovibrio gigas
547	Desulfovibrio putealis
548	Desulfurispirillum indicum
549	Desulfurivibrio alkaliphilus
550	Desulfurobacterium thermolithotrophum
551	Desulfuromonas sp.
552	Dialister micraerophilus
553	Dialister sp.
554	Diaphorina citri
555	Diaphorobacter nitroreducens
556	Dickeya dadantii
557	Dickeya paradisiaca
558	Dickeya zeae
559	Dictyostelium discoideum
560	Dietzia alimentaria
561	Dinoroseobacter shibae
562	Diplorickettsia massiliensis
563	Dolosigranulum pigrum
564	Donghicola xiamenensis
565	Dorea longicatena
566	Dorea sp.
567	Drosophila ananassae
568	Drosophila melanogaster
569	Drosophila mojavensis
570	Drosophila willistoni
571	Drosophila yakuba
572	Dyadobacter alkalitolerans
573	Dyadobacter beijingensis
574	Dyadobacter fermentans
575	Dyella ginsengisoli
576	Dysgonomonas capnocytophagoides
577	Dysgonomonas gadei
578	Echinops telfairi
579	Edwardsiella ictaluri
580	Edwardsiella tarda
581	Eggerthella sp.
582	Eggerthia catenaformis
583	Elephantulus edwardii
584	Elioraea tepidiphila
585	Elizabethkingia anophelis
586	Elizabethkingia meningoseptica
587	Emiliania huxleyi
588	Empedobacter brevis
589	Emticicia oligotrophica
590	Entamoeba histolytica
591	Entamoeba invadens
592	Entamoeba nuttalli
593	Enterobacter asburiae
594	Enterobacter cloacae
595	Enterobacter radicincitans
596	Enterobacter sp.
597	Enterobacteria phage
598	Enterococcus avium
599	Enterococcus caccae
600	Enterococcus casseliflavus
601	Enterococcus columbae
602	Enterococcus faecalis
603	Enterococcus faecium
604	Enterococcus haemoperoxidus
605	Enterococcus italicus
606	Enterococcus mundtii
607	Enterococcus pallens
608	Enterococcus phoeniculicola
609	Enterococcus saccharolyticus

610	Enterococcus sulfureus
611	Enterococcus villorum
612	Enterocytozoon bieneusi
613	Enterovibrio calviensis
614	Epilithonimonas tenax
615	Eptesicus fuscus
616	Equus caballus
617	Equus przewalskii
618	Eremococcus coleocola
619	Erinaceus europaeus
620	Erwinia sp.
621	Erwinia toletana
622	Erysipelotrichaceae bacterium
623	Ethanoligenens harbinense
624	Eubacteriaceae bacterium
625	Eubacterium biforme
626	Eubacterium brachy
627	Eubacterium cylindroides
628	Eubacterium desmolans
629	Eubacterium eligens
630	Eubacterium hallii
631	Eubacterium infirmum
632	Eubacterium rectale
633	Eubacterium siraeum
634	Eubacterium sp.
635	Eubacterium ventriosum
636	Eubacterium xylanophilum
637	Eubacterium yurii
638	Eutrema salsugineum
639	Eutypa lata
640	Facklamia hominis
641	Facklamia ignava
642	Faecalibacterium prausnitzii
643	Faecalibacterium sp.
644	Falco cherrug
645	Felis catus
646	Ferrovum myxofaciens
647	Fibrella aestuarina
648	Fibrobacter succinogenes
649	Ficedula albicollis
650	Filamentous cyanobacterium
651	Filifactor alocis
652	Finegoldia magna
653	Firmicutes bacterium
654	Fischerella sp.
655	Flavobacteria bacterium
656	Flavobacteriaceae bacterium
657	Flavobacterium antarcticum
658	Flavobacterium branchiophilum
659	Flavobacterium chungangense
660	Flavobacterium columnare
661	Flavobacterium daejeonense
662	Flavobacterium denitrificans
663	Flavobacterium filum
664	Flavobacterium frigidarium
665	Flavobacterium gelidilacus
666	Flavobacterium indicum
667	Flavobacterium johnsoniae

668	Flavobacterium psychrophilum
669	Flavobacterium sasangense
670	Flavobacterium soli
671	Flavobacterium sp.
672	Flavobacterium succinicans
673	Flavobacterium suncheonense
674	Flavobacterium tegetincola
675	Flectobacillus major
676	Flexibacter elegans
677	Flexibacter litoralis
678	Flexibacter roseolus
679	Fluviicola taffensis
680	Fomitiporia mediterranea
681	Fragaria vesca
682	Francisella philomiragia
683	Francisella sp.
684	Francisella tularensis
685	Frankia sp.
686	Frankia symbiont
687	Fulvimarina pelagi
688	Fusobacterium gonidiaformans
689	Fusobacterium mortiferum
690	Fusobacterium necrophorum
691	Fusobacterium perfoetens
692	Fusobacterium russii
693	Fusobacterium sp.
694	Fusobacterium ulcerans
695	Fusobacterium varium
696	Gaetbulibacter saemankumensis
697	Gaeumannomyces graminis
698	Galeopterus variegatus
699	Gallibacterium anatis
700	Gallus gallus
701	Gamma proteobacterium
702	gamma proteobacterium
703	Gardnerella vaginalis
704	Geitlerinema sp.
705	Gelidibacter mesophilus
706	Gemella bergeriae
707	Gemella cuniculi
708	Gemella sanguinis
709	Geminicoccus roseus
710	Geminocystis herdmanii
711	Geobacillus sp.
712	Geobacillus thermodenitrificans
713	Geobacter sp.
714	Geospiza fortis
715	Geothrix fermentans
716	Gillisia limnaea
717	Gillisia marina
718	Glaciecola sp.
719	Glomeribacter sp.
720	Gluconacetobacter sp.
721	Glycine max
722	Glycomyces sp.
723	Gordonia hirsuta
724	Gorilla gorilla
725	Gramella echinicola

726	Gramella forsetii
727	Gramella portivictoriae
728	Gryllotalpicola ginsengisoli
729	Haemophilus aegyptius
730	Haemophilus ducreyi
731	Haemophilus paraphrohaemolyticus
732	Haemophilus parasuis
733	Haemophilus somnus
734	Haemophilus sp.
735	Haemophilus sputorum
736	Haliangium ochraceum
737	Haliea salexigens
738	Haliscomenobacter hydrossis
739	Hallella seregens
740	Halobacteroides halobius
741	Halomonas sp.
742	halophilic archaeon
743	Halorhodospira halophila
744	Halotalea alkalilenta
745	Halothiobacillus neapolitanus
746	Hamadaea tsunoensis
747	Helcococcus kunzii
748	Helcococcus sueciensis
749	Helicobacter bizzozeronii
750	Helicobacter canadensis
751	Helicobacter canis
752	Helicobacter cetorum
753	Helicobacter pylori
754	Helicobacter rodentium
755	Helobdella robusta
756	Herminiimonas sp.
757	Heterocephalus glaber
758	Hippea alviniae
759	Hippea sp.
760	Hoeflea phototrophica
761	Holdemania filiformis
762	Homo sapiens
763	Humibacter albus
764	Hydra magnipapillata
765	Hymenobacter aerophilus
766	Ichthyophthirius multifiliis
767	Ideonella sp.
768	Ignatzschineria larvae
769	Ignavibacterium album
770	Ilyobacter polytropus
771	Intrasporangium calvum
772	Isoptericola variabilis
773	Ixodes scapularis
774	Jaculus jaculus
775	Janibacter sp.
776	Jeotgalicoccus marinus
777	Jiangella gansuensis
778	Johnsonella ignava
779	Jonquetella anthropi
780	Joostella marina
781	Kandleria vitulina
782	Ketogulonigenium vulgarum
783	Kingella denitrificans

784	Kingella kingae
785	Kitasatospora setae
786	Kitasatospora sp.
787	Klebsiella oxytoca
788	Klebsiella pneumoniae
789	Kocuria atrinae
790	Kocuria rhizophila
791	Kocuria sp.
792	Kordia algicida
793	Kribbella catacumbae
794	Kribbella flavida
795	Krokinobacter sp.
796	Kurthia huakuii
797	Kurthia sp.
798	Kyrpidia tusciae
799	Lachnoanaerobaculum umeaense
800	Lachnobacterium bovis
801	Lachnoclostridium phytofermentans
802	Lachnospira multipara
803	Lachnospiraceae bacterium
804	Lachnospiraceae oral
805	Lacinutrix sp.
806	Lacticigenium naphtae
807	Lactobacillus acidipiscis
808	Lactobacillus apodemi
809	Lactobacillus casei
810	Lactobacillus ceti
811	Lactobacillus crispatus
812	Lactobacillus equi
813	Lactobacillus gasseri
814	Lactobacillus hayakitensis
815	Lactobacillus iners
816	Lactobacillus jensenii
817	Lactobacillus johnsonii
818	Lactobacillus kefiranofaciens
819	Lactobacillus parafarraginis
820	Lactobacillus plantarum
821	Lactobacillus pobuzihii
822	Lactobacillus reuteri
823	Lactobacillus rhamnosus
824	Lactobacillus sakei
825	Lactobacillus salivarius
826	Lactobacillus vini
827	Lactococcus chungangensis
828	Lactococcus garvieae
829	Lactococcus lactis
830	Laribacter hongkongensis
831	Latimeria chalumnae
832	Leadbetterella byssophila
833	Lechevalieria aerocolonigenes
834	Leeia oryzae
835	Leeuwenhoekiella blandensis
836	Leeuwenhoekiella sp.
837	Legionella drancourtii
838	Legionella pneumophila
839	Leifsonia xyli
840	Leisingera nanhaiensis
841	Lepisosteus oculatus

842	Leptolyngbya boryana
843	Leptomyrmex pallens
844	Leptonychotes weddellii
845	Leptospira biflexa
846	Leptospira borgpetersenii
847	Leptospira kirschneri
848	Leucobacter chironomi
849	Leucobacter salsicius
850	Leuconostoc fallax
851	Leuconostoc kimchii
852	Leuconostoc mesenteroides
853	Lewinella cohaerens
854	Limnohabitans sp.
855	Lipotes vexillifer
856	Listeria grayi
857	Listeria monocytogenes
858	Listeria welshimeri
859	Loxodonta africana
860	Lyngbya majuscula
861	Lysinibacillus boronitolerans
862	Lysinibacillus fusiformis
863	Lysinibacillus sphaericus
864	Lysobacter antibioticus
865	Macaca fascicularis
866	Macaca mulatta
867	Macrococcus caseolyticus
868	Magnetospirillum gryphiswaldense
869	Magnetospirillum magneticum
870	Manacus vitellinus
871	Mannheimia granulomatis
872	Mannheimia haemolytica
873	Mannheimia succiniciproducens
874	Maribacter antarcticus
875	Maribacter sp.
876	Marinilabilia salmonicolor
877	Marinimicrobium sp.
878	Marinobacterium litorale
879	Marinobacterium rhizophilum
880	Marinobacterium stanieri
881	Marinococcus halotolerans
882	Marinomonas posidonica
883	Marinomonas sp.
884	Marivirga tractuosa
885	Marssonina brunnea
886	Massilia alkalitolerans
887	Mastigocoleus testarum
888	Medicago truncatula
889	Megachile rotundata
890	Megamonas funiformis
891	Megamonas hypermegale
892	Megamonas rupellensis
893	Meleagris gallopavo
894	Melissococcus plutonius
895	Melitea salexigens
896	Melopsittacus undulatus
897	Merops nubicus
898	Mesocricetus auratus
899	Mesoflavibacter zeaxanthinifaciens

900	Mesonia mobilis
901	Mesorhizobium ciceri
902	Mesorhizobium sp.
903	Metaseiulus occidentalis
904	Methanobrevibacter smithii
905	Methanobrevibacter sp.
906	Methanoculleus marisnigri
907	Methanohalobium evestigatum
908	Methanomassiliicoccus sp.
909	Methanomethylovorans hollandica
910	Methanosalsum zhilinae
911	Methanosphaerula palustris
912	Methylobacter tundripaludum
913	Methylobacterium populi
914	Methylobacterium sp.
915	Methylocaldum szegediense
916	Methylocapsa acidiphila
917	Methylococcus capsulatus
918	Methylocystis parvus
919	Methylomicrobium album
920	Methylomonas methanica
921	Methylophilus sp.
922	Methylopila sp.
923	Methylosarcina fibrata
924	Methylotenera mobilis
925	Methylotenera sp.
926	Methyloversatilis sp.
927	Microbacterium gubbeenense
928	Microbacterium indicum
929	Microbacterium maritypicum
930	Microbacterium sp.
931	Microbacterium testaceum
932	Microbulbifer agarilyticus
933	Micrococcus luteus
934	Microgenomates bacterium
935	Micromonospora purpureochromogenes
936	Microplitis demolitor
937	Microtus ochrogaster
938	Milkweed yellows
939	Mitsuokella sp.
940	Mobiluncus curtisii
941	Mobiluncus mulieris
942	Modestobacter marinus,
943	Monodelphis domestica
944	Monosiga brevicollis
945	Moraxella boevrei
946	Moraxella caprae
947	Moraxella catarrhalis
948	Moritella dasanensis
949	Mucilaginibacter paludis
950	Mucispirillum schaedleri
951	Mus musculus
952	Musca domestica
953	Mustela putorius
954	Mycobacterium avium
955	Mycobacterium fortuitum
956	Mycobacterium genavense
957	Mycobacterium parascrofulaceum

958	Mycobacterium sp.
959	Mycobacterium tuberculosis
960	Mycobacterium tusciae
961	Mycobacterium vaccae
962	Mycobacterium vanbaalenii
963	Mycoplasma agalactiae
964	Mycoplasma cloacale
965	Mycoplasma leachii
966	Mycoplasma leonicaptivi
967	Mycoplasma mycoides
968	Mycoplasma ovis
969	Mycoplasma primatum
970	Mycoplasma sp.
971	Mycoplasma testudinis
972	Myotis brandtii
973	Myotis davidii
974	Myotis lucifugus
975	Myroides injenensis
976	Myroides odoratimimus
977	Myroides odoratus
978	Myxococcus stipitatus
979	Naegleria gruberi
980	Nafulsella turpanensis
981	Nannospalax galili
982	Naumovozyma dairenensis
983	Nautilia profundicola
984	Neisseria bacilliformis
985	Neisseria lactamica
986	Neisseria macacae
987	Neisseria meningitidis
988	Neisseria polysaccharea
989	Neisseria shayeganii
990	Neisseria sp.
991	Neisseria wadsworthii
992	Nematostella vectensis
993	Neolamprologus brichardi
994	Niastella koreensis
995	Nitratifractor salsuginis
996	Nitratiruptor sp.
997	Nitrobacter winogradskyi
998	Nitrosomonas cryotolerans
999	Nitrosomonas europaea
1000	Nitrosospira briensis
1001	Nocardia farcinica
1002	Nocardia sp.
1003	Nocardioides alkalitolerans
1004	Nocardioides insulae
1005	Nocardioides sp.
1006	Nocardiopsis alba
1007	Nocardiopsis baichengensis
1008	Nocardiopsis gilva
1009	Nocardiopsis potens
1010	Nocardiopsis prasina
1011	Nomascus leucogenys
1012	Nonlabens dokdonensis
1013	Nonomuraea coxensis
1014	Nostoc punctiforme
1015	Novispirillum itersonii

1016	Novosphingobium aromaticivorans
1017	Oceanicola granulosus
1018	Oceanospirillum beijerinckii
1019	Oceanospirillum maris
1020	Ochotona princeps
1021	Ochrobactrum anthropi
1022	Ochrobactrum sp.
1023	Octadecabacter antarcticus
1024	Octodon degus
1025	Odobenus rosmarus
1026	Odoribacter sp.
1027	Odoribacter splanchnicus
1028	Oenococcus kitaharae
1029	Oerskovia turbata
1030	Olivibacter sitiensis
1031	Olleya marilimosa
1032	Olleya sp.
1033	Olsenella uli
1034	Opitutus terrae
1035	Orcinus orca
1036	Oreochromis niloticus
1037	Oribacterium sp.
1038	Ornithinibacillus scapharcae
1039	Ornithobacterium rhinotracheale
1040	Ornithorhynchus anatinus
1041	Orycteropus afer
1042	Oryctolagus cuniculus
1043	Oryza brachyantha
1044	Oryza sativa
1045	Oryzias latipes
1046	Oscillatoriales cyanobacterium
1047	Otolemur garnettii
1048	Ottowia thiooxydans
1049	Ovis aries
1050	Owenweeksia hongkongensis
1051	Oxalobacteraceae bacterium
1052	Paenibacillus ginsengihumi
1053	Paenibacillus graminis
1054	Paenibacillus pasadenensis
1055	Paenibacillus polymyxa
1056	Paenibacillus sanguinis
1057	Paenibacillus senegalensis
1058	Paenibacillus terrae
1059	Paenisporosarcina sp.
1060	Pan paniscus
1061	Pan troglodytes
1062	Pandoraea sp.
1063	Panthera tigris
1064	Pantholops hodgsonii
1065	Pantoea dispersa
1066	Pantoea vagans
1067	Papio anubis
1068	Parabacteroides distasonis
1069	Parabacteroides goldsteinii
1070	Parabacteroides gordonii
1071	Parabacteroides johnsonii
1072	Parabacteroides merdae
1073	Parabacteroides sp.

1074	Paracoccus denitrificans
1075	Paracoccus pantotrophus
1076	Paracoccus yeei
1077	Paraoerskovia marina
1078	Paraprevotella clara
1079	Paraprevotella xylaniphila
1080	Parascardovia denticolens
1081	Parcubacteria bacterium
1082	Parvibaculum lavamentivorans
1083	Pasteurella dagmatis
1084	Pasteurella multocida
1085	Pasteurella pneumotropica
1086	Patulibacter minatonensis
1087	Pectobacterium atrosepticum
1088	Pectobacterium carotovorum
1089	Pediculus humanus
1090	Pediococcus acidilactici
1091	Pedobacter agri
1092	Pedobacter arcticus
1093	Pedobacter borealis
1094	Pedobacter glucosidilyticus
1095	Pedobacter oryzae
1096	Pedobacter saltans
1097	Pedobacter sp.
1098	Pelistega sp.
1099	Pelodiscus sinensis
1100	Peptoclostridium difficile
1101	Peptostreptococcaceae bacterium
1102	Peptostreptococcus anaerobius
1103	Perkinsus marinus
1104	Peromyscus maniculatus
1105	Persephonella marina
1106	Phaeosphaeria nodorum
1107	Phascolarctobacterium sp.
1108	Phaseolus vulgaris
1109	Phoenix dactylifera
1110	Photobacterium damselae
1111	Photobacterium leiognathi
1112	Photorhabdus asymbiotica,
1113	Phycisphaera mikurensis
1114	Physcomitrella patens
1115	Physeter catodon
1116	Planctomyces limnophilus
1117	Planktothrix agardhii
1118	Planktothrix prolifica
1119	Plasmodium berghei
1120	Plasmodium cynomolgi
1121	Pleurocapsa sp.
1122	Poecilia formosa
1123	Poecilia reticulata
1124	Poinsettia branch-inducing
1125	Polaribacter sp.
1126	Polaromonas naphthalenivorans
1127	Pongo abelii
1128	Pontibacter roseus
1129	Populus trichocarpa
1130	Porphyromonas asaccharolytica
1131	Porphyromonas bennonis
1132	Porphyromonas cangingivalis
1133	Porphyromonas catoniae
1134	Porphyromonas gingivalis
1135	Porphyromonas gingivicanis
1136	Porphyromonas levii
1137	Porphyromonas macacae
1138	Porphyromonas somerae
1139	Porphyromonas sp.
1140	Porphyromonas uenonis
1141	Prauserella rugosa
1142	Prevotella albensis
1143	Prevotella amnii
1144	Prevotella aurantiaca
1145	Prevotella baroniae
1146	Prevotella bergensis
1147	Prevotella bivia
1148	Prevotella brevis
1149	Prevotella bryantii
1150	Prevotella buccae
1151	Prevotella copri
1152	Prevotella corporis
1153	Prevotella dentalis
1154	Prevotella dentasini
1155	Prevotella denticola
1156	Prevotella disiens
1157	Prevotella enoeca
1158	Prevotella falsenii
1159	Prevotella fusca
1160	Prevotella histicola
1161	Prevotella intermedia
1162	Prevotella loescheii
1163	Prevotella maculosa
1164	Prevotella marshii
1165	Prevotella micans
1166	Prevotella multisaccharivorax
1167	Prevotella nanceiensis
1168	Prevotella nigrescens
1169	Prevotella oralis
1170	Prevotella oulorum
1171	Prevotella pallens
1172	Prevotella pleuritidis
1173	Prevotella ruminicola
1174	Prevotella saccharolytica
1175	Prevotella scopos
1176	Prevotella shahii
1177	Prevotella sp.
1178	Prevotella stercorea
1179	Prevotella timonensis
1180	Prochlorococcus marinus
1181	Prochlorococcus sp.
1182	Prolixibacter bellariivorans
1183	Propionicicella superfundia
1184	Propionimicrobium lymphophilum
1185	Prosthecochloris aestuarii
1186	Proteiniphilum acetatigenes
1187	Proteobacteria bacterium
1188	Proteus mirabilis
1189	Providencia alcalifaciens

1190	Providencia rettgeri
1191	Providencia rustigianii
1192	Prunus mume
1193	Prunus persica
1194	Pseudoalteromonas flavipulchra
1195	Pseudobutyrivibrio ruminis
1196	Pseudoclavibacter soli
1197	Pseudogulbenkiania sp.
1198	Pseudomonas aeruginosa
1199	Pseudomonas alcaligenes
1200	Pseudomonas alcaliphila
1201	Pseudomonas avellanae
1202	Pseudomonas chloritidismutans
1203	Pseudomonas chlororaphis
1204	Pseudomonas corrugata
1205	Pseudomonas fuscovaginae
1206	Pseudomonas nitroreducens
1207	Pseudomonas phage
1208	Pseudomonas putida
1209	Pseudomonas sp.
1210	Pseudomonas stutzeri
1211	Pseudomonas syringae
1212	Pseudomonas thermotolerans
1213	Pseudonocardia acaciae
1214	Pseudonocardia dioxanivorans
1215	Pseudonocardia spinosispora
1216	Pseudoramibacter alactolyticus
1217	Pseudorhodobacter ferrugineus
1218	Pseudoxanthomonas sp.
1219	Psychrilyobacter atlanticus
1220	Psychrobacter phenylpyruvicus
1221	Psychrobacter sp.
1222	Psychroflexus torquis
1223	Psychroflexus tropicus
1224	Psychroserpens burtonensis
1225	Psychrosinus fermentans
1226	Pteropus alecto
1227	Pyrenophora teres
1228	Python bivittatus
1229	Ralstonia eutropha
1230	Ralstonia pickettii
1231	Ralstonia solanacearum
1232	Ralstonia sp.
1233	Ramlibacter tataouinensis
1234	Rattus norvegicus
1235	Rheinheimera perlucida
1236	Rhizobium etli
1237	Rhizobium larrymoorei
1238	Rhizobium leguminosarum
1239	Rhizobium sp.
1240	Rhizobium tropici
1241	Rhizobium undicola
1242	Rhodanobacter sp.
1243	Rhodobacter capsulatus
1244	Rhodobacter sphaeroides
1245	Rhodococcus equi
1246	Rhodococcus sp.
1247	Rhodopseudomonas palustris

1248	Rhodospirillum photometricum
1249	Rhodothermus marinus
1250	Ricinus communis
1251	Rickettsia helvetica
1252	Riemerella anatipestifer
1253	Riemerella columbina
1254	Rikenella microfusus
1255	Roseburia intestinalis
1256	Roseburia sp.
1257	Roseiflexus sp.
1258	Roseobacter sp.
1259	Roseovarius sp.
1260	Rothia aeria
1261	Rubrivivax gelatinosus
1262	Rudanella lutea
1263	Ruegeria conchae
1264	Ruminococcaceae bacterium
1265	Ruminococcus albus
1266	Ruminococcus flavefaciens
1267	Ruminococcus gnavus
1268	Ruminococcus obeum
1269	Ruminococcus sp.
1270	Ruminococcus torques
1271	Runella limosa
1272	Runella slithyformis
1273	Saccharopolyspora erythraea
1274	Saccharopolyspora spinosa
1275	Saccoglossus kowalevskii
1276	Saimiri boliviensis
1277	Salana multivorans
1278	Salinimicrobium terrae
1279	Salinimicrobium xinjiangense
1280	Salinispora arenicola
1281	Salinispora pacifica
1282	Salinivibrio siamensis
1283	Salisaeta longa
1284	Salmonella enterica
1285	Salpingoeca sp.
1286	Salsuginibacillus kocurii
1287	Sanguibacter keddieii
1288	Saprospira grandis
1289	SAR324 cluster
1290	Sarcophilus harrisii
1291	Schizophyllum commune
1292	Sebaldella termitidis
1293	Sediminibacter sp.
1294	Sediminibacterium salmoneum
1295	Sediminibacterium sp.
1296	Serinus canaria
1297	Serratia liquefaciens
1298	Serratia marcescens
1299	Serratia odorifera
1300	Serratia sp.
1301	Shewanella putrefaciens
1302	Shewanella sediminis
1303	Shimazuella kribbensis
1304	Shuttleworthia satelles
1305	Silanimonas lenta

1306	Simkania negevensis
1307	Simonsiella muelleri
1308	Simplicispira psychrophila
1309	Singularimonas variicoloris
1310	Sinorhizobium meliloti
1311	Sinorhizobium phage
1312	Slackia exigua
1313	Slackia heliotrinireducens
1314	Sodalis glossinidius
1315	Solanum lycopersicum
1316	Solanum tuberosum
1317	Sorex araneus
1318	Sorghum bicolor
1319	Spermophilus tridecemlineatus
1320	Sphingobacterium sp.
1321	Sphingobacterium spiritivorum
1322	Sphingobacterium thalpophilum
1323	Sphingobium sp.
1324	Sphingobium ummariense
1325	Sphingomonas sp.
1326	Sphingomonas wittichii
1327	Sphingomonas-like bacterium
1328	Sphingopyxis baekryungensis
1329	Spirochaeta cellobiosiphila
1330	Spirosoma luteum
1331	Sporocytophaga myxococcoides
1332	Sporolactobacillus inulinus
1333	Staphylococcus aureus
1334	Staphylococcus capitis
1335	Staphylococcus carnosus
1336	Staphylococcus epidermidis
1337	Staphylococcus haemolyticus
1338	Staphylococcus hominis
1339	Staphylococcus lentus
1340	Staphylococcus lugdunensis
1341	Staphylococcus pasteuri
1342	Staphylococcus saprophyticus
1343	Staphylococcus simulans
1344	Staphylococcus sp.
1345	Staphylococcus vitulinus
1346	Staphylococcus warneri
1347	Staphylococcus xylosus
1348	Stegastes partitus
1349	Stenotrophomonas maltophilia
1350	Stomatobaculum longum
1351	Streptobacillus moniliformis
1352	Streptococcus agalactiae
1353	Streptococcus castoreus
1354	Streptococcus constellatus
1355	Streptococcus didelphis
1356	Streptococcus dysgalactiae
1357	Streptococcus equi
1358	Streptococcus gallolyticus
1359	Streptococcus henryi
1360	Streptococcus hyovaginalis
1361	Streptococcus infantarius
1362	Streptococcus iniae
1363	Streptococcus intermedius
1364	Streptococcus lutetiensis
1365	Streptococcus marimammalium
1366	Streptococcus massiliensis
1367	Streptococcus merionis
1368	Streptococcus mutans
1369	Streptococcus oligofermentans
1370	Streptococcus orisratti
1371	Streptococcus ovis
1372	Streptococcus parauberis
1373	Streptococcus pasteurianus
1374	Streptococcus phage
1375	Streptococcus plurextorum
1376	Streptococcus prophage
1377	Streptococcus pseudopneumoniae
1378	Streptococcus pyogenes
1379	Streptococcus sobrinus
1380	Streptococcus sp.
1381	Streptococcus suis
1382	Streptococcus thoraltensis
1383	Streptococcus tigurinus
1384	Streptococcus uberis
1385	Streptococcus urinalis
1386	Streptococcus vestibularis
1387	Streptomyces auratus
1388	Streptomyces avermitilis
1389	Streptomyces bicolor
1390	Streptomyces bingchenggensis
1391	Streptomyces bottropensis
1392	Streptomyces cattleya
1393	Streptomyces davawensis
1394	Streptomyces globisporus
1395	Streptomyces griseus
1396	Streptomyces halstedii
1397	Streptomyces lavenduligriseus
1398	Streptomyces monomycini
1399	Streptomyces natalensis
1400	Streptomyces pristinaespiralis
1401	Streptomyces prunicolor
1402	Streptomyces rapamycinicus
1403	Streptomyces roseochromogenus
1404	Streptomyces sclerotialus
1405	Streptomyces seoulensis
1406	Streptomyces sp.
1407	Streptomyces varsoviensis
1408	Streptomyces xanthophaeus
1409	Strongylocentrotus purpuratus
1410	Subdoligranulum variabile
1411	Succinispira mobilis
1412	Succinivibrio dextrinosolvens
1413	Succinivibrionaceae bacterium
1414	Sulfurihydrogenibium subterraneum
1415	Sulfurospirillum arcachonense
1416	Sulfurospirillum cavolei
1417	Sus scrofa
1418	Sutterella wadsworthensis
1419	Synechococcus sp.
1420	Syntrophobotulus glycolicus
1421	Taeniopygia guttata

1422	Takifugu rubripes
1423	Tannerella forsythia
1424	Tarsius syrichta
1425	Tatumella ptyseos
1426	Tatumella sp.
1427	Taylorella equigenitalis
1428	Tenacibaculum maritimum
1429	Tenacibaculum ovolyticum
1430	Tenacibaculum sp.
1431	Tepidanaerobacter acetatoxydans
1432	Tepidiphilus margaritifer
1433	Teredinibacter turnerae
1434	Terracoccus sp.
1435	Terriglobus saanensis
1436	Terrimonas ferruginea
1437	Tetragenococcus muriaticus
1438	Tetrahymena thermophila
1439	Tetraleurodes acaciae
1440	Tetrapisispora blattae
1441	Tetrasphaera elongata
1442	Thalassobacillus devorans
1443	Thalassolituus oleivorans
1444	Thauera linaloolentis
1445	Thauera sp.
1446	Thauera terpenica
1447	Theobroma cacao
1448	Thermacetogenium phaeum
1449	Thermaerobacter marianensis
1450	Thermoanaerobacter brockii
1451	Thermoanaerobacter sp.
1452	Thermoanaerobacter wiegelii
1453	Thermoanaerobacterium saccharolyticum
1454	Thermodesulfobacterium geofontis
1455	Thermodesulfovibrio thiophilus
1456	Thermomonas fusca
1457	Thermophagus xiamenensis
1458	Thermosipho melanesiensis
1459	Thermotoga lettingae
1460	Thermotoga thermarum
1461	Thioalkalivibrio sp.
1462	Thioalkalivibrio sulfidophilus
1463	Thiobacillus denitrificans
1464	Thiocystis violascens
1465	Thioflavicoccus mobilis
1466	Thiomicrospira pelophila
1467	Thiomonas sp.
1468	Thiothrix disciformis
1469	Tolumonas sp.
1470	Toxoplasma gondii
1471	Tremella mesenterica
1472	Treponema bryantii
1473	Treponema maltophilum
1474	Treponema medium
1475	Treponema pallidum
1476	Treponema pedis
1477	Treponema primitia
1478	Tribolium castaneum
1479	Trichechus manatus

1480	Trichinella spiralis
1481	Trichophyton rubrum
1482	Trypanosoma cruzi
1483	Tupaia chinensis
1484	Turneriella parva
1485	Tursiops truncatus
1486	Uncultured bacterium
1487	Ureibacillus thermosphaericus
1488	Ursus maritimus
1489	Vaccinium witches'-broom
1490	Vanderwaltozyma polyspora
1491	Varibaculum cambriense
1492	Variovorax paradoxus
1493	Verminephrobacter aporrectodeae
1494	Verminephrobacter eiseniae
1495	Verrucomicrobiae bacterium
1496	Verrucosispora maris
1497	Vibrio breoganii
1498	Vibrio cholerae
1499	Vibrio cyclitrophicus
1500	Vibrio fischeri
1501	Vibrio furnissii
1502	Vibrio kanaloae
1503	Vibrio litoralis
1504	Vibrio mimicus
1505	Vibrio parahaemolyticus
1506	Vibrio splendidus
1507	Vibrio tasmaniensis
1508	Vicugna pacos
1509	Vitis vinifera
1510	Vitreoscilla stercoraria
1511	Weeksella virosa
1512	Weissella oryzae
1513	Weissella paramesenteroides
1514	Wigglesworthia glossinidia
1515	Wohlfahrtiimonas chitiniclastica
1516	Xanthomonas axonopodis
1517	Xanthomonas cassavae
1518	Xanthomonas sacchari
1519	Xenopus (Silurana)
1520	Xenorhabdus nematophila
1521	Xiphophorus maculatus
1522	Xylella fastidiosa
1523	Yersinia enterocolitica
1524	Yersinia phage
1525	Zavarzinella formosa
1526	Zea mays
1527	Zobellia galactanivorans
1528	Zunongwangia profunda
1529	Zymophilus raffinosivorans

Table S32 : The number of higher order connectivity nodes for each assembly dataset.

No	Dataset	Number of nodes having: two incoming edges and two outgoing edges	Number of nodes having: more than two incoming edges and more than two outgoing edges (higher order connectivity)	Percentage of higher order connectivity
1.	Order	2371	17	0.71
2.	Family	4013	15	0.37
3.	Genus	18624	56	0.30
4.	Species	24052	49	0.20
5.	MH0006	250830	10645	4.24
6.	MH0012	170284	7619	4.47
7.	MH0047	29972	669	2.23
8.	SRS017227	463942	7533	1.62
9.	SRS018661	136565	2694	1.97
			**Average**	**1.79**

The number of nodes having higher order connectivity and the number of nodes having two incoming edges and two outgoing edges are the number of nodes before aligned to the set of reference genomes.
